# Fifty years of Nordic social medicine and public health: snapshots of a journal

**DOI:** 10.1177/14034948221083108

**Published:** 2022-05-12

**Authors:** Peter Allebeck, Urban Janlert

**Affiliations:** 1 Department of Global Public Health, Karolinska Institutet, Sweden; 2 Department of Epidemiology and Public Health, Umeå University, Sweden

## Abstract

We revied articles published in the Scandinavian Journal of Public Health in a 50 years perspective. Papers reflect development of public health research, policy and debate over the years. Several papers describe early phases of Nordic population based studies that came to have major importance.

There are many ‘*Scandinavian journal of. . .*’. We found around 60 titles in a library database. Searching for ‘*Nordic Journal of. . .*’ we only found a little more than 10. This is in spite of the fact that all ‘*Scandinavian Journal of. . .*’ we know also have Finland and Iceland as partners. It seems as if Scandinavian seems to be a better brand name than Nordic, although Scandinavia usually refers only to a subset of the Nordic countries.

The *Scandinavian Journal of Public Health* (formerly *. . .Social Medicine*) is thus part of a large community that believes there are strong advantages and an international asset to work together under the umbrella of the term Scandinavian. In public health, research and analyses on the ‘Nordic welfare model’ is a central component in our work, so one might think that the Scandinavian journal concept would be of particular interest for us. The fact that there are so many other ‘Scandinavian journals’ shows that Nordic collaboration, and science and policy related to the ‘Nordic model’ or the ‘Nordic welfare states’ is of more general interest for many to capitalise on.

The timing of celebration is often difficult, as there are different views on how and when a certain phenomenon began. It is clear that the first issue of the *Scandinavian Journal of Social Medicine* was published in 1973. But a predecessor, *Acta Sociomedica Scandinavica*, had started already in 1969. Around 40 ‘*Acta. . . Scandinavica*’ still exist, and many are highly esteemed, but many have changed the name to ‘*Scandinavian Journal of. . .*’. One highly esteemed journal ‘*Acta Medica Scandinavica*’ abandoned the Scandinavian brand, and changed to ‘*Journal of Internal Medicine*’, and apparently increased their impact factor considerably.

From the Umeå University Library we have got access to titles and authors of all papers published in the journal throughout its existence (Supplemental Appendix 1). In this paper we present some statistics, and highlight trends we find interesting in the development of the journal. We have picked up some examples of papers from the different Nordic countries, mainly to show that influential authors and influential studies have been represented over the years. Citation analyses and bibliometric figures would of course be interesting, but databases are not reliable over such a long time span, and citation practices have also changed in general over the years.

## The hard facts

**Figure 1.**
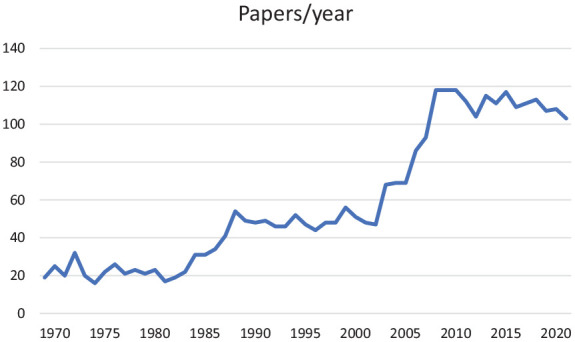
Number of papers published per year.

The number of manuscripts published during the years has increased from around 20 per year in the 1970s to around 120 per year in the past decade (Figure 1). The question is whether the journal just followed the general trend of more research and more publications, or has evolved differently compared to other public health journals. Data from other public health journals are difficult to find, but the general appreciation of the increase in scientific articles confirms that a six-fold increase in the number of publications throughout the period is a general phenomenon in the scientific world [1]. The plateau and even some decline during the past decade is more surprising, because other public health journals have continued to increase output.

Table I shows that the number of authors per paper has increased from 1.6 to 4.6 between 1970 and 2020, which is also consistent with the general trend in scientific publishing.

**Table I.** Statistics regarding the number of authors, articles and mean authors per article, range of the number of authors, number of papers with five or more authors and the number of issues for selected years.

	Year					
	1970	1980	1990	2000	2010	2020
Number of authors	39	51	111	149	443	506
Number of papers	25	23	48	50	118	109
Mean number of authors/paper	1.6	2.2	2.3	3.0	3.9	4.6
Range of number of authors	1–4	1–5	1–5	1–8	1–11	1–20
Number of papers with ⩾5 authors	0	1	3	8	34	51
Number of issues	3	3	4	4	8	8

## The early years

Themes of the papers of the early days were very much what we consider basic components of social medicine and public health: epidemiological investigations, screening projects, intervention studies, social aspects of disease, health services research, mental health problems, alcohol and drug use. A general impression is that more pure descriptive studies were published, that now would not be considered of general interest. It is of interest though that some publications are from persons and projects that were to become at the international forefront. Hannu Vuori had some papers on healthcare quality; he came to hold important posts at the World Health Organization (WHO) and the United Nations. Gudjon Magnusson had a paper on patients in the emergency department; he became director of the Nordic School of Public Health and then also had a leading position at the WHO. Dag Thelle had an early paper from the Tromsö study, that came to be a major project with important findings. Pekka Puska had an early paper on the ground-breaking and internationally well known North Karelia study; he also had leading international positions, for example, at the WHO. Several papers are from the population-based Gothenburg cohorts: men born in 1913, a project that is still alive and very productive, now into men born in 1963; a population-based study on women in Gothenburg, also still alive and very productive. Johannes Ipsen and Jørn Olsen, internationally renowned epidemiologists, published several methodological papers long before epidemiology became every person’s tool in public health.

## The 1990s

Looking at the list of papers during the 1990s the great bulk still originated in the Nordic countries. Some more European collaborative studies can be found, but few papers were submitted from other countries, some from European countries but very few from outside Europe. One general impression is though that papers during these years had a more analytical character, fewer descriptive studies, but follow-up studies, intervention studies, and more elaborate epidemiological methods in papers. Still though there were many papers from small-scale studies of more local public health interest.

Social security systems and disability pension had become an issue in the Nordic countries. The social security systems came under pressure with increasing rates of sickness absence, and the role of social insurance systems in balancing the individual’s capacity with workplace conditions became an important field of research. Social determinants of health had been highlighted in earlier studies, but more systematic studies on inequality in health and how social conditions determine disease outcome now started to appear in the *Scandinavian Journal of Social Medicine*, as well as in other international journals.

## The year 2000, social medicine becomes public health

The term social medicine was well established in many countries from the middle of the 20th century. At the end of the 20th century there was a general search for other terms, and various explanations can be given [2, 3]. Thus units, professorships and journals with the name ‘social medicine’ were renamed into community medicine or public health or some other similar term. As many of our Nordic departments and professorships of social medicine had also got other names, the board of the *Scandinavian Journal of Social Medicin*e started thinking about the name. This coincided with a change of editor, so when Professor Stig Wall, focusing on epidemiology and global health at Umeå University, was approached at the end of the 1990s and agreed to take over the role of editor, it was natural to take the name *Scandinavian Journal of Public Health*, following the orientation of the Umeå team. We do not remember any hard feelings or nostalgic complaints, but rather a general enthusiasm among members for a renewal and internationalisation of the journal.

While there was an ambition to introduce more of global health in the journal in the beginning of the 21st century, very few papers have originated from outside Nordic countries throughout the journal’s existence. There were indeed some papers dealing with health issues in low income countries, mostly originating from collaborations with other countries and supervision of PhD students from these lands. Very few papers have also originated from other European countries.

As seen in Figure 1, there was a considerable increase in the number of published articles in the first decade of the 21th century. There was also a great variety of themes covered. During this decade we found even more papers on social insurance matters as well as work and health in general. Working conditions, stress, demand-control, disability pension were high on the political as well as the public health agenda. The concept of ‘burn-out’ can be found in titles. Overweight and obesity, especially among children and youth was increasing, and a number of papers described trends in different groups, as well as intervention studies to increase the level of physical activity.

While a number of papers dealt with health policy in general and health services research, there seems to have been more interest in public health policy, including public health surveys, public health planning, and intervention studies at the population level. New cohorts were set up, and it is interesting to find an introductory paper by Jørn Olsen in 2001 on the Danish birth cohort, that now has generated lots of publications over the years, and served as a model for other birth cohorts.

## 2010 and onwards

During the past decade, we find a similar broad spectrum of public health-related topics as in previous years, but some topics appear more frequently: mental health, and especially mental health in youth and adolescence; migration and health; inequalities in health. Socioeconomic determinants are now often integrated in titles of many different health issues. Also health economic aspects are more often mentioned in titles. Gender-related papers can be found, but not as often as one could have expected. Some papers are found, for example, on gender-related violence, but compared to social determinants, gender is seldom mentioned in titles. Work and health continues to be an important topic in the journal; working conditions, as well as sickness absence and disability pension.

As in all other health journals, a totally new health problem appeared with many papers in 2021: COVID-19. Many public health colleagues reflected on whether we publish what we should be publishing [4]. How different societies have handled the pandemic, and which measures of control have been more or less adequate, remain major public health research questions that hopefully will be addressed in the Scandinavian journal in the coming years.

## Conclusions

The *Scandinavian Journal of Public Health*, and its predecessors, have been an important arena for sharing research and debate on social medicine and public health issues in the Nordic countries. In the list of authors identified, we find all persons who have been active in social medicine and public health throughout the years. Collaboration has expanded, and papers include authors and research topics from other European and low income countries, but few papers originate from other countries without Nordic collaborators. Titles of papers reflect all major topics of public health, but with an interesting variation over the years, illustrating what has been of particular interest in different time periods.

We congratulate the journal for having reached ‘maturity age’ and look forward to following the future development of the journal.

## Supplemental Material

sj-docx-1-sjp-10.1177_14034948221083108 – Supplemental material for Fifty years of Nordic social medicine and public health: snapshots of a journal Click here for additional data file. Supplemental material, sj-docx-1-sjp-10.1177_14034948221083108 for Fifty years of Nordic social medicine and public health: snapshots of a journal by Peter Allebeck and Urban Janlert in Scandinavian Journal of Public Health
