# Supplementary material for: Fifty years of Nordic social medicine and public health: snapshots of a journal
Source: Scand J Public Health. 2022 May 12;50(7):827–30. doi: 10.1177/14034948221083108 (PMC9578095; doi:10.1177/14034948221083108)
Supplement: sj-docx-1-sjp-10.1177_14034948221083108 – Supplemental material for Fifty years of Nordic social medicine and public health: snapshots of a journal [file sj-docx-1-sjp-10.1177_14034948221083108.docx]

# **Table of concents of Acta socio-medica Scandinavica (1969--1972) Scandinavian Journal of Social Medicine (1973--1998) Scandinavian Journal of Public Health (1999--Ꝏ)**

Colour codes for editors:

|  |  |  |  |  |  |  |
| --- | --- | --- | --- | --- | --- | --- |
| Gunnar Inghe | Ragnar Berfenstam | Lars Olov Bygren | Stig Wall | Finn Kamper-Jørgensen | Ingvar Karlberg | Terje Andreas Eikemo |

Scandinavian Journal of Public Health (

| **Year** | **Vol** | **No** | **Page** | **Author(s)** | **Title** |
| --- | --- | --- | --- | --- | --- |
| 1969 | 1 | 1 | 1-2 |  | Introduction |
| 1969 | 1 | 1 | 3-12 | Onni Kari-Koskinen, Leo Hirvonen | Smoking habits of university students |
| 1969 | 1 | 1 | 13-18 | Folke Pettersson | Smoking in pregnancy |
| 1969 | 1 | 1 | 19-31 | Bengt Lindegård, Sune Nyström | A prospective study of adult male psychiatric in- and out-patients in a Swedish urban population |
| 1969 | 1 | 1 | 33-41 | Léon Bjervig | Attempt at quantitative determination of the need for care by various groups of patients in the geriatric institution De Gamles By, Copenhagen |
| 1969 | 1 | 1 | 43-53 | Erik Brauer, Palle Hjermind and Bent Mackeprang | Need for care in the geriatric institution De garnies By, Copenhagen |
| 1969 | 1 | 1 | 55-60 | Odd Steffen Dalgard | Status inconsistency and functional psychoses in Oslo |
| 1969 | 1 | 2 | 61-68 | Arthur Engel and Gunnar Malmström | The Värmland trial, a mass health screening project |
| 1969 | 1 | 2 | 69-79 | Oleg Gorbatow, Sirkka-Liisa Aho and Tom Henrigson | Mortality and morbidity from myocardial infarction among men aged 30—64 within different social-, professional- and language-groups in the market town of Espoo in 1965 |
| 1969 | 1 | 2 | 81-93 | Victor Lindén | The distribution of sickness absences in a working population |
| 1969 | 1 | 2 | 95-106 | Kari Savonen | Finnish Student Health Service: Organization and forms of preventive work—Plans and practice |
| 1969 | 1 | 2 | 107-116 | Helge Kjems | Coordinated practical family therapy among multi-problem families |
| 1969 | 1 | 2 | 117-128 | Maj-Britt Inghe and Gunnar Inghe | Children of poor families in the welfare state |
| 1969 | 1 | 2 | 129-137 | Lars Olov Bygren | Social effects of Colles' fracture |
| 1969 | 1 | 3 | 139-143 | Anders Chr. Gogstad | Motivation problems in social medicine |
| 1969 | 1 | 3 | 145-164 | Ragnar Berfenstam, Dagmar Lagerberg and Björn Smedby | Victim characteristics in fatal home accidents: Alcohol, mental disorder, and suicidal intent in officially registered accidents in the home |
| 1969 | 1 | 3 | 165-172 | Leo Hirvonen and Onni Kari-Koskinen | The smoking habits of male industrial workers |
| 1969 | 1 | 3 | 173-182 | Victor Lindén | Sickness absences and social background |
| 1969 | 1 | 3 | 183-198 | Lars Olov Bygren | Socio-medical health screening |
| 1969 | 1 | 3 | 199-207 | Jaakko Suominen and Kai Sievers | The structure of private psychiatric practice in Finland |
| 1970 | 2 | 1 | 1-22 | Bengt Lindegård and Sune Nyström | Predisposition for mental illness: A prospective study of adult male psychiatric in- and out-patients in a Swedish urban population |
| 1970 | 2 | 1 | 23-27 | Onni Kari-Koskinen and Leo Hirvonen | Smoking among the female workers of a cotton mill |
| 1970 | 2 | 1 | 29-35 | Hannu Vuori | Quality control in hospitals |
| 1970 | 2 | 1 | 37-40 | Mauri Isokoski | Participants in a general health examination |
| 1970 | 2 | 1 | 41-44 | Oleg Gorbatow and Tom Henricson | Public health care in Finland |
| 1970 | 2 | 1 | 45-53 | Tage Egsmose | The Primary health services in a developing country |
| 1970 | 2 | 1 | 55-63 | Victor Lindén | Social causes of permanent disability |
| 1970 | 2 | 2/3 | 65 |  | INTRODUCTION |
| 1970 | 2 | 2/3 | 66-74 | Børge Stahl | The general practitioner's psychiatric and social therapeutic tasks |
| 1970 | 2 | 2/3 | 75-84 | Tor Bjerkedal | Norwegian health interview survey 1968: Preliminary results on morbidity and use of health services |
| 1970 | 2 | 2/3 | 85-94 | Ketty Kjærbye Kristensen | Married women with families, in their homes and on job: A social psychiatric family study |
| 1970 | 2 | 2/3 | 95-97 | Rolf Hanoa | An evaluation of medical and social needs in a sample of people in poor social circumstances |
| 1970 | 2 | 2/3 | 99-101 | Chr. F. Borchgrevink | Planning of the primary health service in Nord-Trøndelag |
| 1970 | 2 | 2/3 | 103-107 | Berthold Grünfeld | Some psychosocial aspects of hemophilia: A population study |
| 1970 | 2 | 2/3 | 109-115 | Erkki Eskelinen | Ability to work: Conceptual exposition and a method of measurement |
| 1970 | 2 | 2/3 | 117-126 | Harald Natvig | Sociomedical aspects of low back pain causing prolonged sick leave: A retrospective study |
| 1970 | 2 | 2/3 | 127-134 | Claes-Göran Westrin | Low back sick-listing: A nosological and medical insurance investigation |
| 1970 | 2 | 2/3 | 135-142 | Tapani Purola, Raimo Raitasalo and Antti Tamminen | From the quantitative needs for rehabilitation in Finland in the light of an interview survey and clinical examinations |
| 1970 | 2 | 2/3 | 143-147 | Jan Thorson | Incidence of disabled persons among traffic injured |
| 1970 | 2 | 2/3 | 149-159 | Jan Thorson, Hans Fredin, Peter Gerdman and Rune Wiklund | Accidental drowning among children in Sweden 1958—1967 |
| 1970 | 2 | 2/3 | 161-165 | Timo Sahi, Mauri Isokoski, J. Jussila and K. Launiala | Population surveys of lactose malabsorption: Preliminary report |
| 1970 | 2 | 2/3 | 167-176 | Hannu Vuori | Reliability of medical audit |
| 1970 | 2 | 2/3 | 177-192 | Sv. Heinild | Sex guidance considered from a social point of view |
| 1970 | 2 | 2/3 | 193-199 | Arne Vejlby | The organization of social services in Denmark |
| 1970 | 2 | 2/3 | 201-207 | Yngvar Løchen | Social reform in Norway |
| 1970 | 2 | 2/3 | 209-214 | Gunnar Inghe | Social reform in Sweden |
| 1971 | 3 | 1 | 1-16 | Michael D. Warren | The national health service and the planning of general practitioner services in England and Wales |
| 1971 | 3 | 2 | 17-26 | Ingvar Nylander | The feeling of being fat and dieting in a school population: An epidemiologic interview investigation |
| 1971 | 3 | 2 | 27-39 | Oleg Gorbatow, Tom Henricson, Sirkka-Liisa Aho, Pekka Kause and Markku Laulajainen | Coronary Heart Disease in Helsinki: A comparative investigation of the years 1955 and 1965 |
| 1971 | 3 | 2 | 41-49 | Carl Severin Albretsen and Per Vaglum | The Alcoholic's Wife and Her Conflicting Roles — a Cause for Hospitalization: A study of 12 consequtive families. A preliminary report |
| 1971 | 3 | 2 | 51-57 | Matti Virkkunen | Carl Severin Albretsen and Per Vaglum |
| 1971 | 3 | 2 | 59-68 | Hans Carlsen, Finn Christensen and Erik Holst | Drug consumption in Denmark: A pilot study |
| 1971 | 3 | 2 | 69-91 | Harold Sihm Jörgensen | The importance of environment in the aetiology of peptic ulcer with particular reference to iron-ore miners' conditions of employment and recruitment |
| 1971 | 3 | 3 | 93-104 | Arne Sund | Psychiatric disorders in male Norwegian youths |
| 1971 | 3 | 3 | 105-120 | Ulf Lucht | A prospective study of accidental falls and resulting injuries in the home among elderly people |
| 1971 | 3 | 3 | 121-123 | Hans Carlsen, Finn Christensen and Erik Holst | Stocks of drugs in Danish homes: A Pilot Investigation |
| 1971 | 3 | 3 | 125-139 | Victor Lindén | Consumers' and doctors' view of social services |
| 1971 | 3 | 3 | 141-142 | Bengt Pernow | Introduction: Symposium on Sociology in Medical Education |
| 1971 | 3 | 3 | 143-149 | J. Gallagher | Preparing a curriculum in the social sciences for medical students |
| 1971 | 3 | 3 | 151-155 | Per Bjurulf | The aims of teaching social and behavioural sciences in basic medical education |
| 1971 | 3 | 3 | 157-160 | Margot Jefferys | What is the purpose of teaching behavioural sciences in medical education? |
| 1971 | 3 | 3 | 161-167 | Yngvar Løchen | Sociology and medicine |
| 1971 | 3 | 3 | 169-174 | Johann Jürgen Rohde | Teaching sociology to medical students. Aspects of the situation in West Germany |
| 1971 | 3 | 3 | 175-182 | Gunnar Inghe | Need for sociology in social medical training |
| 1971 | 3 | 3 | 183-185 | Per Sundby | Need for sociology in social medical training |
| 1971 | 3 | 3 | 187-195 | Björn Smedby | The role of the sociologist in medical care research |
| 1972 | 4 | 1 | 1-8 | M. Virkkunen | On suicides committed by disability pensioners |
| 1972 | 4 | 1 | 9-12 | K. Leistner | Über soziale Auswirkungen rheumatischer Erkrankungen bei Arbeitern und Angestellten |
| 1972 | 4 | 1 | 13-24 | Arne Sund | Social and medical background data relating to psychiatric disorders of young Norwegian men |
| 1972 | 4 | 1 | 25-35 | Harald Persson | A study of the non-response group of an investigation into the conditions of living of pensioners |
| 1972 | 4 | 1 | 37-58 | Mona Britton | The consequences of informing relatives of intended autopsy in conjunction with death in hospital |
| 1972 | 4 | 2/3 |  |  | Introduction: Proceedings of the second regional meeting of the International Epidemiological Association -- Epidemiology and regional medical care planning, 21-23 May 1970 |
| 1972 | 4 | 2/3 | 61-63 |  | Participants |
| 1972 | 4 | 2/3 | 67 | Gunnar Lindgren | Introduction |
| 1972 | 4 | 2/3 | 69-70 | Bengt Jansson | Opening address |
| 1972 | 4 | 2/3 | 71-78 | Walter W. Holland and C. Watson | Uses of epidemiology in health services planning |
| 1972 | 4 | 2/3 | 79-86 | Maria Blohmke, G. Lindgren, Å. Nordén, G. Biörck and K. White | Contribution of an epidemiologist to medical care planning in West Germany |
| 1972 | 4 | 2/3 | 87-94 | Jiri Fodor, Gösta Tibblin and Lars Wilhelmsen | A cardiovascular control program in Gothenburg |
| 1972 | 4 | 2/3 | 95-105 | Gunnar Biörck and G. Tibblin | Epidemiology of ischemie heart disease and medical care planning |
| 1972 | 4 | 2/3 | 107-118 | Jørgen Fog, Johannes Mosbech and Jørgen Nyboe | Epidemiology of renal diseases and medical care planning: A study of 1966 deaths with renal disease/uremia in Denmark |
| 1972 | 4 | 2/3 | 119-124 | Hans Fritz and Bengt Scherstén | Screening of bacteriuria in planning for health care |
| 1972 | 4 | 2/3 | 125-130 | Johannes Ipsen | Epidemiology and planning |
| 1972 | 4 | 2/3 | 131-137 | Ole Horwitz | Epidemiology of tuberculosis in Denmark |
| 1972 | 4 | 2/3 | 139-142 | Åke Nordén, W. Holland and E. Krohn | Studies of the health problems within a community |
| 1972 | 4 | 2/3 | 143-151 | Kerr L. White | Epidemiologie intelligence requirements for planning personal health services |
| 1972 | 4 | 2/3 | 153-160 | John H. F. Brotherston and E. Krohn | Epidemiology and medical care planning |
| 1972 | 4 | 2/3 | 161-178 | Björn Smedby, W. Holland, J.-E. Spek, T. Bjerkedal and E. Holst | Demographic and socio-economic determinants of medical care use in Sweden and the United States |
| 1972 | 4 | 2/3 | 179-183 | Gillis Albinsson and C. Mellner | A planner's view on epidemiology |
| 1972 | 4 | 2/3 | 185-188 | A. S. Härö | A planner's view on epidemiology |
| 1972 | 4 | 2/3 | 189-192 | Tor Bjerkedal, L. Köhler and J. Brotherston | Health services planning among pre-school children |
| 1972 | 4 | 2/3 | 193-194 | Per Langeland, W. Holland and J. Ipsen | Screening for cancer of the breast in Malmö |
| 1972 | 4 | 2/3 | 195-200 | Per Bjurulf, J. Brotherston, G. Lindgren and K. White | Consumption of resources in out-patient care within a Swedish county |
| 1972 | 4 | 2/3 | 201-202 | Erik Allander | A population study of rheumatoid arthritis in Stockholm |
| 1972 | 4 | 2/3 | 203 | Lars Olov Bygren | Needs of medical and social services – a population survey |
| 1972 | 4 | 2/3 | 205 | Isacsson (rapporteur) | Group discussions I. Preventive cardiology and regional medical care planning |
| 1972 | 4 | 2/3 | 205-206 | Bengtsson (rapporteur) | Group discussions II. Research in community health and medical care planning |
| 1972 | 4 | 2/3 | 206 | Smedby (rapporteur) | Group discussions III. The epidemiologist, the computer and the planning team |
| 1972 | 4 | 2/3 | 206-207 | Horwitz (rapporteur) | Group discussions IV. Epidemiological departments in main hospitals |
| 1972 | 4 | 2/3 | 207-208 | Smedby (rapporteur) | Group discussions V. Epidemiology and planning |
| 1972 | 4 | 2/3 | 208-209 | White (rapporteur) | Group discussions VI. Planning and epidemiology |
| 1973 | 1 | 1 | 1-2 | Gunnar Inghe | Introduction |
| 1973 | 1 | 1 | 3-6 | Rolf Eriksson, Hans Fredin, Peter Gerdman and Jan Thorson | Sequelae of Accidental Near-drowning in Childhood |
| 1973 | 1 | 1 | 7-12 | Carl S. Albretsen and Per Vaglum | The Alcoholic's Wife and Her Conflicting Roles: II. A Follow-up Study |
| 1973 | 1 | 1 | 13-16 | Svend Heinild | Health and the Population: Structure and Substance |
| 1973 | 1 | 1 | 17-24 | Esko Kalimo and Thomas W. Bice | Causal Analysis and Ecological Fallacy in Cross-national Epidemiological Research |
| 1973 | 1 | 1 | 25-31 | Harald Persson | A Mail Inquiry into Pensioners' Living Conditions: Comparison between Response Group and Non-response Group |
| 1973 | 1 | 1 | 33-38 | J. H. Abramson, L. M. Epstein, Sidney L. Kark, Emily Kark and B. Fischler | The Contribution of a Health Survey to a Family Practice |
| 1973 | 1 | 2 | 41-43 | Poul Bonnevie | The Concept of Health: A Socio-medical Approach |
| 1973 | 1 | 2 | 45-51 | Lars Molin | Socio-medical Aspects on Previously Hospitalized Psoriatics in a Metropolitan Area |
| 1973 | 1 | 2 | 53-58 | Sune Nyström, Christer Olsfelt, Carl-Gunnar Eriksson and Tryggve Andén | Suicide in Gothenburg: Demographic and Social Factors and Availment of Care of the Suicide Cases in 1968, with Some Views on Prevention |
| 1973 | 1 | 2 | 59-68 | Leif Arne Heløe | Distribution and Clustering of Social Problems in an Aided Population Group |
| 1973 | 1 | 2 | 69-75 | Per Vaglum | The Patient-centred Family Working Group - a Medium for Collaboration with the "Unmotivated" Family Members: A Model and an Example |
| 1973 | 1 | 2 | 77-79 | Victor Lindén | Medicine and the Behavioral Sciences |
| 1973 | 1 | 3 | 81-90 | Mona Britton | Should Relatives Be Informed That Autopsy Is intended? Opinions of Relatives with Recent Experience |
| 1973 | 1 | 3 | 91-95 | Leif Svanström and Viveca Urwitz | Sociomedical Development Work at a Social Welfare Office In a Metropolitan Area: A Preliminary Report |
| 1973 | 1 | 3 | 97-108 | Erik Allander | Diagnostic Process and Rheumatic Diseases |
| 1973 | 1 | 3 | 109-113 | Jan Thorson | Injury Diagnoses within Morbidity Statistics |
| 1973 | 1 | 3 | 115-123 | Claes-Göran Westrin | Cooperation between Medical and Social Services |
| 1973 | 1 | 3 | 125-131 | Olle Hagnell, Eberhard Nyman and Kerstin Tunving | Dangerous Alcoholics: Personality Varieties in Aggressive and Suicidally Inclined Subjects |
| 1973 | 1 | 3 | 133-147 | Claes Sundelin | Parents' Experiences of Child Health Centers: A Study in the Urban District of Uppsala |
| 1974 | 2 | 1 | 1-3 | K. Reiter | Mortality and Causes of Death in Men Found to be Mentally Abnormal by Forensic Psychiatrists |
| 1974 | 2 | 1 | 5-11 | Asbjörn Medhus | Morbidity among Female Alcoholics |
| 1974 | 2 | 1 | 13-21 | Pekka Sakki, Pentti Sorri and Matti Virkkunen | On Factors Complicating Rehabilitation of Psychiatric Patients |
| 1974 | 2 | 1 | 23-35 | Claes-Göran Westrin | The Reliability of Auto-anamnesis: A Study of Statements Regarding Low Back Trouble |
| 1974 | 2 | 1 | 37-46 | Erik Allander | Distribution of Doctor's Consultations, Operations, and Symptoms with Special Relevance to Social Factors: Report on a Swedish Nation-Wide Health Survey |
| 1974 | 2 | 2 | 49-65 | Lars Olov Bygren | The Driver's Exposure to Risk of Accident |
| 1974 | 2 | 2 | 67-77 | Hans Fredin, Peter Gerdman and Jan Thorson | Industrial Accidents in the Construction Industry |
| 1974 | 2 | 2 | 79-85 | Elina Hemminki | General Practitioners' Indications for Psychotropic Drugs |
| 1974 | 2 | 2 | 87-91 | Bengt Lindegård and Sune Nyström | Short-term Psychiatric Care from a Theoretical Planning and Empirical Point of View |
| 1974 | 2 | 2 | 93-97 | Jens Danielsson, Göran Pettersson, Inge Hesselius and Björn Stenkvis | Computer System for Gynaecological Health Control |
| 1974 | 2 | 2 | 99-104 | Nils Bejerot and Carol Maurice-Bejerot | Methods of Studying Prevalence and Incidence of Drug Abuse |
| 1974 | 2 | 3 | 105-111 | Pekka Sakki, Pentti Sorri and Matti Virkkunen | On the Recommendation of Rehabilitative Measures for Psychiatric Patients: A Factor-analytic Study |
| 1974 | 2 | 3 | 113-120 | Leif Svanström | Falls on Stairs: an Epidemiological Accident Study |
| 1974 | 2 | 3 | 121-128 | Jan Thorson | Pedal Cycle Accidents: With special reference to the prevention of injuries caused by falls |
| 1974 | 2 | 3 | 129-134 | Elina Hemminki | Diseases Leading to Psychotropic Drug Therapy |
| 1974 | 2 | 3 | 135-140 | Leena Räsänen, Antti Ahlström and Matti Rimpelä | Pretesting the Channels of Distribution for a Nutrition Education Leaflet |
| 1975 | 3 | 1 | 1-3 | H. G. Lilius, Erkki J. Valtonen and S. G. Jokipii | Factors Interfering with the Comfort of Old People in Homes for the Aged |
| 1975 | 3 | 1 | 5-11 | Jens Sande and Jan Thorson | An Evaluation of the Officiai Swedish Statistics on Seriously Injured in Road Traffic Accidents |
| 1975 | 3 | 1 | 13-17 | E. Eriksson, E. Allander, B. Andersson and J. Landegren | Varicose Ulcers: A Study of Medical Results and Patients' Assessment of the Outcome of a Combined Dermatological and Surgical Treatment: Some Preliminary Results |
| 1975 | 3 | 1 | 19-22 | Tom Andersen | Organisational Structure and its Effect on the Therapeutic Activity and Effectiveness in an American Mental Health Center |
| 1975 | 3 | 1 | 23-27 | Asbjörn Medhus | Conviction for Drunkenness—A Late Symptom among Female Alcoholics |
| 1975 | 3 | 1 | 29-33 | Asbjörn Medhus | Venereal Diseases among Female Alcoholics |
| 1975 | 3 | 1 | 35-39 | B. Stenkvist, J. Danielsson and G. Pettersson | A Computer Based System for Gynecological Cytology |
| 1975 | 3 | 1 | 41-44 | Søren Andersen, Poul Krebs Lange and Gunnar Rønn | Socio-medical Follow-up Examination of 1 143 Greenlanders Surgically Treated for Pulmonary Tuberculosis at Queen Ingrid's Hospital at Godthåb in the Years 1954-1966 |
| 1975 | 3 | 2 | 45-49 | Asbjörn Medhus | Criminality among Female Alcoholics |
| 1975 | 3 | 2 | 51-60 | Erik Allander and Urban Rosenqvist | Screening—an Efficient Tool in Outpatient Endocrine Care Studies of the Diagnostic Process |
| 1975 | 3 | 2 | 61-67 | Antti Penttilä and Antti Ahonen | Arteriosclerotic and Other Degenerative Heart Diseases in Finland. I. A Death Certificate Study of the Frequency of Degenerative Heart Diseases among Males and Females |
| 1975 | 3 | 2 | 69-74 | Antti Penttilä and Antti Ahonen | Arteriosclerotic and Other Degenerative Heart Diseases in Finland: II. A Death Certificate Study of the Examination of the Cause of Death from Degenerative Heart Diseases |
| 1975 | 3 | 2 | 75-82 | H. Johansson, Å. Rimsten, B. Stenkvist, B. Stenqvist and J. Danielsson | Organization of a Breast Tumour Clinic and Aspects of Data Analysis of a Clinical Material |
| 1975 | 3 | 2 | 83-85 | Victor Lindén | Vitamin D and serum cholesterol |
| 1975 | 3 | 2 | 87-92 | Elina Hemminki and Jorma Heikkilä | Elderly People's Compliance with Prescriptions, and Quality of Medication |
| 1975 | 3 | 2 | 93-101 | Sven-Olof Isacsson, Sven-Eric Lindell, Torsten Carle and Robert O. Malmborg | Detection, Treatment and Follow-up of Hypertension in a Community Sample of 55-Year-Old Men |
| 1975 | 3 | 3 | 105-110 | E. Heikkinen, B. Seppänen and M. Rimpelä | Biological Age, Health, and Health-risk Indicators among 25-57-year-old Men in Two Parts of Finland |
| 1975 | 3 | 3 | 111-115 | Asbjörn Medhus | Mortality among Female Alcoholics |
| 1975 | 3 | 3 | 117-128 | Erik Allander and Urban Rosenqvist | The Diagnostic Process in Outpatient Endocrine Care with Special Reference to Screening. Further Study of Diagnostic Patterns, Their Changes, Total and Diagnosis-specific Resource Consumption |
| 1975 | 3 | 3 | 129-138 | Inge Hesselius, Hans-Olof Lisper, Ann Nordström, Birgitta Anshelm-Olson and Britta Ödlund | Comparison between Participants and Non-participants at a Gynaecological Mass Screening |
| 1975 | 3 | 3 | 139-142 | C. W. Smiley | The Flower Children of Sudbury: The Story of a Street Clinic for Hippies |
| 1975 | 3 | 3 | 143-146 | Asbjörn Medhus | Female Alcoholics and Public Assistance |
| 1976 | 4 | 1 | 1-6 | Elina Hemminki, Kari Hemminki, Timo Hakulinen and Matti Hakama | Increase in Years of Life after Eliminating Causes of Death: Significance for Health Priorities |
| 1976 | 4 | 1 | 7-12 | Erik Allander | Do You Die from Rheumatism? The Five-Year Mortality in a Middle-Aged Population Sample with Respect to Reported Joint Symptoms |
| 1976 | 4 | 1 | 13-20 | Timo Niemi | Hospital treatment of Addicts Arrested for Intoxication in Finland |
| 1976 | 4 | 1 | 21-23 | Paavo Moilanen, Leo Hirvonen, Jouni Timisjärvi and Onni Kari-Koskinen | Smoking and the Subjective Health Condition among Finnish Military Conscripts |
| 1976 | 4 | 1 | 25-29 | Sven-Olof Isacsson and Lars Janzon | Results of a Quit-smoking Research Project in a Randomly Selected Population |
| 1976 | 4 | 1 | 31-40 | Erik Allander and Eva Håkanson | The Layman's Medical Vocabulary: A Study of Illness Definitions and Perceived Need for Medical Care |
| 1976 | 4 | 1 | 41-44 | H. G. Lilius, Erkki J. Valtonen and Juhani Wikström | Sexual Problems in Patients Suffering from Multiple Sclerosis |
| 1976 | 4 | 1 | 34-51 | Onni Kari-Koskinen and Pentti Karvonen | Mental Health and Housing |
| 1976 | 4 | 2 | 53-56 | Johs. Ipsen | Priorities in Epidemiology of the Future |
| 1976 | 4 | 2 | 57-60 | Pekka Puska, Kaj Koskela, Hilkka Pakarinen, Pirjo Puumalainen, Väinö Soininen and Jaakko Tuomilehto | The North Karelia Project: a Programme for Community Control of Cardiovascular Diseases |
| 1976 | 4 | 2 | 61-66 | Töres Theorell, Alf Askergren, Alice Olsson and Torbjörn Åkerstedt | On Risk Factors for Premature Myocardial Infarction in Middle-aged Building Construction Workers: A Comparison with other Selected Illnesses |
| 1976 | 4 | 2 | 67-70 | Jaakko Tuomilehto, Pekka Puska and Aulikki Nissinen | Hypertension Programme of the North Karelia Project |
| 1976 | 4 | 2 | 71-74 | Eino Heikkinen, Birgit Käyhty-Seppänen and Pertti Pohjolainen | Health Situation and Related Social Conditions among 66-Year-Old Finnish Men |
| 1976 | 4 | 2 | 75-83 | Anders Bolin, Bengt Lindström, Alf Sagnér and Leif Svanström | On Pensioners' Circumstances in a Large Swedish City: A Report from Malmö |
| 1976 | 4 | 2 | 85-89 | Timo Niemi, Seppo Sarna and Seppo Kovalainen | Retirement and Psychic Investments |
| 1976 | 4 | 2 | 91-101 | Michael D. Warren | Medical Records in Family Practice: A Review |
| 1976 | 4 | 2 | 103-107 | Per G. Hansson | Sick-leave after Road Traffic Accidents |
| 1976 | 4 | 3 | 109-114 | Karl Evang | Health and Disease Concepts |
| 1976 | 4 | 3 | 115-122 | Ulf de Faire and Töres Theorell | Life Changes and Myocardial Infarction: How Useful Are Life Change Measurements? |
| 1976 | 4 | 3 | 123-129 | Urban Waern, Hans Hedstrand and Hans Åberg | What Middle-aged Men Know of Their Parents' Cause of Death and Age at Death: A Comparison between History and Death Certificate |
| 1976 | 4 | 3 | 131-133 | John Ivar Brevik and Nils Indrevoll | Visits to Small Coastal Communities of Northern Norway by a Doctor and a Health Insurance Officer |
| 1976 | 4 | 3 | 1135-140 | P. S. Parfrey | The Effect of Religious Factors on Intoxicant Use |
| 1976 | 4 | 3 | 141-143 | Asbjörn Medhus and Holger Hansson | Alcohol Problems among Female Gonorrhoea Patients |
| 1976 | 4 | 3 | 145-149 | Asbjörn Medhus | Alcohol Problems among Male Disability Pensioners |
| 1976 | 4 | 3 | 151-157 | Anders Bolin, Bengt Lindström, Alf Sagnér and Leif Svanström | Services to Pensioners and How They Might Be Improved: A Report from Malmö |
| 1976 | 4 | 3 | 159-162 | Asbjörn Medhus | Men with Disability Pension |
| 1977 | 5 | 1 | 1-3 | Claes Sundelin and Ragnar Berfenstam | Child Health in Sweden |
| 1977 | 5 | 1 | 5-13 | Lars H. Gustafsson | Childhood Accidents: Three Epidemiological Studies on the Etiology |
| 1977 | 5 | 1 | 15-19 | Sven Bremberg | Pregnancy in Swedish Teenagers: Perinatal Problems and Social Situation |
| 1977 | 5 | 1 | 21-30 | Johnny Ludvigsson | Diabetics in School: Knowledge and Attitudes of School Staff in Relation to Juvenile Diabetics |
| 1977 | 5 | 1 | 31-39 | Lorentz M. Irgens, Siri Nome Eikhom and Tor Bjerkedal | Maladjusted Children and the School Health Services: Development of a Screening Test for Routine Use |
| 1977 | 5 | 1 | 41-53 | Björn Jonsson and Irma Åstrand | Electrocardiographic Findings in Men and Women aged 18 to 65 |
| 1977 | 5 | 2 |  | Gunnar Lindgren and Ragnar Berfenstam | [Introduction – The death of Gunnar Inghe] |
| 1977 | 5 | 2 | 57-60 | Sv. Heinild | Therapy: A Critique of Virginia Satir |
| 1977 | 5 | 2 | 61-65 | Bent Mackeprang and Erik Brauer | Mortality and Functional Prognosis in a Geriatric Population |
| 1977 | 5 | 2 | 67-72 | Bent Mackeprang and Erik Brauer | Changes in Age and Need for Care among Patients in a Geriatric Institution During a Two-Year Period |
| 1977 | 5 | 2 | 73-75 | Asbjörn Medhus and Hans Kristenson | Mortality among Male Disability Pensioners |
| 1977 | 5 | 2 | 77-83 | Kirsti Selander | Gynaecological State of Health of 2654 Women in Nurmes 1970-71: A Clinical Outpatient Study |
| 1977 | 5 | 2 | 85-90 | Ottar Tangen | Medical Ethics and Child Abuse |
| 1977 | 5 | 2 | 91-95 | Risto Honkanen | Records based on Clinical Examination as an Indicator of Alcohol Involvement in Injuries at Emergency Stations |
| 1977 | 5 | 2 | 97-103 | Lars Dahlin | Standard of Living, Health, and Utilization of Social and Health Services A Survey of the Population of Three Districts in Malmö |
| 1977 | 5 | 3 | 105-114 | Elina Hemminki and Terttu Pesonen | The Function of Drug Company Representatives |
| 1977 | 5 | 3 | 115-121 | Kari Poikolainen | Drug Poisoning Mortality Trends in the Scandinavian Countries 1961-1973 |
| 1977 | 5 | 3 | 123-125 | V. Lindén, J. I. Brevik and T. Hansen | Phenytoin, Phenobarbitone and Serum Cholesterol |
| 1977 | 5 | 3 | 127-135 | Leif-Edvard Aarø, Kjell Bjartveit, Odd D. Vellar and Else-Lill Berglund | Smoking Habits among Norwegian Doctors 1974 |
| 1977 | 5 | 3 | 137-140 | Olof Lannerstad, Nils-Herman Sternby, Sven-Olof Isacsson, Gunnar Lindgren and Sven-Eric Lindell | Effects of a Health Screening on Mortality and Causes of Death in Middle-aged Men: A prospective study from 1970 to 1974 of men in Malmö, born 1914 |
| 1977 | 5 | 3 | 141-144 | Bernard S. Bloom, Egon Jonsson and Marie-Louise Dolk | Utilization of Coronary Care Units in Sweden |
| 1978 | 6 | 1 | 1-3 | Poul Bonnevie | The Evolution and Essence of Epidemiology |
| 1978 | 6 | 1 | 5-6 | Finn Kamper-Jørgensen | Plans for Epidemiologic Research in Primary Health Care in Denmark |
| 1978 | 6 | 1 | 7-16 | Hans Berglind | Early Retirement Pensions in Sweden: Trends and Regional Variations |
| 1978 | 6 | 1 | 17-24 | Erik Brauer, Bent Mackeprang and Michael Weis Bentzon | Prognosis of Survival in a Geriatric Population |
| 1978 | 6 | 1 | 25-29 | Bent Mackeprang and Michael Weis Bentzon | Minimum Need for Care in Geriatric Institutions |
| 1978 | 6 | 1 | 31-35 | Adam Taube and Stig Wall | A Model for the Estimation of Change in Knowledge between Two Interview Occasions with Unreliable Responses |
| 1978 | 6 | 1 | 37-41 | Tapio Videman and Jorma Heikkilä | Frequent Users of Doctor Services in a Small Rural Community |
| 1978 | 6 | 1 | 43-48 | Göran Aurelius and Elsa Ryde-Blomqvist | Pregnancy and Delivery among Immigrants |
| 1978 | 6 | 2 | 49-54 | Calle Bengtsson, Leif Hallberg, Tore Hällström, Arvid Hultborn, Björn Isaksson, Jan Lennartsson, Olof Lindquist, Sven Lindstedt, Henry Noppa, Lars Redvall and Sverker Samuelsson | The Population Study of Women in Göteborg 1974-1975—the Second Phase of a Longitudinal Study: General Design, Purpose and Sampling Results |
| 1978 | 6 | 2 | 55-58 | Adesuwa C. Emovon and Thomas Adeoye Lambo | A Survey of Criminal Homicide in Nigeria |
| 1978 | 6 | 2 | 59-62 | Bent Mackeprang and Michael Weis Bentzon | Some Aspects Concerning Admittance to and Accommodation in Geriatric Institutions Housing Persons with Varying Degrees of Need for Care |
| 1978 | 6 | 2 | 63-67 | Bent Mackeprang and Michael Weis Bentzon | Minimum Variability in the Need for Care in Geriatric Institutions |
| 1978 | 6 | 2 | 68 | E. Brauer, B. Mackeprang and M. Benzton | ERRATUM: Prognosis of Survival in a Geriatric Population |
| 1978 | 6 | 2 | 69-77 | Finn Olav Kinge and Tor Bjerkedal | Work and Disability at the Age of 30 Years: A Sociomedical Study of a Birth-cohort from Bergen I. Description of Project and Methods |
| 1978 | 6 | 2 | 79-83 | Finn Olav Kinge | Work and Disability at the Age of 30 Years: A Sociomedical Study of a Birth-cohort from Bergen II. Frequency of disability |
| 1978 | 6 | 2 | 85-95 | Kurt Svärdsudd and Nils Blomqvist | A New Method for Investigating the Relation between Change and Initial Value in Longitudinal Blood Pressure Data: I. Description and Application of the Method |
| 1978 | 6 | 3 | 97-104 | Bernard S. Bloom and Egon Jonsson | Distributing Medical Care Services: Coronary Care Units in the United States and Sweden |
| 1978 | 6 | 3 | 105-109 | Jorma Takala, Pekka Kopteff, Anneli Takala and Kai Sievers | Use of Physician Services by a Middle-aged Population in a Rural Health Centre District in Southwest Finland: Descriptive Distributions |
| 1978 | 6 | 3 | 111-115 | Marja Ekblom, Olavi Elo, Juhani Laurinkari and Pauli Niemelä | Costs and Benefits of Measles Vaccination in Finland |
| 1978 | 6 | 3 | 117-124 | Pål Hartvig | Untreated Psychiatric Disability: A Study of Disabled Persons with Major Psychiatric Health Impairment, Having Never Received Psychiatric Treatment |
| 1978 | 6 | 3 | 125-129 | Nils Blomqvist and Kurt Svärdsudd | A New Method for Investigating the Relation between Change and Initial Value in Longitudinal Blood Pressure Data: II. Comparison with Other Methods |
| 1978 | 6 | 3 | 131-136 | Finn Olav Kinge | Work and Disability at the Age of 30 Years: A Sociomedical Study of a Birth-cohort from Bergen |
| 1978 | 6 | 3 | 137-143 | Markku Koskenvuo, Seppo Sarna and Jaakko Kaprio | Mortality by Marital Status and Social Class in Finland during 1969-1971: Mortality from Natural and Violent Causes |
| 1979 | 7 | 1 | 1-6 | Jørn Olsen and Svend Sabroe | Researching Occupational Mortality: The Problem of Comparison |
| 1979 | 7 | 1 | 7-15 | R. Andersson, B. Johansson, K. Lindén, K. Svanström and L. Svanström | What Part Does the Occupational Environment Play in the Occurrence of Accidents? Views of Accident Victims, Their Fellow-workers, Safety Delegates, and Enterprise Managements |
| 1979 | 7 | 1 | 17-25 | Finn Olav Kinge | Work and Disability at the Age of 30 Years: A Sociomedical Study of a Birth-cohort from Bergen: IV. Economic activity, occupation, and earned income |
| 1979 | 7 | 1 | 27-32 | Helena Simonen, Antti Apajalahti, Liisa Ferm and Lars Emil Tötterman | The Long-Term Treatment of the Aged: An Assessment Method |
| 1979 | 7 | 1 | 33-39 | D. Bergqvist and H. Hedelin | Trends in Blunt Abdominal Trauma among Hospital In-patients: Developments in a Swedish Rural District over 30 Years |
| 1979 | 7 | 1 | 41-47 | Olof Lannerstad, Sven-Olof Isacsson and Sven-Eric Lindell | Risk Factors for Premature Death in Men 56-60 Years Old: A Prospective Study of Men Born 1914, Living in Malmö, Sweden |
| 1979 | 7 | 2 | 49-54 | Jørn Olsen and Svend Sabroe | Mortality among Bricklayers and Carpenters/cabinet makers |
| 1979 | 7 | 2 | 55-62 | Sisko Asp, Sven Hernberg and Yrjö Collan | Mortality among Finnish Doctors, 1953-1972 |
| 1979 | 7 | 2 | 63-65 | Timo Niemi | Effect of Loneliness on Mortality After Retirement |
| 1979 | 7 | 2 | 67-72 | Anders Ahlbom and Rolf Nordlander | Application of Diagnostic Criteria in the Diagnosis of Myocardial Infarction |
| 1979 | 7 | 2 | 73-78 | Dagmund Svendsen and Finn Olav Kinge | Work and Disability at the Age of 30 Years: A Sociomedical Study of a Birth-cohort from Bergen V. Social background and recruitment to the school system |
| 1979 | 7 | 2 | 79-85 | Irma Soini and Matti Hakama | Inverse Association Between Risk Factors for Benign and Malignant Breast Lesions |
| 1979 | 7 | 2 | 87-91 | Jorma Takala, Kai Sievers and Anneli Takala | A Multiphasic Screening Programme at the Health Centre Level: the Säkylä-Köyliö Project: Variables, Methods and Participation |
| 1979 | 7 | 2 | 93-95 | Björn G. Jonsson and Gunnar Berggren | Physical Work Capacity of Young Men in Sweden |
| 1979 | 7 | 3 | 97-104 | Svend Sabroe and Jørn Olsen | Health Complaints and Work Conditions among Lacquerers in the Danish Furniture Industry |
| 1979 | 7 | 3 | 105-113 | Göran Aurelius | Adjustment and Behaviour of Finnish and Southern European Immigrant Children in Stockholm: I. The Teachers' Assessment |
| 1979 | 7 | 3 | 115-117 | Timo Niemi | The Mortality of Male Old-age Pensioners Following Spouse's.Death |
| 1979 | 7 | 3 | 119-126 | Dagmund Svendsen and Finn Olav Kinge | Work and Disability at the Age of 30 Years: A Sociomedical Study of a Birth-cohort from Bergen VI. Education, Intellectual Ability and Occupation |
| 1979 | 7 | 3 | 127-130 | Anders Ahlbom | Seasonal Variations in the Incidence of Acute Myocardial Infarction in Stockholm |
| 1979 | 7 | 3 | 131-142 | Björn G. Jonsson and Irma Åstrand | Physical Work Capacity in Men and Women Aged 18 to.65 |
| 1979 | 7 | 3 | 143-149 | Johan A. Wallin | Child Traffic Accidents: An Investigation of Accident Factors |
| 1980 | 8 | 1 | 1-7 | Liisa Neittaanmäki, Kaj Koskelaa, Pekka Puska and Alfred L. McAlister | The Role of Lay Workers in Community Health Education: Experiences of the North Karelia Project |
| 1980 | 8 | 1 | 9-15 | Jaakko Tuomilehto, Pekka Puska, Jarmo Virtamo and Aulikki Nissinen | Hypertension Control in North Karelia before the Intervention of the North Karelia Project |
| 1980 | 8 | 1 | 17-23 | Aulikki Nissinen, Jaakko Tuomilehto and Pekka Puska | Management of Hypertension and Changes in Blood Pressure Level in Patients Included in the Hypertension Register of the North Karelia Project |
| 1980 | 8 | 1 | 25-31 | Olof Lannerstad | Morbidity Related to Smoking and Other Risk Factors |
| 1980 | 8 | 1 | 33-38 | Risto Honkanen and Jarl-Erik Michelsson | Construction of the Computerized Accident Registration System in a Casualty Department |
| 1980 | 8 | 1 | 39-41 | Timo Niemi | Retirement and Mortality |
| 1980 | 8 | 1 | 43-48 | Göran Aurelius | Adjustment and Behaviour of Finnish Immigrant Children in Stockholm: II. The Parents' Assessment |
| 1980 | 8 | 2 | 49-54 | Olav H. Førde and Dag S. Thelle | The Tromsø Heart Study: A Multiple Regression Analysis of the Relationship between Coronary Risk Factors and Some Physical and Social Variables |
| 1980 | 8 | 2 | 55-61 | Leif Öjesjö and Olle Hagnell | Prevalence of Male Alcoholism in a Cohort Observed for 25 Years |
| 1980 | 8 | 2 | 63-66 | Risto Honkanen, Olli Kiviluoto and Rolf Nordström | Victims of Assault Attending Casualty Departments |
| 1980 | 8 | 2 | 67-72 | Frej Stenbäck and Heikki Päivärinta | Relation between Clinical and Autopsy Diagnoses, Especially as Regards Cancer |
| 1980 | 8 | 2 | 73-80 | Odd Søreide and Jostein Lillestøl | Survival Patterns Following Primary Prosthetic Replacement for Acute Femoral Neck Fractures in the Elderly |
| 1980 | 8 | 3 | 81-87 | Paula Rantakallio and Antero Myhrman | The Child and Family Eight Years after Undesired Conception: The Child and Family after Undesired Conception |
| 1980 | 8 | 3 | 89-94 | Pamela Rachootin and Jørn Olsen | Secular Changes in the Twinning Rate in Denmark 1931 to 1977 |
| 1980 | 8 | 3 | 95-98 | Bernard Jeune | Mortality of Male Members of the Danish Semiskilled Workers' Union: Standardization by County |
| 1980 | 8 | 3 | 99-104 | Pekka Oja, Kalevi Pyörälä, Risto Kärävä and Sven Punsar | Mortality and Incidence of Chest Pain after an Exercise Intervention Programme |
| 1980 | 8 | 3 | 105-115 | Töres Theorell, Agneta Vigborg, Anders Kallner, Gösta Dahlén and Olov Wålinder | A comparison between Finnish immigrants and native Swedes in the greater Stockholm region—social and medical risk indicators of ischemie heart disease |
| 1980 | 8 | 3 | 117-123 | Björn G. Jonsson and Irma Åstrand | Resting Systolic Blood Pressure and Heart Rate at Work |
| 1980 | 8 | 3 | 125-129 | Mats Berglund | Health Insurance Records in the Course of Alcoholism: A Two-year Follow-up Study |
| 1980 | 8 | 3 | 131-136 | Dagmar Westerling and Björn G. Jonsson | Pain from the Neck-Shoulder Region and Sick Leave |
| 1980 | 8 | 3 | 137-139 | Einar Skarfors, Urban Waern and Christer Lidell | Findings at a re-examination of a Self-administered Questionnaire after Two and a half Years in a Sample of Sixty-year-old Men |
| 1980 | 8 | 3 | 141-148 | Gudjon Magnusson | Utilization of a Hospital Emergency Department in Stockholm: The Effects of Age, Sex and Marital Status |
| 1980 | 8 | 3 | 149-156 | Gudjon Magnusson | The Hospital Emergency Department as the Primary Source of Medical Care |
| 1981 | 9 | 1 | 1-10 | Elina Hemminki and Petter Bomann-Larsen | Drug Reimbursement in Four Nordic Countries |
| 1981 | 9 | 1 | 11-18 | Kurt Kolstad and Anders Wigren | Costs of and Needs for Resources in Hospital Care: A Calculation Model and Applications in Some Typical Cases in Orthopaedic Surgery |
| 1981 | 9 | 1 | 19-24 | Jørn Olsen | Some Methodologic Problems Encountered in Occupational Health Research |
| 1981 | 9 | 1 | 25-31 | Sture Roupe and Alvar Svanborg | Previous Job and Health at the Age of 70 |
| 1981 | 9 | 1 | 33-39 | Bengt Kristiansson | Ill-Health in Families of Infants with Low Rate of Weight Gain |
| 1981 | 9 | 1 | 41-47 | Johann A. Sigurdsson and Calle Bengtsson | Prevalence and Management of Arterial Hypertension in a Population Sample of Swedish Women |
| 1981 | 9 | 2 | 49-50 | Sune Nyström | Relation of Social Problems and Habitual Vulnerability to Medical Cases |
| 1981 | 9 | 2 | 51-57 | Markku Nurminen and Peter C. Holmberg | Temporal Characteristics Related to Leave from Work and Pregnancy among Mothers with Congenitally Malformed Offspring |
| 1981 | 9 | 2 | 59-61 | Lindsay Edouard and E. Edouard | The Dynamics of Perinatal Mortality: Maternal Age, Parity and Legitimacy |
| 1981 | 9 | 2 | 63-73 | Ragnhild Kornfält and Göran Ejlertsson | Total Consumption of Health Services by Schoolchildren in a Primary Health Care District in Southern Sweden: A Contemporary Registration of Consumption at the School Health Clinic, the Community Care Centre and the Hospital |
| 1981 | 9 | 2 | 75-79 | Bo Netterstrøm and Peter Laursen | Incidence and Prevalence of Ischaemic Heart Disease among Urban Busdrivers in Copenhagen |
| 1981 | 9 | 3 | 81-89 | Ragnhild Kornfält | Behavioural Assessments in the School Health Service: A Comparison of Information Obtained from Interviews with Parents, Teachers and Children |
| 1981 | 9 | 3 | 91-99 | Ragnhild Kornfält | Health Problems of 14-16-Year-Old Schoolchildren: A Follow-up of Previous Health and Behavioural Assessments |
| 1981 | 9 | 3 | 101-108 | Anders Foldspang | Statistical Prediction of Case Closure Status and Time Spent at a Rehabilitation Clinic: A Cost-Effectiveness Perspective |
| 1981 | 9 | 3 | 109-117 | Jukka T. Salonen, Jaakko Tuomilehto and Pekka Puska | The Relation of Physical Activity Changes to Changes in Serum Cholesterol and Body Weight in a Three-Year Follow-up of Population Sample |
| 1981 | 9 | 3 | 119-126 | Anders Bjelle, Erik Allander and Bengt Lundquist | Geographic Distribution of Rheumatic Disorders and Working Conditions in Sweden |
| 1981 | 9 | 3 | 127-133 | Lars Janzon, Sven-Erik Lindell and Erik Trell | Smoking and Disease: Attitudes and Knowledge in Middle-aged Men |
| 1982 | 10 | 1 | 1-2 | Per Sundby | Comment on Sune Nyström. Relation of prevention of social problems to virtues and morality |
| 1982 | 10 | 1 | 3-10 | Matilde Lajer | Unemployment and Hospitalization among Bricklayers |
| 1982 | 10 | 1 | 11-16 | Bo Mikaelsson, Nils Stjernberg and Lars-Gösta Wiman | The Prevalence of Bronchial Asthma and Chronic Bronchitis in an Industrialized Community in Northern Sweden |
| 1982 | 10 | 1 | 17-22 | Birgitta Kolmodin-Hedman, Lars Hedström and Bertil Grönqvist | Menopausal Age and Spontaneous Abortion in a Group of Women Working in a Swedish Steel Works |
| 1982 | 10 | 1 | 23-26 | Jørn Olsen | Confounding as a Result of Differential Diagnosis |
| 1982 | 10 | 1 | 27-32 | Gösta Tibblin, Lennart Welin, Bo Larsson, Inga-Lisa Ljungberg and Kurt Svärdsudd | The Influence of Repeated Health Examinations on Mortality in a Prospective Cohort Study, with a Comment on the Autopsy Frequence: The Study of Men Born in 1913 |
| 1982 | 10 | 2 | 33-41 | Dan Mellström, Åke Nilsson, Anders Odén, Åke Rundgren and Alvar Svanborg | Mortality among the Widowed in Sweden |
| 1982 | 10 | 2 | 43-47 | Marianne Damlund, Steffen Gøth, Peter Hasle, Bernard Jeune and Karen Munk | The Incidence of Disability Pensions and Mortality among Semi-skilled Construction Workers in Copenhagen: A Retrospective Cohort Study with Two Control Groups |
| 1982 | 10 | 2 | 49-56 | Reijo Luoma and Antti Koivikko | Occurrence of Atopic Diseases in Three Generations |
| 1982 | 10 | 2 | 57-61 | Jan Olof Hörnquist | The Concept of Quality of Life |
| 1982 | 10 | 2 | 63-69 | Bengt Jönsson and Raymunda Silverberg | Variations between and within Countries in Hospital Care for Peptic Ulcer: A Comparison between Denmark and Sweden |
| 1982 | 10 | 3 | 73-76 | Bernard Jeune | Survival Experience of Semi-skilled Disability Pensioners in Denmark |
| 1982 | 10 | 3 | 77-80 | Elsebeth Lynge and Bernard Jeune | Mortality Studies in Unskilled and Semi-skilled Workers in Denmark: A Comparative Analysis |
| 1982 | 10 | 3 | 81-85 | Niels Sten Hansen and Bernard Jeune | Incidence of Disability Pensions among Slaughterhouse Workers in Denmark: With Special Regard to Diagnosis of the Musculo-skeletal System |
| 1982 | 10 | 3 | 87-93 | Jørgen Aagaard | Social Factors and Life Events as Predictors for Children's Health: A One-year Prospective Study after Discharge from Hospital |
| 1982 | 10 | 3 | 95-99 | Tuomo Visuri and Risto Honkanen | The Role of Socio-economic Status and Place of Residence in Total Hip Replacement |
| 1982 | 10 | 3 | 101-104 | Åke Andrén-Sandberg and Anders Lindstran | Injuries Sustained in Junior League Handboll: A Prospective Study of Validity in the Registration of Sports Injuries |
| 1982 | 10 | 3 | 105-112 | P. Pietinen, A. Tanskanen and J. Tuomilehto | Assessment of Sodium Intake by a Short Dietary Questionnaire |
| 1982 | 10 | 3 | 113-118 | Jan Olof Hörnquist | Abuse of Alcohol and Disability Pension: The Effect of the Liberalization in Sweden in 1977 |
| 1983 | 11 | 1 | 3-6 | Jørn Olsen, Lene Zeuthen Heidam and Lene Zeuthen Hediam | Analysis of Pathological Outcome of Pregnancy |
| 1983 | 11 | 1 | 7-10 | Liisa Keltikangas-Järvinen, L. Keltikangas-Järwinen and Seppo Autio | Psychological Obstacles to Genetic Education |
| 1983 | 11 | 1 | 11-16 | Iwan Mark | The Newborn Addict, Ante- and Postnatal Care |
| 1983 | 11 | 1 | 17-22 | Paula Rantakallio | Family Background to and Personal Characteristics Underlying Teenage Smoking: Background to Teenage Smoking |
| 1983 | 11 | 1 | 23-26 | Knut Dalaker, Berthold Grünfeld and Arild Jansen | Some Social Background Variables among Smoking and Non-smoking Primiparae |
| 1983 | 11 | 1 | 27-32 | Peter Allebeck, Kjell Ljungström and Erik Allander | Rheumatoid arthritis in a medical information system: How valid is the diagnosis? |
| 1983 | 11 | 2 | 33-35 | Svend Heinild | On the changes in the Concept of Illness throughout History |
| 1983 | 11 | 2 | 36 | Lars Garby and Jørgen Cohn | Letter to the Editor |
| 1983 | 11 | 2 | 37-40 | Elsebeth Lynge and Bernard Jeune | Excess Mortality among Male Unskilled and Semi-skilled Workers: A Negative Slope with Age |
| 1983 | 11 | 2 | 41-52 | Kari Vinni | Occupational Morbidity Based on Social Insurance Records in Finland |
| 1983 | 11 | 2 | 53-58 | Martin Elton and Jan Olof Hörnquist | Grounds for Disability Pension: The Younger Abuser as Compared with Older Abusers of Alcohol |
| 1983 | 11 | 2 | 59-64 | Poul Erik Petersen | Dental Visits, Dental Health Status and Need for Dental Treatment in a Danish Industrial Population |
| 1983 | 11 | 3 | 65-68 | Pekka Puska, Aulikki Nissinen, Jukka T. Salonen and Jaakko Toumilehto | Ten Years of the North Karelia Project: Results with Community-based Prevention of Coronary Heart disease |
| 1983 | 11 | 3 | 69-73 | Anders Green and Niels Kromann | Fertility and Mortality 1950-1974 in the Upernavik District, Greenland |
| 1983 | 11 | 3 | 75-80 | Iwan Mark | Medroxyprogesterone Acetate as a Contraceptive for Female Drug Addicts |
| 1983 | 11 | 3 | 81-85 | Lene Zeuthen Heidam | Spontaneous Abortions among Factory Workers: The Importance of Gravidity Control |
| 1983 | 11 | 3 | 87-89 | Christina Jorup-Rönström and Sven Britton | Efficacy of Reporting Systems of Adverse Reactions to Drugs and Care |
| 1983 | 11 | 3 | 91-96 | Jan Olof Hörnquist and Martin Elton | A Prospective Longitudinal Study of Abusers of Alcohol Granted Disability Pension |
| 1983 | 11 | 3 | 97-106 | Sune Nyström | Personality Variations in a Population: Intelligence |
| 1983 | 11 | 3 | 107-111 | Peter Bjerregaard | Housing Standards, Social Group, and Respiratory Infections in Children of Upernavik, Greenland |
| 1983 | 11 | 3 | 113-117 | Svend Lings, Jørn Jensen, Søren Christensen and Jens T. Møller | Some Public Expenses in Connection with Occupational Accidents: A Follow-up Study of an Emergency Department Material |
| 1983 | 11 | 3 | 119 | Jørn Olsen and Pamela Rachootin | The end of the decline in twinning rates? |
| 1984 | 12 | 1 | 1-2 | Jørn Olsen | Information Bias in Case-control Studies in Occupational Health Epidemiology |
| 1984 | 12 | 1 | 3-5 | Simo Räisänen and Pekka Puska | Fish Tapeworm, a Disappearing Health Problem in Finland |
| 1984 | 12 | 1 | 7-14 | Tom-Harald Edna and Johan Cappelen | Hospital Admitted Head Injury: A Prospective Study in Trøndelag, Norway, 1979-80 |
| 1984 | 12 | 1 | 15-23 | Gunnar Mowé and Bjørn Gylseth | Medico-legal Aspects of Malignant Mesothelioma |
| 1984 | 12 | 1 | 25-29 | Svend Lings, Jørn Jensen, Søren Christensen and Jens T. Møller | The Consequences to the Injured of Occupational Accidents: A Follow-up Study of an Emergency Department Material |
| 1984 | 12 | 1 | 31-37 | Sirkka-Liisa Kivelä | Predictors of Mortality in a Disabled and Elderly Population Receiving Domiciliarly Based Services |
| 1984 | 12 | 1 | 39-47 | Bjarne Roed-Petersen | Smoking habits in East Denmark at October 1979 |
| 1984 | 12 | 1 | 49-53 | Jyri Raudsepp and Mati Rahu | Smoking among Schoolteachers in Estonia 1980 |
| 1984 | 12 | 1 | 55-63 | Jørn Olsen and Svend Sabroe | Screening for Neuropsychiatrie Dysfunction: A Latent Structure Analysis of Two Questionnaires |
| 1984 | 12 | 2 | 65-68 | J. A. Muir Gray | The Elderly in Scandinavia Demographic, Economic, Social and Health Conditions in 1834 |
| 1984 | 12 | 2 | 69-74 | I. Kuorinka and M. Nurminen | Arduousness of Work, Career, and Disability Pensioning of Finnish Iron Ore Miners |
| 1984 | 12 | 2 | 75-82 | Eeva Widström, Bo Nilsson and Thore Martinsson | Use of Dental Services by Finnish Immigrants in Sweden Assessed by Questionnaire |
| 1984 | 12 | 2 | 83-90 | J. Aagaard, E. Amdrup, C. Aminoff and F. Hanberg Sørensen | A Predictor Analysis of Patients' Assessment of Outcome after Operation for Duodenal Ulcer: A One-year Prospective Study |
| 1984 | 12 | 2 | 91-103 | Peter de Château and Britt Wiberg | Long-term Effect on Mother–Infant Behaviour of Extra Contact during the First Hour post Partum: III. Follow-up at One Year |
| 1984 | 12 | 3 | 105-107 | Eero Lahelma | Does Unemployment Challenge Public Health? |
| 1984 | 12 | 3 | 109-110 | Urban Janlert | Unemployment—a Challenge to Public Health |
| 1984 | 12 | 3 | 111-114 | Johannes Ipsen and Jørn Olsen | Estimating Sensitivity and Specificity in Order to Correct for Misclassification |
| 1984 | 12 | 3 | 115-120 | E. M. Damsgaard, A. Frøland, A. Green and M. Hauge | An Alternative Sampling Approach to the Study of Diabetes Prevalence |
| 1984 | 12 | 3 | 121-127 | Johannes Ipsen | The Ubiquitous 2×2 Table, its Parameters and Their Confidence Limits |
| 1984 | 12 | 3 | 129-136 | Eeva Widström and Bo Nilsson | Dental Health and Perceived Treatment Needs of Finnish Immigrants in Sweden |
| 1984 | 12 | 4 | 137-138 | Gro Harlem Brundtland | Privatization of the Health Services |
| 1984 | 12 | 4 | 139 | Gunnar Biörck | Fair distribution of health services |
| 1984 | 12 | 4 | 141-146 | Margus Mägi, Erik Allander, Anders Bjelle and Arni Ragnarsson | Rheumatic Disorders in a Health Survey: How Valid and Reliable Are the Reports? |
| 1984 | 12 | 4 | 147-154 | Peter Allebeck and Gudrun Lindberg | Rheumatic Diseases in a Health Interview Survey and in In-patient Care: A Record Linkage Study on Two Registers |
| 1984 | 12 | 4 | 155-164 | Bo J. A. Haglund | Geographical and Socioeconomic Distribution of Physical Activity at Work and Leisure Time and Its Relation to Morbidity in a Swedish Rural County |
| 1984 | 12 | 4 | 165-169 | Margareta Leijon and Bo Mikaelsson | Repeated Short-term Sick-leave as a Possible Symptom of Psycho-social Problems |
| 1984 | 12 | 4 | 171-176 | Sture Malmgren and Gunnar Andersson | Corporate Reported Sick Leave and its Relationship with Education, Responsibility and Blood Pressure |
| 1984 | 12 | 4 | 177-181 | Jaakko Tuomilehto, Hannes Enlund, Jukka T. Salonen and Aulikki Nissinen | Alcohol, Patient Compliance and Blood Pressure Control in Hypertensive Patients |
| 1984 | 12 | 4 | 183-190 | Per E. Børdahl | The Social and Gynaecological Background of 218 Sterilized Women |
| 1984 | 12 | 4 | 191-194 | Per E. Børdahl | The Attitudes of Sterilized Women to Contraceptive Sterilization |
| 1984 | 12 | 4 | 195-199 | Viljo Nyyssönen, Ilkka Paunio and Jevgenij Borovsky | Oral Health in Finland and the Soviet Union: A Joint Study |
| 1985 | 13 | 1 | 1-13 | Steinar Tretli, Kjell Bjartveit, Olav Per Foss, Trond Haider and Per G. Lund-Larsen | Intervention on Cardiovascular Disease Risk Factors in Finnmark County: Changes after a Period of Three Years: The Cardiovascular Disease Study in Finnmark County, Norway |
| 1985 | 13 | 1 | 15-22 | Gudrun Silverbåge Carlsson and Kurt Svärdsudd | Comparison of Participants and Non-Participants in a Population Study of Injuries: The Study of Men Born in 1913 and 1923 |
| 1985 | 13 | 1 | 23-27 | Tom-Harald Edna and Johan Cappelen | Head Injury in Road Traffic Accidents: A Prospective Study in Trøndelag, Norway, 1979-80 |
| 1985 | 13 | 1 | 29-34 | P. Magnus, K. Berg, T. Bjerkedal and W. E. Nance | The Heritability of Smoking Behaviour in Pregnancy, and the Birth Weights of Offspring of Smoking-discordant Twins |
| 1985 | 13 | 1 | 35-40 | Flemming Scheutz | Dental Habits, Knowledge, and Attitudes of Young Drug Addicts |
| 1985 | 13 | 1 | 41-47 | P. E. Børdahl | Long-term Regret Among 216 Sterilized Women: A Six-year Follow-up Investigation |
| 1985 | 13 | 2 | 49-52 | Wiesław Jedrychowski, Beata Tobiasz-Adamczyk, Antoni Olma and Piotr Gradzikiewicz | Survival Rates among Seventh Day Adventists Compared with the General Population in Poland |
| 1985 | 13 | 2 | 53-66 | Bo J. A. Haglund | Geographical and Socioeconomic Distribution of High Blood Pressure and Borderline High Blood Pressure in a Swedish Rural County |
| 1985 | 13 | 2 | 67-74 | Carl-Göran Ohlson and Berit Ydreborg | Participants and Non-participants of Different Categories in a Health Survey: A Cross-sectional Register Study |
| 1985 | 13 | 2 | 75-82 | Sirkka-Liisa Kivelä | Disability among Home-nursed Patients and Home-help Clients |
| 1985 | 13 | 2 | 83 | Åke Pålsson | A Five-year Follow-up of 305 Men with Chronic Alcoholism |
| 1985 | 13 | 3 | 85-86 | Eeva Kuuskoski Vikatmaa | Future Challenges to Health Services |
| 1985 | 13 | 3 | 87-88 | Hans Draminsky Petersen and Peter Jacobsen | Life-threatening Torture without Visible Marks |
| 1985 | 13 | 3 | 89-93 | Hans Draminsky Petersen, Ulrik Abildgaard, Gedske Daugaard, Per Jess, Henrik Marcussen and Marianne Wallach | Psychological and Physical Long-term Effects of Torture: A Follow-up Examination of 22 Greek Persons Exposed to Torture 1967-1974 |
| 1985 | 13 | 3 | 95-97 | Olof Johnell, Hans Kristenson and Inga Redlund-Johnell | Lower Limb Fractures and Registration for Alcoholism |
| 1985 | 13 | 3 | 99-102 | Jukka T. Salonen, Heikki Hämynen, Ulla Leino, Ella Kostiainen and Timo Sahi | Relation of Alcohol, Physical Activity, Dietary Fat and Smoking to Serum HDL and Total Cholesterol in Young Finnish Men |
| 1985 | 13 | 3 | 103-108 | Jukka T. Salonen, Heikki Hämynen and Olli P. Heinonen | Impact of a Health Education Program and Other Factors on Stopping Smoking after Heart Attack |
| 1985 | 13 | 3 | 109-112 | Heikki Tuutti, Eino Honkala and Juhani Laurinkari | Acceptability of Fluoride Use in Finnish Municipalities |
| 1985 | 13 | 3 | 113-118 | Elina Hemminki | Perinatal Mortality Distributed by Type of Hospital in the Central Hospital District of Helsinki, Finland |
| 1985 | 13 | 3 | 119-126 | Sari Anttila, Jorma Takala, Anneli Takala, Olli-Pekka Ollila, Eero Vierimaa and Marjatta Koski | Health Status and Social Conditions of the Elderly in Remote Districts: Results of a Screening Programme |
| 1985 | 13 | 3 | 127-132 | Klavs Lindeneg | Economic Appraisal in Occupational Health and Safety Regulation: A Study of Some Standard Methods |
| 1985 | 13 | 4 | 133-134 | Nils Rosdahl | Health for All by the Year 2000: Nordic Responsibilities and Potential Approaches |
| 1985 | 13 | 4 | 135-136 | Sven-Olof Isacsson | Health for All in Europe |
| 1985 | 13 | 4 | 137-146 | Paul J. Freund and Katele Kalumba | Monitoring and Evaluation of Primary Health Care in Rural Zambia: A Comparative Study |
| 1985 | 13 | 4 | 147-152 | Olav Axelson | Dealing with the Exposure Variable in Occupational and Environmental Epidemiology |
| 1985 | 13 | 4 | 153-157 | Pål Hartvig and Grethe Sterner | Childhood Psychologic Environmental Exposure in Women with Diagnosed Somatoform Disorders: A Case-control Study |
| 1985 | 13 | 4 | 159-163 | Lene Zeuthen Heidam and Jørn Olsen | Self-reported Data on Spontaneous Abortions Compared with Data Obtained by Computer Linkage with the Hospital Registry |
| 1985 | 13 | 4 | 165-167 | Denny Vågerö and Robert Olin | How Do We Analyse a Cohort of Healthy Workers? |
| 1985 | 13 | 4 | 169-172 | Reidar Almås and Jørn Ødegård | Morbidity among Self-employed Farmers in Norway |
| 1985 | 13 | 4 | 173-180 | Jaakko Tuomilehto, Laura Jalkanen, Jukka T. Salonen and Aulikki Nissinen | Factors Associated with Changes in Body Weight during a Five-year Follow-up of a Population with High Blood Pressure |
| 1985 | 13 | 4 | 181-184 | Sven Bremberg and Bo Mikaelsson | Predictability of School Absence for Rate of Sick Leave |
| 1986 | 14 | 1 | 1-2 | Poul Erik Petersen | Community Dentistry and Sociology |
| 1986 | 14 | 1 | 3-6 | Sigurður Björnsson, Ásmundur Brekkan and Hrafn Tulinius | Mass Screening for Breast Cancer |
| 1986 | 14 | 1 | 7-14 | K. Schmidt, J. Mosbech and E. Worsøe | Cimetidine Utilization in Denmark 1977–1981 |
| 1986 | 14 | 1 | 15-23 | Jan Erik Otterstad, Ingunn Tjore and Per Sundby | Social Function of Adults with Isolated Ventricular Septal Defects: Possible Negative Effects of Surgical Repair? |
| 1986 | 14 | 1 | 25-30 | Gunnar Andersson and Sture Malmgren | Risk Factors and Reported Sick Leave among Employees of Saab-Scania, Linköping, Sweden, between the Ages of 50 and 59 |
| 1986 | 14 | 1 | 31-38 | Sirkka-Liisa Kivelä and Risto Honkanen | Mortality from Violent Causes among the Elderly Population in Finland in 1951–1979 |
| 1986 | 14 | 1 | 39-47 | Seppo Aro, Leena Räsänen and Risto Telama | Social Class and Changes in Health-related Habits in Finland in 1973–1983 |
| 1986 | 14 | 2 | 49-50 | Anders Christian Gogstad and A. E. Gogstad | The Use of Social Background Data in Social Medicine Research |
| 1986 | 14 | 2 | 51-56 | Ronan M. Conroy, Suzanne Cahill, Risteard Mulcahy, Howard Johnson, N. Johnson, Ian M. Graham and Noel Hickey | The Relation of Social Class to Risk Factors, Rehabilitation, Compliance and Mortality in Survivors of Acute Coronary Heart Disease |
| 1986 | 14 | 2 | 57-59 | Antero Myhrman | Longitudinal Studies on Unwanted Child: A Review on Studies in Prague and Northern Finland |
| 1986 | 14 | 2 | 61-66 | Finn Egil Skjeldestad | Induced Abortions and Births: Trends in Seven Counties, Norway, 1972-1983 |
| 1986 | 14 | 2 | 67-74 | Steffen Bülow, Niels Vilstrup Holm and Mogens Hauge | The Incidence and Prevalence of Familial Polyposis Coli in Denmark |
| 1986 | 14 | 2 | 75-82 | Lothar Schelp and Leif Svanström | One-year Incidence of Home Accidents in a Rural Swedish Municipality |
| 1986 | 14 | 2 | 83-86 | Ulf Björnstig and Paul Tordai | Tobogganing and Sledging Accidents: Mechanisms and Injuries |
| 1986 | 14 | 2 | 87-91 | Mariann Olsson, Olof Edhag and Urban Rosenqvist | Emergency Care: Identification of Psychosocial Problems |
| 1986 | 14 | 2 | 93-96 | O. Edhag, M. Olsson, M. Rosenqvist and U. Rosenqvist | Emergency Room Triage by a Medical Specialist |
| 1986 | 14 | 2 | 97-103 | Gunnel Widlund and Bengt Höjer | Work Pattern of Child Health Centre Nurses in Stockholm |
| 1986 | 14 | 2 | 105-111 | Kerstin Hulter Åsberg | Assessment of ADL in Home-care for the Elderly: Change in ADL and Use of Short-term Hospital Care |
| 1986 | 14 | 3 | 113-118 | Peter Ebbesen, Mads Melbye and Jørn Beckmann | Fear of AIDS: A Communication from Biologists to Psychologists/Sociologists |
| 1986 | 14 | 3 | 119-122 | Bengt-Einar Dahlgren and Per-Gunnar Svensson | Public Opinion on the Ambulance Service in a Rural and an Urban Region |
| 1986 | 14 | 3 | 123-126 | Eino Honkala | The Critics of Modern Medicine and Its Implications for Dentistry |
| 1986 | 14 | 3 | 127-132 | Kerstin Hulter Åsberg | Physicians' Outcome Predictions for Elderly Patients: Survival, Hospital Discharge, and Length of Stay in a Department of Internal Medicine |
| 1986 | 14 | 3 | 133-140 | Sture Malmgren and Gunnar Andersson | Who Were Reached by and Participated in a One Year Newspaper Health Information Campaign? |
| 1986 | 14 | 3 | 141-146 | Gunnar Andersson and Sture Malmgren | Changes in Self-reported Experienced Health and Psychosomatic Symptoms in Voluntary Participants in a 1-Year Extensive Newspaper Exercise Campaign |
| 1986 | 14 | 3 | 147-150 | Jørn Olsen and Lisbeth B. Knudsen | Twinning Rates by Residence in Denmark 1978 to 1982 |
| 1986 | 14 | 3 | 151-160 | Denny Vågerö and Gunnar Persson | Occurrence of Cancer in Socioeconomic Groups in Sweden: An Analysis Based on the Swedish Cancer Environment Registry |
| 1986 | 14 | 3 | 161-168 | Kurt Rasmussen and Svend Sabroe | Neuropsychological Symptoms among Metal Workers Exposed to Halogenated Hydrocarbons |
| 1986 | 14 | 4 | 169-170 | Gösta Carlsson | There Must Be an Explanation |
| 1986 | 14 | 4 | 171-178 | Jógvan Petersen, Peter Skinhøj and Thorkil Thorsen | An Epidemic of Cirrhosis in Danish Women Revisited |
| 1986 | 14 | 4 | 179-181 | Anders Knutsson | Computerization in Industry Causes Problems for People with Reading and Writing Difficulties (Dyslexia) |
| 1986 | 14 | 4 | 183-195 | John Gunnar Mæland and Odd Erik Havik | Return to Work after a Myocardial Infarction: the Influence of Background Factors, Work Characteristics and Illness Severity |
| 1986 | 14 | 4 | 197-204 | Lothar Schelp and Leif Svanström | One-year Incidence of Occupational Accidents in a Rural Swedish Municipality |
| 1986 | 14 | 4 | 205-209 | Finn Egil Skjeldestad and Leiv S. Bakketeig | Induced Abortion: Trends in the Tendency to Repeat, Norway, 1972-1981 |
| 1986 | 14 | 4 | 211-212 | Sven Britton | Psychosocial Aspects of HTLV-III Infections |
| 1987 | 15 | 1 | 1-2 | Kirsten Schmidt and Henrik Zoffmann | AIDS and Social Medicine: Strategies for Research |
| 1987 | 15 | 1 | 3-10 | Timo Hakulinen, Knut Magnus and Leena Tenkanen | Is Smoking Sufficient to Explain the Large Difference in Lung Cancer Incidence between Finland and Norway? |
| 1987 | 15 | 1 | 11-17 | Anders Ericson, Margareta Eriksson, Bengt Källén and Olav Meirik | Birth Weight Distribution as an Indicator of Environmental Effects on Fetal Development |
| 1987 | 15 | 1 | 19-29 | J. A. Haglund | Geographical and Socioeconomic Distribution of Overweight and Morbidity in a Rural Swedish County |
| 1987 | 15 | 1 | 31-35 | B. Jacobsson and L. Schelp | Home Accidents among Children and Teenagers in a Swedish Rural Municipality |
| 1987 | 15 | 1 | 37-40 | O. Pärssinen, K. M. Saari and J. Kirjonen | Need for Near Vision in Daily Work in Different Occupational Groups |
| 1987 | 15 | 1 | 41-47 | Bjarne K. Jacobsen, Synnøve F. Knutsen and Raymond Knutsen | The Tromsø Heart Study: Comparison of Information from a Short Food Frequency Questionnaire with a Dietary History Survey |
| 1987 | 15 | 1 | 49-53 | J. Heidmann and H. Birn | The Self-fulfilling Prophecy in Dental Prophylaxis |
| 1987 | 15 | 1 | 55-56 | Niels Lynöe | Informed Consent |
| 1987 | 15 | 2 | 57-61 | Hannu Vuori | WHO—A Source of Inspiration for Researchers? |
| 1987 | 15 | 2 | 63-66 | Paula Rantakallio | Social Class Differences in Mental Retardation and Subnormality |
| 1987 | 15 | 2 | 67-72 | Leena Tenkanen and Lyly Teppo | Migration, Marital Status and Smoking as Risk Determinants of Cancer |
| 1987 | 15 | 2 | 73-77 | Hallvard Gjerde | Daily Drinking and Drunken Driving |
| 1987 | 15 | 2 | 79-85 | Göran Aurelius, Arne Rådestad, Ingvar Nylander and Rolf Zetterström | Psychosocial Factors and Pregnancy Outcome: Part Two of a Prospective Longitudinal Study of Children in a New Stockholm Suburb |
| 1987 | 15 | 2 | 87-97 | Lena Westbom and Ragnhild Kornfält | Chronic Illness among Children in a Total Population: An Epidemiological Study in a Swedish Primary Health Care District |
| 1987 | 15 | 2 | 99-103 | O. Pärssinen, J. Kirjonen and K. M. Saari | Wearing of Spectacles and Occurrence of Ocular Symptoms in Close Work in Different Occupations |
| 1987 | 15 | 2 | 105-109 | Goran Westman, Marianne Hanning and Bengt Mattsson | Utilization of Inpatient and Emergency Care: Effects of Changes in Primary Care System |
| 1987 | 15 | 2 | 111-116 | Leiv S. Bakketeig | How to Evaluate Perinatal Care |
| 1987 | 15 | 3 | 117-122 | Reijo K. Salmela | Is Primary Health Care Progressing in Europe? |
| 1987 | 15 | 3 | 123-130 | Ulla Lindskog, Per Lindskog and Stig Wall | Water Supply, Sanitation and Health Education Programmes in Developing Countries: Problems of Evaluation |
| 1987 | 15 | 3 | 131-137 | Gunnar Tellnes, Per Fugelli and Arild Bjørndal | Treatment of Mental Problems in General Practice: A One Year Prospective Study from an Island Community in Northern Norway |
| 1987 | 15 | 3 | 139-144 | Melcher G. K. Falkenberg | Diabetes Mellitus: Prevalence and Local Risk Factors in a Primary Health Care District |
| 1987 | 15 | 3 | 145-151 | Olavi Pärssinen | The Wearing of Spectacles in Different Social and Educational Groups in a Sample of the Population of Central Finland |
| 1987 | 15 | 3 | 153-157 | Mats Brommels, Reijo Tilvis and Lauri Autio | Cerebrovascular Disease: Declining Incidence but Increasing Hospital Utilisation |
| 1987 | 15 | 3 | 159-167 | Ulla Waldenström and Gunilla Lindmark | Early and Late Discharge after Hospital Birth: A Comparative Study of Parental Background Characteristics |
| 1987 | 15 | 3 | 169-174 | Carsten Hendriksen, Ellinor Lund and Edith Strømgård | Use of Social and Health Services by Elderly People during the Terminal 18 Months of Life |
| 1987 | 15 | 3 | 175-183 | Ingemar Åkerlind, Jan Olof Hörnquist and Birgit Hansson | Loneliness Correlates in Advanced Alcohol Abusers: I. Social Factors and Needs |
| 1987 | 15 | 3 | 185-189 | Nils-Ove Månsson and Bo Israelsson | Middle-aged Men before and after Disability Pension: Health Screening Profile with Special Emphasis on Alcohol Consumption |
| 1987 | 15 | 3 | 191-198 | Laura Jalkanen, Jaakko Tuomilehto, Antti Tanskanen and Pekka Puska | Accuracy of Self-reported Body Weight Compared to Measured Body Weight: A Population Survey |
| 1987 | 15 | 3 | 199-203 | Ole M. Böstman | Suicidal Attempts by Jumping from Heights: A Three-year Prospective Study of Patients Admitted to an Urban University Accident Department |
| 1987 | 15 | 3 | 204 | Jørn Olsen | Reviewed Work: Modern Epidemiology by K. J. Rothman |
| 1987 | 15 | 4 | 205-209 | Hans T. Waaler, Gorm Jensen, Peter Schnohr, Pirjo Pietinen, Aulikki Nissinen, Bo Larsson and Calle Bengtson | Body Mass Index in the Scandinavian Countries |
| 1987 | 15 | 4 | 211-217 | Björn Söderfeldt, Berth Danermark and Sven Larsson | Social Class and Sickness Absences: A Comparative Study of Four Ways to Measure Social Class |
| 1987 | 15 | 4 | 219-224 | Heikki Hämynen, Erkki Vartiainen, Timo Sahi, Unto Pallonen and Jukka T. Salonen | Social, Personality and Environmental Determinants of Smoking in Young Finnish Men |
| 1987 | 15 | 4 | 225-232 | Jan Olof Hörnquist and Ingemar Åkerlind | Loneliness Correlates in Advanced Alcohol Abusers: II. Clinical and Psychological Factors |
| 1987 | 15 | 4 | 233-240 | Måns Rosén, Stig Wall, Marianne Hanning, Gudrun Lindberg and Lennarth Nyström | Smoking Habits and their Confounding Effects among Occupational Groups in Sweden |
| 1987 | 15 | 4 | 241 | Henrik Toft Sørensen, Jørgen Østergaard Nielsen and Jørn Nielsen | Head Injuries Caused by Occupational Accidents |
| 1987 | 15 | 4 | 243-246 | Bengt B. Arnetz, Lars-Gunnar Hörte, Anders Hedberg and Hans Malker | Suicide among Swedish Dentists: A Ten-Year Follow-Up Study |
| 1987 | 15 | 4 | 247-252 | Bjarne R. Jansson | The Yield of Systems for Continuous and Periodic Injury Surveillance in Emergency Care with Emphasis on Farm-work-related Accidents |
| 1987 | 15 | 4 | 253-260 | Anders Foldspang | Standardized Performance Tests and Their Impact on the Decisions Determining the Type of Rehabilitation Program: A Health Service Case Study from a Vocational Rehabilitation Clinic |
| 1987 | 15 | 4 | 261-265 | Kerstin Hulter Åsberg | Disability as a Predictor of Outcome for the Elderly in a Department of Internal Medicine: A Comparison of Predictions Based on Index of ADL and Physician Predictions |
| 1988 | 16 | 1 | 1-3 | Tapani Melkas | Health for All by the Year 2000—National Finnish Impact on the Global Development Programme |
| 1988 | 16 | 1 | 5-13 | Gudrun E. Hedberg | The Period Prevalence of Musculoskeletal Complaints among Swedish Professional Drivers |
| 1988 | 16 | 1 | 15-19 | CHRISTIAN BRAHE PEDERSEN and BENGT ZACHAU-CHRISTIANSEN | Chronic Otitis Media and Sequelae in the Population of Greenland |
| 1988 | 16 | 1 | 21-25 | Bo Jacobsson and Lothar Schelp | One-Year Incidence of Occupational Injuries among Teenagers in a Swedish Rural Municipality |
| 1988 | 16 | 1 | 27-33 | Jan Olof Hörnquist, Birgit Hansson and Ingemar Åkerlind | The Working Capacity of the Alcohol Abuser: Prognostic Multiple Regression Analyses |
| 1988 | 16 | 1 | 35-40 | Colin Sanderson and Leif Svanström | Contributions of Social Medicine and Systems Analysis to Formulating Objectives for a Community-based Cancer Prevention Programme |
| 1988 | 16 | 1 | 41-48 | Ingrid Thranov, Anne Grete Kjersgaard, Ole Vedel Rasmussen and Jens Hertz | Regret among 547 Danish Sterilized Women |
| 1988 | 16 | 1 | 49-52 | Jørn Olsen and Grethe Frische | Comparison between Data Obtained through Questionnaires and Interviews: Life-style Habits of Pregnant Women |
| 1988 | 16 | 1 | 53-61 | M. Bågedahl-Strindlund and G. Undén | Mentally Ill Mothers and Their Children: A Controlled Study of Social Welfare Utilization |
| 1988 | 16 | 1 | 62 | Editorial | The Quality of the Journal |
| 1988 | 16 | 1 | 63-64 | Anne Loft and Myra Lewinter | Explaining Regional Variations in Health Care |
| 1988 | 16 | 2 | 65-66 | Sven Britton | AIDS Epidemics—Part Two |
| 1988 | 16 | 2 | 67-71 | Bengt Brorsson and Claes Herlitz | The AIDS Epidemic in Sweden: Changes in Awareness, Attitudes and Behavior |
| 1988 | 16 | 2 | 73-74 | Bengt Brorsson and Claes Herlitz | AIDS Epidemic Ranked as "Public Enemy Number One' |
| 1988 | 16 | 2 | 75-79 | Dimitrios Trichopoulos | Passive Smoking and Lung Cancer: The Ipsen Lecture 1987 |
| 1988 | 16 | 2 | 81-85 | John M. Carstensen, Lottie Wicksell, Gunnar Eklund and Jan-Åke Gustafsson | Lung Cancer Incidence among Swedish Bakers and Pastrycooks: Temporal Variation |
| 1988 | 16 | 2 | 87-93 | Kjerstin Genell Andrén | A Study of the Relationship between Social Network, Perceived Ill Health and Utilization of Emergency Care: A Case-Control Study |
| 1988 | 16 | 2 | 95-100 | Jan E. Levin, Harald Persson, Per Bjurulf and Sven Bremberg | Health Careers—a Method of Health Education at School for Adolescents |
| 1988 | 16 | 2 | 101-104 | Bjarne Koster Jacobsen and Dag Steinar Thelle | The Tromsø Heart Study: Responders and Non-responders to a Health Questionnaire, Do They Differ? |
| 1988 | 16 | 2 | 105-109 | Björn U. C. Gerdle and Gudrun E. Hedberg | Alcohol Consumption and Complaints from the Musculoskeletal System among Engine Drivers—an Epidemiological Study |
| 1988 | 16 | 2 | 111-113 | Hallvard Gjerde and Jørg Mørland | A Two Year Prospective Study of Rearrests for Drunken Driving |
| 1988 | 16 | 2 | 115-119 | Finn Egil Skjeldestad | Induced Abortions and Births: Changes in Maternal Age and Parity in Six Counties, Norway, 1972-1981 |
| 1988 | 16 | 2 | 121-127 | M. Bågedahl-Strindlund | Children of Mentally Ill Mothers: Mental Development, Somatic Growth and Social Outcome |
| 1988 | 16 | 2 | 128 | Niels Lynöe | Reviewed Work: Philosophy of Medicine. An Introduction by H. R. Wulff, S. Andur Pedersen, R. Rosenberg |
| 1988 | 16 | 3 | 129-135 | Karin Fahlgren | Two Doses of MMR Vaccine—Sufficient to Eradicate Measles, Mumps and Rubella? |
| 1988 | 16 | 3 | 137 | Henrik Toft Sørensen, Bo Christensen and Erling Kjærulff | A Follow-up Investigation with High Response Rate |
| 1988 | 16 | 3 | 139-143 | Piroska Östlin and Mats Thorslund | Problems with Cross-sectional Data in Research on Working Environment and Health |
| 1988 | 16 | 3 | 145-148 | Bengt Brorsson | Evaluation of Welfare Programs—A Mission Impossible? |
| 1988 | 16 | 3 | 149-153 | Richard Stensman and Ulla-Britt Sundqvist-Stensman | Physical Disease and Disability among 416 Suicide Cases in Sweden |
| 1988 | 16 | 3 | 155-160 | Bo K. Bergman, Gunilla E. Larsson, Bo G. Brismar and Marie E. Klang | Battered Women—Their Susceptibility to Treatment |
| 1988 | 16 | 3 | 161-166 | Gunilla Larsson and Mona Andersson | Violence in the Family: Morbidity and Medical Consumption |
| 1988 | 16 | 3 | 167-171 | Fredrik Almqvist | Disadvantaged Adolescents in Psychiatric Treatment |
| 1988 | 16 | 3 | 173-178 | Gunilla Brattberg | Priority Setting with Regard to Placement on Waiting List to a Pain Clinic: The Feasibility of a Delegated Ranking Procedure |
| 1988 | 16 | 3 | 179-182 | Anders Eriksson, Ulf Björnstig and Krister Kullenberg | Snowy Roofs—a Potential Hazard |
| 1988 | 16 | 3 | 183-186 | Lottie Wicksell, John M. Carstensen, Gunnar Eklund and Jan-Åke Gustafsson | Lung Cancer Incidence among Swedish Bakers and Pastrycooks: Geographical Variation |
| 1988 | 16 | 3 | 187-191 | Veijo J. Notkola and Kaj R. Husman | Mortality among Female Farmers in Finland in 1979-1985 |
| 1988 | 16 | 3 | 192 | Jørn Olsen | Developments in Public Health Research in the Nordic Countries |
| 1988 | 16 | 4 | 193-195 | Niels Lynöe | Consensus and Consensus Conferences |
| 1988 | 16 | 4 | 197-204 | Marie-Louise Sandén and Per Bjurulf | Pregnant Women's Attitudes and Knowledge in Relation to Access to Serum-alpha-fetoprotein Test |
| 1988 | 16 | 4 | 205-208 | Jørn Olsen | Limitations in the Use of Job Exposure Matrices |
| 1988 | 16 | 4 | 209-215 | Troels Lyngbye, Ole Noerby Hansen and Philippe Grandjean | Bias from Non-participation: A Study of Low-level Lead Exposure in Children |
| 1988 | 16 | 4 | 217-221 | Jørn Olsen | Methodological Problems in the Studies of Reproductive Failures |
| 1988 | 16 | 4 | 223-226 | I. H. Monrad Aas | Quality of Hospital Data and DRGs |
| 1988 | 16 | 4 | 227-232 | Björn Söderfeldt, Berth Danermark and Sven Larsson | Income Redistribution Effect of the Swedish Sickness Allowance Insurance in a Comparison of Two Concepts of Social Class |
| 1988 | 16 | 4 | 233-234 | Leif Svanström and Åke Thörn | Research Development in Nicaragua |
| 1988 | 16 | 4 | 235-236 | Rafael Amador Rodezno, Julio Piura Lopez and Rodolfo Pena Garcia | Research and Development at the Department of Preventive Medicine at the Medical School of the "Universidad Nacional Autonoma de Nicaragua", León, Nicaragua |
| 1988 | 16 | 4 | 237-240 | Aurora Aragon, Rodolfo Pena, Leif Svanström and Åke Thörn | Development of a Community Intervention Programme on Miners' Health and Working Environment in Nicaragua |
| 1988 | 16 | 4 | 241-250 | Pekka Puska, Helena Niemensivu, Pertti Puhakka, Liisa Alhainen, Kaj Koskela, Simo Moisio, Simo Mosio and Liisa Viri | Results of a One-year Worksite and Mass Media Based Intervention on Health Behaviour and Chronic Disease Risk Factors |
| 1988 | 16 | 4 | 251-255 | Dimitrios Trichopoulos and Eleni Petridou | Promoting Health among School Age Children |
| 1988 | 16 | 4 | 257-263 | Pascal Guenel, Henrik Møller and Elsebeth Lynge | Incidence of the Upper Respiratory and Digestive Tract Cancers and Consumption of Alcohol and Tobacco in Denmark |
| 1988 | 16 | 4 | 265-271 | Marie-Louise Sandén and Per Bjurulf | Pregnant Women's Attitudes for Accepting or Declining a Serum-alpha-fetoprotein Test |
| 1988 | 16 | 4 | 273-276 | Henrik Hasle, Bernard Jeune and Axel Skytthe | Differential Mortality among Semiskilled Applicants of Disability Pension |
| 1988 | 16 | 4 | 277-282 | Sonja Vaglum, Per Vaglum and Øivind Larsen | Family Risk Factors of Alcoholism and Drinking Patterns among Non Alcoholic Women: an Inverse Relationship? |
| 1988 | 16 | 4 | 283-285 | Ossi Rahkonen, Hannele Palosuo and Elina Hemminki | The Place of Medical Sociology |
| 1988 | 16 | 4 | 287-288 | Niels Lynöe | Book Review: A Danish Consensus Report on Secretory Otitis Media (Glue Ear) |
| 1989 | 17 | 1 | 1 | Editorial | A bidecennial anniversary |
| 1989 | 17 | 1 | 3-6 | Elianne Riska | How the Sex Differences in the Use of Psychotropic Drugs Are Explained |
| 1989 | 17 | 1 | 7-11 | Bjarne Jansson and Leif Svanström | Evaluation of a System for Injury Surveillance in Swedish Emergency Care |
| 1989 | 17 | 1 | 13-20 | Hans D. Petersen | The Controlled Study of Torture Victims: Epidemiological Considerations and Some Future Aspects |
| 1989 | 17 | 1 | 21-24 | Irma Moilanen and Antero Myhrman | What Protects a Child during Migration? |
| 1989 | 17 | 1 | 25-31 | J. Neiderud | Greek Immigrant Children in Southern Sweden in Comparison with Greek and Swedish Children: I. General Living Conditions |
| 1989 | 17 | 1 | 33-38 | Malcolm Rees | HIV Infectiousness and the AIDS Epidemic |
| 1989 | 17 | 1 | 39-48 | Claes Herlitz and Bengt Brorsson | The AIDS Epidemic in Sweden: Estimates of Costs, 1986, 1987 and 1990 |
| 1989 | 17 | 1 | 49-58 | Denny Vågerö and Staffan E. Norell | Mortality and Social Class in Sweden — Exploring a New Epidemiological Tool |
| 1989 | 17 | 1 | 59-65 | Lars Lidberg, Nils Wiklund and Sten W. Jakobsson | Mortality among Criminals with Suspected Mental Disturbance |
| 1989 | 17 | 1 | 67-75 | Ulla-Kaija Lammi, Sirkka-Liisa Kivelä, Aulikki Nissinen, Juha Pekkanen and Sven Punsar | Functional Capacity and Associated Factors in Elderly Finnish Men |
| 1989 | 17 | 1 | 77-84 | Lennart Dimberg, Ari Olafsson, Eythor Stefansson, Hans Aagaard, Anders Odén, Gunnar B. J. Andersson, Carl-Göran Hagert and Tommy Hansson | Sickness Absenteeism in an Engineering Industry—an Analysis with Special Reference to Absence for Neck and Upper Extremity Symptoms |
| 1989 | 17 | 1 | 85-91 | Ann-Margret Rydell | School Health Services as Prescribed and Practised—Do They Correspond? |
| 1989 | 17 | 1 | 93-102 | John Gunnar Mæland and Odd Erik Havik | Use of Health Services after a Myocardial Infarction |
| 1989 | 17 | 1 | 103-107 | Elisabeth Arborelius and Ditte Lindell | Psychological Aspects of Early and Late Discharge after Hospital Delivery: An Interview Study of 44 Families |
| 1989 | 17 | 1 | 109-119 | Synnøve Fønnebø Knutsen and Raymond Knutsen | The Tromsø Heart Study: Family Approach to Intervention on CHD: Feasibility of Risk Factor Reduction in High-risk Persons—Project Description |
| 1989 | 17 | 1 | 121-122 | Denny Vågerö and Claes-Göran Stefansson | LETTER TO THE EDITOR |
| 1989 | 17 | 1 | 123-124 | Björn Söderfeldt, Sven Larsson and Berth Danermark | LETTER TO THE EDITOR |
| 1989 | 17 | 1 | 125 | Gunnar Ahlborg Jr | Reviewed Work: Occupational Medicine - Principles and Practical Applications. 2nd ed. by C. Zenz |
| 1989 | 17 | 1 | 126 | Mikael Sandlund | Reviewed Work: Community Health Aide/Practitioner Manual by Robert D. Burgess |
| 1989 | 17 | 1 | 127 | Jørn Olsen | Reviewed Work: The role of HIV testing in AIDS preventation by B. Kutchinsky |
| 1989 | 17 | 1 | 128 | Gunnar Kaati | Reviewed Work: Dealing with Medical Malpractice. The British and Swedish Experience by M. M. Rosenthal |
| 1989 | 17 | 2 | 129-133 | Anne Helset and Grete Botten | Local action groups in prevention of child accidents |
| 1989 | 17 | 2 | 135-140 | Johanne Sundby | Methodological considerations in the study of frequency, risk factors and outcome of reduced fertility |
| 1989 | 17 | 2 | 141-145 | Calle Bengtsson, Thomas Gredmark, Leif Hallberg, Tore Hällström, Björn Isaksson, Leif Lapidus, Olof Lindquist, Sven Lindstedt, Mirek Lurie, Ernst Nyström, Göran Rybo, Sverker Samuelsson, Vilhjalmur Rafnsson and Johann A. Sigurdsson | The Population Study of Women in Gothenburg 1980-81—the Third Phase of a Longitudinal Study: Comparison between Participants and Non-Participants |
| 1989 | 17 | 2 | 147-150 | E Greve | Computerized tomography findings among workers with chronic intoxication of the brain |
| 1989 | 17 | 2 | 151-156 | Arja Rimpelä and Juha Teperi | Respiratory Symptoms and Low Tar Cigarette Smoking—a Longitudinal Study on Young People |
| 1989 | 17 | 2 | 157-164 | O. Lyngenbo, S. Groth, M. Groth, O. Olsen and N. Rossing | Occupational Lung Function Impairment in Never-smoking Danish Welders |
| 1989 | 17 | 2 | 165-169 | Kerstin Isaksson | Unemployment, Mental Health and the Psychological Functions of Work in Male Welfare Clients in Stockholm |
| 1989 | 17 | 2 | 171-179 | M. Bågedahl-Strindlund, L. Rosencrantz-Larsson and P. Wilkner-Svanfeldt | Children of Mentally Ill Mothers: Social Situation and Psychometric Testing of Mental Development |
| 1989 | 17 | 2 | 181-191 | B. Wiberg, K. Humble and P. de Château | Long-term effect on mother-infant behaviour of extra contact during the first hour post partum. V. Follow-up at three years |
| 1989 | 17 | 2 | 193-201 | Ingalill Morén-Hybbinette, Ulrich Moritz and Bengt Scherstén | Diabetes mellitus and disability pension: A Descriptive Study of All Diabetic Subjects Granted Disability Pension in Sweden in 1980 |
| 1989 | 17 | 2 | 203-206 | Bo Netterstrøm and Knud Juel | Low Back Trouble among Urban Bus Drivers in Denmark |
| 1989 | 17 | 2 | 207-215 | Björn Söderfeldt, Berth Danermark and Sven Larsson | Class Inequality in Health: A Methodological Study of Two Measures of Social Class in Relation to Sickness Insurance Diagnoses |
| 1989 | 17 | 2 | 217-222 | Göran Westman, Carl-Gunnar Eriksson and Hampus von Post | Health Care Utilization and Attitudes to Health Care before and after Development Work in a Health Centre: Results from two independent postal surveys |
| 1989 | 17 | 2 | 223 | Svend Juul | Reviewed Work: Statistical Methods in Cancer Research. Volume II - The Design and Analysis of Cohort Studies. IARC Scientific Publications No. 82 by NE Breslow, NE Day |
| 1989 | 17 | 2 | 223-224 | Johannes Ipsen | Reviewed Work: Atlas of Cancer Incidence in the Nordic Countries by O. M. Jensen, B. Carstensen, E. Glattre, E. Malker, E. Pukkala, H. Tulinius |
| 1989 | 17 | 3 | 225-226 | Lars Olov Bygren | Doctors on salary |
| 1989 | 17 | 3 | 227-230 | Bo J. A. Haglund | Preventive activities in primary health care in Stockholm county, Sweden: Results from a survey in November 1985 |
| 1989 | 17 | 3 | 231-237 | Gudrun E. Hedberg and Stina M. Langendoen | Factors Influencing the Turnover of Swedish Professional Drivers |
| 1989 | 17 | 3 | 239-244 | Helena Gylling and Olii Simonen | Use of hospital care and hospital case-fatality of myocardial infarction during three years' follow-up in Finland |
| 1989 | 17 | 3 | 245-251 | Gunnar Tellnes and Tor Bjerkedal | Epidemiology of Sickness Certification – A methodological approach based on a study from Buskerud county in Norway |
| 1989 | 17 | 3 | 253-256 | Bengt Brorsson | The Risk of Accidents Among Older Drivers |
| 1989 | 17 | 4 | 257-263 | Niels Lynöe | Theoretical and empirical problems in the assessment of alternative medical technologies |
| 1989 | 17 | 4 | 265-270 | Piroska Östlin | The 'health-related selection effect' on occupational morbidity rates |
| 1989 | 17 | 4 | 271-276 | Margit Groth and Ole Lyngenbo | Respiratory Symptoms in Danish Welders |
| 1989 | 17 | 4 | 277-280 | Jørn Olsen, Grethe Frische, Anni O. Poulsen and Helle Kirchheiner | Changing Smoking, Drinking, and Eating Behaviour among Pregnant Women in Denmark: Evaluation of a health campaign in a local region |
| 1989 | 17 | 4 | 281-286 | K. Schmidt, A. Krasnik, E. Brendstrup, H. Zoffmann and S. O. Larsen | Attitudes towards HIV infection and sexual risk behaviour: A survey among Danish men 16-55 years of age |
| 1989 | 17 | 4 | 287-290 | Bengt Brorsson | Age and Injury Severity |
| 1989 | 17 | 4 | 291-299 | Helen Hansagi, Peter Allebeck and Olof Edhag | Health care utilization after referral from a hospital emergency department |
| 1990 | 18 | 1 | 1-8 | A J Fox | Socio-economic Differences in Mortality and Morbidity |
| 1990 | 18 | 1 | 9-15 | Andréasson S. and Allebeck P. | Cannabis and mortality among young men: A longitudinal study of Swedish conscripts |
| 1990 | 18 | 1 | 17-23 | Birger Kaada and Erling Sivertsen | Sudden Infant Death Syndrome During Weekends and Holidays in Norway in 1967-1985 |
| 1990 | 18 | 1 | 25-30 | Anna Bexell, Lennart Råstam, Sven-Olof Isacsson and Leif Ingvarsson | Parents' response to recurrent middle ear infection in their children |
| 1990 | 18 | 1 | 31-37 | Dagmar Lagerberg, Ulf Janson, Ulf Jansson, Claes Sundelin and Gunilla Larsson | Services to Families with Children: A Study of Community Work in Uppsala |
| 1990 | 18 | 1 | 39-44 | Per Winkel, Bernard E. Statland, Mogens E. Brammer, Bjørn Christau and Erik Rahbek Østergaard | Present and Projected Consumption of Hospital Bed-days as a Function of Terminal Days Before Death |
| 1990 | 18 | 1 | 45-51 | Joep Perk, Bo Hedbäck and Jan Engvall | Effects of Cardiac Rehabilitation after Coronary Artery Bypass Grafting on Readmissions, Return to Work, and Physical Fitness: A Case-control Study |
| 1990 | 18 | 1 | 53-57 | Hans Olaf Bang | Lipid Research in Greenland: Preventive and Therapeutic Consequences |
| 1990 | 18 | 1 | 59-61 | Ilkka Antti-Poika, Timo Pohjolainen and Hannu Alaranta | Severe Frostbite of the Upper Extremities - a Psychosocial Problem Mostly Associated with Alcohol Abuse |
| 1990 | 18 | 1 | 63-68 | Berit Schei | Prevalence of Sexual Abuse History in a Random Sample of Norwegian Women |
| 1990 | 18 | 1 | 69-79 | Jan Olof Hörnquist | Quality of Life: Concept and Assessment |
| 1990 | 18 | 2 | 81-89 | P. Dalla-Vorgia, A.J. Sasco, Y. Skalkidis, K. Katsouyanni and D. Trichopoulos | An Evaluation of the Effectiveness of Tobacco-Control Legislative Policies in European Community Countries |
| 1990 | 18 | 2 | 91-95 | J. O. Hörnquist, B. Hansson, M. Leijon and B. Mikaelsson | Repeated Short-Term Sick-Leave and Quality of Life: An Evaluation of a Clinical Socio-Medical Intervention |
| 1990 | 18 | 2 | 97-102 | Bo Netterstrøm and Knud Juel | Peptic ulcer among urban bus drivers in Denmark |
| 1990 | 18 | 2 | 103-113 | Allan Krasnik, Jan R. Fouchard, Thomas Bayer and Niels Keiding | Health Workers and AIDS: Knowledge, Attitudes and Experiences as Determinants of Anxiety |
| 1990 | 18 | 2 | 115-124 | Gun M. Nordström, Claes R. Nyman and Töres Theorell | The Impact on work Ability of Ileal Conduit Urinary Diversion |
| 1990 | 18 | 2 | 125-132 | Béatrice Kvist, Mårten Kvist and Jukka Rajantie | School Absences, School Achievements and Personality Traits of the Haemophilic Child |
| 1990 | 18 | 2 | 133-138 | Monica Löfvander and Dimitri Papastavrou | Clinical Factors, Psycho-Social Stressors and Sick-Leave Patterns in a Group of Swedish and Greek Patients |
| 1990 | 18 | 2 | 139-142 | Bjarne Jansson and Carl-Gunnar Eriksson | Accident Involvement and Attitudes towards Hazards and Countermeasures in a Swedish Rural Population |
| 1990 | 18 | 2 | 142-148 | Sven Cnattingius, Anders Ericson, Jan Gunnarskog and Bengt Källén | A Quality Study of a Medical Birth Registry |
| 1990 | 18 | 2 | 149-153 | Niels Lynöe | Is the Effect of Alternative Medical Treatment Only a Placebo Effect? |
| 1990 | 18 | 2 | 155-156 | Kenneth Wind-Andersen | Review: The urban poor  Reviewed Work: In the Shadow of the City, Community Health and the Urban Poor by T. Harphan, P. Vaughan, L. Lusty |
| 1990 | 18 | 2 | 157 | Jørn Olsen | Review: Practical guide for critical appraisal  Reviewed Work: Causal relationships in medicine. A practical system for critical appraisal by J. Mark Elwood |
| 1990 | 18 | 2 | 158 | Rebecca Fuhrer | Review: Epidemiology and the Prevention of Mental Disorders  Reviewed Work: World Psychiatric Association, eds. Epidemiology and the Prevention of Mental Disorders by B Cooper, T. Helgason |
| 1990 | 18 | 3 | 161-164 | Elianne K. Riska | Strategies in the Education of Humane Physicians |
| 1990 | 18 | 3 | 165-169 | Leif Svanström | Simply osteoporosis – or multifactorial genesis for the increasing incidence of fall injuries in the elderly? The need for a scientific approach to reducing injuries |
| 1990 | 18 | 3 | 171-174 | Helge Kjersem and Søren Jepsen | Varicella Among Immigrants from the Tropics, a Health Problem |
| 1990 | 18 | 3 | 175-178 | Helge Kjersem, Søren Jepsen, Leith Larsen and Finn Black | Salmonella and "Shigella" Carriers Among Refugees from the Middle East and Sri Lanka in Denmark |
| 1990 | 18 | 3 | 179-183 | Finn Tüchsen and Ursula Zebitz | Work and "lifestyle" in occupational mortality in Denmark |
| 1990 | 18 | 3 | 185-193 | V Fønnebø and A J Søgaard | The Penetrating Educational Effect of a Mass-Media Based Fund-Raising Campaign "Heart for Life" |
| 1990 | 18 | 3 | 195-201 | M. Fakhr El-Islam and Sanaa I. Abu-Dagga | Illness behaviour in mental ill-health in Kuwait |
| 1990 | 18 | 3 | 203-206 | Erik Bendvold, Narve Moe and Julie Skjæraasen | Social Conditions of Children born after Artificial Insemination by Donor |
| 1990 | 18 | 3 | 207-211 | Nan B Oldereid, Hallgeir Rui and Kenneth Purvis | Male Partners in Infertile Couples. Personal Attitudes and Contact with the Norwegian Health Service |
| 1990 | 18 | 3 | 213-219 | Hans Hallberg and Bengt Mattsson | Premature Deaths Among Men in a Swedish Municipality – Civil Status and Primary Health Care Utilization Prior to Death |
| 1990 | 18 | 3 | 221-224 | Sven Andréasson, Marianne Parmander and Peter Allebeck | A Trial that Failed, and the Reasons Why: Comparing the Minnesota Model with Outpatient Treatment and Non-treatment for Alcohol Disorders |
| 1990 | 18 | 3 | 225-229 | Guttorm Brattebø and Torben Wisborg | HIV Infection and Health Personnel: Health Care Workers' Opinions Concerning Some Ethical Dilemmas |
| 1990 | 18 | 3 | 231-235 | Anne Eskild, Per Magnus, Øyvind Nilsen and Ingar Holme | Survival of AIDS Patients in Norway |
| 1990 | 18 | 3 | 237 | Anders Ahlbom | Reviewed Work: Surveillance in Health and Disease by WJ Eylenbosch, NG Noah |
| 1990 | 18 | 3 | 238 | Guðjón Magnússon | Reviewed Work: Public Health and Human Ecology by John M. Last |
| 1990 | 18 | 4 | 239-240 | Niels Lynoe | Consensus conferences — A postmodern phenomenon? |
| 1990 | 18 | 4 | 241-247 | Heikki S. Vuorinen | Family Resources and Children's Use of Primary Health Care Services in Finland in 1979 |
| 1990 | 18 | 4 | 249-255 | Helen Hansagi | Referral of non-urgent cases from an emergency department: patient compliance, satisfaction and attitudes |
| 1990 | 18 | 4 | 257-261 | Risto J. Honkanen and Raija Mönkkönen | Reliability of causal indicators for nonfatal injuries |
| 1990 | 18 | 4 | 263-267 | Ulf Björnstig, Christer Hildingsson and Göran Toolanen | Soft-Tissue injury of the neck in a hospital based material |
| 1990 | 18 | 4 | 269-271 | Bent Erling Lindblad, Carsten Juhl Terkelsen and Lisbeth Nørgaard Lindblad | Product-related childhood accidents A survey of 1590 cases |
| 1990 | 18 | 4 | 273 | JD Urschel, JW Urschel and WC Mackenzie | The Role of Alcohol in Frostbite Injury |
| 1990 | 18 | 4 | 275-280 | Tomas Svensson and Mikael Sandlund | Ethics and Preventive Medicine |
| 1990 | 18 | 4 | 281-286 | Asmerom Kidane | Mortality estimates of the 1984-85 Ethiopian famine |
| 1991 | 19 | 1 | 1-6 | Jørn Olsen | Causes and Prevention |
| 1991 | 19 | 1 | 7-13 | Bo Wärneryd, Mats Thorslund and Piroska Östlin | The quality of retrospective questions about occupational history – a comparison between survey and census data |
| 1991 | 19 | 1 | 14-19 | Thorkild I. A. Sørensen | Genetic epidemiology utilizing the adoption method: Studies of obesity and of premature death in adults: Held as the Ipsen Lecture 18th May, 1990 |
| 1991 | 19 | 1 | 20-25 | Carl-Göran Ohlson, Lennart Bodin, Ing-Liss Bryngelsson, Marie Helsing and Lars Malmberg | Winter weather conditions and myocardial infarctions |
| 1991 | 19 | 1 | 26-31 | Berit Schei, Sven Ove Samuelsen and Leiv S. Bakketeig | Does Spousal Physical Abuse Affect the Outcome of Pregnancy? |
| 1991 | 19 | 1 | 32-38 | Katri Makkonen and Elina Hemminki | Different contraceptive practices: Use of contraceptives in Finland and other Nordic countries in the 1970s and 1980s |
| 1991 | 19 | 1 | 39-43 | Susanne K. Kjær, Edith I. Svare, Birthe J. Haugaard, Chantal Teisen, René B. Christensen, Elsebeth Lynge and Ole M. Jensen | Contraceptive Use in Random Samples of Greenlandic and Danish Women – Changes from 1986 to 1988 |
| 1991 | 19 | 1 | 44-46 | Bernard Jeune and Hanne Wielandt | Prevalence of Smoking and Oral Contraception in a Sample of Danish Young Women |
| 1991 | 19 | 1 | 47-52 | Timo A. Virtanen, Irma K. Moilanen and Minna M. Ihalainen | What Causes Stress for Mothers of Children with MBD? |
| 1991 | 19 | 1 | 53-56 | Claes-Göran Westrin | Strategies implemented but goals not attained: Some comments on an evaluation of the Swedish mental health services |
| 1991 | 19 | 1 | 57-65 | Berit Rokne Hanestad, Jan Olof Hörnquist and Grethe Albrektsen | Self-assessed Quality of Life and Metabolic Control in Persons with Insulin-Dependent Diabetes Mellitus (IDDM) |
| 1991 | 19 | 1 | 66-71 | Laura Jalkanen | The effect of a weight reduction program on cardiovascular risk factors among overweight hypertensives in primary health care |
| 1991 | 19 | 1 | 72-78 | Sven-Arne Jacobsson, Clas Rehnberg and Krister Djerf | Risks, Benefits and Economic Consequences of Total Hip Arthroplasty in an Aged Population |
| 1991 | 19 | 1 | 79 | Hannu Vuori | Reviewed Work: Uteliggarna i välfärdssamhället (The homeless in the welfate society) by L Stenberg, L Svanström, S Åhs |
| 1991 | 19 | 1 | 80 | Henning Poulsen | Reviewed Work: Disablement in the community by DL Patrick, H Peach |
| 1991 | 19 | 2 | 81-85 | Bengt Haglund, Tore Nilstun, Björn Smed and Claes-Göran Westrin | Longitudinal studies on environmental factors and disease: A model for analysis of ethical conflicts |
| 1991 | 19 | 2 | 86-93 | Catharina Gåfvels, Bengt Börjesson and Folke Lithner | The social consequences of insulin-treated diabetes mellitus in patients 20-50 years of age: An epidemiological case-control study |
| 1991 | 19 | 2 | 94-98 | VANJA BLOMQVIST, LENA JONSSON and TÖRES THEORELL | Life events and coping patterns reported in HIV-infected hemophiliacs a year after diagnosis |
| 1991 | 19 | 2 | 99-104 | Jan Olof Hörnquist and Birgit Hansson | Long-term sick-listing, medical rehabilitation and life quality change: A patient evaluation |
| 1991 | 19 | 2 | 105-109 | Vibeke Vestermark, Claus K. Høgdall, Grete Plenov, Michael Birch and Kim Toftager-Larsen | The duration of breast-feeding: A longitudinal prospective study in Denmark |
| 1991 | 19 | 2 | 110-115 | Willy Pedersen and Nils Johan Lavik | Role modelling and cigarette smoking: vulnerable working class girls? A longitudinal study |
| 1991 | 19 | 2 | 116-126 | Lars Møller, Tage S. Kristensen and Hanne Hollnagel | Social Class and Cardiovascular Risk Factors in Danish Men |
| 1991 | 19 | 2 | 127-133 | Karen Vitting Andersen, Aksel P. Lange and Karin Helweg-Larsen | A Perinatal Audit of Stillbirths in Three Danish Counties |
| 1991 | 19 | 2 | 134-139 | Kerstin Hulter Åsberg and Anders Parrow | Event, incidence, and fatality rates of cerebrovascular diseases in Enköping-Håbo, Sweden, 1986-1988 |
| 1991 | 19 | 2 | 140 | Marianne Kastrup | Reviewed Work: Den ojämlika ohälsan by O Lundberg |
| 1991 | 19 | 2 | 140-141 | L. R. Karhausen | Reviewed Work: Philosophy of Medicine - An Introduction. 2. edition by HR Wulff, SA Pedersen, R Rosenberg |
| 1991 | 19 | 2 | 141-142 | Sigurd Mikkelsen | Reviewed Work: The Year Book of Occupational and Environmental Medicine 1990 by EA Emmet, SM Brooks, RL Harris, MB Schenker |
| 1991 | 19 | 2 | 142 | Erik Lykkegaard | Reviewed Work: Color Atlas of Child Sexual Abuse by DL Chadwick, Berkowitz |
| 1991 | 19 | 2 | 142 | Gert Almind | Reviewed Work: Nye tider - nye œldre (other times, other elderly) by M Schroll |
| 1991 | 19 | 2 | 142-143 | Povl Munk-Jørgensen | Reviewed Work: The Incidence of Mental Illness over a Quarter of a Century by O Hagnell, E Essen-Möller, J Lanke, L Ôjesjö, B Rorsman |
| 1991 | 19 | 3 | 145-147 | Leiv S. Bakketeig | Perinatal Epidemiology - A Nordic Challenge |
| 1991 | 19 | 3 | 148-153 | Lars O. Ljungdahl and Per Bjurulf | The accordance of diagnoses in a computerized sick-leave register with doctor's certificates and medical records |
| 1991 | 19 | 3 | 154-161 | Christina Nerbrand, Kurt Svärdsudd, Lars-Gunnar Hörte and Gösta Tibblin | Are geographical differences in cardiovascular mortality due to morbidity differences or to methodological differences? The Project "Myocardial Infarction in Mid-Sweden" |
| 1991 | 19 | 3 | 162-169 | Torsten Skov and Elsebeth Lynge | Non-Hodgkin's lymphoma and occupation in Denmark |
| 1991 | 19 | 3 | 170-173 | Hólmfriður Gunnarsdóttir and Vilhjálmur Rafnsson | Cancer Incidence among Icelandic Farmers 1977-1987 |
| 1991 | 19 | 3 | 174-180 | Britta-Lena Rundcrantz, Birgitta Johnsson, Ulrich Moritz and Gertrud Roxendal | Occupational cervico-brachial disorders among dentists: Psychosocial work environment, personal harmony and life-satisfaction |
| 1991 | 19 | 3 | 181-186 | Hans Hallberg and Bengt Mattsson | Premature deaths among men in a Swedish municipality – social characteristics prior to death |
| 1991 | 19 | 3 | 187-189 | Sune Nyström, Lars Olof Bygren and Daniel R. Vining Jr | Reproduction and level of intelligence |
| 1991 | 19 | 3 | 190-198 | Gunilla Bjärås | The need of leadership for motivation of participants in a community intervention programme |
| 1991 | 19 | 3 | 199-204 | Elina Hemminki, Taina Mäntyranta, Maili Malin and Päivikki Koponen | A survey on the use of alternative drugs during pregnancy |
| 1991 | 19 | 3 | 205-207 | Maja-Lisa Løchen | University Medical School in Tromsø, Norway |
| 1991 | 19 | 4 | 209-217 | Nancy Milio | Toward Healthy Longevity: Lessons in Food and Nutrition Policy Development from Finland and Norway |
| 1991 | 19 | 4 | 218-224 | Cecilia Björkelund and Calle Bengtsson | Cardiovascular Risk Factor Characterisation of Women in the Community of Strömstad, Sweden, Compared With Other Female Populations |
| 1991 | 19 | 4 | 225-234 | Per-Olof Östergren, Michael Freitag, Bertil S Hanson, Eva Hedin, Sven-Olof Isacsson, Håkan Odeberg and Sven-Eric Svensson | Social network and social support predict improvement of physical working capacity in rehabilitation of patients with first myocardial infarction |
| 1991 | 19 | 4 | 235-241 | Jørn Olsen and Sjurdur F. Olsen | A Suggestion for Improving Intelligibility in Multivariate Confounder Adjustment Using Alcohol Intake and Birth Weight as an Example: A 'Confounder Score' Approach in Analyzing Continuous Data |
| 1991 | 19 | 4 | 242-247 | Peter Allebeck, Christer Allgulander, Lars Henningsohn and Sten W. Jakobsson | Causes of death in a cohort of 50 465 young men – Validity of recorded suicide as underlying cause of death |
| 1991 | 19 | 4 | 248-255 | Heikki S. Vuorinen | Social variation in infant mortality in a core city of Finland during the 19th and early 20th centuries: Positive effect of industrialization? |
| 1991 | 19 | 4 | 245-259 | Jan van Reek and Hans Adriaanse | Smoking by physicians in Scandinavia: 1952-1989 |
| 1991 | 19 | 4 | 260-261 | Joseph M. Merrill, Lila F. Laux and John I. Thornby | LETTER TO THE EDITOR |
| 1992 | 20 | 1 | 1-4 | Nils-Holger Areskog | The new medical education at the Faculty of Health Sciences, Linköping University — A challenge for both students and teachers |
| 1992 | 20 | 1 | 5-10 | Lars Cernerud | Differences in height between socially more and less privileged 10 year old Stockholm children born in 1933-1963 |
| 1992 | 20 | 1 | 11-13 | Sven Cnattingius and Bengt Haglund | Socio-Economic Factors and Feto-Infant Mortality |
| 1992 | 20 | 1 | 14-18 | Urban Janlert, Kjell Asplund and Lars Weinehall | Unemployment and cardiovascular risk indicators: Data from the MONICA survey in northern Sweden |
| 1992 | 20 | 1 | 19-24 | Claes Herlitz | The HIV pandemic in Sweden: What do people really think about mandatory HIV testing? |
| 1992 | 20 | 1 | 25-30 | Anders I. Larsen, Inger Schaumburg and Anni L. Larsen | Management of occupational risk to reproduction in a Danish county |
| 1992 | 20 | 1 | 31-36 | Vingård Eva, Alfredsson Lars, Fellenius Evy and Hogstedt Christer | Disability pensions due to musculo-skeletal disorders among men in heavy occupations: A case-control study |
| 1992 | 20 | 1 | 37-44 | Inga-Britt Bränholm and Else-Ann Degerman | Life satisfaction and Activity Preferences in Parents of Down's syndrome Children |
| 1992 | 20 | 1 | 45-50 | Lars Hansson and Mats Berglund | Stability of therapeutic alliance and its relationship to outcome in short-term inpatient psychiatric care |
| 1992 | 20 | 1 | 51-54 | Kiki Tsamandouraki, Yannis Tountas and Dimitrios Trichopoulos | Relative survival of terminal cancer patients in home versus hospital care |
| 1992 | 20 | 1 | 55-60 | Niels Lynöe and Tomas Svensson | Kiki Tsamandouraki, Yannis Tountas and Dimitrios Trichopoulos |
| 1992 | 20 | 1 | 61 | Kim Overvad | Reviewed Work: Nutrition policy for food-rich countries. A strategic analysis. by Nancy Milio |
| 1992 | 20 | 2 | 65-72 | A. Hultén and D. Wasserman | Suicide among young people aged 10-29 in Sweden |
| 1992 | 20 | 2 | 73-78 | Jaakko M. Teikari, Jaakko Kaprio, Markku Koskenvuo and James O'Donnell | Heritability of defects of far vision in young adults — a twin study |
| 1992 | 20 | 2 | 79-84 | Sari K. Anttila | Diseases and symptoms as predictors of hospital care in an aged population: A prospective register-based study |
| 1992 | 20 | 2 | 85-86 | Tapio Joensuu and Simo Näyhä | Reliability of Hospital Discharge Diagnoses of Acute Myocardial Infarction |
| 1992 | 20 | 2 | 87-93 | Henrik Sælan, Lars Møller and Anne Køster | Alcohol consumption in a Danish cohort during 11 years |
| 1992 | 20 | 2 | 94-101 | Marlene Stenbacka, Peter Allebeck, Lena Brandt and Anders Romelsjö | Intravenous drug abuse in young men: Risk factors assessed in a longitudinal perspective |
| 1992 | 20 | 2 | 102-109 | Claes Herlitz | Condom use due to the risk of AIDS: Trends in the general population of Sweden |
| 1992 | 20 | 2 | 110-114 | Inger Schaumburg and Jesper L. Boldsen | Waiting time to pregnancy and pregnancy outcome among Danish workers in the textile, clothing, and footwear industries |
| 1992 | 20 | 2 | 115-118 | Vicenta Escribà-Agüir | Nurses' Attitudes Towards Shiftwork and Quality of Life |
| 1992 | 20 | 2 | 119-126 | Raija M. T. Laukkanen, Pekka Oja, Katriina H. Ojala, Matti E. Pasanen and Ilkka M. Vuori | Feasibility of a 2-km walking test for fitness assessment in a population study |
| 1992 | 20 | 2 | 127 | Virve Siirak | LETTER TO THE EDITOR |
| 1992 | 20 | 2 | 128 | Jørn Olsen | Reviewed Work: Public Health & Preventive Medicine. 13th edition by JM Last, RB Wallace |
| 1992 | 20 | 3 | 129-133 | Tómas Helgason | Epidemiological research needs access to data |
| 1992 | 20 | 3 | 134-142 | Gunnar Ågren and Anders Romelsjö | Mortality in alcohol-related diseases in Sweden during 1971-80 in relation to occupation, marital status and citizenship in 1970 |
| 1992 | 20 | 3 | 143-150 | Torild Hammer and Per Vaglum | Further course of mental health and use of alcohol and tranquilizers after cessation or persistence of cannabis use in young adulthood: A longitudinal study |
| 1992 | 20 | 3 | 151-157 | Merete Osler, Jørgen Lous and Niels K. Rasmussen | Knowledge, attitudes and cardiovascular risk factors in Danish adults |
| 1992 | 20 | 3 | 158-164 | Lillian Werdelin, Jens Misfeldt, Mads Melbye and Jørn Olsen | Merete Osler, Jørgen Lous and Niels K. Rasmussen |
| 1992 | 20 | 3 | 165-172 | Liisa Hyssälä, Päivi Rautava and Matti Sillanpää | Health Behaviour of Fathers of Young Families Expecting their First Baby |
| 1992 | 20 | 3 | 173-178 | Ann-Charlotte Sandstedt, Vivi Ann Carlsson, Jan Olof Hörnquist and Mikael Thyberg | War-wounded refugees. Quality of life after 2-3 years in Sweden |
| 1992 | 20 | 3 | 179-183 | Bo K. Bergman and Bo G. Brismar | Do not forget the battered male! A comparative study of family and non-family violence victims |
| 1992 | 20 | 3 | 184-187 | Bjarne K. Jacobsen, Inger Stensvold, Knut Fylkesnes, Ivar S. Kristiansen and Dag S. Thelle | The Nordland Health Study: Design of the study, description of the population, attendance and questionnaire response |
| 1992 | 20 | 3 | 188-189 | Alicja Kuskowska-Wolk and Stephan Rössner | Self-reported weight and height considerably affect the weight distribution of a population |
| 1992 | 20 | 3 | 190-191 | Tine Brink Henriksen | Reviewed Work: Making sense of Data-A self instruction Manual on the Interpretation of Epidemiological Data by JH. Abramson |
| 1992 | 20 | 4 | 193-195 | Per Magnus | Genetic epidemiology - possibilities and problems |
| 1992 | 20 | 4 | 196-203 | Arne T. Høstmark, John Berg, Stein Brudal, Steinar R. Berge, Peter Kierulf and Tor Bjerkedal | Coronary risk factors in middle-aged men as related to smoking, coffee intake and physical activity |
| 1992 | 20 | 4 | 204-208 | Bjarne Koster Jacobsen and Inger Stensvold | Milk - a better drink?: Relationships with total serum cholesterol in a cross-sectional survey. The Nordland Health Study |
| 1992 | 20 | 4 | 209-212 | Riitta Luoto, Elina Hemminki, Päivi Topo, Antti Uutela and Ilka Kangas | Hysterectomy among Finnish women: Prevalence and women's own opinions |
| 1992 | 20 | 4 | 213-216 | Elisabeth Arborelius, Toomas Timpka and James M. Nyce | Patients comment on video-recorded consultations - the "good" GP and the "bad" |
| 1992 | 20 | 4 | 217-225 | Niels Lynöe | Ethical and professional aspects of the practice of alternative medicine |
| 1992 | 20 | 4 | 226-233 | Karin M. Stjernqvist | Extremely low birth weight infants less than 901 g. Impact on the family during the first year |
| 1992 | 20 | 4 | 234-239 | Agneta Isacsson, Bertil S. Hanson, Lars Janzon and Gunnel Kugelberg | The epidemiology of sick leave in an urban population in Malmö, Sweden |
| 1992 | 20 | 4 | 240-246 | Lena Kanström, Lars-Erik Holm and Bo J. A. Haglund | Creating partnership with the food industry in the Stockholm cancer prevention program |
| 1992 | 20 | 4 | 247-252 | Jesper L. Boldsen, Bernard Jeune and Paul C. Madsen | Aspects of comfort and safety of condom: A study of two thousand intercourses among volunteer couples |
| 1992 | 20 | 4 | 253 | Jens Lauritsen | Reviewed Work: Searching for Causes of Work-Related Diseases. An introduction to Epidemiology at the Work Site by J Olsen, F Merletti, D Snashall, K Vuylsteek |
| 1993 | 21 | 1 | 1-2 | L. R. Karhausen | Incidence Density is no Density |
| 1993 | 21 | 1 | 3-9 | Ulf Lindblad, Lennart Råstam, Jonas Ranstam and Magnus Peterson | Validity of register data on acute myocardial infarction and acute stroke: The Skaraborg Hypertension Project |
| 1993 | 21 | 1 | 10-16 | Anders Ericson, Margareta Eriksson, Bengt Källén and Rolf Zetterström | Secular Trends in the Effect of Socio-Economic Factors on Birth Weight and Infant Survival in Sweden |
| 1993 | 21 | 1 | 17-23 | Sven Ove Samuelsen, Anne Inger Borge, Per Magnus and Leiv S. Bakketeig | Temporal and regional trends in fatal childhood injuries in Norway 1971-1989 |
| 1993 | 21 | 1 | 24-30 | Matti Isohanni, Hannu Oja, Irma Moilanen, Paula Rantakallio and Markku Koiranen | The relation between teenage smoking and drinking, with special reference to non-standard family background |
| 1993 | 21 | 1 | 31-39 | Marlene Stenbacka, Peter Allebeck and Anders Romelsjö | Initiation into drug abuse: The pathway from being offered drugs to trying cannabis and progression to intravenous drug abuse |
| 1993 | 21 | 1 | 40-50 | Knut Fylkesnes | Determinants of Health Care Utilization - Visits and Referrals |
| 1993 | 21 | 1 | 51-58 | Antonis D. Koutis, Åke Isacsson, Christos D. Lionis, Lars H. Lindholm, Karin Svenninger and Michael Fioretos | Differences in the diagnose panorama in primary health care in Dalby, Sweden and Spili, Crete |
| 1993 | 21 | 1 | 59 | Jørn Olsen | Reviewed Work: Toxic Oil Syndrome. Current knowledge and future perspectives. WHO Regional Publications European Series No. 42 by |
| 1993 | 21 | 1 | 59-60 | Kim Overvad | Reviewed Work: Death rates for malignant neoplasms for selected sites by sex and five-year age group in 33 countries. 1953-57 to 1983-87 by K. Aoki, N. Hayakawa, M. Kurihara, S. Suzuki |
| 1993 | 21 | 1 | 60-61 | Jan Mainz | Review: [Untitled]  Reviewed Work: Choices in Health Care. Zoetermeer, The Netherlands, Ministry of Welfare, Health and Cultural Affairs by AJ. Dunning |
| 1993 | 21 | 2 | 63-65 | Milton I. Roemer | The Meanings of Social Medicine and Public Health |
| 1993 | 21 | 2 | 66-68 | Ruth Riise | Nordic Registers of Visually Impaired Children |
| 1993 | 21 | 2 | 69-76 | Anders Ericson, Margareta Eriksson, Bengt Källén and Rolf Zetterström | Methods for the Evaluation of Social Effects on Birth Weight – Experiences with Swedish Population Registries |
| 1993 | 21 | 2 | 77-82 | Gunnel Boström, Johan Hallqvist, Bo J. A. Haglund, Anders Romelsjö, Leif Svanström and Finn Diderichsen | Socioeconomic Differences in Smoking in an Urban Swedish Population: The bias introduced by non-participation in a mailed questionnaire |
| 1993 | 21 | 2 | 83-89 | Agneta Österling, Mats Berglund, Lars-Håkan Nilsson and Hans Kristenson | Sex Differences in Response Style to Two Self-report Screening Tests on Alcoholism |
| 1993 | 21 | 2 | 90-97 | Jørn Olsen and Grethe Frische | Social Differences in Reproductive Health: A Study on Birth Weight, Stillbirths and Congenital Malformations in Denmark |
| 1993 | 21 | 2 | 98-106 | Seppo Olkkonen, Uolevi Lahdenranta, Pär Slätis and Risto Honkanen | Bicycle accidents often cause disability – An analysis of medical and social consequences of nonfatal bicycle accidents |
| 1993 | 21 | 2 | 107-115 | Seppo Miilunpalo | Measurement of problem-oriented need for The Primary Health Care Services: Need for exercise counseling as an example |
| 1993 | 21 | 2 | 116-119 | Dag Bruusgaard, Åsa Rytter Evensen and Tor Bjerkedal | Fibromyalgia - a new cause for disability pension |
| 1993 | 21 | 2 | 120-121 | Hogne Sandvik and Steinar Hunskaar | Incontinence pads – prevalence of use and individual consumption |
| 1993 | 21 | 2 | 122-125 | Johan Lökk, Bengt Arnetz and Töres Theorell | Physiological effects on patients following temporary closing of a geriatric day care unit |
| 1993 | 21 | 2 | 126-128 | Ida Hydle | Abuse and Neglect of The Elderly – a Nordic Perspective Report from a Nordic Research Project |
| 1993 | 21 | 2 | 129-134 | Frode Thuen and John Gunnar Mæland | Accident Prevention Activities in the Norwegian Municipalities: The Local Response to a Nationwide Campaign |
| 1993 | 21 | 2 | 135-140 | Merete Osler, Elisabeth Tornberg Hansen and Elisabeth Tornberg-Hansen | Dietary knowledge and behaviour among schoolchildren in Copenhagen, Denmark |
| 1993 | 21 | 2 | 141-142 | Holger Ursin | comment to the article: Physiological effects on patients following temporary closing of a geriatric day care unit: Scand J Soc Med 1993; 21: 122-125 |
| 1993 | 21 | 3 | 143-145 | Elisabeth Backe-Hansen | Children at Risk – A Presenation of a Nordic Project |
| 1993 | 21 | 3 | 146-149 | Lennart Köhler | Children with and without disabilities in the Nordic countries. A Nordic Project |
| 1993 | 21 | 3 | 150-158 | Pēteris Zvidriņš and Juris Krūmiņš | Morbidity and Mortality in Estonia, Latvia and Lithuania in the 1980's |
| 1993 | 21 | 3 | 159-163 | Myra Lewinter, Selin S. Kesmez and Kerim Gezgin | Self-reported health and function status of elderly Turkish immigrants in Copenhagen, Denmark |
| 1993 | 21 | 3 | 164-170 | Jan Olof Hörnquist, Margareta Zar and Birgit Hansson | Precursors of repeated short-term sick-leave: an empirical review of some background, job and well-being characteristics |
| 1993 | 21 | 3 | 171-175 | Yannis Alamanos, Kiki Tsamandouraki, Antonis Koutis and Michael Fioretos | Working at the loom and musculoskeletal disorders in a female population of Crete, Greece |
| 1993 | 21 | 3 | 176-187 | Ragnar Westerling | Indicators of "Avoidable" Mortality in Health Administrative Areas in Sweden 1974-1985 |
| 1993 | 21 | 3 | 188-196 | J. Sakari Karvonen, Arja H. Rimpelä and Matti Rimpelä | Modernization and Smoking — Regional Differences in Adolescent Smoking in Finland in 1977-1987 |
| 1993 | 21 | 3 | 197-202 | Jørn Olsen | Predictors of Smoking Cessation in Pregnancy |
| 1993 | 21 | 3 | 203-210 | Marianne E. Lindell, Henny M. Olsson and Henny M. Olsen | Students Opinions about Sex Education in a Swedish School Region |
| 1993 | 21 | 3 | 211-219 | Per Fink, Jørgen Jensen and Carsten Stig Poulsen | A study of hospital admissions over time, using longitudinal latent structure analysis |
| 1993 | 21 | 3 | 220-222 | Per Holm | "Project Lifestyle" and new forms of living for the mentally retarded in the local community |
| 1993 | 21 | 4 | 223-226 | Anne Helset | Elderly Women in the Nordic Countries; Level of Living and Situation in Life |
| 1993 | 21 | 4 | 227-232 | Sander Greenland | Summarization, smoothing, and inference in epidemiologic analysis |
| 1993 | 21 | 4 | 233-246 | Kirsten Avlund, Svend Kreiner and Kirsten Schultz-Larsen | Construct validation and the Rasch model: Functional ability of healthy elderly people |
| 1993 | 21 | 4 | 247-255 | Margareta Grafström, Astrid Nordberg and Bengt Winblad | Abuse is in the eye of the beholder: Reports by family members about abuse of demented persons in home care: A total population-based study |
| 1993 | 21 | 4 | 256-263 | Anna-Lisa Melin and Lars Olov Bygren | Perceived functional health of frail elderly in a primary home care programme and correlation of self-perception with objective measurements |
| 1993 | 21 | 4 | 264-271 | Olli-Pekka Ryynänen, Sirkka-Liisa Kivelä, Risto Honkanen, Pekka Laippala and Veijo Saano | Medications and Chronic Diseases as Risk Factors for Falling Injuries in the Elderly |
| 1993 | 21 | 4 | 272-280 | Karin Styrborn and Mats Thorslund | Delayed discharge of elderly hospital patients — A study of bed-blockers in a health care district in Sweden |
| 1993 | 21 | 4 | 281-285 | Eleni Petridou, C-Chung Hsieh, Yannis Skalkidis, Nektaria Toupadaki and Yannis Athanassopoulos | Suggestion of concomitant changes of electric power consumption and childhood leukemia in Greece |
| 1994 | 22 | 1 | 1-6 | W. W. Holland | Changing Names |
| 1994 | 22 | 1 | 7-14 | Agneta Grimby and Ingela Wiklund | Health-Related Quality of Life in Old Age. A Study among 76-Year-Old Swedish Urban Citizens |
| 1994 | 22 | 1 | 15-19 | Karin Holmén, Kjerstin Ericsson and Bengt Winblad | Loneliness and living conditions of the oldest old |
| 1994 | 22 | 1 | 20-26 | Margareta Bondestam and Finn Rasmussen | Preschool children's absenteeism from Swedish municipal day-care centres because of illness in 1977 and 1990. Geographical variations and characteristics of the day-care centres |
| 1994 | 22 | 1 | 27-34 | Kristina Alexanderson, Margareta Leijon, Ingemar Åkerlind, Hillevi Rydh and Per Bjurulf | Epidemiology of sickness absence in a Swedish county in 1985, 1986 and 1987: A Three Year Longitudinal Study with Focus on Gender, Age and Occupation |
| 1994 | 22 | 1 | 35-40 | Fernando C. Barros, Birger C. Forsberg, César G. Victora, Ana G. K. Maranhäo, Marijke Stegeman, Alejandro Gonzalez-Richmond, Reynaldo M. Martins, Zilda A. Neumann, Jay McAuliffe and João A. Branco Jr. | Quality assurance of a diarrhoea control programme in northeastern Brazil |
| 1994 | 22 | 1 | 41-45 | Ole Böstman | Economic Considerations on Avoiding Implant Removals after Fracture Fixation by Using Absorbable Devices |
| 1994 | 22 | 1 | 46-49 | Östen Helgesson, Calle Bengtsson, Leif Lapidus, Christer Merck and Pär Sparén | Malignant Disease Observed in a Cohort of Women: A validation of Swedish Cancer Registry data |
| 1994 | 22 | 1 | 50-57 | Noritoshi Tanida | Japanese Attitudes towards Truth Disclosure in Cance |
| 1994 | 22 | 1 | 58-67 | Lars G. Dahlgren and Anita I. M. Sandström | Norm Systems in Transitions: Changes in Health-Related Values among Male Smelters at an Industrial Plant in Northern Sweden |
| 1994 | 22 | 1 | 68-73 | Finn Egil Skjeldestad | When Pregnant - Why Induced Abortion? |
| 1994 | 22 | 1 | 74-80 | Ipatia Apostolidou, Klea Katsouyanni, Giota Touloumi, Nikolaos Kalpoyannis, Andreas Constantopoulos and Dimitrios Trichopoulos | Seasonal variation of neonatal and infant deaths by cause in Greece |
| 1994 | 22 | 2 | 81-85 | Søren Holm | Moral Reasoning in Biomedical Research Protocols |
| 1994 | 22 | 2 | 86-89 | Tomas Faresjö, Kurt Svärdsudd and Gösta Tibblin | Social Mobility and Health in a Prospective Study of Middle-Aged Men |
| 1994 | 22 | 2 | 90-96 | Henrik Östberg and Sven-Mårten Samuelsson | Occupational Retirement in Women due to Age: Health Aspects |
| 1994 | 22 | 2 | 97-106 | Preben Bendtsen and Jan Olof Hörnquist | Rheumatoid arthritis, coping and well-being: Cross-sectional sub-group comparisons and correlational analyses |
| 1994 | 22 | 2 | 107-112 | Niina Jaakkola, Risto Ruotsalainen and Jouni J. K. Jaakkola | What Are the Determinants of Children's Exposure to Environmental Tobacco Smoke at Home? |
| 1994 | 22 | 2 | 113-119 | Sven Bremberg and Elisabeth Arborelius | Effects on adolescent alcohol consumption of a school based student-centred health counselling programme |
| 1994 | 22 | 2 | 120-126 | Anne Hammarström and Urban Janlert | Epidemiology of School Injuries in the Northern Part of Sweden |
| 1994 | 22 | 2 | 127-131 | Eleni Petridou, Charalambos Proukakis, Donald Tong, Dimitrios Kassimos, Fani Athanassiadou-Piperopoulou, Stavros Haidas, Maria Kalmanti, Dimitrios Koliouskas, Helen Kosmidis, Annie Louizi, Simos Simopoulos and Dimitrios Trichopoulos | Trends and Geographical Distribution of Childhood Leukemia in Greece in Relation to the Chernobyl Accident |
| 1994 | 22 | 2 | 132-138 | Ilmo Keskimäki, Seppo Aro and Juha Teperi | Regional variation in surgical procedure rates in Finland |
| 1994 | 22 | 2 | 139-144 | Johanne Sundby, Anne Olsen and Berit Schei | Quality of care for infertility patients. An evaluation of a plan for a hospital investigation |
| 1994 | 22 | 2 | 145-158 | B.I.B. Lindahl and Lars Age Johansson | Multiple cause-of-death data as a tool for detecting artificial trends in the underlying cause statistics: a methodological study |
| 1994 | 22 | 2 | 159-160 | David Lester | The variation of suicide rates over time in Scandinavia |
| 1994 | 22 | 3 | 161-165 | Lars Cernerud | Are there still social inequalities in height and body mass index of Stockholm children? |
| 1994 | 22 | 3 | 166-169 | Airi Värnik, Danuta Wasserman and Gunnar Eklund | Suicides in the Baltic Countries, 1968-90 |
| 1994 | 22 | 3 | 170-177 | D Wasserman, M Fellman, U Bille-Brahe, T Bjerke, L Jacobsson, G Jessen, JK Lönnqvist, O Njåstad, A Ostamo and E Salander-Renberg | Parasuicide in the Nordic Countries |
| 1994 | 22 | 3 | 178-186 | Carl Göran Svedin, Kristina Back and Marie Wadsby | Mental Health Among Immigrant and Refugee Children of Divorced Parents |
| 1994 | 22 | 3 | 187-193 | Lena Edén, Göran Ejlertsson, Berndt Lamberger, Ido Leden, Bertil Nordbeck and Pär Sundgren | Immigration and Socio-economy as Predictors of Early Retirement Pensions |
| 1994 | 22 | 3 | 194-200 | Maymuna M. Omar, Ulf Högberg and Brita Bergström | Fertility, Infertility and Child Survival of Somali Women |
| 1994 | 22 | 3 | 201-203 | Henrietta P. Uniken Venema and Henk F.L. Garretsen | Health and health care use of Turkish and Dutch inhabitants of the Netherlands |
| 1994 | 22 | 3 | 204-208 | Mads Melbye and Robert J. Biggar | A Profile of HIV-Risk Behaviours Among Travellers — A Population Based Study of Danes Visiting Greenland |
| 1994 | 22 | 3 | 209-212 | Anne Eskild and Georg Petersen | Cigarette Smoking and Drinking of Alcohol are not Associated with Rapid Progression to Acquired Immunodeficiency Syndrome among Homosexual Men in Norway |
| 1994 | 22 | 3 | 213-218 | Jørn Olsen | The Association Between Birth Weight, Placenta Weight, Pregnancy Duration, Subfecundity, and Child Development |
| 1994 | 22 | 3 | 219-224 | Sjurður F. Olsen and Jørn Olsen | A birth weight adjusted comparison of perinatal mortality in the Faroe Islands and Denmark |
| 1994 | 22 | 3 | 225-227 | Epidemiological Analysis of Serious Occupational Accidents in Southern Finland | Epidemiological Analysis of Serious Occupational Accidents in Southern Finland |
| 1994 | 22 | 3 | 228-234 | Åke Thörn | Case Report on a Sick Building: Analysis and Interpretation in the Context of Its Disease History |
| 1994 | 22 | 3 | 235-236 | J. J. Boddewyn | The Effects of Tobacco Sales Promotion on Initiation of Smoking |
| 1994 | 22 | 3 | 237 | Hans Wedel | Reviewed Work: Teaching Epidemiology: What you should know and what you can do Oxford, UK by J Olsen, D Trichopoulos |
| 1994 | 22 | 3 | 238-239 | Henrik Sælan | Reviewed Work: Health Issues Related to Alcohol Consumption by PM Verschuren |
| 1994 | 22 | 4 |  | Jørn Olsen and Anders Foldspang | Johannes Ipsen 20 May 1911 - 19 May 1994 |
| 1994 | 22 | 4 | 242-248 | Baizhuang Xu, Arja Rimpelä, Marjo-Riitta Järvelin and Mauri Nieminen | Sex Differences of Infant and Child Mortality in China |
| 1994 | 22 | 4 | 249-255 | Håkan Elmén | Death Rates and Causes of Death among Children and Youth in Göteborg, Sweden 1971-85: Indicators for Public Health Work in a City |
| 1994 | 22 | 4 | 256-263 | Birger Gran | Population Based CVD Health Risk Appraisal: A method to create a "critical mass" of health-conscious people |
| 1994 | 22 | 4 | 264-272 | Lars-Göran Persson, Kjell Lindström, Hans Lingfors and Calle Bengtsson | A study of men aged 33-42 in Habo, Sweden with special reference to cardiovascular risk factors: Design, health profile and characteristics of participants and non-participants |
| 1994 | 22 | 4 | 273-282 | Randi Selmer and Aage Tverdal | Mortality from stroke, coronary heart disease and all causes related to blood pressure and length of follow-up |
| 1994 | 22 | 4 | 283-292 | Olga Augustsson, Bo G. Eriksson, Ulf Rosenhall, Elisabet Rothenberg, Bertil Wärne and Bertil Steen | The Johanneberg study – a sociomedical survey in an urban elderly population: I. General presentation of the study including an analysis of non-response and identification of risk groups |
| 1994 | 22 | 4 | 292-298 | Grethe Johansen | An Economic Appraisal of Two Strategies in Geriatric Screening |
| 1994 | 22 | 4 | 299-302 | Anna-Lena Andersson, Lars-Olof Dahlbäck and Peter Allebeck | Psychosocial consequences of traffic accidents: a two year follow-up |
| 1994 | 22 | 4 | 303-308 | Jan Lundbom, Hans Olav Myhre, Brynjulf Ystgaard, Svend Aakhus, Arve Tromsdal, Randi Sudbø, Bjørnar Klykken, Tore Salvesen, Gunnar Rongved, Torstein Holm Morstøl, Audun Heskestad, Randi Hammervold and Olaf W. Levang | Exercise Tolerance and Work Ability Following Aorto-coronary Bypass Surgery |
| 1994 | 22 | 4 | 309-314 | Anne Eskild, Per Magnus, Christian Sohlberg, Finn Jensen and Peter Kittelsen | A Comparison of the Progression Rate to Acquired Immunodeficiency Syndrome between Intravenous Drug Users and Homosexual Men |
| 1994 | 22 | 4 | 315-317 | Zoran Radovanovic, Ljubomir Eric and Viktorija Cucic | Institute "Jakes" - an Experiment which Ceased to Exist |
| 1995 | 23 | 1 | 1-2 | Lars Olov Bygren, Anders Foldspang and John Gunnar Mœland | Scandinavian Journal of Social Medicine: Continuation and Change |
| 1995 | 23 | 1 | 3-8 | Peter M. Nilsson, Lars Møller and Per-Olof Östergren | Social Class and Cardiovascular Disease – an Update |
| 1995 | 23 | 1 | 9-16 | Birger Gran | Major Differences in Cardiovascular Risk Indicators by Educational Status: Results from a population based screening program |
| 1995 | 23 | 1 | 17-22 | Agneta Isacsson, Bertil S. Hanson, Jonas Ranstam, Lennart Råstam and Sven-Olof Isacsson | Social Network, Social Support and the Prevalence of Neck and Low Back Pain after Retirement: A Population Study of Men Born in 1914 in Malmö, Sweden |
| 1995 | 23 | 1 | 23-27 | Lars Cernerud and Jörn Elfving | Social Inequality in Height: A Comparison between 10-Year-Old Helsinki and Stockholm Children |
| 1995 | 23 | 1 | 28-31 | Lars Cernerud | Height and Social Mobility: A Study of the Height of 10 Year Olds in Relation to Socio-economic Background and Type of Formal Schooling |
| 1995 | 23 | 1 | 32-38 | Matti Isohanni, Hannu Oja, Irma Moilanen, Markku Koiranen and Paula Rantakallio | Smoking or Quitting during Pregnancy: Associations with Background and Future Social Factors |
| 1995 | 23 | 1 | 39-46 | Bo Burström, Bo JA Haglund, Per Tillgren, Lars Berg, Eva Wallin, Henrik Ullén and Christopher Smith | Health Promotion in Schools: Policies and Practices in Stockholm County, 1990 |
| 1995 | 23 | 1 | 47-52 | Eeva Ollila, Elina Hemminki and Kristiina Kajesalo | Physicians' Experience with Norplant ® Implantable Contraceptives in Finland |
| 1995 | 23 | 1 | 53-59 | Knut-Inge Klepp | Nonresponse Bias Due to Consent Procedures in School-Based, Health-Related Research |
| 1995 | 23 | 1 | 60-65 | A.H. Rimpelä, B. Savonius, M. K. Rimpelä and T. Haahtela | Asthma and Allergic Rhinitis among Finnish Adolescents in 1977-1991 |
| 1995 | 23 | 1 | 66-74 | Anneli Helminen, Pirjo Halonen, Tuomo Rankinen, Aulikki Nissinen and Rainer Rauramaa | Validity Assessment of a Social Support Index |
| 1995 | 23 | 1 | 75-80 | Mika Gissler, Juha Teperi, Elina Hemminki and Jouni Meriläinen | Data Quality after Restructuring a National Medical Registry |
| 1995 | 23 | 2 | 81-84 | Tore Nilstun and Rolf Ohlsson | Should Health Care be Rationed by Age? |
| 1995 | 23 | 2 | 85-87 | Vinjar Fønnebø | The Tromsø Study: Does Husband's Religion Influence the Lifestyle of Seventh-Day Adventist Women? |
| 1995 | 23 | 2 | 88-94 | Saija Laitinen, Leena Räsänen, Jorma Viikari and Hans K. Åkerblom | Diet of Finnish Children in Relation to the Family's Socio-Economic Status |
| 1995 | 23 | 2 | 95-102 | P.-O. Östergren, E. Lindbladh, S.-O. Isacsson, H. Odeberg and S.-E. Svensson | Social Network, Social Support and the Concept of Control — a Qualitative Study Concerning the Validity of Certain Stressor Measures Used in Quantitative Social Epidemiology |
| 1995 | 23 | 2 | 103-109 | Inger Stensvold and Aage Tverda | The Relationship of Coffee Consumption to Various Self-Reported Cardiovascular Events in Middle-Aged Norwegian Men and Women |
| 1995 | 23 | 2 | 110-120 | A. Jeanne M. van Loon, Johannes Burg, R. Alexandra Goldbohm and Piet A. van den Brandt | Differences in Cancer Incidence and Mortality Among Socio-Economic Groups |
| 1995 | 23 | 2 | 121-128 | Tore J. Larsson and Ulf Björnstig | Persistent Medical Problems and Permanent Impairment Five Years after Occupational Injury |
| 1995 | 23 | 2 | 129-136 | Anna-Maija Pietilä, Paula Rantakallio and Esa Läärä | Background Factors Predicting Non-Response in a Health Survey of Northern Finnish Young Men |
| 1995 | 23 | 2 | 137-140 | Matti Isohanni, Ilkka Winblad and Pentti Nieminen | Quantitative Assessment of Life Span of the Aged Persons — A Preliminary Study |
| 1995 | 23 | 2 | 141-142 | Niels Lynöe and Mikael Sandlund | Primary Drop-out: An Estimation of Size |
| 1995 | 23 | 2 | 143 | Daniel Andersen | Reviewed Work: Physician-assisted death by JM Humber, RF Almeder, GA Kasting |
| 1995 | 23 | 2 | 144 | Jan Mainz | Reviewed Work: Health care and Medical Priorities Commission. No Easy Choices. The Difficult Priorities of Health Care by |
| 1995 | 23 | 3 | 145-149 | Victor W. Sidel and George A. Silve | Social Medicine: A 1990's perspective from the United States |
| 1995 | 23 | 3 | 150-155 | Lars B. Larsen, Thomas K. Poulsen and Hans G. Johannsen | The age-dependent incidence of injuries due to road traffic accidents in Odense, Denmark from 1980 to 1992 |
| 1995 | 23 | 3 | 156-161 | Jørn Olsen and Pitchaya Tuntiseranee | Is moderate alcohol intake in pregnancy associated with the craniofacial features related to the fetal alcohol syndrome? |
| 1995 | 23 | 3 | 162-166 | Jørn Olsen | Moderate alcohol consumption in pregnancy and subsequent left-handedness: A follow-up study |
| 1995 | 23 | 3 | 167-172 | Anneli Helminen, Tuomo Rankinen, Michele Mercuri and Rainer Rauramaa | Carotid atherosclerosis in middle-aged men: Relation to conjugal circumstances and social support |
| 1995 | 23 | 3 | 173-178 | Willy Pedersen and Henrik Aas | Sexual victimization in Norwegian children and adolescents: victims, offenders, assaults |
| 1995 | 23 | 3 | 179-188 | Siri Næss, Siri Naess, Kristian Midthjell, Torbjøm Moum, Tom Sørensen and Kristian Tambs | Diabetes mellitus and psychological well-being. Results of the Nord-Trøndelag health survey |
| 1995 | 23 | 3 | 189-192 | Elle Laursen and Lis Larsen | Socio-economic status of AIDS patients |
| 1995 | 23 | 3 | 193-201 | Per Tillgren, Bo J. A. Haglund, Tuula Ainetdin and Lars-Erik Holm | Who is a successful quitter? One-year follow-up of a National Tobacco Quit and Win Contest in Sweden |
| 1995 | 23 | 3 | 202-208 | Jostein Rise and Reidar Jakobsen | Organization of HIV/AIDS related attitudes and predictors of attitudes |
| 1995 | 23 | 3 | 209-215 | Mårten Lagergren | Determining the appropriate level of care: An analysis of factors affecting the staff's overall needs assessments, using data collected through the ASIM monitoring system |
| 1995 | 23 | 3 | 216-219 | Niels Lynöe and Mikael Sandlund | Ethical and educational aspects of clinical training: A study in the experience and attitudes of medical students |
| 1995 | 23 | 3 | 220-224 | Margareta Falkeborn, Ingemar Persson, Tord Naessén and Ulf Kressner | Validity of information on gynecological operations in the Swedish in-patient registry |
| 1995 | 23 | 4 | 225-226 | Elina Hemminki | The future of population strategies in prevention: drugs for all? |
| 1995 | 23 | 4 | 227-232 | Jan Marcus Sverre | A comparative study of trends in mortality rates of the ageing population in Norway, Sweden, Denmark, and Finland, 1966-1986 |
| 1995 | 23 | 4 | 233-235 | Jørn Olsen | Is left-handedness a sensitive marker of prenatal exposures or indicators of fetal growth? |
| 1995 | 23 | 4 | 236-241 | Elise A-L Kosunen, Arja H. Rimpelä and Matti K. Rimpelä | Sixteen-year-old oral contraceptive users in Finland, 1981-1993 |
| 1995 | 23 | 4 | 242-250 | Kåre-S. Tveit, Allan Nyfors, Arvid Nilsen and Reidar Jacobsen | Knowledge of HIV infection and the fear of mixing socially with HIV-infected and AIDS-sick patients among STD patients in Bergen, Norway |
| 1995 | 23 | 4 | 251-257 | Sven-Olof Andersson, Bengt Mattsson and Niels Lynöe | Patients frequently consulting general practitioners at a primary health care centre in Sweden — A comparative study |
| 1995 | 23 | 4 | 258-264 | Harald R. Lie, Bengt Lagerkvist, Finn Rasmussen, Johan Hagelsteen, Marie-Christine Börjeson, Jan Lagergren and Lennart Köhler | Nordic children with myelomeningocele: the utilization of and satisfaction with health care and medical services |
| 1995 | 23 | 4 | 265-272 | Nelson Blank and Finn Diderichsen | Short-term and long-term sick-leave in Sweden: relationships with social circumstances, working conditions and gender |
| 1995 | 23 | 4 | 273-285 | Karin Styrborn | Early discharge planning for elderly patients in acute hospitals — an intervention study |
| 1996 | 24 | 1 | 2-7 | Allan Krasnik | The concept of equity in health services research |
| 1996 | 24 | 1 | 8-13 | L. R. Karhausen | The logic of causation in epidemiology |
| 1996 | 24 | 1 | 14-26 | Eleni Petridou, Mary Koussouri, Nektaria Toupadaki, Antigoni Papavassiliou, Sotiris Youroukos, Effi Katsarou and Dimitrios Trichopoulos | Risk factors for cerebral palsy: a case-control study in Greece |
| 1996 | 24 | 1 | 27-35 | Ingemar Åkerlind, Kristina Alexanderson, Gunnel Hensing, Margareta Leijon and Per Bjurulf | Sex differences in sickness absence in relation to parental status |
| 1996 | 24 | 1 | 36-42 | Annett Arntzen, Torbjørn Moum, Per Magnus and Leiv S. Bakketeig | Marital status as a risk factor for fetal and infant mortality |
| 1996 | 24 | 1 | 43-49 | Björn Söderfeldt, Marie Söderfeldt, Carl-Göran Ohlson and Lars-Erik Warg | Psychosomatic symptoms in human service work: A Study on Swedish Social Workers and Social Insurance Personnel |
| 1996 | 24 | 1 | 50-54 | Kitty Strand, Ebba Wergeland and Tor Bjerkedal | Fertility patterns according to occupational grouping in Norway, 1989 |
| 1996 | 24 | 1 | 55-61 | Marie-Louise Nordström and Sven Cnattingius | Effects on birthweights of maternal education, socio-economic status, and work-related characteristics |
| 1996 | 24 | 1 | 62-66 | Hjalmar Freysteinsson and Johann A. Sigurdsson | Breast-feeding in Iceland: Predictive factors and effects of interventive measures |
| 1996 | 24 | 1 | 67-76 | Jan Qvist, Sven-Erik Johansson and Leena Maria Johansson | Multivariate analyses of mortality from coronary heart disease due to biological and behavioural factors |
| 1996 | 24 | 1 | 77-78 | László Márk, György Simondán and András Kondacs | Cardiovascular risk differences among employed and unemployed village people in Hungary |
| 1996 | 24 | 2 | 81-89 | Nelson Blank and Finn Diderichsen | Social inequalities in the experience of illness in Sweden: a "double suffering" |
| 1996 | 24 | 2 | 90-101 | Birgit Ljungquist and Gerdt Sundström | Health and social networks as predictors of survival in old age |
| 1996 | 24 | 2 | 102-106 | R. Más, V. Escribà and C. Colomer | Who quits smoking during pregnancy? |
| 1996 | 24 | 2 | 107-113 | Torbjörn Messner and Bo Petersson | Alcohol consumption and Ischemic Heart Disease mortality in Sweden |
| 1996 | 24 | 2 | 114-120 | Bo A. Rix and Elsebeth Lynge | Cancer incidence in Danish health care workers |
| 1996 | 24 | 2 | 121-123 | Lea Käsmä-Ronkainen and Hannu Virokannas | Concern about the environment among medical students |
| 1996 | 24 | 2 | 124-131 | Gisela Rose and Calle Bengtsson | Effects of a health examination programme on quality of life and subjective well-being |
| 1996 | 24 | 2 | 132-139 | Veikko Viitasalo, Aulikki Nissinen, Paula Kivinen and Jorma Takala | The last year of life: mortality, cause of death and need for help among old men |
| 1996 | 24 | 2 | 140-144 | Maja-Lisa Løchen and Knut Rasmussen | Palpitations and lifestyle: impact of depression and self-rated health. The Nordland Health Study |
| 1996 | 24 | 3 | 145-149 | A. Weber, H. Strebl and G. Lehnert | Development and status of social medicine in Germany |
| 1996 | 24 | 3 | 150-151 | Leo Uzych | Social medicine and the United States |
| 1996 | 24 | 3 | 151 | Victor W. Sidel and Goerge Silver | Reply: Social Medicine: A 1990s Perspective from the United States |
| 1996 | 24 | 3 | 152-154 | Elina Hemminki and Sinikka Sihvo | Knowledge of prenatal care among non-pregnant women: an explanation for early attendance |
| 1996 | 24 | 3 | 155-156 | Zhou Weijin and Jørn Olsen | Is foetal growth reduction induced by smoking modified by body mass or gestational weight gain? |
| 1996 | 24 | 3 | 157-160 | G. Steneroth, G. Streneroth, M. Eriksson, B. Jonsson, L. Billing and R. Zetterström | Support to drug-addicted parents: a help to the children? |
| 1996 | 24 | 3 | 161-168 | Sven Ingmar Andersson | Appraisal, coping, motivational factors and gender in vocational rehabilitation |
| 1996 | 24 | 3 | 169-176 | Kristina Alexanderson, Kristina Alexandersson, Adam Sydsjö, Gunnel Hensing, Gunilla Sydsjö and John Carstensen | Impact of pregnancy on gender differences in sickness absence |
| 1996 | 24 | 3 | 177-184 | Madhavi Bajekal, Sundquist Jan and Brian Jarman | The Swedish UPA score: An administrative tool for identification of underprivileged areas |
| 1996 | 24 | 3 | 185-192 | Lisa Rönnberg, Lars Welin and Bengt Winblad | Mental impairment and utilization of community services: a study of the elderly in a parish of Stockholm |
| 1996 | 24 | 3 | 193-198 | Preben Bendtsen and Jan Olof Hörnquist | Disease course and prognosis in rheumatoid arthritis: An interdisciplinary prospective longitudinal study |
| 1996 | 24 | 3 | 199-205 | Jan Marcus Sverre and Petter Laake | Trends in diagnosis-specific mortality for the aging population in Norway, 1966–1986 |
| 1996 | 24 | 3 | 206-217 | Kirsten Avlund, Pertti Era, Michael Davidsen and Ingrid Gause-Nilsson | Item bias in self-reported functional ability among 75-year-old men and women in three Nordic localities |
| 1996 | 24 | 3 | 218-224 | Olle Lundberg and Kristiina Manderbacka | Assessing reliability of a measure of self-rated health |
| 1996 | 24 | 4 | 225-226 | Milton I. Roemer | Health care regulation in the United States |
| 1996 | 24 | 4 | 227-236 | Marie Wadsby, Gunilla Sydsjö and Carl Göran Svedin | Children of Psychosocial Risk-mothers: life events, social interaction, and behaviour problems at the age of 8 years |
| 1996 | 24 | 4 | 237-242 | Roland Rosmond, Leif Lapidus and Per Björntorp | A comparative review of psychosocial and occupational environment in native Swedes and immigrants |
| 1996 | 24 | 4 | 243-252 | Katarina Hjelm, Åke Isacsson, Jan Apelqvist, Jan Sundquist and Per Nyberg | Foreign- and Swedish-born diabetic patients – a population-based study of prevalence, glycaemic control and social position |
| 1996 | 24 | 4 | 253.258 | Lars Gunnar Hörte, Richard Stensman and UllaBritt Sundqvist-Stensman | Physical disease among 21 suicide cases: Interviews of relatives and friends |
| 1996 | 24 | 4 | 259-263 | Emmanuel Agapitos, Franco Mollo, Lorenzo Tomatis, Klea Katsouyanni, Loren Lipworth, Luisa Delsedime, Anna Kalandidi, Anna Karakatsani, Elio Riboli, Rodolfo Saracci and Dimitrios Trichopoulos | Epithelial, possibly precancerous, lesions of the lung in relation to smoking, passive smoking, and socio-demographic variables |
| 1996 | 24 | 4 | 264-271 | Grethe E. Borchgrevink, Inggard Lereim, Linn Røyneland, Arild Bjørndal and Olav Haraldseth | National health insurance consumption and chronic symptoms following mild neck sprain injuries in car collisions |
| 1996 | 24 | 4 | 272-281 | Anna-Mari Aalto, Antti Uutela and Tero Kangas | Health behaviour, social integration, perceived health and dysfunction. A comparison between patients with type I and II diabetes and controls |
| 1996 | 24 | 4 | 282-292 | Kent S. Lindqvist, Toomas Timpka and Per Bjurulf | Injuries during leisure physical activity in a Swedish municipality |
| 1996 | 24 | 4 | 293-301 | Peter Nyberg, Ulf Björnstig and L-O Bygren | Road characteristics and bicycle accidents |
| 1997 | 25 | 1 | 1-3 | Darryl Wieland | The art and science of targeting geriatrics programs |
| 1997 | 25 | 1 | 4-7 | Pertti Karppi and Reijo Tilvis | Outcome of aged patients in Finnish supervised home care |
| 1997 | 25 | 1 | 8-13 | Lars Nyberg, Yngve Gustafson, Anders Janson, Per-Olof Sandman and Sture Eriksson | Incidence of falls in three different types of geriatric care: A Swedish prospective study |
| 1997 | 25 | 1 | 14-16 | D. Kocijan-Hercigonja, Z. Knezovic, J. Grguric, R. Stuvland and B. Lagerkvist | Psychosocial support in war areas using Community-Based Rehabilitation Strategy |
| 1997 | 25 | 1 | 17-25 | Katarina Hamberg, Eva Johansson, Gerd Lindgren and Göran Westman | The impact of marital relationship on the rehabilitation process in a group of women with long-term musculoskeletal disorders |
| 1997 | 25 | 1 | 26-27 | Sirkku Vilkman, Timo Keistinen, Tuili Tuuponen and Sirkka-Liisa Kivelä | Population ageing will lead to an increase in hospitalisation for chronic illnesses like asthma and COPD |
| 1997 | 25 | 1 | 28-32 | Tomas Faresjö, Kurt Svärdsudd and Gösta Tibblin | The concept of status incongruence revisited: a 22-year follow-up of mortality for middle-aged men |
| 1997 | 25 | 1 | 33-38 | Marit B Veierød, Anne Eskild, Hein Stigum, Johannes Thorvaldsen and Per Magnus | Prevalence and trends in homosexual behaviour in Norway |
| 1997 | 25 | 1 | 39-43 | Per E Wändell, Bengt Brorsson and Hans Åberg | Psychic and socioeconomic consequences with diabetes compared to other chronic conditions |
| 1997 | 25 | 1 | 44-49 | Lone G. Nielsen, Morten Frisch and Mads Melbye | Cancer risk in a cohort of Danes working in Greenland |
| 1997 | 25 | 1 | 50-57 | Sören Brage, Tor Bjerkedal and Dag Bruusgaard | Occupation-specific morbidity of musculoskeletal disease in Norway |
| 1997 | 25 | 1 | 58-63 | Gudrún Kristjánsdóttir | Prevalence of pain combinations and overall pain: a study of headache, stomach pain and back pain among school-children |
| 1997 | 25 | 2 | 65-66 | Milton I. Roemer | Social Insurance for Health Service |
| 1997 | 25 | 2 | 67-69 | Inge-Bert Täljedal | Weak and strong holism |
| 1997 | 25 | 2 | 70-73 | Gösta Carlsson | Pitfalls in epidemiological analysis |
| 1997 | 25 | 2 | 74-82 | Signild Vallgårda | Why was the perinatal mortality rate higher in Denmark than in Sweden? The development in the 1970s and 1980s |
| 1997 | 25 | 2 | 83-92 | Ossi Rahkonen, Sara Arber and Eero Lahelma | Health-related social mobility: a comparison of currently employed men and women in Britain and Finland |
| 1997 | 25 | 2 | 93-99 | Willy Eriksen, Leiv Sandvik and Dag Bruusgaard | Social support and the smoking behaviour of parents with preschool children |
| 1997 | 25 | 2 | 100-110 | Görel K. Näslund | Relationships between health behavior, knowledge, and beliefs among Swedish blue-collar workers |
| 1997 | 25 | 2 | 111-118 | Torbjörn Eriksson, Andreas Sonesson and Åke Isacsson | HIV/AIDS — Information and knowledge: a comparative study of Kenyan and Swedish teenagers |
| 1997 | 25 | 2 | 119-125 | Eleni Petridou, Themistoklis Karpathios, Nick Dessypris, Effi Simou and Dimitrios Trichopoulos | The role of dairy products and non alcoholic beverages in bone fractures among schoolage children |
| 1997 | 25 | 2 | 126-135 | Lars A. Lindholm, Måns E. Rosén and Magnus E. Stenbeck | Determinants of willingness to pay taxes for a community-based prevention programme |
| 1997 | 25 | 2 | 136-143 | Seppo Aro, Anja Noro and Marjo Salinto | Deinstitutionalization of the elderly in Finland, 1981-91 |
| 1997 | 25 | 3 | 145-148 | Erik Allander and B. I. B. Lindahl | Why is prevention so difficult and slow? |
| 1997 | 25 | 3 | 149-155 | Kristina Berg-Kelly, Bengt Alvén, Laslo Erdes, Torbjörn Erneholm, Ingemar Johannisson and Eva Mattsson-Elofson | Health habits and risk behavior among youth in three communities with different public health approach |
| 1997 | 25 | 3 | 156-167 | M. Aarnio, U.M. Kujala and J. Kaprio | Associations of health-related behaviors, school type and health status to physical activity patterns in 16 year old boys and girls |
| 1997 | 25 | 3 | 168-175 | Catharina Gåfvels and Folke Lithner | Lifestyle as regards physical exercise, smoking and drinking, of adult insulin-treated diabetic people compared with non-diabetic controls |
| 1997 | 25 | 3 | 176-179 | M. Morales-Suárez-Varela, F. Jaén-Martínez, A. Llopis-Gonzalez and B. Silla Sobrecases | Sociodemographic characteristics of female habitual benzodiazepine consumers in the catchment area of a health care centre |
| 1997 | 25 | 3 | 180-184 | Miroslaw Lurie, Marianne Gustafsson, Margareta Lindh and Harald Sanne | Incidence of long-term sick-listing in an urban area of Sweden and its relationship with demographic data of the population |
| 1997 | 25 | 3 | 185-192 | Gunnel Hensing, Fredrik Spak, Kristina Alexanderson and Peter Allebeck | Sick-leave among women and the role of psychiatric disorder |
| 1997 | 25 | 3 | 193-201 | Kitty Strand, Ebba Wergeland and Tor Bjerkedal | Work load, job control and risk of leaving work by sickness certification before delivery, Norway 1989 |
| 1997 | 25 | 3 | 202-206 | Neven Henigsberg, Bengt Lagerkvist, Zrinjka Matek and Ivica Kostovic | War victims in need of physical rehabilitation in Croatia |
| 1997 | 25 | 3 | 207-209 | Anders Hjern and Peter Allebeck | Health examinations and health services for asylum seekers in Sweden |
| 1997 | 25 | 3 | 210-216 | Niels Lynöe and Tomas Svensson | Doctors' attitudes towards empirical data—a comparative study |
| 1997 | 25 | 3 | 217-223 | Harmeet Sjögren, Ulf Björnstig and Anders Eriksson | Comparison between blood analysis and police assessment of drug and alcohol use by injured drivers |
| 1997 | 25 | 4 | 225-228 | Niels Lynöe | Medical ethics—an endeavour for social medicine? |
| 1997 | 25 | 4 | 229-237 | Toomas Timpka, Margareta Leijon, Göran Karlsson, Lilian Svensson and Per Bjurulf | Long-term economic effects of team-based clinical case management of patients with chronic minor disease and long-term absence from working life |
| 1997 | 25 | 4 | 238-242 | Tuili Tuuponen, Timo Keistinen and Sirkka-Liisa Kivelä | Regional differences in long-term mortality among hospital-treated asthma and COPD patients |
| 1997 | 25 | 4 | 243-245 | Lennart Nordenfelt | Holism reconsidered: a reply to Täljedahl |
| 1997 | 25 | 4 | 246 | In all fairness | In all fairness |
| 1997 | 25 | 4 | 247-248 | Lennart Nordenfelt | On holism and conceptual structures |
| 1997 | 25 | 4 | 249-257 | Bertil S. Hanson, Per-Olof Östergren, Sölve Elmståhl, Sven-Olof Isacsson and Jonas Ranstam | Reliability and validity assessments of measures of social networks, social support and control - results from the Malmö Shoulder and Neck Study |
| 1997 | 25 | 4 | 258-265 | Dejian Lai | Spatial statistical analysis of Chinese cancer mortality: a comparison study of the D statistic |
| 1997 | 25 | 4 | 266-270 | Anne Hammarström and Urban Janlert | Unemployment and sexual risk-taking among adolescents |
| 1997 | 25 | 4 | 271-279 | David R. Ragland, Birgit A. Greiner, Barbara L. Holman and June M. Fisher | Hypertension and years of driving in transit vehicle operators |
| 1997 | 25 | 4 | 280-288 | Elisabeth Dejin-Karlsson, Bertil S Hanson and Per-Olof Östergren | Psychosocial resources and persistent alcohol consumption in early pregnancy - a population study of women in their first pregnancy in Sweden |
| 1997 | 25 | 4 | 289-295 | Wallis Jansson, Margareta Grafström and Bengt Winblad | Daughters and sons as caregivers for their demented and non-demented elderly parents. A part of a population-based study carried out in Sweden |
| 1997 | 25 | 4 | 296-301 | Walid K. H. Fakhoury, Mark McCarthy and Julia Addington-Hall | Carers' health status: is it associated with their evaluation of the quality of palliative care? |
| 1998 | 26 | 1 | 1-7 | Bjørgulf Claussen | Restricting the influx of disability beneficiaries by means of law: experiences in Norway |
| 1998 | 26 | 1 | 8-9 | David Lester and Ann-Christine Savlid | Homicide and suicide in Swedish Counties |
| 1998 | 26 | 1 | 10-17 | Baizhuang Xu, B.Z. Xu and Paula Rantakallio | Low birth weight in China and Finland |
| 1998 | 26 | 1 | 18-25 | Anders Hjern, Birgitta Angel and Olle Jeppson | Political violence, family stress and mental health of refugee children in exile |
| 1998 | 26 | 1 | 26-33 | M. Elovainio and M. Kivimäki | Work and strain on physicians in Finland |
| 1998 | 26 | 1 | 34-43 | Sären Brage, Jan F Nygård and Gunnar Tellnes | The gender gap in musculoskeletal-related long term sickness absence in Norway |
| 1998 | 26 | 1 | 44-52 | T. Hahn and A. Foldspang | Prevalent knee pain and sport |
| 1998 | 26 | 1 | 53-55 | Juhani Visapää | Significant changes in the terminal care of aged patients in the long-term care in Helsinki |
| 1998 | 26 | 1 | 56-62 | Olov Sandberg, Yngve Gustafson, Benny Brännström and Gustaf Bucht | Prevalence of dementia, delirium and psychiatric symptoms in various care settings for the elderly |
| 1998 | 26 | 1 | 63-70 | Kristinn Tómasson and Per Vaglum | Social consequences of substance abuse: the impact of comorbid psychiatric disorders. A prospective study of a nation-wide sample of treatment-seeking patients |
| 1998 | 26 | 1 | 71-74 | Anna M. S. Kindlundh, Dag G. L. Isacson, Lars Berglund and Fred Nyberg | Doping among high school students in Uppsala, Sweden: A presentation of the attitudes, distribution, side effects, and extent of use |
| 1998 | 26 | 1 | 75-80 | Per-Gunnar Persson, Sofia Carlsson, Valdemar Grill, Ulla Hagman, Ann-Carolin Lundgren, Claes-Göran Östenson, Magdalena Perers and Anna Wallén | Food frequency questionnaire versus 7-day weighed dietary record information on dietary fibre and fat intake in middle-aged Swedish men |
| 1998 | 26 | 2 | 81-84 | Sven Bremberg | Health promotion in school age children |
| 1998 | 26 | 2 | 85-86 | Bengt Lagerkvist | Rehabilitation research under fire |
| 1998 | 26 | 2 | 87-95 | Terje A. Murberg, Edvin Bru, Torbjørn Aarsland and Sven Svebak | Social support, social disability and their role as predictors of depression among patients with congestive heart failure |
| 1998 | 26 | 2 | 96-100 | Pål Gulbrandsen, Per Hjortdahl and Per Fugelli | Work disability and health-affecting psychosocial problems among patients in general practice |
| 1998 | 26 | 2 | 102-105 | Nils-Ove Månsson, Lennart Råstam and Åke Adolfsson | Disability pension in Malmöhus county: Aspects on long term financial effects |
| 1998 | 26 | 2 | 106-114 | Stefanija Tholdy Doncevic, Anders Romelsjö and Töres Theorell | Comparison of stress, job satisfaction, perception of control, and health among district nurses in Stockholm and prewar Zagreb |
| 1998 | 26 | 2 | 115-120 | Karl E. Lund, Anders Skrondal, Harri Vertio and Ásgeir R. Helgason | Children's residential exposure to environmental tobacco smoke varies greatly between the Nordic countries |
| 1998 | 26 | 2 | 121-123 | Merja Ahto, Raimo Isoaho, Hannu Puolijoki, Pekka Laippala, Matti Romo and Sirkka-Liisa Kivelä | Social functioning of elderly coronary heart disease patients |
| 1998 | 26 | 2 | 124-130 | Marianne Malmström, Jan Sundquist, Madhavi Bajekal and Sven-Erik Johansson | Indices of need and social deprivation for primary health care |
| 1998 | 26 | 2 | 131-132 | Walid KH Fakhoury | Quality of palliative care: why nurses are more valued than doctors? |
| 1998 | 26 | 2 | 133-144 | Gunnel Hensing, Kristina Alexanderson, Peter Allebeck and Per Bjurulf | How to measure sickness absence? Literature review and suggestion of five basic measures |
| 1998 | 26 | 2 | 145-153 | Kristiina Manderbacka | Examining what self-rated health question is understood to mean by respondents |
| 1998 | 26 | 2 | 154-160 | Eiliv Lund and Inger Torhild Gram | Response rate according to title and length of questionnaire |
| 1998 | 26 | 3 | 161-165 | Lisa B. Signorello and Dimitrios Trichopoulos | Perinatal determinants of adult cardiovascular disease and cancer |
| 1998 | 26 | 3 | 166-172 | Bo Burström, Finn Diderichsen, Eva Bernhardt and Lars Smedman | Use of a historical register in social epidemiology: child mortality in Stockholm at the turn of the 19th century |
| 1998 | 26 | 3 | 173-180 | Robert Friis, Agneta Yngve and Viveka Persson | Review of social epidemiologic research on migrants' health: findings, methodological cautions, and theoretical perspectives |
| 1998 | 26 | 3 | 181-182 | Kiki Tsamandouraki, Yannis Alamanos, Marina Economou, Natassa Papadopoulou and Yannis Tountas | Planning a worksite health promotion program: health profile of a population of Greek sailors |
| 1998 | 26 | 3 | 183-189 | Anders Knutsson and Tohr Nilsson | Tobacco use and exposure to environmental tobacco smoke in relation to certain work characteristics |
| 1998 | 26 | 3 | 190-197 | Karin A. Melinder and Ragnar Andersson | Differences in injury mortality between the Nordic countries - with special reference to differences in coding practices |
| 1998 | 26 | 3 | 198-203 | Noriyuki Nakanishi, Kozo Tatara, Fumiaki Shinsho, Toshio Takatorige, Shigeki Murakami and Hideki Fukuda | Prevalence of intellectual dysfunctioning and its correlates in a community-residing elderly population |
| 1998 | 26 | 3 | 204-213 | Margareta Leijon, Gunnel Hensing and Kristina Alexanderson | Gender trends in sick-listing with musculoskeletal symptoms in a Swedish county during a period of rapid increase in sickness absence |
| 1998 | 26 | 3 | 214-222 | Mikael Rahmqvist and John Carstensen | Trend of psychological distress in a Swedish population from 1989 to 1995 |
| 1998 | 26 | 3 | 223-231 | Carola Svärdh, Dag Isacson and Nancy L. Pedersen | Self-rated health among cardiovascular drug users in a study of Swedish twins |
| 1998 | 26 | 3 | 232-237 | Olle Hellström, Jennifer Bullington, Gunnar Karlsson, Per Lindqvist and Bengt Mattsson | Doctors' attitudes to fibromyalgia: a phenomenological study |
| 1998 | 26 | 3 | 238 | Bengt Mattsson and Monica Mattsson | To sing in a choir and be healthy — which are the mediating mechanisms? |
| 1998 | 26 | 4 | 241-249 | Kristina Alexanderson | Sickness absence: a review of performed studies with focused on levels of exposures and theories utilized |
| 1998 | 26 | 4 | 250-258 | Jon Håvard Loge and Stein Kaasa | Short Form 36 (SF-36) health survey: normative data from the general Norwegian population |
| 1998 | 26 | 4 | 259-264 | U.-G. Gerdtham and G. Sundberg | Equity in the delivery of health care in Sweden |
| 1998 | 26 | 4 | 265-269 | Jaana Ahlamaa-Tuompo, Miika Linna and Martti Kekomäki | Impact of user charges and socio-economic environment on visits to paediatric trauma unit in Finland |
| 1998 | 26 | 4 | 270-271 | A.-L. Kirkkola, I. Virjo, M. Isokoski and K. Mattila | Finnish health centre physicians' participation in family planning |
| 1998 | 26 | 4 | 272-280 | Annika Rosengren, Kristina Orth-Gomér and Lars Wilhelmsen | Socioeconomic differences in health indices, social networks and mortality among Swedish men. A study of men born in 1933 |
| 1998 | 26 | 4 | 281-288 | Tze-Wai Wong, Siu-Lan Wong, Tak-Sun Yu, Joseph L. Y. Liu and Owen L. Lloyd | Socioeconomic correlates of infant mortality in Hong Kong, 1979-93 |
| 1998 | 26 | 4 | 289-292 | Merete Osler and Søren Klebak | Social differences in health in an affluent Danish county |
| 1998 | 26 | 4 | 293-298 | Merete Osler, Eva Prescott, Adam Gottschau, Anders Bjerg, Hans Ole Hein, Anette Sjøl and Peter Schnohr | Trends in smoking prevalence in Danish adults, 1964–1994: The influence of gender, age, and education |
| 1998 | 26 | 4 | 299 | David Lester | Tobacco consumption and national rates of personal violence (suicide and homicide) |
| 1998 | 26 | 4 | 300-301 | Eve Blair | Risk factors of cerebral palsy |
| 1999 | 27 | 1 | 1-2 |  | EDITORIAL: From "Scandinavian Journal of Social Medicine" to "Scandinavian Journal of Public Health": A Change of Name but not of Vision |
| 1999 | 27 | 1 | 3-4 | Lars Olov Bygren | Need for medical care and social services |
| 1999 | 27 | 1 | 5-10 | Rafael Lindqvist and Owe Grape | Vocational rehabilitation of the socially disadvantaged long-term sick: inter-organizational co-operation between welfare state agencies |
| 1999 | 27 | 1 | 11-17 | Eivind Meland, John Gunnar Mæland and Even Lærum | The importance of self-efficacy in cardiovascular risk factor change |
| 1999 | 27 | 1 | 18-21 | S Van McCrary and Jeffrey W Swanson | Physicians' legal defensiveness and knowledge of medical law: comparing Denmark and the USA |
| 1999 | 27 | 1 | 22-29 | Krister Järbrink, Anders Carlsten and Svend Otto Frederiksen | Swedish physicians' inclination to prescribe benzodiazepines: differences between regions and characteristics of the prescriber |
| 1999 | 27 | 1 | 30-37 | Kajsa-Lena Thundal, Stefan Granbom and Peter Allebeck | Women's alcohol dependence and abuse: the relation to social network and leisure time |
| 1999 | 27 | 1 | 38-42 | Päivi Vaara, Harri Sintonen, Jari Peltonen, Hannele Hokkanen, Mikko Poussa and Soini Ryöppy | Health-related quality of life in patients with diastrophic dysplasia |
| 1999 | 27 | 1 | 43-47 | M. Halldórsson, A. E. J. M. Cavelaars, A. E. Kunst and J. P. Mackenbach | Socioeconomic differences in health and well-being of children and adolescents in Iceland |
| 1999 | 27 | 1 | 48-53 | Knud Juel and Karin Helweg-Larse | Drug-related mortality in Denmark 1970-93 |
| 1999 | 27 | 1 | 54-55 | Mika Gissler and Elina Hemminki | Pregnancy-related violent deaths |
| 1999 | 27 | 1 | 56-62 | L. Anders Nordlund, John M. Carstensen and Göran Pershagen | Are male and female smokers at equal risk of smoking-related cancer: evidence from a Swedish prospective study |
| 1999 | 27 | 1 | 63-72 | Hege R. Eriksen, Camilla Ihlebæk and Holger Ursin | A scoring system for subjective health complaints (SHC) |
| 1999 | 27 | 1 | 73-77 | Holger Theobald, Lars Olov Bygren, John Carstensen and Peter Engfeldt | Validity of two questions on alcohol use in a health survey questionnaire |
| 1999 | 27 | 1 | 78 | Jørn Olsen, Olga Basso and Henrik Toft Sørensen | What is a population-based registry? |
| 1999 | 27 | 2 | 81-84 | Donna Armstrong | "Controversies in epidemiology", teaching causality in context at the University at Albany, School of Public Health |
| 1999 | 27 | 3 | 85-88 | Jukka Hintikka, Pirjo I Saarinen and Heimo Viinamäki | Suicide mortality in Finland during an economic cycle, 1985 - 1995 |
| 1999 | 27 | 3 | 89-93 | Rikke Lund, Jens Modvig, Jørgen Hilden, Nils Rosdahl, Lotte Kure and Kirsten Schmidt | Risk of low birthweight in social districts of Copenhagen |
| 1999 | 27 | 3 | 94-100 | Bjørgulf Claussen | Health and re-employment in a five-year follow-up of long-term unemployed |
| 1999 | 27 | 3 | 101-105 | Dick Larsson, Ibe Lager and Peter M Nilsson | Socio-economic characteristics and quality of life in diabetes mellitus—relation to metabolic control |
| 1999 | 27 | 3 | 106-111 | Marja Jylhä and Antti Hervonen | Functional status and need of help among people aged 90 or over: a mailed survey with a total home-dwelling population |
| 1999 | 27 | 3 | 112-115 | Øydis Ueland | Characteristics of injured skiers in Norway: A case-control study |
| 1999 | 27 | 3 | 116-123 | Lena Holm, J David Cassidy, Yvonne Sjögren and Åke Nygren | Impairment and work disability due to whiplash injury following traffic collisions: An analysis of insurance material from the Swedish Road Traffic Injury Commission |
| 1999 | 27 | 3 | 124-127 | Elina Hemminki, Mika Gissler and Hanna Saarikoski | Variation in referring newborns to special care in Finland |
| 1999 | 27 | 3 | 128-136 | Ole Olsen and Mette Madsen | Effects of maternal education on infant mortality and stillbirths in Denmark |
| 1999 | 27 | 3 | 137-142 | Signild Vallgårda | Is old age necessarily connected with high hospital admission rates? |
| 1999 | 27 | 3 | 143-147 | Arto T Vehviläinen, Esko A Kumpusalo and Jorma K Takala | Drinking problems load health centre hospitals in Finland |
| 1999 | 27 | 3 | 148-151 | Jan Hartvigsen, Svend Lings and Elisabeth H Corder | Coding of occupation for the "young cohort" of the Danish twin register: A resource for future epidemiologic research |
| 1999 | 27 | 3 | 152-159 | Niels Lynöe, Mikael Sandlund and Lars Jacobsson | Research ethics committees: a comparative study of assessment of ethical dilemmas |
| 1999 | 27 | 3 | 161-165 | Carles Muntaner | Teaching social inequalities in health: barriers and opportunities |
| 1999 | 27 | 3 | 166-172 | Elisabeth Rosén | Genetic information and genetic discrimination how medical records vitiate legal protection: A comparative analysis of international legislation and policies |
| 1999 | 27 | 3 | 173-180 | Gert P Westert and Peter P Groenewegen | Medical practice variations: changing the theoretical approach |
| 1999 | 27 | 3 | 181-188 | Terkel Christiansen and Sjoerd Kooiker | Inequalities in health: evidence from Denmark of the interaction of circumstances and health-related behaviour |
| 1999 | 27 | 3 | 189-195 | Marianne Malmström, Jan Sundquist, Sven-Erik Johansson and Leena Maria Johansson | The influence of social deprivation as measured by the CNI on psychiatric admissions |
| 1999 | 27 | 3 | 196-202 | Willy Eriksen, Bård Natvig and Dag Bruusgaard | Marital disruption and long-term work disability: A four-year prospective study |
| 1999 | 27 | 3 | 203-205 | James S Lawson | The link between socioeconomic status and breast cancer—a possible explanation |
| 1999 | 27 | 3 | 206-212 | Thomas A. Cable, Eivind Meland, Tone Søberg and Svein Slagsvold | Lessons from the Oslo Study Diet and Anti-Smoking Trial: a qualitative study of long-term behaviour change |
| 1999 | 27 | 3 | 213-219 | Anne Kjersti Daltveit, Stein Emil Vollset and Lorentz M Irgens | Population density and perinatal mortality in Norway and Sweden 1975-1988 |
| 1999 | 27 | 3 | 220-227 | Christina Ahlgren and Anne Hammarström | Has increased focus on vocational rehabilitation led to an increase in young employees' return to work after work-related disorders? |
| 1999 | 27 | 3 | 228-234 | Magnus A. Björkgren, Unto Häkkinen, U. Harriet Finne-Soveri and Brant E. Fries | Validity and reliability of Resource Utilization Groups (RUG-III) in Finnish long-term care facilities |
| 1999 | 27 | 3 | 235-236 | Margus Lember, Heili Sarapuu, Aili Pikk and Ruth Kalda | Family planning and family doctors in Estonia |
| 1999 | 27 | 3 | 237-238 | Gunilla Bjärås, Lotta Klinge Härberg and Claes-Göran Östenson | Walking campaigns - a useful way to get people involved in physical activity? Experience from the Stockholm Diabetes Prevention Program (SDPP) |
| 1999 | 27 | 3 | 239-240 | David Lester | Predicting the variation in suicide rates in Norwegian counties |
| 1999 | 27 | 4 | 241-244 | Anne Hammarström | Why feminism in public health? |
| 1999 | 27 | 4 | 245-246 | Karen Willis | Feminist public health practice and population-based health strategies: breast cancer screening in Australia |
| 1999 | 27 | 4 | 247-253 | Gunilla Risberg, Eva Lundgren and Göran Westman | Prevalence of sexualized violence among women: A population-based study in a primary healthcare district |
| 1999 | 27 | 4 | 254-259 | Kirsti Malterud and Hanne Hollnagel | Encouraging the strengths of women patients: A case study from general practice on empowering dialogue |
| 1999 | 27 | 4 | 260-266 | Julie Hepworth | Gender and the capacity of women with NIDDM to implement medical advice |
| 1999 | 27 | 4 | 267-272 | Elina Oinas | Young women's perspectives on public health knowledge and adolescent bodies |
| 1999 | 27 | 4 | 273-278 | Marilys N. Guillemin | Managing menopause: a critical feminist engagement |
| 1999 | 27 | 4 | 279-285 | Johanne Sundby | Are women disfavoured in the estimation of Disability Adjusted Life Years and the Global Burden of Disease? |
| 1999 | 27 | 4 | 286-289 | Anne Hammarström and Margie Ripper | What could a feminist perspective on power bring into public health? |
| 1999 | 27 | 4 | 290-295 | Peri J. Ballantyne | The social determinants of health: a contribution to the analysis of gender differences in health and illness |
| 1999 | 27 | 4 | 296-300 | Hilde Grimstad, Berit Schei, Bjørn Backe and Geir Jacobsen | Anxiety, physical abuse, and low birth weight |
| 1999 | 27 | 4 | 301-305 | Eha Nurk, Maurice B Mittelmark, Lagle Suurorg, Inna Tur and Ester Luiga | Trends in tobacco use among Estonian and Russian youth in Tallinn |
| 1999 | 27 | 4 | 306-310 | Ulrica Lövdahl, Åsa Riska and Elianne Riska | Gender display in Scandinavian and American advertising for antidepressants |
| 1999 | 27 | 4 | 311-317 | Gunilla Krantz and Per-Olof Östergren | Women's health: do common symptoms in women mirror general distress or specific disease entities? |
| 2000 | 28 | 1 | 1 | Henrik Saelan, Peter Allebeck, Gudjon Magnusson, Sakari Suominen and Gunnar Tellnes | New name, new format, new editors — Our thanks to the outgoing team |
| 2000 | 28 | 1 | 2-3 | Stig Wall | Public health in transition |
| 2000 | 28 | 1 | 4-9 | Ramune Kalediene and Jadvyga Petrauskiene | Inequalities in life expectancy in Lithuania by level of education |
| 2000 | 28 | 1 | 10-15 | Anne Hammarström and Urban Janlert | Do early unemployment and health status among young men and women affect their chances of later employment? |
| 2000 | 28 | 1 | 16-22 | Karin A Melinder and Ragnar Andersson | Stable and dynamic differences in injury mortality between the Nordic countries. What do they say about inherent national characteristics with regard to risk? |
| 2000 | 28 | 1 | 23-31 | Lena Annerstedt, Sölve Elmståhl, Bengt Ingvad and Sven-Mårten Samuelsson | Family caregiving in dementia: An analysis of the caregiver's burden and the "breaking-point" when home care becomes inadequate |
| 2000 | 28 | 1 | 32-40 | Steffen J. Rosenstock, Torben Jørgensen, Leif P. Andersen and Olaf Bonnevie | Association of Helicobacter pylori infection with lifestyle, chronic disease, body-indices, and age at menarche in Danish adults |
| 2000 | 28 | 1 | 41-46 | Sari K Anttila, Heini S Huhtala, Markku J Pekurinen and Timo K Pitkäjärvi | Cost-effectiveness of an innovative four-year post-discharge programme for elderly patients: Prospective follow-up of hospital and nursing home use in project elderly and randomized controls |
| 2000 | 28 | 1 | 47-53 | Runo Axelsson | The organizational pendulum: Healthcare management in Sweden 1865—1998 |
| 2000 | 28 | 1 | 54-61 | Mette Kjøller and Marie Helweg-Larse | Suicidal ideation and suicide attempts among adult Danes |
| 2000 | 28 | 1 | 62-70 | Helena Hämäläinen, Richard Smith, Pauli Puukka, Jouko Lind, Veikko Kallio, Kari Kuttila and Tapani Rönnemaa | Social support and physical and psychological recovery one year after myocardial infarction or coronary artery bypass surgery |
| 2000 | 28 | 1 | 71-76 | Anders Skrondal, Anne Eskild and Johannes Thorvaldsen | Changes in condom use after HIV diagnosis |
| 2000 | 28 | 1 | 77-78 | Per Tillgren, Tuula Ainetdin and Marie-Louise Stjerna | Classification of non-respondents in a population-based tobacco cessation contest—"Quit and Win" |
| 2000 | 28 | 1 | 79-80 | Jukka Hintikka, Osmo Kontula, Leo Niskanen, Kaj Koskela and Heimo Viinamäki | Increase in the prevalence of common mental disorders during an upswing in the national economy |
| 2000 | 28 | 2 | 81-83 | Elina Hemminki | Hormone replacement therapy: discrepancies between evidence and recommendations |
| 2000 | 28 | 2 | 84-87 | Elianne Riska | Women's health: issues and prospects |
| 2000 | 28 | 2 | 88-94 | Christina Ahlgren and Anne Hammarström | Back to work? Gendered experiences of rehabilitation |
| 2000 | 28 | 2 | 95-101 | Anu Sauvola, Taru Mäkikyrö, Jari Jokelainen, Matti Joukamaa, Marjo-Riitta Järvelin and Matti Isohanni | Single-parent family background and physical illness in adulthood: a follow-up study of the Northern Finland 1966 Birth Cohort |
| 2000 | 28 | 2 | 102-110 | Ewa Forsberg, Runo Axelsson and Bengt Arnetz | Effects of performance-based reimbursement in healthcare |
| 2000 | 28 | 2 | 111-116 | Tore Nilstun, Gunilla Melltorp and Göran Hermerén | Surveys on attitudes to active euthanasia and the difficulty of drawing normative conclusions |
| 2000 | 28 | 2 | 117-122 | Marko Elovainio, Marjukka Mäkelä, Timo Sinervo, Mika Kivimäki, Martin Eccles and James Kahan | Effects of job characteristics, team climate, and attitudes towards clinical guidelines |
| 2000 | 28 | 2 | 123-131 | Jan Sundquist and Sven-Erik Johansson | High demand, low control, and impaired general health: working conditions in a sample of Swedish general practitioners |
| 2000 | 28 | 2 | 132-136 | Ewa Menckel and Lucie Laflamme | Injuries to boys and girls in Swedish schools: different activities, different results? |
| 2000 | 28 | 2 | 137-145 | Anne-Louise Bergh, Lars-Olof Persson and Stig Attvall | Psychometric properties of the Swedish version of the Well-Being Questionnaire in a sample of patients with diabetes type |
| 2000 | 28 | 2 | 146-153 | Anders Foldspang and Edith Montgomery | Criterion-validity-based assessment of four scale constructs |
| 2000 | 28 | 2 | 154-157 | Margarete Eriksson, Björn Jonsson, Gun Steneroth and Rolf ZettRhidian Hugheserström | Amphetamine abuse during pregnancy: environmental factors and outcome after 14-15 years |
| 2000 | 28 | 2 | 158-160 | Rhidian Hughes | Illicit drug injectors' strategies for cleaning needles and syringes: findings from qualitative research |
| 2000 | 28 | 3 | 161-163 | Thomas A. Pearson | Scandinavia's lessons to the world of public health |
| 2000 | 28 | 3 | 164-173 | Andres Vikat, Matti Rimpelä, Jouko J Salminen, Arja Rimpelä, Annikki Savolainen and Suvi M Virtanen | Neck or shoulder pain and low back pain in Finnish adolescents |
| 2000 | 28 | 3 | 174-178 | Boinkum B Konlaan, Lars O Bygren and Sven-Erik Johansson | Visiting the cinema, concerts, museums or art exhibitions as determinant of survival: a Swedish fourteen-year cohort follow-up |
| 2000 | 28 | 3 | 179-187 | Sakari B. Suominen, Raili S. Välimaa, Jorma A. Tynjälä and Lasse K Kannas | Minority status and perceived health: a comparative study of Finnish- and Swedish-speaking schoolchildren in Finland |
| 2000 | 28 | 3 | 188-193 | Eva Sellström, Sven Bremberg, Anita Gärling and Jan Olof Hörnquist | Risk of childhood injury: predictors of mothers' perceptions |
| 2000 | 28 | 3 | 194-199 | Henrik Brønnum-Hansen | Socioeconomic differences in health expectancy in Denmark |
| 2000 | 28 | 3 | 200-208 | Martin Lindström, Bertil S. Hanson, Per-Olof Östergren and Göran Berglund | Socioeconomic differences in smoking cessation: the role of social participation |
| 2000 | 28 | 3 | 209-213 | Knut Steen and Steinar Hunskaar | Violence in an urban community: a population-based interview study |
| 2000 | 28 | 3 | 214-221 | Karin Helweg-Larsen and Knud Juel | Sex differences in mortality in Denmark during half a century, 1943–92 |
| 2000 | 28 | 3 | 222-229 | Ann-Kristin M. Rönnberg and Anne Hammarström | Barriers within the health care system to dealing with sexualized violence: a literature review |
| 2000 | 28 | 3 | 230-239 | Jan Hartvigsen, Charlotte Leboeuf-Yde, Svend Lings and Elisabeth H Corder | Is sitting-while-at-work associated with low back pain? A systematic, critical literature review |
| 2000 | 28 | 4 | 241-243 | Finn Kamper-Jørgensen | Knowledge-base, evidence and evaluation in Public Health |
| 2000 | 28 | 4 | 244-252 | Inger Sandanger, Jan F. Nygård, Sören Brage and Gunnar Tellnes | Relation between health problems and sickness absence: gender and age differences: A comparison of low-back pain, psychiatric disorders, and injuries |
| 2000 | 28 | 4 | 253-259 | Susanne Gustafsson-Larsson and Anne Hammarström | Can women's network activities lead to improved health? |
| 2000 | 28 | 4 | 260-265 | Vassiliki Benetou, Yiannis Chloptsios, Xenofon Zavitsanos, Dimitrios Karalis, Androniki Naska and Antonia Trichopoulou | Total cholesterol and HDL-cholesterol in relation to socioeconomic status in a sample of 11,645 Greek adults: the EPIC study in Greece |
| 2000 | 28 | 4 | 266-269 | Liisa A. Hiltunen and Sirkka M. Keinänen-Kiukaanniemi | Effects of the WHO 1985 criteria and the proposed new diagnostic criteria on the prevalence of diabetes in an elderly population |
| 2000 | 28 | 4 | 270-274 | Anna Birna Almarsdóttir and Almar Grímsson | Over-the-counter codeine use in Iceland: the impact of increased access |
| 2000 | 28 | 4 | 275-282 | Michael Bohman, Peter Wennberg and Tommy Andersson | Alcohol habits in a suburban male cohort |
| 2000 | 28 | 4 | 283-293 | Leeni T. Berntsson and Jan-Eric Gustafsson | Determinants of psychosomatic complaints in Swedish schoolchildren aged seven to twelve years |
| 2000 | 28 | 4 | 294-297 | Juan Merlo, Per-Olof Östergren, Nils-Ove Månsson, Bertil S Hanson, Jonas Ranstam, Göran Blennow, Sven-Olof Isacsson and Arne Melander | Mortality in elderly men with low psychosocial coping resources using anxiolytic-hypnotic drugs |
| 2000 | 28 | 4 | 298-308 | Charli Eriksson | Learning and knowledge-production for public health: a review of approaches to evidence-based public health |
| 2000 | 28 | 4 | 309-311 | B. I. B. Lindahl | Health and evolution |
| 2000 | 28 | 4 | 312-316 | Peter Wennberg, Michael Bohman and Tommy Andersson | Variations and stability in drinking patterns in a cohort of Swedish males |
| 2000 | 28 | 4 | 317 | Piroska Östlin | Reviewed Work: Norwegian Journal of Epidemiology on "Gender Inequalities in Health" (No. 2) by |
| 2001 | 29 | 1 | 1-4 | Maria Emmelin | Challenges for publishing medical and public health research in the 2000s |
| 2001 | 29 | 1 | 5-12 | Kim Moesgaard Iburg, Henrik Brønnum-Hansen and Peter Bjerregaard | Health expectancy in Greenland |
| 2001 | 29 | 1 | 13-22 | Carola Bardage, Dag Isacson and Nancy L. Pedersen | Self-rated health as a predictor of mortality among persons with cardiovascular disease in Sweden |
| 2001 | 29 | 1 | 23-31 | Willy Eriksen, Bård Natvig and Dag Bruusgaard | Sleep problems: a predictor of long-term work disability? A four-year prospective study |
| 2001 | 29 | 1 | 32-39 | Merete Osler, Bjørn Holstein, Kirsten Avlund, Mogens Trab Damsgaard and Niels Kr Rasmussen | Socioeconomic position and smoking behaviour in Danish adults |
| 2001 | 29 | 1 | 40-42 | Merete Osler, Torben Jørgensen, Michael Davidsen, Morten Grønbæk, Henrik Brønnum-Hansen, Mette Madsen, Ulrik Gerdes and Marianne Schroll | Socioeconomic status and trends in alcohol drinking in the Danish MONICA population, 1982-92 |
| 2001 | 29 | 1 | 44-54 | Leeni T Berntsson, Lennart Köhler and Jan-Eric Gustafsson | Psychosomatic complaints in schoolchildren: a Nordic comparison |
| 2001 | 29 | 1 | 55-62 | Annette Wigertz and Ragnar Westerling | Measures of prevalence: which healthcare registers are applicable? |
| 2001 | 29 | 1 | 63-70 | Christina Lindholm, Bo Burström and Finn Diderichsen | Does chronic illness cause adverse social and economic consequences among Swedes? |
| 2001 | 29 | 1 | 71-75 | Elin O Rosvold and Espen Bjertness | Physicians who do not take sick leave: hazardous heroes? |
| 2001 | 29 | 1 | 76-77 | Måns Rosén | Can the WHO Health Report improve the performance of health systems? |
| 2001 | 29 | 1 | 78-80 | Christopher JL Murray and Julio Frenk Mora | A reply to Rosén |
| 2001 | 29 | 2 | 81-83 | Robert Beaglehole and Ruth Bonita | Challenges for public health in the global context — prevention and surveillance |
| 2001 | 29 | 2 | 84-86 | Peter Byass | Person, place and time — but who, where, and when? |
| 2001 | 29 | 2 | 87-95 | Irene Isohanni, Marjo-Riitta Järvelin, Paula Rantakallio, Jari Jokelainen, Peter B Jones, Pentti Nieminen, Tim Croudace and Matti Isohanni | Juvenile and early adulthood smoking and adult educational achievements — A 31-year follow-up of the Northern Finland 1966 Birth Cohort |
| 2001 | 29 | 2 | 96-103 | Marianne Upmark, Ingvar Lundberg, Jonas Sadigh and Carolina Bigert | Conditions during childhood and adolescence as explanations of social class differences in disability pension among young men |
| 2001 | 29 | 2 | 104-112 | Karin Borg, Gunnel Hensing and Kristina Alexanderson | Predictive factors for disability pension — An 11-year follow up of young persons on sick leave due to neck, shoulder, or back diagnoses |
| 2001 | 29 | 2 | 113-121 | Franka JM Meiland, Jos AC Danse, Johannes F Wendte, Niek S Klazinga and Louise J Gunning-Schepers | Caring for relatives with dementia — Caregiver experiences of relatives of patients on the waiting list for admission to a psychogeriatric nursing home in The Netherlands |
| 2001 | 29 | 2 | 122-128 | Johan Lökk, Mats Nilsson, Bo Norberg, Johan Hultdin, Herbert Sandström and Göran Westman | Shifts in B₁₂ opinions in primary health care of Sweden |
| 2001 | 29 | 2 | 129-132 | Bjørn Backe | Overutilization of antenatal care in Norway |
| 2001 | 29 | 2 | 133-139 | Nils-Ove Månsson, Juan Merlo and Per-Olof Östergren | The use of analgesics and hypnotics in relation to self-rated health and disability pension — A prospective study of middle-aged men |
| 2001 | 29 | 2 | 140-150 | Margarete Kristenson, Zita Kucinskiene, Björn Bergdahl and Kristina Orth-Gomér | Risk factors for coronary heart disease in different socioeconomic groups of Lithuania and Sweden — the LiVicordia study |
| 2001 | 29 | 2 | 151-158 | Nils-Ove Månsson and Lennart Råstam | Self-rated health as a predictor of disability pension and death — A prospective study of middle-aged men |
| 2001 | 29 | 3 | 161-165 | Krisela Steyn and Debbie Bradshaw | Non-communicable disease surveillance in developing countries |
| 2001 | 29 | 3 | 166-174 | Margaretha Voss, Birgitta Floderus and Finn Diderichsen | Changes in sickness absenteeism following the introduction of a qualifying day for sickness benefit – findings from Sweden Post |
| 2001 | 29 | 3 | 175-182 | Pål Nystuen, Kåre B. Hagen and Jeph Herrin | Mental health problems as a cause of long-term sick leave in the Norwegian workforce |
| 2001 | 29 | 3 | 183-188 | Kirsti Malterud, Hanne Hollnagel and Klaus Witt | Gendered health resources and coping – A study from general practice |
| 2001 | 29 | 3 | 189-193 | Tuula M Pesonen, Jukka Hintikka, Kari O Karkola, Pirjo I Saarinen, Marja Antikainen and Johannes Lehtonen | Male suicide mortality in eastern Finland – urban-rural changes during a 10-year period between 1988 and 1997 |
| 2001 | 29 | 3 | 194-199 | Knut Stavem, Dag Hofoss, Olaf Gjerløw Aasland and Jon Håvard Loge | The self-perceived health status of Norwegian physicians compared with a reference population and foreign physicians |
| 2001 | 29 | 3 | 200-207 | Lucy J Robertson and Bjørn Gjerde | Occurrence of "Cryptosporidium" oocysts and "Giardia" cysts in raw waters in Norway |
| 2001 | 29 | 3 | 208-217 | Margareta Barnekow-Bergkvist, Gudrun Hedberg, Urban Janlert and Eva Jansson | Adolescent determinants of cardiovascular risk factors in adult men and women |
| 2001 | 29 | 3 | 215-225 | Harald Reiso, Jan F Nygård, Sören Brage, Pål Gulbrandsen and Gunnar Tellnes | Work ability and duration of certified sickness absence |
| 2001 | 29 | 3 | 226-232 | Ari Haukkala, Mikko Laaksonen and Antti Uutela | Smokers who do not want to quit – Is consonant smoking related to lifestyle and socioeconomic factors? |
| 2001 | 29 | 3 | 233-239 | Karin C Ringsberg, Kristina A E Alexanderson, Karin E Borg and Gunnel K E Hensing | The health-line – a method for collecting data on self-rated health over time: a pilot study |
| 2001 | 29 | 4 | 241-244 | Derek Yach | Economics and public health - reflections from the past and challenges for the future |
| 2001 | 29 | 4 | 245 |  | Public health in Scandinavia – the case of Sweden |
| 2001 | 29 | 4 | 246-247 | Gunnar Ågren | The new Swedish national public health policy |
| 2001 | 29 | 4 | 248-255 | Janecke Thesen | Being a psychiatric patient in the community – reclassified as the stigmatized "other" |
| 2001 | 29 | 4 | 256-262 | Kirsten Fonager and Svend Sabroe | A comparative analysis of different methods for obtaining estimates of alcohol consumption in a Danish population survey |
| 2001 | 29 | 4 | 263-270 | Pia Maria Jonsson, Lennarth Nyström, Göran Sterky and Stig Wall | Sociodemographic predictors of self-rated health in patients with diabetes of short duration |
| 2001 | 29 | 4 | 271-278 | Örjan Garpenholt, Hans Fredlund and Toomas Timpka | Immunization against Haemophilus influenzae type b in Sweden – A study of the introduction process |
| 2001 | 29 | 4 | 279-284 | Eleni Petridou, Maria Moustaki, Efi Gemanaki, Carol Djeddah and Dimitrios Trichopoulos | Intentional childhood injuries in Greece 1996-97 – Data from a population-based Emergency Department Injury Surveillance System (EDISS) |
| 2001 | 29 | 4 | 285-291 | Gunnel Östlund, Elisabet Cedersund, Kristina Alexanderson and Gunnel Hensing | "It was really nice to have someone" – Lay people with musculoskeletal disorders request supportive relationships in rehabilitation |
| 2001 | 29 | 4 | 292-299 | Maila Upanne | A model-based analysis of professional practices in suicide prevention |
| 2001 | 29 | 4 | 300-307 | Jørn Olsen, Mads Melbye, Sjurdur F Olsen, Thorkild IA Sorensen, Peter Aaby, Anne-Marie Nybo Andersen, Dorthe Taxbøl, Kit Dynnes Hansen, Mette Juhl, Tina Broby Schow, Henrik Toft Sørensen, Jente Andresen, Erik Lykke Mortensen, Annette Wind Olesen and Charlotte Søndergaard | The Danish National Birth Cohort – its background, structure and aim |
| 2001 | 29 | 4 | 308-313 | Sijmen Reijneveld, Robert Verheij, Loes van Herten, Dinny de Bakker and S.A. REIJNEVELD | Contacts of general practitioners with illegal immigrants |
| 2001 | 29 | 4 | 314-317 | M. Rosaria Galanti, Ingvar Rosendahl, Ann Post and Hans Gilljam | Early gender differences in adolescent tobacco use — The experience of a Swedish cohort |
| 2001 | 29 | 4 | 318-319 | Lene Dreyer, Jeanette Falck Winther and Jørgen Olsen | Avoidable deaths from cancer in the Nordic countries around the year 2000 |
| 2002 | 30 | 1 | 1-4 | Peter Allebeck | The revised Helsinki declaration: Good for patients? Good for public health? |
| 2002 | 30 | 1 | 5-11 | Henrik Brønnum-Hansen | Predicting the effect of prevention of ischaemic heart disease |
| 2002 | 30 | 1 | 12-19 | Bo Hedblad, Juan Merlo, Jonas Manjer, Gunnar Engström, Göran Berglund and Lars Janzon | Incidence of cardiovascular disease, cancer and death in postmenopausal women affirming use of hormone replacement therapy |
| 2002 | 30 | 1 | 20-29 | Camilla Ihlebæk, Hege R. Eriksen and Holger Ursin | Prevalence of subjective health complaints (SHC) in Norway |
| 2002 | 30 | 1 | 30-35 | Rune Lindqvist and Hans Åberg | Locus of control in relation to smoking cessation during pregnancy |
| 2002 | 30 | 1 | 36-40 | Merete Osler, Torben Jørgensen, Lars U Gerdes, Michael Davidsen, Henrik Brønnum-Hansen, Mette Madsen and Marianne Schroll | Changes in community and individual level psychosocial coronary risk factors in the Danish MONICA population, 1982-92 |
| 2002 | 30 | 1 | 41-46 | Nelson Blank and Bo Burström | Limiting long-term illness and the experience of financial strain in Sweden |
| 2002 | 30 | 1 | 47-53 | Yrsa A. Hundrup, Henrik Thoning, Erik B. Obel, Niels K. Rasmussen and John Philip | Lifestyle factors and choice of hormone replacement therapy among Danish nurses |
| 2002 | 30 | 1 | 54-61 | Jane Jensen, Lillemor Lundin-Olsson, Lars Nyberg and Yngve Gustafson | Falls among frail older people in residential care |
| 2002 | 30 | 1 | 62-69 | Mette Rasmussen, Mogens T. Damsgaard, Pernille Due and Bjørn E. Holstein | Boys and girls smoking within the Danish elementary school classes: a group-level analysis |
| 2002 | 30 | 1 | 70-75 | Wenche Nystad, Per Nafstad and Jouni J. K. Jaakkola | The effect of respiratory tract infections on reported asthma symptoms |
| 2002 | 30 | 1 | 76-79 | Margareta Troein, Lennart Råstam and Staffan Selander | Changes in health beliefs after labelling with hypercholesterolaemia |
| 2002 | 30 | 2 | 81-85 | Måns Rosén | National Health Data Registers: a Nordic heritage to public health |
| 2002 | 30 | 2 | 86-93 | NV Toan, LN Trong, B Höjer and LÅ Persson | Public health services use in a mountainous area, Vietnam: implications for health policy |
| 2002 | 30 | 2 | 94-102 | Måns Waldenström, Töres Theorell, Gunnel Ahlberg, Malin Josephson, Patrik Nise, Kerstin Waldenström, Eva Vingård and the MUSIC-study group | Assessment of psychological and social current working conditions in epidemiological studies: experiences from the MUSIC-Norrtälje study |
| 2002 | 30 | 2 | 103-112 | Jonas Manjer, Sölve Elmståhl, Lars Janzon and Göran Berglund | Invitation to a population-based cohort study: differences between subjects recruited using various strategies |
| 2002 | 30 | 2 | 113-124 | Steinar Krokstad and Steinar Westin | Health inequalities by socioeconomic status among men in the Nord-Trøndelag Health Study, Norway |
| 2002 | 30 | 2 | 125-132 | Elin O. Rosvold and Espen Bjertness | Illness behaviour among Norwegian physicians |
| 2002 | 30 | 2 | 133-140 | Kaire Innos, Kaja Rahu, Aleksei Baburin and Mati Rahu | Cancer incidence and cause-specific mortality in male and female physicians: a cohort study in Estonia |
| 2002 | 30 | 2 | 141-147 | Ásgeir R Helgason and Karl E Lund | General practitioners' perceived barriers to smoking cessation – results from four Nordic countries |
| 2002 | 30 | 2 | 148-160 | Sylvia F. Kaaya, Alan J. Flisher, Jessie K. Mbwambo, Herman Schaalma and Leif Edvard Aarø | A review of studies of sexual behaviour of school students in sub-Saharan Africa |
| 2002 | 30 | 3 | 161 |  | Highlights from this issue of Scandinavian Journal of Public Health |
| 2002 | 30 | 3 | 162-165 | Stig Wall | Bridging the gaps: can we afford not to invest in global health? |
| 2002 | 30 | 3 | 166-175 | Lena Hillert, Pirjo Savlin, Adrienne Levy Berg, Anna Heidenberg and Birgitta Kolmodin-Hedman | Environmental illness — effectiveness of a salutogenic group-intervention programme |
| 2002 | 30 | 3 | 176-183 | Gunilla Krantz and Per-Olof Östergren | Do common symptoms in women predict long spells of sickness absence? A prospective community-based study on Swedish women 40 to 50 years of age |
| 2002 | 30 | 3 | 184-190 | Leif Persson, Per-Olof Östergren, Bertil S. Hanson, Anna Lindgren and Anders Naucler | Social network, social support and the rate of decline of CD4 lymphocytes in asymptomatic HIV-positive homosexual men |
| 2002 | 30 | 3 | 191-199 | Marianne Upmark and Kajsa-Lena Thundal | An explorative, population-based study of female disability pensioners: the role of childhood conditions and alcohol abuse/dependence |
| 2002 | 30 | 3 | 200-208 | Sirkka L. Elo and Johan B. Calltorp | Health promotive action and preventive action model (HPA model) for the classification of health care services in public health nursing |
| 2002 | 30 | 3 | 209-215 | R. Sutinen, M. Kivimäki, M. Elovainio and M. Virtanen | Organizational fairness and psychological distress in hospital physicians |
| 2002 | 30 | 3 | 216-222 | Thomas Quaade, Gerda Engholm, Anne Mette T. Johansen and Henrik Møller | Mortality in relation to early retirement in Denmark: a population-based study |
| 2002 | 30 | 3 | 223-230 | Jørn Hetland, Torbjørn Torsheim and Leif E. Aarø | Subjective health complaints in adolescence: dimensional structure and variation across gender and age |
| 2002 | 30 | 3 | 231-237 | Amir Baigi, Benkt Högstedt, Anders Odén, Sven-Olof Isacsson and Per Herrström | Life expectancy in the province of Halland, Sweden, 1911-50: the progress of public health in a long-living population |
| 2002 | 30 | 3 | 238-239 | Måns Rosén, Rikard Lindqvist and Magnus Stenbeck | Revise the review process of the Cochrane collaboration |
| 2002 | 30 | 4 | 241-243 | Olle Lundberg and Joakim Palme | A balance sheet for welfare: Sweden in the 1990s |
| 2002 | 30 | 4 | 244-248 | Sigurður Thorlacius, Sigurjón B. Stefánsson, Stefán Ólafsson and Vilhjálmur Rafnsson | Changes in the prevalence of disability pension in Iceland 1976-1996 |
| 2002 | 30 | 4 | 249-258 | Michael Lundberg, Finn Diderichsen and Johan Hallqvist | Is the association between short stature and myocardial infarction explained by childhood exposures – a population-based case referent study (SHEEP) |
| 2002 | 30 | 4 | 259-266 | Daniel Larsson, Tomas Hemmingsson, Peter Allebeck and Ingvar Lundberg | Self-rated health and mortality among young men: what is the relation and how may it be explained? |
| 2002 | 30 | 4 | 267-273 | Nils-Ove Månsson, Juan Merlo and Per-Olof Östergren | Is there an interaction between self-rated health and medication with analgesics and hypnotics in the prediction of disability pension? |
| 2002 | 30 | 4 | 274-280 | Anna Kattainen, Antti Reunanen, Seppo Koskinen, Tuija Martelin, Paul Knekt and Arpo Aromaa | Secular changes in prevalence of cardiovascular diseases in elderly Finns |
| 2002 | 30 | 4 | 281-287 | Sissel Steihaug, Birgitte Ahlsen and Kirsti Malterud | "I am allowed to be myself": women with chronic muscular pain being recognized |
| 2002 | 30 | 4 | 288-292 | Bård Natvig, Willy Eriksen and Dag Bruusgaard | Low back pain as a predictor of long-term work disability |
| 2002 | 30 | 4 | 293-299 | Vibeke Rasch, Hanne Wielandt and Lisbeth B. Knudsen | Living conditions, contraceptive use and the choice of induced abortion among pregnant women in Denmark |
| 2002 | 30 | 4 | 300-305 | Elise A-L Kosunen, Andres Vikat, Mika Gissler and Matti K. Rimpelä | Teenage pregnancies and abortions in Finland in the 1990s |
| 2002 | 30 | 4 | 306-312 | Dina C Balabanova and Martin McKee | Self-reported health in Bulgaria: levels and determinants |
| 2002 | 30 | 4 | 313-314 | Robert Erikson and Gordon Marshall | An olive tree for public health and social research is being planted |
| 2002 | 30 | 4 | 315-316 | Lars Bo Andersen and Bengt Saltin | Comments on Brønnum-Hansen: Predicting the effect of prevention of ischaemic heart disease |
| 2002 | 30 | 4 | 317-318 | Henrik Brønnum-Hansen | A reply to Lars Bo Andersen and Bengt Saltin |
| 2002 | 30 | 4 | 319 |  | Highlights from the next issue of Scandinavian Journal of Public Health |
| 2003 | 31 | 1 | 1-4 | Mikko Kautto | Welfare in Finland in the 1990s |
| 2003 | 31 | 1 | 5-11 | Ulrik Kesmodel, Pia S Kesmodel, Alice Larsen and Niels J Secher | Use of alcohol and illicit drugs among pregnant Danish women, 1998 |
| 2003 | 31 | 1 | 12-16 | Helle Larsen, Gunnar L. Nielsen, Jörgen Bendsen, Carolin Flint, Jørn Olsen and Henrik T. Sørensen | Predictive value and completeness of the registration of congenital abnormalities in three Danish population-based registries |
| 2003 | 31 | 1 | 17-23 | Hilde H. Holte, Kristian Tambs and Tor Bjerkedal | Time trends in disability pensioning for rheumatoid arthritis, osteoarthritis and soft tissue rheumatism in Norway 1968–97 |
| 2003 | 31 | 1 | 24-30 | Susanne Høidrup, Thorkild I. A. Sørensen, Morten Grønbæk and Marianne Schroll | Incidence and characteristics of falls leading to hospital treatment: a one-year population surveillance study of the Danish population aged 45 years and over |
| 2003 | 31 | 1 | 31-36 | Unnur Valdimarsdóttir, Ásgeir R. Helgason, Carl-Johan Fürst, Jan Adolfsson and Gunnar Steineck | Long-term effects of widowhood after terminal cancer: a Swedish nationwide follow-up |
| 2003 | 31 | 1 | 37-43 | Mikko Laaksonen, Ritva Prättälä and Eero Lahelma | Sociodemographic determinants of multiple unhealthy behaviours |
| 2003 | 31 | 1 | 44-50 | Monica Christianson, Eva Johansson, Maria Emmelin and Göran Westman | "One-night stands" – risky trips between lust and trust: qualitative interviews with Chlamydia trachomatis infected youth in north Sweden |
| 2003 | 31 | 1 | 51-57 | Karin Helweg-Larsen and Marie Kruse | Violence against women and consequent health problems: a register-based study |
| 2003 | 31 | 1 | 58-62 | Peter Byass, Yemane Berhane, Anders Emmelin and Stig Wall | Patterns of local migration and their consequences in a rural Ethiopian population |
| 2003 | 31 | 1 | 63-68 | Migle Gamperiene, Jan F. Nygård, Sören Brage, Tor Bjerkedal and Dag Bruusgaard | Duration of employment is not a predictor of disability of cleaners: a longitudinal study |
| 2003 | 31 | 1 | 69-72 | Juha M. Veijola, Pirjo H. Mäki, Matti I. Joukamaa, Esa Läärä, Helinä Hakko, Markku M. Nieminen and Matti K. Isohanni | Adulthood mortality of infants isolated at birth due to tuberculosis in the family |
| 2003 | 31 | 1 | 73-76 | Cecilia Ekéus and Kyllike Christensson | Socioeconomic characteristics of fathers of children born to teenage mothers in Stockholm, Sweden |
| 2003 | 31 | 1 | 77-78 |  | Public health websites |
| 2003 | 31 | 1 | 79 |  | Acknowledgements |
| 2003 | 31 | 1 | 80 |  | Highlights for next issue |
| 2003 | 31 | 2 | 81-84 | Grete Botten, Kari Tove Elvbakken and Nanna Kildal | The Norwegian welfare state on the threshold of a new century |
| 2003 | 31 | 2 | 85-91 | Jyrki A Korkeila, Saara Töyry, Kirsti Kumpulainen, Juha-Matti Toivola, Kimmo Räsänen and Raija Kalimo | Burnout and self-perceived health among Finnish psychiatrists and child psychiatrists: a national survey |
| 2003 | 31 | 2 | 92-99 | Peter Bjerregaard, Eric Dewailly, T. Kue Young, Carole Blanchet, Robert A. Hegele, Sven E. O. Ebbesson, Patricia M. Risica and Gert Mulvad | Blood pressure among the Inuit (Eskimo) populations in the Arctic |
| 2003 | 31 | 2 | 100-106 | Tommi T. Sulander, Ossi J. Rahkonen and Antti K. Uutela | Functional ability in the elderly Finnish population: time period differences and associations, 1985-99 |
| 2003 | 31 | 2 | 107-112 | Sissel Steihaug and Kirsti Malterud | Part process analysis: a qualitative method for studying provider-patient interaction |
| 2003 | 31 | 2 | 113-118 | Ingela Danielsson, Inga Sjöberg, Hans Stenlund and Marianne Wikman | Prevalence and incidence of prolonged and severe dyspareunia in women: results from a population study |
| 2003 | 31 | 2 | 119-125 | Christian Kronborg Andersen, Jørgen Lauridsen, Kjeld Andersen and Per Kragh-Sørensen | Cost of dementia: impact of disease progression estimated in longitudinal data |
| 2003 | 31 | 2 | 125-136 | Kirsten Avlund, Bjørn E. Holstein, Merete Osler, Mogens T. Damsgaard, Poul Holm-Pedersen and Niels K. Rasmussen | Social position and health in old age: the relevance of different indicators of social position |
| 2003 | 31 | 2 | 137-142 | Aldona Gaiz̆auskienė, Z̆ilvinas Padaiga, Vytautas Basys, Grigorij Grigorjev and Rūta Mizerienė | Risk factors of perinatal mortality in Lithuania, 1997-1998 |
| 2003 | 31 | 2 | 143-148 | Marlene K. Ockander and Toomas Timpka | Women's experiences of long term sickness absence: implications for rehabilitation practice and theory |
| 2003 | 31 | 2 | 149-155 | Ingrid Willaing, Torben Jørgensen and Lars Iversen | How does individual smoking behaviour among hospital staff influence their knowledge of the health consequences of smoking? |
| 2003 | 31 | 2 | 156-157 | Per-Erik Liss | Hard choices in public health: the allocation of scarce resources |
| 2003 | 31 | 2 | 158 |  | PUBLIC HEALTH CALENDAR |
| 2003 | 31 | 2 |  |  | PUBLIC HEALTH CALENDAR |
| 2003 | 31 | 3 | 161-168 | Maria Emmelin and Stig Wall | Our editorial process - some experiences and reflections |
| 2003 | 31 | 3 | 169-177 | Katja Gillander Gådin and Anne Hammarström | Do changes in the psychosocial school environment influence pupils' health development? Results from a three-year follow-up study |
| 2003 | 31 | 3 | 178-186 | Beate Søholt Lupton, Vinjar Fønnebø and Anne Johanne Søgaard | The Finnmark Intervention Study: is it possible to change CVD risk factors by community-based intervention in an Arctic village in crisis? |
| 2003 | 31 | 3 | 187-193 | Birger Aaen-Larsen and Peter Bjerregaard | Changes in causes of death and mortality rates among children in Greenland from 1987-91 to 1992-99 |
| 2003 | 31 | 3 | 194-203 | Monica Mortimer, Gunnel Ahlberg and the MUSIC-Norrtälje study group | To seek or not to seek? Care-seeking behaviour among people with low-back pain |
| 2003 | 31 | 3 | 204-210 | Saadhna Panday, Sasiragha P. Reddy and Erik Bergström | A qualitative study on the determinants of smoking behaviour among adolescents in South Africa |
| 2003 | 31 | 3 | 211-215 | Gunnel Boström and Måns Rosén | Measuring social inequalities in health - politics or science? |
| 2003 | 31 | 3 | 216-223 | Melkiory C. Masatu, Gunnar Kvåle and Knut-Inge Klepp | Frequency and perceived credibility of reported sources of reproductive health information among primary school adolescents in Arusha, Tanzania |
| 2003 | 31 | 3 | 224-228 | Anders Carlsten, Margda Waern, Per Holmgren and Peter Allebeck | The role of benzodiazepines in elderly suicides |
| 2003 | 31 | 3 | 229-232 | Anne Hammarström and Urban Janlert | Unemployment - an important predictor for future smoking: a 14-year follow-up study of school leavers |
| 2003 | 31 | 3 | 233-237 | Cecilia Boldeman, Bjarne Jansson, Henrik Dal and Henrik Ullén | Sunbed use among Swedish adolescents in the 1990s: a decline with an unchanged relationship to health risk behaviors |
| 2003 | 31 | 3 | 238 |  | PUBLIC HEALTH CALENDAR |
| 2003 | 31 | 3 |  |  | Highlights for next issue |
| 2003 |  | 4 | 241-245 | Jon Kvist | A Danish welfare miracle? Policies and outcomes in the 1990s |
| 2003 |  | 4 | 242546 | Sturla Gjesdal and Espen Bratberg | Diagnosis and duration of sickness absence as predictors for disability pension: Results from a three-year, multi-register based and prospective study |
| 2003 |  | 4 | 255-260 | Peter M. Nilsson, Marianne Engberg, Jan-Åke Nilsson, Bo Karlsmose and Torsten Lauritzen | Adverse social factors predict early ageing in middle-aged men and women: the Ebeltoft Health Study, Denmark |
| 2003 |  | 4 | 261-266 | Lisa Forsén, Christian Arstad, Sidsel Sandvig, Annemarie Schuller, Ulf Røed and Anne J Søgaard | Prevention of hip fracture by external hip protectors: an intervention in 17 nursing homes in two municipalities in Norway |
| 2003 |  | 4 | 267-274 | Alastair H. Leyland and Peter P. Groenewegen | Multilevel modelling and public health policy |
| 2003 |  | 4 | 275-282 | Gunnel M Östlund, Karin E Borg, Peter Wide, Gunnel K E Hensing and Kristina A E Alexanderson | Clients' perceptions of contact with professionals within healthcare and social insurance offices |
| 2003 |  | 4 | 283-290 | Karri Silventoinen, Pekka Jousilahti, Erkki Vartiainen and Jaakko Tuomilehto | Appropriateness of anthropometric obesity indicators in assessment of coronary heart disease risk among Finnish men and women |
| 2003 |  | 4 | 292-296 | Phillimon Ndubani, Virginia Bond, Rita Liljeström and Bengt Höjer | Understanding young men's sexual health and prospects for sexual behaviour change in rural Zambia |
| 2003 |  | 4 | 297-304 | Berit Nilsson, Lars Holmgren, Birgitta Stegmayr and Göran Westman | Sense of coherence — stability over time and relation to health, disease, and psychosocial changes in a general population: A longitudinal study |
| 2003 |  | 4 | 305-311 | Karin M Henriksson, Ulf Lindblad, Bo Ågren, Peter Nilsson-Ehle and Lennart Råstam | Associations between unemployment and cardiovascular risk factors varies with the unemployment rate: The Cardiovascular Risk Factor Study in Southern Sweden (CRISS) |
| 2003 |  | 4 | 312-318 | Ellen J Amundsen and Harald Fekjær | Progression to AIDS slowed even more after the first two years with highly active antiretroviral therapy |
| 2003 |  | 4 | 319 |  | PUBLIC HEALTH CALENDAR |
| 2003 |  | 4 |  |  | Highlights for next issue |
| 2003 | 31 | 5 | 321-323 | Lars Werkö | The determinants of the research agenda |
| 2003 | 31 | 5 | 324-333 | Eleonor I M Fransson, Lars S Alfredsson, Ulf H de Faire, Anders Knutsson, A. KNUTSON and Peter J M Westerholm | Leisure time, occupational and household physical activity, and risk factors for cardiovascular disease in working men and women: the WOLF study |
| 2003 | 31 | 5 | 334-341 | Bjørn E. Holstein, Ebba Holme Hansen, Pemille Due and Anna Birna Almarsdóttir | Self-reported medicine use among 11- to 15-year-old girls and boys in Denmark 1988-1998 |
| 2003 | 31 | 5 | 342-349 | Ragnar Westerling | Decreasing gender differences in "avoidable" mortality in Sweden |
| 2003 | 31 | 5 | 350-358 | Kersti Pärna, Kaja Rahu, Krista Fischer, Helena Mussalo-Rauhamaa, Irina Zhuravleva, Triin Umbleja and Mati Rahu | Smoking and associated factors among adolescents in Tallinn, Helsinki and Moscow: a multilevel analysis |
| 2003 | 31 | 5 | 359-366 | Torbjörn Messner, Vivan Lundberg and Birgitta Stegmayr | Cardiovascular risk factor levels differ between communities of different sizes in the Northern Sweden MONICA Project |
| 2003 | 31 | 5 | 367-374 | Solveig Petersen, Erik Bergström and Christine Brulin | High prevalence of tiredness and pain in young schoolchildren |
| 2003 | 31 | 5 | 375-381 | Knut Stavem, Dag Hofoss and Olaf G. Aasland | Work characteristics and morbidity as predictors of self-perceived health status in Norwegian physicians |
| 2003 | 31 | 5 | 382-388 | Ingrid Mogren, Bernt Lindahl and Ulf Högberg | Impaired fasting glucose and impaired glucose tolerance are related to both heredity and low birth weight |
| 2003 | 31 | 5 | 389-394 | Elianne Riska | Developments in Scandinavian and American medical sociology |
| 2003 | 31 | 5 | 395-398 | Camilla Ihlebæk and Hege R. Eriksen | Are the "myths" of low back pain alive in the general Norwegian population? |
| 2003 | 31 | 5 | 399 |  | PUBLIC HEALTH CALENDAR |
| 2003 | 31 | 5 |  |  | Highlights for next issue |
| 2003 | 31 | 6 | 401-404 | Stefán Ólafsson | Welfare trends of the 1990s in Iceland |
| 2003 | 31 | 6 | 405-410 | Lotti Helström, Viveca Odlind, Catharina Zätterström, Monica Johansson, Fredrik Granath, Nestor Correia and Anders Ekbom | Abortion rate and contraceptive practices in immigrant and native women in Sweden |
| 2003 | 31 | 6 | 411-417 | Jon A. Hardie, Per S. Bakke and Odd Mørkve | Non-response bias in a postal questionnaire survey on respiratory health in the old and very old |
| 2003 | 31 | 6 | 418-427 | Amir Baigi, Benkt Högstedt, Sven-Olof Isacsson, Anders Odén and Per Herrström | Causes of death between 1911-1950 in a Swedish province with a population characterized by longevity: Effects on life expectancy |
| 2003 | 31 | 6 | 428-438 | Tiina H. Pensola and Pekka Martikainen | Effect of living conditions in the parental home and youth paths on the social class differences in mortality among women |
| 2003 | 31 | 6 | 439-443 | Ole Mygind, Tove Rønne, Anne-Lise Søe, Carsten Henrik Wachmann and Philip Ricks | Comparative intervention study among Danish daycare children: the effect on illness of time spent outdoors |
| 2003 | 31 | 6 | 444-450 | Martin Lindström, Mahnaz Moghaddassi, Kristian Bolin, Björn Lindgren and Juan Merlo | Social participation, social capital and daily tobacco smoking: a population-based multilevel analysis in Malmö, Sweden |
| 2003 | 31 | 6 | 451-459 | Jyrki Korkeila, Ville Lehtinen, Rob Bijl, Odd-Steffer Dalgard, Viviane Kovess, Antony Morgan and Hans Joachim Salize | Establishing a set of mental health indicators for Europe |
| 2003 | 31 | 6 | 460-474 | Elsy Söderberg and Kristina Alexanderson | Sickness certification practices of physicians: a review of the literature |
| 2003 | 31 | 6 | 475-477 | N. H. Antia | Health for all: an alternative strategy |
| 2003 | 31 | 6 | 478 |  | PUBLIC HEALTH CALENDAR |
| 2003 | 31 | 4 |  |  | Highlights for next issue |
| 2004 | 32 | 1 | 1-2 | Stig Wall and Urban Janlert | The World Health Report 2003: conveying new insights while refurbishing old ideas |
| 2004 | 32 | 1 | 3-5 | Sven Britton | The HIV/AIDS pandemic: at last good news |
| 2004 | 32 | 1 | 6-16 | Johan Fritzell, Magnus Nermo and Olle Lundberg | The impact of income: assessing the relationship between income and health in Sweden |
| 2004 | 32 | 1 | 17-23 | Karin Helweg-Larsen, Ashraf Hasan Abdel-Jabbar Al-Qadi, Jalal Al-Jabriri and Henrik Brønnum-Hansen | Systematic medical data collection of intentional injuries during armed conflicts: a pilot study conducted in West Bank, Palestine |
| 2004 | 32 | 1 | 24-29 | Lisbeth Sachs | The new age of the molecular family: An anthropological view on the medicalisation of kinship |
| 2004 | 32 | 1 | 30-39 | Unto Häkkinen and Jutta Järvelin | Developing the formula for state subsidies for health care in Finland |
| 2004 | 32 | 1 | 40-46 | Anders Hjern | Illicit drug abuse in second-generation immigrants: a register study in a national cohort of Swedish residents |
| 2004 | 32 | 1 | 47-52 | Kristinn Tómasson, Hólmfríður Gunnarsdóttir, Guðbjörg L. Rafnsdottir and Berglind Helgadóttir | Correlates of probable alcohol abuse among women working in nursing homes |
| 2004 | 32 | 1 | 53-59 | Marie Norredam, Allan Krasnik, Tine Moller Sorensen, Niels Keiding, Jette Joost Michaelsen and Anette Sonne Nielse | Emergency room utilization in Copenhagen: a comparison of immigrant groups and Danish-born residents |
| 2004 | 32 | 1 | 60-67 | Laidon Shapo, Joceline Pomerleau and Martin McKee | Physical Inactivity in a country in transition: a population-based survey in Tirana City, Albania |
| 2004 | 32 | 1 | 68-74 | Mona Sundh and Curt Hagquist | The importance of a minimum age law for the possibility of purchase of tobacco by adolescents: a study based on Swedish experiences |
| 2004 | 32 | 1 | 75-77 | Ulf Högberg | An "American dilemma" in Scandinavian childbirth: unmet needs in healthcare? |
| 2004 | 32 | 1 | 78 |  | PUBLIC HEALTH CALENDAR |
| 2004 | 32 | 1 | 79 |  | Acknowledgements |
| 2004 | 32 | 1 |  |  | Highlights for next issue |
| 2004 | 32 | 2 | 81-83 | Richard Madeley | Notes from a small island |
| 2004 | 32 | 2 | 84-93 | Michael Calnan, Emma Wadsworth, Margaret May, Andrew Smith and David Wainwright | Job strain, effort-reward imbalance, and stress at work: competing or complementary models? |
| 2004 | 32 | 2 | 94-101 | Margareta Leijon, Gunnel Hensing and Kristina Alexanderson | Sickness absence due to musculoskeletal diagnoses: association with occupational gender segregation |
| 2004 | 32 | 2 | 102-110 | Cristina Renzi, Ester Zantedeschi, Carlo Signorelli and the NEM Group | Voluntary HIV testing in Europe |
| 2004 | 32 | 2 | 111-117 | Linda Kærlev, Lene B. Jacobsen, Jørn Olsen and Jens Peter Bonde | Long-term sick leave and its risk factors during pregnancy among Danish hospital employees |
| 2004 | 32 | 2 | 118-129 | Anna R. Spein, Harold Sexton and Siv Kvernmo | Predictors of smoking behaviour among indigenous Sami adolescents and non-indigenous peers in North Norway |
| 2004 | 32 | 2 | 130-135 | Lars Buhl, Henrik Sælan and Tine Møller Sørensen | Adverse events due to change in organization of problem drug users' treatment? |
| 2004 | 32 | 2 | 136-143 | Yrsa Andersen Hundrup, Susanne Høidrup, Erik B. Obel and Niels Kr. Rasmussen | The validity of self-reported fractures among Danish female nurses: comparison with fractures registered in the Danish National Hospital Register |
| 2004 | 32 | 2 | 144-152 | Katarina Hamberg, Gunilla Risberg and Eva Johansson | Male and female physicians show different patterns of gender bias: A paper-case study of management of irritable bowel syndrome |
| 2004 | 32 | 2 | 153-156 | Julia F. Sollenberger and Michele Shipley | The state of scientific publishing: problems and promise |
| 2004 | 32 | 2 | 157 |  | PUBLIC HEALTH CALENDAR |
| 2004 | 32 | 2 |  |  | PUBLIC HEALTH CALENDAR |
| 2004 | 32 | 3 | 161-163 | Stephen Tollman | Establishing long-term research partnerships: aligning rhetoric and reality |
| 2004 | 32 | 3 | 164-171 | Karen Albertsen, Harald Hannerz, Vilhelm Borg and Hermann Burr | Work environment and smoking cessation over a five-year period |
| 2004 | 32 | 3 | 172-178 | Ingela Krantz, Lisbeth Sachs and Tore Nilstun | Ethics and vaccination |
| 2004 | 32 | 3 | 179-187 | Birgit Lie | The psychological and social situation of repatriated and exiled refugees: a longitudinal, comparative study |
| 2004 | 32 | 3 | 188-193 | Peter Vedsted, Henrik T Sørensen, Jørgen N Nielsen and Frede Olesen | Variation in proportion of frequent attenders between Danish general practices |
| 2004 | 32 | 3 | 194-202 | Kontie M. Moussa, Martin Lindström and P-O Östergren | Socioeconomic and demographic differences in exposure to environmental tobacco smoke at work: the Scania Public Health Survey 2000 |
| 2004 | 32 | 3 | 203-209 | Reija Klemetti, Mika Gissler and Elina Hemminki | Equity in the use of IVF in Finland in the late 1990s |
| 2004 | 32 | 3 | 210-216 | Hanne Kronborg and Michael væth | The influence of psychosocial factors on the duration of breastfeeding |
| 2004 | 32 | 3 | 217-223 | Christiaan W.S. Monden | Socioeconomic health inequalities in Latvia: a cross-sectional study |
| 2004 | 32 | 3 | 224-229 | Klaus Hoeyer, Bert-Ove Olofsson, Tom Mjörndal and Niels Lynöe | Informed consent and biobanks: a population-based study of attitudes towards tissue donation for genetic research |
| 2004 | 32 | 3 | 230-231 | Jeffrey V. Lazarus | Response to "The HIV/AIDS pandemic: at last good news" editorial by Sven Britton Is the news on HIV/AIDS good? |
| 2004 | 32 | 3 | 232-233 | Sven Britton | The HIV pandemic: darkness prevails but there is light |
| 2004 | 32 | 3 | 234-235 |  | Public health websites |
| 2004 | 32 | 3 | 236-237 |  | PUBLIC HEALTH CALENDAR |
| 2004 | 32 | 4 | 241-242 | Robert Beaglehole | Challenging the public health workforce |
| 2004 | 32 | 4 | 243-249 | Martin Lindström and Elin Axén | Social capital, the miniaturization of community and assessment of patient satisfaction in primary healthcare: a population-based study |
| 2004 | 32 | 4 | 250-256 | Christina Schnohr, Lise højbjerre, Mette Riegels, Luise Ledet, Tine Larsen, Kirsten Schultz-Larsen, Liselotte Petersen, Eva Prescott and Morten Grønbæk | Does educational level influence the effects of smoking, alcohol, physical activity, and obesity on mortality? A prospective population study |
| 2004 | 32 | 4 | 257-263 | Örjan B Ekblom, Kristjan Oddsson and Björn T Ekblom | Prevalence and regional differences in overweight in 2001 and trends in BMI distribution in Swedish children from 1987 to 2001 |
| 2004 | 32 | 4 | 264-271 | Anne Kjersti Daltveit, Stein Emil Vollset, Britt Lande and Henriette Øien | Changes in knowledge and attitudes of folate, and use of dietary supplements among women of reproductive age in Norway 1998-2000 |
| 2004 | 32 | 4 | 272-278 | Karin Borg, Gunnel Hensing and Kristina Alexanderson | Risk factors for disability pension over 11 years in a cohort of young persons initially sick-listed with low back, neck, or shoulder diagnoses |
| 2004 | 32 | 4 | 279-286 | Ole Raaschou-Nielsen, Josephine Obel, Susanne Dalton, Anne Tjønneland and Johnni Hansen | Socioeconomic status and risk of childhood leukaemia in Denmark |
| 2004 | 32 | 4 | 287-295 | Jette J. Michaelsen, Allan Krasnik, Anette S. Nielsen, Marie Norredam and Ana Maria Torres | Health professionals' knowledge, attitudes, and experiences in relation to immigrant patients: a questionnaire study at a Danish hospital |
| 2004 | 32 | 4 | 296-302 | Nina Støvring, Kirsten Avlund, Kirsten Schultz-Larsen and Marianne Schroll | The cumulative effect of smoking at age 50, 60, and 70 on functional ability at age 75 |
| 2004 | 32 | 4 | 303-309 | Miika Hemelahti, Urho M. Kujala, Jaakko Kaprio and Urho M. Kujula | Stability and change of volume and intensity of physical activity as predictors of hypertension |
| 2004 | 32 | 4 | 310-316 | Pekka Ikäheimo, Tuili Tuuponen, Sirpa Hartikainen, Jorma Kiuttu and Timo Klaukka | Achievements and shortcomings of Finnish asthma care |
| 2004 | 32 | 4 |  |  | PUBLIC HEALTH CALENDAR |
| 2004 | 32 | 4 |  |  | Highlights for next issue |
| 2004 | 32 | 5 | 321-323 | Kristina Alexanderson and Gunnel Hensing | More and better research needed on sickness absence |
| 2004 | 32 | 5 | 324-332 | Daniel P Novak, Lars Lindholm, Monica Jonsson and Roger B Karlsson | A Swedish cost-effectiveness analysis of community-based Chlamydia trachomatis PCR testing of postal urine specimens obtained at home |
| 2004 | 32 | 5 | 333-339 | Ann-Charlotte Grahn Kronhed, Inger Knutsson, Owe Löfman, Toomas Timpka, Göran Toss and Margareta Möller | Is calcaneal stiffness more sensitive to physical activity than forearm bone mineral density? A population-based study of persons aged 20-79 years |
| 2004 | 32 | 5 | 340-348 | Sturla Gjesdal, Rolv T. Lie and John Gunnar Maelan | Variations in the risk of disability pension in Norway 1970-99: A gender-specific age-period-cohort analysis |
| 2004 | 32 | 5 | 349-355 | Trinidad Caldera, Andrés Herrera, Ellinor Salander Renberg and Gunnar Kullgren | Parasuicide in a low-income country: results from three-year hospital surveillance in Nicaragua |
| 2004 | 32 | 5 | 356-360 | Eva Theander, Gun-Britt Jarnlo, Ewald Ornstein and Magnus K. Karlsson | Activities of daily living decrease similarly in hospital-treated patients with a hip fracture or a vertebral fracture: a one-year prospective study in 151 patients |
| 2004 | 32 | 5 | 361-367 | Sindre Hoel, Bjørn Magne Eriksen, Hans-Johan Breidablik and Eivind Meland | Adolescent alcohol use, psychological health, and social integration |
| 2004 | 32 | 5 | 368-373 | Martin Hyde, Jan Hagberg, Gabriel Oxenstierna, Töres Theorell and Hugo Westerlund | Bridges, pathways and valleys: labour market position and risk of hospitalization in a Swedish sample aged 55-63 |
| 2004 | 32 | 5 | 374-380 | Ramune Kalediene, Skirmante Starkuviene and Jadvyga Petrauskiene | Mortality from external causes in Lithuania: looking for critical points in time and place |
| 2004 | 32 | 5 | 381-389 | Annett Arntzen and Anne Marie Nybo Andersen | Social determinants for infant mortality in the Nordic countries, 1980-2001 |
| 2004 | 32 | 5 | 390-395 | Peter Bjerregaard, T Kue Young, Eric Dewailly and Sven OE Ebbesson | Indigenous health in the Arctic: an overview of the circumpolar Inuit population |
| 2004 | 32 | 5 | 396-398 | Lars Cernerud and Henny Olsson | Humour seen from a public health perspective |
| 2004 | 32 | 5 | 399 |  | PUBLIC HEALTH CALENDAR |
| 2004 | 32 | 6 | 401 |  | EDITORIAL COMMENTARY |
| 2004 | 32 | 6 | 401-402 | Urban Janlert | The population as patient: on public health work |
| 2004 | 32 | 6 | 403-410 | Kirsten Ekerholt and Astrid Bergland | The first encounter with Norwegian psychomotor physiotherapy: patients' experiences, a basis for knowledge |
| 2004 | 32 | 6 | 411-418 | Alan J. Flisher, Holan Liang, Ria Laubscher and Carl F. Lombard | Suicide trends in South Africa, 1968-90 |
| 2004 | 32 | 6 | 419-425 | Rikke Lund, Kirsten Avlund, Jens Modvig, Pernille Due and Bjørn E. Holstein | Development in self-rated health among older people as determinant of social relations |
| 2004 | 32 | 6 | 426-434 | Ari Väänänen, Raija Kalimo, Salla Toppinen-Tanner, Pertti Mutanen, José Maria Peiró, Mika Kivimäki and Jussi Vahtera | Role clarity, fairness, and organizational climate as predictors of sickness absence: A prospective study in the private sector |
| 2004 | 32 | 6 | 435-441 | Hella Danø, Rune Jacobsen, Kasper Daniel Hansen, Jørn Korsbø Petersen and Elsebeth Lynge | Use of census data for construction of fertility history for Danish women |
| 2004 | 32 | 6 | 442-449 | Elisabeth Dejin-Karlsson and Per-Olof Östergren | Country of origin, social support and the risk of small for gestational age birth |
| 2004 | 32 | 6 | 450-455 | Mikael Nordenmark | Balancing work and family demands: Do increasing demands increase strain? A longitudinal study |
| 2004 | 32 | 6 | 456-463 | Alec S Ostry, Ruth Hershler, Lisa Chen and Clyde Hertzman | A longitudinal study comparing the effort–reward imbalance and demand–control models using objective measures of physician utilization |
| 2004 | 32 | 6 | 464-471 | Magnus A. Björkgren, Brant E. Fries, Unto Häkkinen and Mats Brommels | Case-mix adjustment and efficiency measurement |
| 2004 | 32 | 6 | 472-475 | Niels Lynöe, Mikael Sandlund, Lars Jacobsson, Gunnar Nordberg and Taiyi Jin | Informed consent in China: quality of information provided to participants in a research project |
| 2004 | 32 | 6 | 476-478 | Jeffrey V. Lazarus, Gunilla Essner and Jerker Liljestrand | ICPD@ten: is reproductive health under fire? |
| 2004 | 32 | 6 | 479 |  | PUBLIC HEALTH CALENDAR |
| 2004 | 32 | 6 |  |  | Highlights for next issue |
| 2005 | 33 | 1 | 1-3 | CAMILLA STOLTENBERG | Merging genetics and epidemiology: What is in it for public health? |
| 2005 | 33 | 1 | 4-10 | EVA T. JACOBSEN, SUSANNE R. RASMUSSEN, MAJBRITT CHRISTENSEN, MARIANNE ENGBERG and TORSTEN LAURITZEN | Perspectives on lifestyle intervention: The views of general practitioners who have taken part in a health promotion study |
| 2005 | 33 | 1 | 11-18 | LENA OLDFORS ENGSTRÖM and BIRGITTA ÖBERG | Patient adherence in an individualized rehabilitation programme: A clinical follow-up |
| 2005 | 33 | 1 | 19-25 | KARIN ENGSTRÖM, JOHAN HALLQVIST, JETTE MÖLLER and LUCIE LAFLAMME | Do episodes of peer victimization trigger physical injury? A case-crossover study of Swedish school children |
| 2005 | 33 | 1 | 26-34 | CATARINA A. CANIVET, PER-OLOF ÖSTERGREN, ANNE-SOFIE ROSÉN, IRÉNE L. JAKOBSSON and BARBRO M. HAGANDER | Infantile colic and the role of trait anxiety during pregnancy in relation to psychosocial and socioeconomic factors |
| 2005 | 33 | 1 | 35-41 | PÄIVI-LEENA K. HONKINEN, SAKARI B. SUOMINEN, RAILI S. VÄLIMAA, HANS Y. HELENIUS and PÄIVI T. RAUTAVA | Factors associated with perceived health among 12-year-old school children. Relevance of physical exercise and sense of coherence |
| 2005 | 33 | 1 | 42-49 | SIMEON KEDIKOGLOU, MARIA BELECHRI, XANTHI DEDOUKOU, THEMIS SPYRIDOPOULOS, DELIA-MARINA ALEXE, M.A. DELIA, EVANTHIA PAPPA, ASIMOULA STAMOU and ELENI PETRIDOU | A maternity hospital-based infant car-restraint loan scheme: Public health and economic evaluation of an intervention for the reduction of road traffic injuries |
| 2005 | 33 | 1 | 50-56 | NIKLAS RUDHOLM | Pharmaceutical insurance and the demand for prescription Pharmaceuticals in Västerbotten, Sweden |
| 2005 | 33 | 1 | 57-64 | LENNART NATHELL | Effects on sick leave of an inpatient rehabilitation programme for asthmatics in a randomized trial |
| 2005 | 33 | 1 | 65-71 | MARIT D. GISSELMANN | Education, infant mortality, and low birth weight in Sweden 1973-1990: Emergence of the low birth weight paradox |
| 2005 | 33 | 1 | 72-76 | LIV GRØTVEDT and KNUT STAVEM | Association between age, gender and reasons for smoking cessation |
| 2005 | 33 | 1 | 77 |  | PUBLIC HEALTH CALENDAR |
| 2005 | 33 | 1 |  |  | Highlights for next issue |
| 2005 | 33 | 2 | 81-83 | JØRN OLSEN | Public health epidemiology – what next? |
| 2005 | 33 | 2 | 84-90 | MIKAEL STATTIN and BENGT JÄRVHOLM | Occupation, work environment, and disability pension: A prospective study of construction workers |
| 2005 | 33 | 2 | 91-98 | BETTY J. PETTERSEN and ROAR JOHNSEN | More physicians in public health: Less public health work? |
| 2005 | 33 | 2 | 99-106 | LENE FROST ANDERSEN, INGER THERESE L. LILLEGAARD, NINA ØVERBY, LESLIE LYTLE, KNUT-INGE KLEPP and LARS JOHANSSON | Overweight and obesity among Norwegian schoolchildren: Changes from 1993 to 2000 |
| 2005 | 33 | 2 | 107-113 | ANNA HERTTING, KERSTIN NILSSON, TÖRES THEORELL and ULLABETH SÄTTERLUND LARSSON | Assistant nurses in the Swedish healthcare sector during the 1990s: A hard-hit occupational group with a tough job |
| 2005 | 33 | 2 | 114-122 | LARS ERIK KJEKSHUS | Primary health care and hospital interactions: Effects for hospital length of stay |
| 2005 | 33 | 2 | 123-130 | ANNERS LERDAL, ASTRID K. WAHL, TONE RUSTØEN, BERIT R. HANESTAD and TORBJØRN MOUM | Fatigue in the general population: A translation and test of the psychometric properties of the Norwegian version of the fatigue severity scale |
| 2005 | 33 | 2 | 131-137 | ÖRJAN HEMSTRÖM | Does high income buffer the association between adverse working conditions and ill health? |
| 2005 | 33 | 2 | 138-145 | CHARLOTTA PISINGER, JØRGEN VESTBO, KNUT BORCH-JOHNSEN, TROELS THOMSEN and TORBEN JØRGENSEN | Acceptance of the smoking cessation intervention in a large population-based study: The Inter99 study |
| 2005 | 33 | 2 | 146-150 | RAINER SAUERBORN, ADJIMA GBANGOU, A.A. GBANGOU, HENGJIN DONG, H.H. DONG, JUDE M. PRZYBORSKI and MICHAEL LANZER | Willingness to pay for hypothetical malaria vaccines in rural Burkina Faso |
| 2005 | 33 | 2 | 151-155 | MÅNS ROSÉN and BENGT HAGLUND | From healthy survivors to sick survivors – implications for the twenty-first century |
| 2005 | 33 | 2 | 156-158 | MARTIN LINDSTRÖM | Price restrictions and other restrictions on alcohol availability in Denmark and Sweden: A historical perspective with implications for the current debate |
| 2005 | 33 | 2 | 159 |  | PUBLIC HEALTH CALENDAR |
| 2005 | 33 | 2 |  |  | Highlights for next issue |
| 2005 | 33 | 3 | 161 | Sakari Suominen | Note from the owners of Scand J Public Health |
| 2005 | 33 | 3 | 162-163 | JOUNI T. TUOMISTO and JUHA PEKKANEN | Assessing environmental health risks or net health benefits? |
| 2005 | 33 | 3 | 164-165 | CHRISTER HOGSTEDT and ROBERT ERIKSON | Inventory and evaluation of Swedish public health research |
| 2005 | 33 | 3 | 166-174 | BJARTE SANNE, STEFFEN TORP, ARNSTEIN MYKLETUN and ALV A. DAHL | The Swedish Demand-Control-Support Questionnaire (DCSQ): Factor structure, item analyses, and internal consistency in a large population |
| 2005 | 33 | 3 | 175-182 | ELLIS JANZON, GUNNAR ENGSTRÖM, MARTIN LINDSTRÖM, GÖRAN BERGLUND, BO HEDBLAD and LARS JANZON | Who are the "quitters"? a cross-sectional study of circumstances associated with women giving up smoking |
| 2005 | 33 | 3 | 183-189 | ULRIKA AF SILLÉN, JAN-ÅKE NILSSON, NILS-OVE MÅNSSON and PETER M. NILSSON | Self-rated health in relation to age and gender: Influence on mortality risk in the Malmö Preventive Project |
| 2005 | 33 | 3 | 190-196 | ABDONAS TAMOSIUNAS, REGINA REKLAITIENE, RICARDAS RADISAUSKAS and KRISTINA JURENIENE | Prognosis of risk factors and trends in mortality from external causes among middle-aged men in Lithuania |
| 2005 | 33 | 3 | 197-202 | ANNE METTE SKOVGAARD, ELSE MARIE OLSEN, TINE HOUMANN, EVA CHRISTIANSEN, VIBEKE SAMBERG, ANNE LICHTENBERG and TORBEN JØRGENSEN | The Copenhagen County child cohort: Design of a longitudinal study of child mental health |
| 2005 | 33 | 3 | 203-207 | LOTTE HVAS, SUSANNE REVENTLOW, HANS L. JENSEN and KIRSTI MALTERUD | Awareness of risk of osteoporosis may cause uncertainty and worry in menopausal women |
| 2005 | 33 | 3 | 208-214 | CHARLOTTE BJÖRKENSTAM, ANNIKA EDBERG, SHIVA AYOUBI and MÅNS ROSÉN | Are cancer patients at higher suicide risk than the general population? A nationwide register study in Sweden from 1965 to 1999 |
| 2005 | 33 | 3 | 215-221 | ØYVIND NÆSS, BJØRGULF CLAUSSEN, DAG S. THELLE and GEORGE DAVEY SMITH | Four indicators of socioeconomic position: relative ranking across causes of death |
| 2005 | 33 | 3 | 222-227 | JEANNE D. JOHANSEN, ELSE SMITH, KNUD JUEL and NILS ROSDAHL | The AIDS epidemic in the city of Copenhagen, Denmark: Potential years of life lost and impact on life expectancy |
| 2005 | 33 | 3 | 228-232 | HÅKAN BERGH, AMIR BAIGI and BERTIL MARKLUND | Consultations for injuries by frequent attenders are found to be medically appropriate from general practitioners' perspective |
| 2005 | 33 | 3 | 233-235 | STEFAN HANSON | Is HIV control in sub-Saharan Africa losing focus?: The need for simplified, prioritized prevention strategies |
| 2005 | 33 | 3 | 236-238 | ANNA-KARIN HURTIG and MIGUEL SAN SEBASTIÁN | The People's Health Movement: What is it and should we care? |
| 2005 | 33 | 3 | 239 |  | PUBLIC HEALTH CALENDAR |
| 2005 | 33 | 3 |  |  | Highlights for next issue |
| 2005 | 33 | 4 | 241-242 | PETER BJERREGAARD | Development of a public health programme in Greenland |
| 2005 | 33 | 4 | 243 |  | EDITORIAL COMMENTARY |
| 2005 | 33 | 4 | 244-249 | PETER SCHNOHR, MORTEN GRØNBÆK, LISELOTTE PETERSEN, HANS OLE HEIN and THORKILD IA SØRENSEN | Physical activity in leisure-time and risk of cancer: 14-year follow-up of 28,000 Danish men and women |
| 2005 | 33 | 4 | 250-260 | E. ROOS, E. LAHELMA, P. SAASTAMOINEN and J.-I. ELSTAD | The association of employment status and family status with health among women and men in four Nordic countries |
| 2005 | 33 | 4 | 261-267 | MAIGUN EDHBORG, MALIN FRIBERG, WENDELA LUNDH and ANN-MARIE WIDSTRÖM | "Struggling with life": Narratives from women with signs of postpartum depression |
| 2005 | 33 | 4 | 268-275 | KNUT HAGEN, JOHN-ANKER ZWART, SVEN SVEBAK, GUNNAR BOVIM and LARS JACOB STOVNER | Low socioeconomic status is associated with chronic musculoskeletal complaints among 46,901 adults in Norway |
| 2005 | 33 | 4 | 276-284 | MARTIN LINDSTRÖM and KRISTINA SUNDQUIST | The impact of country of birth and time in Sweden on overweight and obesity: A population-based study |
| 2005 | 33 | 4 | 285-291 | LENA JÖRGENSEN, NIKLAS HAMMAR, JAAKKO KAPRIO, MARKKU KOSKENVUO, ÅKE SVENSSON and Å SVENSON | Migration and smoking: An epidemiological study of Finnish twins in Sweden |
| 2005 | 33 | 4 | 292-299 | INGRID POULSEN, BJØRN HESSELBO, INGE PIETERSEN and MARIANNE SCHROLL | Implementation of functional assessment scales in geriatric practice: A feasibility study |
| 2005 | 33 | 4 | 300-306 | INGRID M. MOGREN | Previous physical activity decreases the risk of low back pain and pelvic pain during pregnancy |
| 2005 | 33 | 4 | 307-313 | VILLE M. MATTILA, JARI PARKKARI, TOMI LINTONEN, PEKKA KANNUS and ARJA RIMPELÄ | Occurrence of violence and violence-related injuries among 12-18 year-old Finns |
| 2005 | 33 | 4 | 314-320 | ELSY SÖDERBERG and KRISTINA ALEXANDERSON | Sickness certificates as a basis for decisions regarding entitlement to sickness insurance benefits |
| 2005 | 33 | 4 | 321-324 | BIRGITTA STEGMAYR, MATS ELIASSON and BRAD RODU | The Decline of Smoking in Northern Sweden |
| 2005 | 33 | 4 | 325 |  | PUBLIC HEALTH CALENDAR |
| 2005 | 33 | 4 |  |  | Highlights for next issue |
| 2005 | 33 | 5 | 329-333 |  | Health Research for Development: Making health research work ... for everyone |
| 2005 | 33 | 5 | 334-342 | ANN-CHRISTIN SOLLERHED, GÖRAN EJLERTSSON and ERWIN APITZSCH | Predictors of strong sense of coherence and positive attitudes to physical education in adolescents |
| 2005 | 33 | 5 | 343-352 | PIA M. JOHANSSON, PER E. TILLGREN, KARIN A. GULDBRANDSSON and LARS A. LINDHOLM | A model for cost-effectiveness analyses of smoking cessation interventions applied to a Quit-and-Win contest for mothers of small children |
| 2005 | 33 | 5 | 353-359 | ANETTE EKSTRÖM, ANN-SOFI MATTHIESEN, ANN- MARIE WIDSTRÖM and EVA NISSEN | Breastfeeding attitudes among counselling health professionals |
| 2005 | 33 | 5 | 360-369 | RØNNAUG AA. FAGERLI, MARIANNE E. LIEN, GRETE S. BOTTEN and MARGARETA WANDEL | Role dilemmas among health-workers in cross-cultural patient encounters around dietary advice |
| 2005 | 33 | 5 | 370-375 | EVA VINGÅRD, PER LINDBERG, MALIN JOSEPHSON, MARGARETHA VOSS, BODIL HEIJBEL, LARS ALFREDSSON, STEFAN STARK and ÅKE NYGREN | Long-term sick-listing among women in the public sector and its associations with age, social situation, lifestyle, and work factors: A three-year follow-up study |
| 2005 | 33 | 5 | 373-383 | ANN-DORTHE O. ZWISLER, ULLA I. TFÆDEN, JØRGEN VIDEBÆK and METTE MADSEN | Cardiac rehabilitation services in Denmark: Still room for expansion |
| 2005 | 33 | 5 | 384-391 | JENNY RUNDBERG, JONAS LIDFELDT, CHRISTINA NERBRAND, GÖRAN SAMSIOE, ANDERS ROMELSJÖ and AGNETA ÖJEHAGEN | Few middle-aged women with severe mental symptoms use psychotropic drugs: The Women's Health in Lund Area (WHILA) Study |
| 2005 | 33 | 5 | 392-400 | EVA THEANDER and ANNA-KARIN EDBERG | Preventive home visits to older people in Southern Sweden |
| 2005 | 33 | 5 | 401-406 | CAMILLA IHLEBÆK and HEGE R. ERIKSEN | Myths and perceptions of back pain in the Norwegian population, before and after the introduction of guidelines for acute back pain |
| 2005 | 33 | 5 | 407 |  | PUBLIC HEALTH CALENDAR |
| 2005 | 33 | 5 |  |  | Highlights for next issue |
| 2005 | 33 | 6 | 409-411 | ULF HÖGBERG | The World Health Report 2005: "Make every mother and child count" - including Africans |
| 2005 | 33 | 6 | 412-423 | PETER M. NILSSON, JAN-ÅKE NILSSON, PER-OLOF ÖSTERGREN and GÖRAN BERGLUND | Social mobility, marital status, and mortality risk in an adult life course perspective: The Malmö Preventive Project |
| 2005 | 33 | 6 | 424-431 | ANETTE EKSTRÖM, ANN-MARIE WIDSTRÖM and EVA NISSEN | Process-oriented training in breastfeeding alters attitudes to breastfeeding in health professionals |
| 2005 | 33 | 6 | 432-438 | LOUISE STJERNBERG and JOHAN BERGLUND | Tick prevention in a population living in a highly endemic area |
| 2005 | 33 | 6 | 439-446 | LINDA MIDTTUN and PÅL E. MARTINUSSEN | Hospital waiting time in Norway: What is the role of organizational change? |
| 2005 | 33 | 6 | 447-454 | KRISTINA SUNDQUIST, SVEN-ERIK JOHANSSON, JAN QVIST and JAN SUNDQUIST | Does occupational social class predict coronary heart disease after retirement? A 12-year follow-up study in Sweden |
| 2005 | 33 | 6 | 455-463 | GABRIEL OXENSTIERNA, JANE FERRIE, MARTIN HYDE, HUGO WESTERLUND and TÖRES THEORELL | Dual source support and control at work in relation to poor health |
| 2005 | 33 | 6 | 464-471 | SYED MONIRUZZAMAN and RAGNAR ANDERSSON | Age- and sex-specific analysis of homicide mortality as a function of economic development: A cross-national comparison |
| 2005 | 33 | 6 | 472-477 | MALIN KARK and FINN RASMUSSEN | Growing social inequalities in the occurrence of overweight and obesity among young men in Sweden |
| 2005 | 33 | 6 | 478-486 | MARIA NYHOLM, JUAN MERLO, LENNART RÅSTAM and ULF LINDBLAD | Overweight and all-cause mortality in a Swedish rural population: Skaraborg Hypertension and Diabetes Project |
| 2005 | 33 | 6 | 487 |  | PUBLIC HEALTH CALENDAR |
| 2005 | 33 | 6 |  |  | Highlights for next issue (1-2006) |
| 2006 | 34 | 1 | 1-4 | MARY C. ELLSBERG | Violence against women: A global public health crisis |
| 2006 | 34 | 1 | 5-10 | BERIT ROSTAD, BERIT SCHEI and JOHANNE SUNDBY | Fertility in Norwegian women: Results from a population-based health survey |
| 2006 | 34 | 1 | 11-16 | SIDSEL GRAFF-IVERSEN, NIKLAS HAMMAR, DAG S. THELLE and SERENA TONSTAD | Use of oral contraceptives and mortality during 14 years' follow-up of Norwegian women |
| 2006 | 34 | 1 | 17-25 | LOTTA DELLVE, CATARINA KARLBERG, PETER ALLEBECK, BIRGITTA HERLOFF and MATS HAGBERG | Macro-organizational factors, the incidence of work disability, and work ability among the total workforce of home care workers in Sweden |
| 2006 | 34 | 1 | 26-31 | PETER BYASS, EDWARD FOTTRELL, DAO LAN HUONG, YEMANE BERHANE, TUMANI CORRAH, KATHLEEN KAHN, LULU MUHE and DO DUC VAN | Refining a probabilistic model for interpreting verbal autopsy data |
| 2006 | 34 | 1 | 32-40 | ANETTE JOHANSEN, SØREN RASMUSSEN and METTE MADSEN | Health behaviour among adolescents in Denmark: Influence of school class and individual risk factors |
| 2006 | 34 | 1 | 41-48 | KRISTINA JOHNELL, NILS-OVE MÅNSSON, JAN SUNDQUIST, ARNE MELANDER, GÖRAN BLENNOW and JUAN MERLO | Neighborhood social participation, use of anxiolytic-hypnotic drugs, and women's propensity for disability pension: a multilevel analysis |
| 2006 | 34 | 1 | 49-58 | MARIANNE BORRITZ, REINER RUGULIES, JAKOB B. BJORNER, EBBE VILLADSEN, OLE A. MIKKELSEN and TAGE S. KRISTENSEN | Burnout among employees in human service work: design and baseline findings of the PUMA study |
| 2006 | 34 | 1 | 59-68 | ELAINE SJÖGREN, MARGARETA KRISTENSON and THE LINQUESTGROUP | Can gender differences in psychosocial factors be explained by socioeconomic status? |
| 2006 | 34 | 1 | 69-75 | MARTIN LINDSTRÖM | Social capital and lack of belief in the possibility to influence one's own health: A population-based study |
| 2006 | 34 | 1 | 76-82 | MONICA ÅBERG YNGWE, OLLE LUNDBERG and BO BURSTRÖM | On the importance of internalized consumption norms for ill health |
| 2006 | 34 | 1 | 83-91 | MARINA CLARKE, JUDY DICK and LENNART BOGG | Cost-effectiveness analysis of an alternative tuberculosis management strategy for permanent farm dwellers in South Africa amidst health service contraction |
| 2006 | 34 | 1 | 92-99 | JEFFREY V. LAZARUS, HIMEDAN MOHAMMED HIMEDAN, LISE ROSENDAL ØSTERGAARD and JERKER LILJESTRAND | HIV/AIDS knowledge and condom use among Somali and Sudanese immigrants in Denmark |
| 2006 | 34 | 1 | 100-109 | NITIN GARG and ADNAN A. HYDER | Road traffic injuries in India: A review of the literature |
| 2006 | 34 | 1 | 110 |  | PUBLIC HEALTH CALENDAR |
| 2006 | 34 | 1 | 111 |  | PUBLIC HEALTH CALENDAR |
| 2006 | 34 | 2 | 113-115 | TÖRES THEORELL | New directions for psychosocial work environment research |
| 2006 | 34 | 2 | 116-123 | TIINA HETEMAA, KRISTIINA MANDERBACKA, ANTTI REUNANEN, SEPPO KOSKINEN and ILMO KESKIMÄKI | Socioeconomic inequities in invasive cardiac procedures among patients with incident angina pectoris or myocardial infarction |
| 2006 | 34 | 2 | 124-131 | MARGARETA LARSSON, KARIN EURENIUS, RAGNAR WESTERLING and TANJA TYDÉN | Evaluation of a sexual education intervention among Swedish high school students |
| 2006 | 34 | 2 | 132-139 | FRIDA CARLSSON, JUAN MERLO, MARTIN LINDSTRÖM, PER-OLOF ÖSTERGREN and THOR LITHMAN | Representativity of a postal public health questionnaire survey in Sweden, with special reference to ethnic differences in participation |
| 2006 | 34 | 2 | 140-149 | MARIA EMMELIN, ANNE N. NAFZIGER, HANS STENLUND, LARS WEINEHALL and STIG WALL | Cardiovascular risk factor burden has a stronger association with self-rated poor health in adults in the US than in Sweden, especially for the lower educated |
| 2006 | 34 | 2 | 150-158 | LEIF EDVARD AARØ, ALAN J. FLISHER, SYLVIA KAAYA, HANS ONYA, MINOU FUGLESANG, KNUT-INGE KLEPP and HERMAN SCHAALMA | Promoting sexual and reproductive health in early adolescence in South Africa and Tanzania: Development of a theory- and evidence-based intervention programme |
| 2006 | 34 | 2 | 159-167 | ANNIKA M. H. ÅHS and RAGNAR WESTERLING | Mortality in relation to employment status during different levels of unemployment |
| 2006 | 34 | 2 | 168-174 | JUKKA MARTTILA and RITVA NUPPONEN | Assessing attitude: The case of health-enhancing physical activity |
| 2006 | 34 | 2 | 175-181 | ANNIKA S. K. FORSSÉN and GUNILLA CARLSTEDT | "It's heavenly to be alone!": A room of one's own as a health-promoting resource for women. Results from a qualitative study |
| 2006 | 34 | 2 | 182-189 | MARCUS WESTIN and RAGNAR WESTERLING | Health and healthcare utilization among single mothers and single fathers in Sweden |
| 2006 | 34 | 2 | 190-198 | KAROLIINA HARKONMÄKI, EERO LAHELMA, PEKKA MARTIKAINEN, OSSI RAHKONEN and KARRI SILVENTOINEN | Mental health functioning (SF-36) and intentions to retire early among ageing municipal employees: The Helsinki Health Study |
| 2006 | 34 | 2 | 199-208 | NAWI NG, HOANG VAN MINH, FIKRU TESFAYE, RUTH BONITA, PETER BYASS, HANS STENLUND, LARS WEINEHALL and STIG WALL | Combining risk factors and demographic surveillance: Potentials of WHO STEPS and INDEPTH methodologies for assessing epidemiological transition |
| 2006 | 34 | 2 | 209-216 | SADIQ MOHAMMAD ALI and MARTIN LINDSTRÖM | Psychosocial work conditions, unemployment, and leisure-time physical activity: A population-based study |
| 2006 | 34 | 2 | 217-221 | YULIA SUNGUROVA, SVEN-ERIK JOHANSSON and JAN SUNDQUIST | East–west health divide and east–west migration: Self-reported health of immigrants from Eastern Europe and the former Soviet Union in Sweden |
| 2006 | 34 | 2 | 222 |  | PUBLIC HEALTH CALENDAR |
| 2006 | 34 | 3 | 225-227 | DAVID MCCOY, MIKE ROWSON and DAVID SANDERS | The Global Health Watch: A global health report with a difference |
| 2006 | 34 | 3 | 228-237 | CATHERINE CUBBIN, KRISTINA SUNDQUIST, HELENA AHLÉN, SVEN-ERIK JOHANSSON, MARILYN A. WINKLEBY and JAN SUNDQUIST | Neighborhood deprivation and cardiovascular disease risk factors: Protective and harmful effects |
| 2006 | 34 | 3 | 238-246 | GUNILLA KRANTZ and ULF LUNDBERG | Workload, work stress, and sickness absence in Swedish male and female white-collar employees |
| 2006 | 34 | 3 | 247-253 | ULRICA VON THIELE, PETRA LINDFORS and ULF LUNDBERG | Evaluating different measures of sickness absence with respect to work characteristics |
| 2006 | 34 | 3 | 254-261 | JANUS L. THOMSEN, BO KARLSMOSE, ERIK T. PARNER, ANE M. THULSTRUP, TORSTEN LAURITZEN and MARIANNE ENGBERG | Secondary healthcare contacts after multiphasic preventive health screening: A randomized trial |
| 2006 | 34 | 3 | 262-271 | RUNAR VILHJALMSSON and GUDRUN KRISTJANSDOTTIR | Sociodemographic variations in parental role strain: Results from a national general population survey |
| 2006 | 34 | 3 | 272-278 | WILLY URASSA, SYLVIA KAAYA, DAVIS MWAKAGILE, MEGAN O'BRIEN, GRETCHEN ANTELMAN, DAVID HUNTER, WAFAIE FAWZI and GERNARD MSAMANGA | Evidence of a substantial decline in prevalence of HIV-1 infection among pregnant women: Data from 1995 to 2003 in Dar es Salaam, Tanzania |
| 2006 | 34 | 3 | 279-286 | LISBETH FÄRNKVIST and LARS WEINEHALL | Assessment of intervention intensity: Experiences from a small-scale Swedish cardiovascular disease prevention programme |
| 2006 | 34 | 3 | 287-294 | GÖRAN HENRIKSSON, PETER ALLEBECK, GUNILLA RINGBÄCK WEITOFT and DAG THELLE | Income distribution and mortality: Implications from a comparison of individual-level analysis and multilevel analysis with Swedish data |
| 2006 | 34 | 3 | 295-303 | CATRINE RYDSTRÖM and SVEN TÖRNBERG | Cervical cancer incidence and mortality in the best and worst of worlds |
| 2006 | 34 | 3 | 304-311 | FREDRIK BERGGREN and PAUL NYSTEDT | Changes in alcohol consumption: An analysis of self-reported use of alcohol in a Swedish national sample 1988-89 and 1996-97 |
| 2006 | 34 | 3 | 312-319 | GUNNAR TELLNES, JOHAN LUND, LEIV SANDVIK, ELISE KLOUMAN and BØRGE YTTERSTAD | Long-term effects of community-based injury prevention on the island of Værøy in Norway: A 20-year follow up |
| 2006 | 34 | 3 | 320-326 | SUSANNE REVENTLOW and HANNE BANG | Brittle bones: Ageing or threat of disease Exploring women's cultural models of osteoporosis |
| 2006 | 34 | 3 | 327-331 | TIIA AINLA, TOOMAS MARANDI, REIN TEESALU, ALEKSEI BABURIN, MÄRT ELMET, ANITA LIIVER, MARGUS PEEBA and JÜRI VOITK | Diagnosis and treatment of acute myocardial infarction in tertiary and secondary care hospitals in Estonia |
| 2006 | 34 | 3 | 332 | INGVAR KARLBERG | Comments on Midttun and Martinussen: "Hospital waiting time in Norway: What is the role of organizational change?" |
| 2006 | 34 | 3 | 333 | LINDA MIDTTUN and PÅL MARTINUSSEN | Response to Karlberg's comments on "Hospital waiting time in Norway: What is the role of organizational change?" |
| 2006 | 34 | 3 | 334 |  | PUBLIC HEALTH CALENDAR |
| 2006 | 34 | 4 | 337-341 | STIG WALL, MARIA EMMELIN, URBAN JANLERT, LENA MUSTONEN and BARBRO SKOG | Who submits to and publishes in this journal? A peer-review study of 772 manuscripts 2000-2004 |
| 2006 | 34 | 4 | 342-345 | LISBETH SACHS | Effects of magic come in from the cold: Pictorial evidence of placebo effect |
| 2006 | 34 | 4 | 346-352 | DAVID TELL and PETER M. NILSSON | Early ageing in middle-aged men is associated with adverse social factors and increased mortality risk: The Malmö Preventive Project |
| 2006 | 34 | 4 | 353-362 | VILLE V. HELASOJA, EERO LAHELMA, RITVA S. PRÄTTÄLÄ, KRISTIINA M. PATJA, JURATE KLUMBIENE, IVETA PUDULE and ANU KASMEL | Determinants of daily smoking in Estonia, Latvia, Lithuania, and Finland in 1994-2002 |
| 2006 | 34 | 4 | 363-370 | ULLA CHRISTENSEN, LONE SCHMIDT, MARGIT KRIEGBAUM, CHARLOTTE ØRSTED HOUGAARD and BJØRN E. HOLSTEIN | Coping with unemployment: Does educational attainment make any difference? |
| 2006 | 34 | 4 | 371-377 | PETER VESTERGAARD, LARS REJNMARK and LEIF MOSEKILDE | Socioeconomic aspects of fractures within universal public healthcare: A nationwide case-control study from Denmark |
| 2006 | 34 | 4 | 378-386 | PÄIVI SAINIO, SEPPO KOSKINEN, MARKKU HELIÖVAARA, TUIJA MARTELIN, TOMMI HÄRKÄNEN, HEIKKI HURRI, SEPPO MIILUNPALO and ARPO AROMAA | Self-reported and test-based mobility limitations in a representative sample of Finns aged 30+ |
| 2006 | 34 | 4 | 387-396 | NILS M. ERIKSSON and BERNDT G. T. STENBERG | Baseline prevalence of symptoms related to indoor environment |
| 2006 | 34 | 4 | 397-405 | MARTIN LINDSTRÖM, MAHNAZ MOGHADDASSI and JUAN MERLO | Individual and contextual determinants of self-reported poor psychological health: A population-based multilevel analysis in southern Sweden |
| 2006 | 34 | 4 | 406-413 | BO WERNER, LENNART BODIN and SVEN BREMBERG | Data on height and weight from school health records as a national public health surveillance tool: The case of Sweden |
| 2006 | 34 | 4 | 414-421 | MARIE KLINGBERG-ALLVIN, NGUYEN THU NGA, ANNA-BERIT RANSJÖ-ARVIDSON and ANNIKA JOHANSSON | Perspectives of midwives and doctors on adolescent sexuality and abortion care in Vietnam |
| 2006 | 34 | 4 | 422-431 | KRISTIAN BOLIN, BJÖRN LINDGREN and STEPHAN RÖSSNER | The significance of overweight and obesity for individual health behaviour: An economic analysis based on the Swedish surveys of living conditions 1980-81, 1988-89, and 1996-97 |
| 2006 | 34 | 4 | 432-436 | RUNE JACOBSEN, NIELS KEIDING and ELSEBETH LYNGE | Causes of death behind low life expectancy of Danish women |
| 2006 | 34 | 4 | 437-444 | NOËL C. BARENGO, AULIKKI NISSINEN, HEIKKI PEKKARINEN, PIRJO HALONEN and JAAKKO TUOMILEHTO | Twenty-five-year trends in lifestyle and socioeconomic characteristics in Eastern Finland |
| 2006 | 34 | 4 | 445 |  | PUBLIC HEALTH CALENDAR |
| 2006 | 34 | 5 | 449-452 | GISELA DAHLQUIST | Ethics in research: Why and how? |
| 2006 | 34 | 5 | 453-461 | K. MARGARETA ERIKSSON, CARL-JOHAN WESTBORG and MATS C. E. ELIASSON | A randomized trial of lifestyle intervention in primary healthcare for the modification of cardiovascular risk factors: The Björknäs study |
| 2006 | 34 | 5 | 462-471 | JENS GUNDGAARD | Income-related inequality in utilization of health services in Denmark: Evidence from Funen County |
| 2006 | 34 | 5 | 472-479 | TERRY HARTIG and URBAN FRANSSON | Housing tenure and early retirement for health reasons in Sweden |
| 2006 | 34 | 5 | 480-487 | SOFIA CARLSSON, TOMAS ANDERSSON, ALICJA WOLK and ANDERS AHLBOM | Low physical activity and mortality in women: Baseline lifestyle and health as alternative explanations |
| 2006 | 34 | 5 | 488-495 | DAIVA RASTENYTE, DIANA SOPAGIENE, DALIA VIRVICIUTE and KRISTINA JURENIENE | Diverging trends in the incidence and mortality of stroke during the period 1986-2002: A study from the Stroke register in Kaunas, Lithuania |
| 2006 | 34 | 5 | 496-503 | DORTE REFF OLSEN, EDITH MONTGOMERY, SØREN BØJHOLM and ANDERS FOLDSPANG | Prevalent musculoskeletal pain as a correlate of previous exposure to torture |
| 2006 | 34 | 5 | 504-514 | JAN EGGERT and KRISTINA SUNDQUIST | Socioeconomic factors, country of birth, and years in Sweden are associated with first birth fertility trends during the 1990s: A national cohort study |
| 2006 | 34 | 5 | 515-525 | SALLA-MAARIT VOLANEN, SAKARI SUOMINEN, EERO LAHELMA, MARKKU KOSKENVUO and KARRI SILVENTOINEN | Sense of coherence and its determinants: A comparative study of the Finnish-speaking majority and the Swedish-speaking minority in Finland |
| 2006 | 34 | 5 | 526-533 | GLORIA MACASSA, ANTONIO PONCE DE LEON and BO BURSTRÖM | The impact of water supply and sanitation on area differentials in the decline of diarrhoeal disease mortality among infants in Stockholm 1878-1925 |
| 2006 | 34 | 5 | 534-543 | CARL-GUSTAF BORNEHAG, JAN SUNDELL, TORBEN SIGSGAARD and STAFFAN JANSON | Potential self-selection bias in a nested case-control study on indoor environmental factors and their association with asthma and allergic symptoms among pre-school children |
| 2006 | 34 | 5 | 544-554 | EVA SELLSTRÖM and SVEN BREMBERG | The significance of neighbourhood context to child and adolescent health and well-being: A systematic review of multilevel studies |
| 2006 | 34 | 5 | 555-558 | CAMILLA IHLEBÆK, TOMMY H. HANSSON, EVEN LÆRUM, SØREN BRAGE, HEGE R. ERIKSEN, STEN H. HOLM, ROLF SVENDSRØD and AAGE INDAHL | Prevalence of low back pain and sickness absence: A "borderline" study in Norway and Sweden |
| 2006 | 34 | 5 | 559 |  | PUBLIC HEALTH CALENDAR |
| 2006 | 34 | 6 | 561-565 | ILONA KICKBUSCH | The need for a European Strategy on Global Health |
| 2006 | 34 | 6 | 566-567 | MICHAEL F. DRUMMOND, GRETE BOTTEN, UNTO HÄKKINEN and KJELD MØLLER PEDERSEN | Assessing the quality of Swedish health economics research |
| 2006 | 34 | 6 | 568-575 | SUSANNA CALLING, BO HEDBLAD, GUNNAR ENGSTRÖM, GÖRAN BERGLUND and LARS JANZON | Effects of body fatness and physical activity on cardiovascular risk: Risk prediction using the bioelectrical impedance method |
| 2006 | 34 | 6 | 576-583 | MARGARETA NORBERG, HANS STENLUND, BERNT LINDAHL, KURT BOMAN and LARS WEINEHALL | Contribution of Swedish moist snuff to the metabolic syndrome: A wolf in sheep's clothing? |
| 2006 | 34 | 6 | 584-588 | BERTIL HAGSTROM, BENGT MATTSSON, ANDERS WIMO and RONNY K. GUNNARSSON | More illness and less disease? A 20-year perspective on chronic disease and medication |
| 2006 | 34 | 6 | 589-597 | LISE LUND HÅHEIM, INGAR HOLME, INGVAR HJERMANN and SERENA TONSTAD | Risk-factor profile for the incidence of subarachnoid and intracerebral haemorrhage, cerebral infarction, and unspecified stroke during 21 years' follow-up in men |
| 2006 | 34 | 6 | 598-608 | RITVA LAAMANEN, JOHN ØVRETVEIT, JARI SUNDELL, NINA SIMONSEN-REHN, SAKARI SUOMINEN and MATS BROMMELS | Client perceptions of the performance of public and independent not-for-profit primary healthcare |
| 2006 | 34 | 6 | 609-615 | BO HEDBLAD, GUNNAR ENGSTRÖM, ELLIS JANZON, GÖRAN BERGLUND and LARS JANZON | COHb% as a marker of cardiovascular risk in never smokers: Results from a population-based cohort study |
| 2006 | 34 | 6 | 616-622 | TINA B. MIKKELSEN, MERETE OSLER, IVANKA OROZOVA-BEKKEVOLD, VIBEKE K. KNUDSEN and SJURDUR F. OLSEN | Association between fruit and vegetable consumption and birth weight: A prospective study among 43,585 Danish women |
| 2006 | 34 | 6 | 623-631 | THOMAS DRIVSHOLM, LENE FALGAARD EPLOV, MICHAEL DAVIDSEN, TORBEN JØRGENSEN, HANS IBSEN, HANNE HOLLNAGEL and KNUT BORCH-JOHNSEN | Representativeness in population-based studies: A detailed description of non-response in a Danish cohort study |
| 2006 | 34 | 6 | 632-640 | MIKAEL FOGELHOLM, RAISA VALVE, PILVIKKI ABSETZ, HEIKKI HEINONEN, ANTTI UUTELA, KRISTIINA PATJA, ANTTI KARISTO, RIIKKA KONTTINEN, TIINA MÄKELÄ, AULIKKI NISSINEN, PIIA JALLINOJA, OLLI NUMMELA and MARTTI TALJA | Rural-urban differences in health and health behaviour: A baseline description of a community health-promotion programme for the elderly |
| 2006 | 34 | 6 | 641-653 | LARS A. HAGBERG and LARS LINDHOLM | Cost-effectiveness of healthcare-based interventions aimed at improving physical activity |
| 2006 | 34 | 6 | 654-659 | KAROLINA ANDERSSON, TOVE JÖRGENSEN and ANDERS CARLSTEN | Physicians' opinions and experiences of the Pharmaceutical Benefits Reform |
| 2006 | 34 | 6 | 660-664 | PETER M. NILSSON, STEFAN HOFVENDAHL, ERIK HOFVENDAHL, LENA BRANDT and ANDERS EKBOM | Smoking in pregnancy in relation to gender and adult mortality risk in offspring: The Helsingborg Birth Cohort Study |
| 2006 | 34 | 6 | 665-668 | EMILIE E. AGARDH, ANDERS AHLBOM, TOMAS ANDERSSON and CLAES-GÖRAN ÖSTENSON | The magnitude of bias in a cross-sectional study on lifestyle factors in relation to Type 2 diabetes |
| 2006 | 34 | 6 | 669 |  | PUBLIC HEALTH CALENDAR |
| 2007 | 35 | 1 | 1-3 | JOHAN P. MACKENBACH | Public health ethics in times of global environmental change: time to look beyond human interests |
| 2007 | 35 | 1 | 4-10 | LISE LUND HÅHEIM, SERENA TONSTAD, INGVAR HJERMANN, PAUL LEREN and INGAR HOLME | Predictiveness of body mass index for fatal coronary heart disease in men according to length of follow-up: A 21-year prospective cohort study |
| 2007 | 35 | 1 | 11-16 | LENA FURUGREN and LUCIE LAFLAMME | Hip fractures among the elderly in a Swedish urban setting: Different perspectives on the significance of country of birth |
| 2007 | 35 | 1 | 17-22 | RICKARD LJUNG and JOHAN HALLQVIS | Misclassification of occupation-based socioeconomic position and gender comparisons of socioeconomic risk |
| 2007 | 35 | 1 | 23-30 | KARINA FRIIS, OLA EKHOLM, YRSA A. HUNDRUP, ERIK B. OBEL and MORTEN GRØNBÆK | Influence of health, lifestyle, working conditions, and sociodemography on early retirement among nurses: The Danish Nurse Cohort Study |
| 2007 | 35 | 1 | 31-38 | MAJA-LISA LØCHEN, INGER TORHILD GRAM, SIGRID SKATTEBO and NILS KOLSTRUP | Tobacco images and texts in Norwegian magazines and newspapers |
| 2007 | 35 | 1 | 39-47 | OLLI P. NUMMELA, TOMMI T. SULANDER, HEIKKI S. HEINONEN and ANTTI K. UUTELA | Self-rated health and indicators of SES among the ageing in three types of communities |
| 2007 | 35 | 1 | 48-54 | NINA KARNEHED, FINN RASMUSSEN and MALIN KARK | Obesity in young adulthood and later disability pension: A population-based cohort study of 366,929 Swedish men |
| 2007 | 35 | 1 | 55-61 | MONICA CHRISTIANSON, ANN LALOS, GÖRAN WESTMAN and EVA E. JOHANSSON | "Eyes Wide Shut' - Sexuality and risk in HIV-positive youth in Sweden: A qualitative study |
| 2007 | 35 | 1 | 62-69 | JANE E. FERRIE, HUGO WESTERLUND, GABRIEL OXENSTIERNA and TÖRES THEORELL | The impact of moderate and major workplace expansion and downsizing on the psychosocial and physical work environment and income in Sweden |
| 2007 | 35 | 1 | 70-77 | KIRSTEN DANIELSEN, ANDREW M. GARRATT, ØYVIND ANDRESEN BJERTNÆS and KJELL I. PETTERSEN | Patient experiences in relation to respondent and health service delivery characteristics: A survey of 26,938 patients attending 62 hospitals throughout Norway |
| 2007 | 35 | 1 | 78-85 | KAREN ALBERTSEN, THOMAS LUND, KARL B. CHRISTENSEN, TAGE S. KRISTENSEN and EBBE VILLADSEN | Predictors of disability pension over a 10-year period for men and women |
| 2007 | 35 | 1 | 86-94 | BIRGIT DREWS, CLAUS V. NIELSEN, METTE S. RASMUSSEN, JAKOB HJORT and JENS P. BONDE | Improving motivation and goal setting for return to work in a population on sick leave: A controlled study |
| 2007 | 35 | 1 | 95-103 | LEENA K. KOIVUSILTA, TOMI P. LINTONEN and ARJA H. RIMPELÄ | Orientations in adolescent use of information and communication technology: A digital divide by sociodemographic background, educational career, and health |
| 2007 | 35 | 1 | 104-110 | STEN-ÅKE STENBERG, DENNY VÅGERÖ, REIDAR ÖSTERMAN, EMMA ARVIDSSON, CECILIA VON OTTER and CARL-GUNNAR JANSON | Stockholm Birth Cohort Study 1953-2003: A new tool for life-course studies |
| 2007 | 35 | 1 | 111 |  | PUBLIC HEALTH CALENDAR |
| 2007 | 35 | 2 | 113-115 | DENNY VÅGERÖ | Health inequalities across the globe demand new global policies |
| 2007 | 35 | 2 | 116-124 | ANNE M. HEIKKINEN, GUSTAV J. WICKSTRÖM and HELENA LEINO-KILPI | Privacy in occupational health practice: Promoting and impeding factors |
| 2007 | 35 | 2 | 125-132 | LINDA HÄGERHED ENGMAN, CARL-GUSTAF BORNEHAG and JAN SUNDELL | How valid are parents' questionnaire responses regarding building characteristics, mouldy odour, and signs of moisture problems in Swedish homes? |
| 2007 | 35 | 2 | 133-139 | STEPHANIE BURROWS, MARJAN VAEZ and LUCIE LAFLAMME | Sex-specific suicide mortality in the South African urban context: The role of age, race, and geographical location |
| 2007 | 35 | 2 | 140-147 | THOMAS MILDESTVEDT and EIVIND MELAND | Examining the "Matthew Effect" on the motivation and ability to make lifestyle changes in 217 heart rehabilitation patients |
| 2007 | 35 | 2 | 148-156 | ÅSA KETTIS-LINDBLAD, LENA RING, EVA VIBERTH and MATS G. HANSSON | Perceptions of potential donors in the Swedish public towards information and consent procedures in relation to use of human tissue samples in biobanks: A population-based study |
| 2007 | 35 | 2 | 157-163 | KIRSTI VALSET, SILLE OHREM NAPER, BJØRGULF CLAUSSEN and ODD STEFFEN DALGARD | Does mastering have an effect on disability pensioning independent of health, and may it explain divides of education in the Oslo Health Survey? |
| 2007 | 35 | 2 | 164-171 | FRANK KRARUP ANDERSEN, KAARE CHRISTENSEN and HENRIK FREDERIKSEN | Self-rated health and age: A cross-sectional and longitudinal study of 11,000 Danes aged 45-102 |
| 2007 | 35 | 2 | 172-179 | TINA B. MIKKELSEN, SJURDUR F. OLSEN, SALKA E. RASMUSSEN and MERETE OSLER | Relative validity of fruit and vegetable intake estimated by the food frequency questionnaire used in the Danish National Birth Cohort |
| 2007 | 35 | 2 | 180-186 | DAN K. KAYE, ANNA MIA EKSTRÖM, ANNIKA JOHANSSON, GRACE BANTEBYA and FLORENCE M. MIREMBE | Escaping the triple trap: Coping strategies of pregnant adolescent survivors of domestic violence in Mulago hospital, Ugand |
| 2007 | 35 | 2 | 187-196 | KRISTIAN BOLIN and BJÖRN LINDGREN | Smoking, healthcare cost, and loss of productivity in Sweden 2001 |
| 2007 | 35 | 2 | 197-204 | VIVECA ÖSTBERG and CARIN LENNARTSSON | Getting by with a little help: The importance of various types of social support for health problems |
| 2007 | 35 | 2 | 205-211 | SIGNILD VALLGÅRDA | Public health policies: A Scandinavian model? |
| 2007 | 35 | 2 | 212-215 | PEKKA MARTIKAINEN, MIKKO LAAKSONEN, KUSTAA PIHA and TEA LALLUKKA | Does survey non-response bias the association between occupational social class and health? |
| 2007 | 35 | 2 | 216-221 | MOHABBAT MOHSENI and MARTIN LINDSTRÖM | Comparison of two items on self-rated health |
| 2007 | 35 | 2 | 222 |  | PUBLIC HEALTH CALENDAR |
| 2007 | 35 | 2 | 223 |  | Acknowledgements |
| 2007 | 35 | 3 | 225-227 | ANDERS BRÄNDSTRÖM | Ageing - a cross-cutting research and policy challenge |
| 2007 | 35 | 3 | 228-234 | ANNIKA S. K. FORSSÉN | Humour, beauty, and culture as personal health resources: Experiences of elderly Swedish women |
| 2007 | 35 | 3 | 235-242 | VIDA VOLBEKIENĖ and AUŠRA GRICIṺTĖ | Health-related physical fitness among schoolchildren in Lithuania: A comparison from 1992 to 2002 |
| 2007 | 35 | 3 | 243-249 | BJØRN E. HOLSTEIN, PERNILLE DUE, GERT ALMIND and KIRSTEN AVLUND | Eight-year change in functional ability among 70- to 95-year-olds |
| 2007 | 35 | 3 | 250-256 | CHRISTOPHER SHIELS and MARK B. GABBAY | Patient, clinician, and general practice factors in long-term certified sickness |
| 2007 | 35 | 3 | 257-264 | PETRA LÖFSTEDT, GEBRENEGUS GHILAGABER and ANNIKA JOHANSSON | Birth-spacing patterns in Huaning County, Yunnan Province, PRC: Is the adoption of a small family norm sustainable? |
| 2007 | 35 | 3 | 265-271 | ALECK OSTRY, STEFANIA MAGGI, JAMES TANSEY, JAMES DUNN, RUTH HERSHLER, LISA CHEN, A. M. LOUIE and CLYDE HERTZMAN | The impact of psychosocial work conditions on attempted and completed suicide among western Canadian sawmill workers |
| 2007 | 35 | 3 | 272-277 | ELLIS JANZON, GUNNAR ENGSTRÖM, BO HEDBLAD, GÖRAN BERGLUND and LARS JANZON | Smoking as a determinant of the geographical pattern of cardiac events among women in an urban population |
| 2007 | 35 | 3 | 278-287 | ELENI TH. PETRIDOU, SPYROS KYLLEKIDIS, SUSANNE JEFFREY, PARVEEN CHISHTI, NICK DESSYPRIS and DAVID H. STONE | Unintentional injury mortality in the European Union: How many more lives could be saved? |
| 2007 | 35 | 3 | 288-297 | CARL OTTO SCHELL, MARIE REILLY, HANS ROSLING, STEFAN PETERSON and ANNA MIA EKSTRÖM | Socioeconomic determinants of infant mortality: A worldwide study of 152 low-, middle-, and high-income countries |
| 2007 | 35 | 3 | 298-305 | MINNA NIKULA, PÄIVIKKI KOPONEN, ELINA HAAVIO-MANNILA and ELINA HEMMINKI | Sexual health among young adults in Finland: Assessing risk and protective behaviour through a general health survey |
| 2007 | 35 | 3 | 306-312 | TORE TYNES and TOR HALDORSEN | Mortality in the Sami population of North Norway, 1970-98 |
| 2007 | 35 | 3 | 312-322 | BIRGIT V. L. NICLASEN and PETER BJERREGAARD | Child health in Greenland |
| 2007 | 35 | 3 | 323-329 | MADS KAMPER-JØRGENSEN, JAN WOHLFAHRT, JACOB SIMONSEN and CHRISTINE S. BENN | The Childcare Database: a valuable register linkage |
| 2007 | 35 | 3 | 330-334 | KATARINA SWAHNBERG and KLAAS WIJMA | Validation of the Abuse Screening Inventory (ASI) |
| 2007 | 35 | 3 | 335 |  | PUBLIC HEALTH CALENDAR |
| 2007 | 35 | 4 | 337-340 | JOHN WYN OWEN | Foundations of Europe: Making globalization work for health |
| 2007 | 35 | 4 | 341-347 | NADINE E. KARLSSON, JOHN M. CARSTENSEN, STURLA GJESDAL and KRISTINA A. E. ALEXANDERSON | Mortality in relation to disability pension: Findings from a 12-year prospective population-based cohort study in Sweden |
| 2007 | 35 | 4 | 348-355 | KUSTAA PIHA, PEKKA MARTIKAINEN, OSSI RAHKONEN, EVA ROOS and EERO LAHELMA | Trends in socioeconomic differences in sickness absence among Finnish municipal employees 1990-99 |
| 2007 | 35 | 4 | 356-364 | ANNE M. HAUSKEN, SVETLANA SKURTVEIT, ELIN O. ROSVOLD, JØRGEN G. BRAMNESS and KARI FURU | Psychotropic drug use among persons with mental distress symptoms: A population-based study in Norway |
| 2007 | 35 | 4 | 365-372 | SUSANNE R. RASMUSSEN, JANUS L. THOMSEN, JANNIE KILSMARK, ANNE HVENEGAARD, MARIANNE ENGBERG, TORSTEN LAURITZEN and JES SØGAARD | Preventive health screenings and health consultations in primary care increase life expectancy without increasing costs |
| 2007 | 35 | 4 | 373-379 | CURT HAGQUIST, MONA SUNDH and CHARLI ERIKSSON | Smoking habits before and after the introduction of a minimum-age law for tobacco purchase: Analysis of data on adolescents from three regions of Sweden |
| 2007 | 35 | 4 | 380-386 | PETER BJERREGAARD, MARIT EIKA JØRGENSEN and KNUT BORCH-JOHNSEN | Cardiovascular risk amongst migrant and non-migrant Greenland Inuit in a gender perspective |
| 2007 | 35 | 4 | 387-395 | NETTA E. MÄKI and PEKKA T. MARTIKAINEN | Socioeconomic differences in suicide mortality by sex in Finland in 1971-2000: A register-based study of trends, levels, and life expectancy differences |
| 2007 | 35 | 4 | 296-402 | BIAO XU, VINOD K. DIWAN and LENNART BOGG | Access to tuberculosis care: What did chronic cough patients experience in the way of healthcare-seeking? |
| 2007 | 35 | 4 | 403-409 | METTE AADAHL, MICHAEL KJÆR and TORBEN JØRGENSEN | Perceived exertion of physical activity: Negative association with self-rated fitness |
| 2007 | 35 | 4 | 410-417 | MIKKEL VASS, KIRSTEN AVLUND and CARSTEN HENDRIKSEN | Randomized intervention trial on preventive home visits to older people: Baseline and follow-up characteristics of participants and non-participants |
| 2007 | 35 | 4 | 418-423 | VILLE M. MATTILA, PENTTI KURONEN and HARRI PIHLAJAMÄKI | Nature and risk factors of injury hospitalization in young adults: A follow-up of 135,987 military conscripts |
| 2007 | 35 | 4 | 424-431 | ANN-BRITT E. WIRÉHN, H. MIKAEL KARLSSON and JOHN M. CARSTENSEN | Estimating disease prevalence using a population-based administrative healthcare database |
| 2007 | 35 | 4 | 432-441 | ANNE TJØNNELAND, ANJA OLSEN, KATJA BOLL, CONNIE STRIPP, JANE CHRISTENSEN, GERDA ENGHOLM and KIM OVERVAD | Study design, exposure variables, and socioeconomic determinants of participation in Diet, Cancer and Health: A population-based prospective cohort study of 57,053 men and women in Denmark |
| 2007 | 35 | 4 | 442-444 | CHRISTIN BEXELIUS, KLAUS HOEYER and NIELS LYNÖE | Will forensic use of medical biobanks decrease public trust in healthcare services? Some empirical observations |
| 2007 | 35 | 4 | 445 |  | PUBLIC HEALTH CALENDAR |
| 2007 | 35 | 5 | 449-453 | STIG WALL | Globalization makes new demands on public health research |
| 2007 | 35 | 5 | 454-459 | ANDERS MAGNUSON, LENNART BODIN and SCOTT M. MONTGOMERY | Father's occupation and sex ratio of offspring |
| 2007 | 35 | 5 | 460-467 | MARTIN LINDSTRÖM and ELLIS JANZON | Social capital, institutional (vertical) trust and smoking: A study of daily smoking and smoking cessation among ever smokers |
| 2007 | 35 | 5 | 468-474 | EBBA HEDLUND, JAAKKO KAPRIO, ANDERS LANGE, MARKKU KOSKENVUO, LAURA JARTTI, TAPANI RÖNNEMAA and NIKLAS HAMMAR | Migration and coronary heart disease: A study of Finnish twins living in Sweden and their co-twins residing in Finland |
| 2007 | 35 | 5 | 475-480 | MARWAN KHAWAJA and MONA MOWAFI | Types of cultural capital and self-rated health among disadvantaged women in outer Beirut, Lebanon |
| 2007 | 35 | 5 | 481-489 | LINDA KAERLEV, SØREN DAHL, PER SABRO NIELSEN, JØRN OLSEN, HARALD HANNERZ, ANKER JENSEN and FINN TÜCHSEN | Hospital contacts for chronic diseases among Danish seafarers and fishermen: A population-based cohort study |
| 2007 | 35 | 5 | 490-496 | KARI H. MOEN, HJØRDIS K. BAKKE, ØYVIND BAKKE and EGIL A. FORS | Preschool children's sickness absenteeism from Norwegian regular and outdoor day care centres: A comparative study |
| 2007 | 35 | 5 | 497-502 | NIELS HENRIK HJOLLUND, FINN BREINHOLT LARSEN and JOHAN HVIID ANDERSEN | Register-based follow-up of social benefits and other transfer payments: Accuracy and degree of completeness in a Danish interdepartmental administrative database compared with a population-based survey |
| 2007 | 35 | 5 | 503-509 | ROB H. VAN HOOREN, BART W. VAN DEN BORNE, LEOPOLD M. G. CURFS and GUY A. M. WIDDERSHOVEN | Ethics of prevention: An interactive computer-tailored program |
| 2007 | 35 | 5 | 510-515 | HENRIK BRØNNUM-HANSEN, KNUD JUEL, MICHAEL DAVIDSEN and JAN SØRENSEN | Impact of selected risk factors on quality-adjusted life expectancy in Denmark |
| 2007 | 35 | 5 | 516-523 | THERESE STENLUND, CHRISTINA AHLGREN, BERNT LINDAHL, GUNILLA BURELL, ANDERS KNUTSSON, BIRGITTA STEGMAYR and LISBETH SLUNGA BIRGANDER | Patients with burnout in relation to gender and a general population |
| 2007 | 35 | 5 | 524-532 | PER MUHR, FINN RASMUSSEN and ULF ROSENHALL | Prevalence of hearing loss among 18-year-old Swedish men during the period 1971-1995 |
| 2007 | 35 | 5 | 533-539 | MIKAEL RAHMQVIST and ANA-CLAUDIA BARA | Patients retrieving additional information via the Internet: A trend analysis in a Swedish population, 2000-05 |
| 2007 | 35 | 5 | 540-547 | SI̊GVE OLTEDAL, ANDREW GARRATT, ØYVIND BJERTNÆS, MARGRÉT BJØRNSDOTTÌR, MORTEN FREIL and MAGNA SACHS | The NORPEQ patient experiences questionnaire: Data quality, internal consistency and validity following a Norwegian inpatient survey |
| 2007 | 35 | 5 | 548-554 | SOPHIA ZACKRISSON, MARTIN LINDSTRÖM, MAHNAZ MOGHADDASSI, INGVAR ANDERSSON and LARS JANZON | Social predictors of non-attendance in an urban mammographie screening programme: A multilevel analysis |
| 2007 | 35 | 5 | 555-558 | ELISABETH SVENSSON, DEBORAH L. REAS, INGER SANDANGER and JAN F. NYGÅRD | Urban-rural differences in BMI, overweight and obesity in Norway (1990 and 2001) |
| 2007 | 35 | 5 | 559 |  | PUBLIC HEALTH CALENDAR |
| 2007 | 35 | 6 | 561-563 | RAINER SAUERBORN | Climate change: An agenda for research and teaching in public health |
| 2007 | 35 | 6 | 564-569 | ALDONA GAIŽAUSKIENĖ, ŽILVINAS PADAIGA, SKIRMANTĖ STARKUVIENĖ and RŪTA MIZERIENĖ | Prediction of perinatal mortality at an early stage of pregnancy |
| 2007 | 35 | 6 | 570-576 | ØYVIND NÆSS, BJØRGULF CLAUSSEN and GEORGE DAVEY SMITH | Housing conditions in childhood and cause-specific adult mortality: The effect of sanitary conditions and economic deprivation on 55,761 men in Oslo |
| 2007 | 35 | 6 | 577-584 | MARIANNE UPMARK, KARIN BORG and KRISTINA ALEXANDERSON | Gender differences in experiencing negative encounters with healthcare: A study of long-term sickness absentees |
| 2007 | 35 | 6 | 585-590 | ELIANNE RISKA and THOMAS HEIKELL | Gender and images of heart disease in Scandinavian drug advertising |
| 2007 | 35 | 6 | 591-598 | THOMAS MILDESTVEDT, EIVIND MELAND and GEIR EGIL EIDE | No difference in lifestyle changes by adding individual counselling to group-based rehabilitation RCT among coronary heart disease patients |
| 2007 | 35 | 6 | 599-608 | BRIAN WELLS PENCE, PHILOMENA NYARKO, JAMES F. PHILLIPS and CORNELIUS DEBPUUR | The effect of community nurses and health volunteers on child mortality: The Navrongo Community Health and Family Planning Project |
| 2007 | 35 | 6 | 609-617 | MARCUS WESTIN and RAGNAR WESTERLING | Social capital and inequality in health between single and couple parents in Sweden |
| 2007 | 35 | 6 | 618-622 | BENGT HAGLUND, MATS ELIASSON, MAGNUS STENBECK and MÅNS ROSÉN | Is moist snuff use associated with excess risk of IHD or stroke? A longitudinal follow-up of snuff users in Sweden |
| 2007 | 35 | 6 | 623-630 | ANTONIO PONCE DE EON, LEIF SVANSTRÖM, GLENN WELANDER, LOTHAR SCHELP, PER SANTESSON and ROBERT EKMAN | Differences in child injury hospitalizations in Sweden: The use of timetrend analysis to compare various community injury-prevention approaches |
| 2007 | 35 | 6 | 631-639 | ASJA LEMCKE, METTE KJØLLER, OLA EKHOLM and ELSE SMITH | HIV testing in the Danish population: A national representative survey, 2000 |
| 2007 | 35 | 6 | 640-647 | ROBERT JONZON, NGUYEN DANG VUNG, KARIN C. RINGSBERG and GUNILLA KRANTZ | Violence against women in intimate relationships: Explanations and suggestions for interventions as perceived by healthcare workers, local leaders, and trusted community members in a northern district of Vietnam |
| 2007 | 35 | 6 | 648-654 | JØRGEN NEXØE, PEDER ANDREAS HALVORSEN and IVAR SØNBØ KRISTIANSEN | Critiques of the risk concept - valid or not? |
| 2007 | 35 | 6 | 655-661 | MARIE NORREDAM and DAG ALBUM | Prestige and its significance for medical specialties and diseases |
| 2007 | 35 | 6 | 662-665 | LARS JERDÉN, PIA BILDT-STRÖM, GUNILLA BURELL, LARS WEINEHALL and ERIK BERGSTRÖM | Personal health documents in school health education: A feasibility study |
| 2007 | 35 | 6 | 666-669 | MIGUEL SAN SEBASTIÁN and ANNA-KARIN HURTIG | Is GATS a concern for the Swedish healthcare system? |
| 2007 | 35 | 6 | 670 |  | PUBLIC HEALTH CALENDAR |
| 2008 | 36 | 1 | 1-2 | FINN KAMPER-JØRGENSEN | New editor and new publisher for the "Scandinavian Journal of Public Health" |
| 2008 | 36 | 1 | 3-11 | ANNI BRIT STERNHAGEN NIELSEN, VOLKERT SIERSMA, LINE CONRADSEN HIORT, THOMAS DRIVSHOLM, SVEND KREINER and HANNE HOLLNAGEL | Self-rated general health among 40-year-old Danes and its association with all-cause mortality at 10-, 20-, and 29 years' follow-up |
| 2008 | 36 | 1 | 12-20 | HANS-JOHAN BREIDABLIK, EIVIND MELAND and STIAN LYDERSEN | Self-rated health in adolescence: A multifactorial composite |
| 2008 | 36 | 1 | 21-27 | CARIN STALAND-NYMAN, KRISTINA ALEXANDERSON and GUNNEL HENSING | Associations between strain in domestic work and self-rated health: A study of employed women in Sweden |
| 2008 | 36 | 1 | 28-34 | MOHABBAT MOHSENI and MARTIN LINDSTRÖM | Social capital, political trust and self rated-health: A population-based study in southern Sweden |
| 2008 | 36 | 1 | 35-43 | ELINA NIHTILÄ and PEKKA MARTIKAINEN | Why older people living with a spouse are less likely to be institutionalized: The role of socioeconomic factors and health characteristics |
| 2008 | 36 | 1 | 44-51 | HENRIK BRØNNUM-HANSEN and MIKKEL BAADSGAARD | Increase in social inequality in health expectancy in Denmark |
| 2008 | 36 | 1 | 52-61 | NANNA KURTZE, VEGAR RANGUL, BO-EGIL HUSTVEDT and W DANA FLANDERS | Reliability and validity of self-reported physical activity in the Nord-Trøndelag Health Study – HUNT 1 |
| 2008 | 36 | 1 | 62-68 | MARJOLEIN M. IVERSEN, KRISTIAN MIDTHJELL, TRULS ØSTBYE, GRETHE S. TELL, ELIZABETH CLIPP, RICHARD SLOANE, MONICA W. NORTVEDT, SVERRE UHLVING and BERIT R. HANESTAD | History of and factors associated with diabetic foot ulcers in Norway: The Nord-Trøndelag Health Study |
| 2008 | 36 | 1 | 69-75 | E. FHÄRM, O. ROLANDSSON and L. WEINEHALL | Guidelines improve general trend of lowered cholesterol levels in type 2 diabetes patients in spite of low adherence |
| 2008 | 36 | 1 | 76-83 | ANNA MYGIND, MARIE NORREDAM, ANETTE S. NIELSEN, JENS BAGGER and ALLAN KRASNIK | The effect of patient origin and relevance of contact on patient and caregiver satisfaction in the emergency room |
| 2008 | 36 | 1 | 84-91 | PER SJÖLANDER, SVEN HASSLER and URBAN JANLERT | Stroke and acute myocardial infarction in the Swedish Sami population: Incidence and mortality in relation to income and level of education |
| 2008 | 36 | 1 | 92-98 | KIRSTI MALTERUD and JANECKE THESEN | When the helper humiliates the patient: A qualitative study about unintended intimidations |
| 2008 | 36 | 1 | 99-106 | A. DE COSTA, V. SARAF, M. JHALANI, V. K. MAHADIK and V. K. DIWAN | Managing with maps? The development and institutionalization of a map-based health management information system in Madhya Pradesh, India |
| 2008 | 36 | 1 | 107-111 | Paula Risikko and Gudlaugur Thor Thordarson | News on Health Policy and Public Health |
| 2008 | 36 | 1 | 112 |  | PUBLIC HEALTH CALENDAR |
| 2008 | 36 | 2 | 113-116 | ANDERS FOLDSPANG | Public health education in Europe and the Nordic Countries: status and perspectives |
| 2008 | 36 | 2 | 117-125 | KATHRINE CARLSEN, SUSANNE OKSBJERG DALTON, KIRSTEN FREDERIKSEN, FINN DIDERICHSEN and CHRISTOFFER JOHANSEN | Cancer and the risk for taking early retirement pension: A Danish cohort study |
| 2008 | 36 | 2 | 126-134 | MIKAEL ROSTILA | The Swedish labour market in the 1990s: The very last of the healthy jobs? |
| 2008 | 36 | 2 | 135-142 | ÅSE VIKANES, ANDREJ M. GRPBOVSKI, SIRI VANGEN and PER MAGNUS | Variations in prevalence of hyperemesis gravidarum by country of birth: A study of 900,074 pregnancies in Norway, 1967-2005 |
| 2008 | 36 | 2 | 143-152 | E. REGUSHEVSKAYA, T. DUBIKAYTIS, M. NIKULA, O. KUZNETSOVA and E. HEMMINKI | The socioeconomic characteristics of risky sexual behaviour among reproductive-age women in St Petersburg |
| 2008 | 36 | 2 | 153-160 | JEPPE MATTHIESSEN, MARGIT VELSING GROTH, SISSE FAGT, ANJA BILTOFT-JENSEN, ANDERS STOCKMARR, JENS STRODL ANDERSEN and ELLEN TROLLE | Prevalence and trends in overweight and obesity among children and adolescents in Denmark |
| 2008 | 36 | 2 | 161-168 | ASTRID IRENE NERØIEN and BERIT SCHEI | Partner violence and health: Results from the first national study on violence against women in Norway |
| 2008 | 36 | 2 | 169-176 | GUNILLA RINGBÄCK WEITOFT, MATS ELIASSON and MÅNS ROSÉN | Underweight, overweight and obesity as risk factors for mortality and hospitalization |
| 2008 | 36 | 2 | 177-182 | EVA JAKOBSSON, FANNIE GASTON-JOHANSSON, JOAKIM ÖHLÉN and INGRID BERGH | Clinical problems at the end of life in a Swedish population, including the role of advancing age and physical and cognitive function |
| 2008 | 36 | 2 | 183-189 | ANNEMARIE R. BOLLERUP, MARTIN C. DONOGHOE, JEFFREY V. LAZARUS, STINE NIELSEN and SRDAN MATIC | Access to highly active antiretroviral therapy (HAART) in the WHO European Region 2003-2005 |
| 2008 | 36 | 2 | 190-196 | MARINA TALOYAN, SVEN-ERIK JOHANSSON, JAN SUNDQUIST, TAHIRE O. KOCTÜRK and LEENA MARIA JOHANSSON | Psychological distress among Kurdish immigrants in Sweden |
| 2008 | 36 | 2 | 197-204 | HELGE GARÅSEN, ROLF WINDSPOLL and ROAR JOHNSEN | Long-term patients' outcomes after intermediate care at a community hospital for elderly patients: 12-month follow-up of a randomized controlled trial |
| 2008 | 36 | 2 | 205-210 | ANNE R. HANSEN, OLA EKHOLM and METTE KJØLLER | Health behaviour among non-Western immigrants with Danish citizenship |
| 2008 | 36 | 2 | 211-216 | INGELA KRANTZ, BO ERIKSSON, CRISTINA LUNDQUIST-PERSSON, BETH MAINA AHLBERG and TORE NILSTUN | Screening for postpartum depression with the Edinburgh Postnatal Depression Scale (EPDS): An ethical analysis |
| 2008 | 36 | 2 | 217-222 | Jakob Axel Nielsen and Sylvia Brustad | News on Health Policy and Public Health |
| 2008 | 36 | 2 | 223 |  | PUBLIC HEALTH CALENDAR |
| 2008 | 36 | 3 | 225-227 | MARK MCCARTHY | Public health research in Europe: SPHERE and the Nordic countries |
| 2008 | 36 | 3 | 228-234 | KARIN GULDBRANDSSON, HENRY BÄCK and SVEN BREMBERG | The significance of policy documents in municipal child health promotion |
| 2008 | 36 | 3 | 235-241 | ESTHER ZIMMERMANN, OLA EKHOLM, MORTEN GRØNBÆK and TINE CURTIS | Predictors of changes in physical activity in a prospective cohort study of the Danish adult population |
| 2008 | 36 | 3 | 242-249 | KERSTIN WALDENSTRÖM and ANNIKA HÄRENSTAM | Does the job demand-control model correspond to externally assessed demands and control for both women and men? |
| 2008 | 36 | 3 | 250-257 | STEFAN N. BACKE and RAGNAR ANDERSSON | Monitoring the "tip of the iceberg": Ambulance records as a source of injury surveillance |
| 2008 | 36 | 3 | 258-264 | SIGNE L. RAYCE, ULLA CHRISTENSEN, CHARLOTTE Ø. HOUGAARD and FINN DIDERICHSEN | Economic consequences of incident disease: The effect on loss of annual income |
| 2008 | 36 | 3 | 265-271 | KLAS-GÖRAN SAHLEN, CURT LÖFGREN, BRITT MARI HELLNER and LARS LINDHOLM | Preventive home visits to older people are cost-effective |
| 2008 | 36 | 3 | 272-278 | JACOB HILDEN WINSLØW and VILHELM BORG | Resources and quality of care in services for the elderly |
| 2008 | 36 | 3 | 279-283 | STEIN ATLE LIE, HEGE R. ERIKSEN, HOLGER URSIN and ELI MOLDE HAGEN | A multi-state model for sick-leave data applied to a randomized control trial study of low back pain |
| 2008 | 36 | 3 | 284-291 | CATARINA A. CANIVET, PER-OLOF ÖSTERGREN, IRÉNE L. JAKOBSSON, ELISABETH DEJIN-KARLSSON and BARBRO M. HAGANDER | Infantile colic, maternal smoking and infant feeding at 5 weeks of age |
| 2008 | 36 | 3 | 292-297 | NADIM AL-ADILI, MOHAMMAD SHAHEEN, STAFFAN BERGSTROM and ANNIKA JOHANSSON | Survival, family conditions and nutritional status of motherless orphans in the West Bank, Palestine |
| 2008 | 36 | 3 | 298-302 | FRED A. KOOPMAN, CHARLES D. H. PARRY, BRONWYN MYERS and GAVIN REAGON | Addressing alcohol problems in primary care settings: A study of general medical practitioners in Cape Town, South Africa |
| 2008 | 36 | 3 | 303-309 | JEFFREY V. LAZARUS, LINA JARUSEVICIENE and JERKER LILJESTRAND | Lithuanian general practitioners' knowledge of confidentiality laws in adolescent sexual and reproductive healthcare: A cross-sectional study |
| 2008 | 36 | 3 | 310-317 | SENNEN H. HOUNTON, ISSIAKA SOMBIE, JOHN TOWNEND, THOMAS OUEDRAOGO, NICOLAS MEDA and WENDY J. GRAHAM | The tip of the iceberg: Evidence of seasonality in institutional maternal mortality and implications for health resources management in Burkina Faso |
| 2008 | 36 | 3 | 318-325 | RODOLFO PEÑA, WILTON PÉREZ, MARLON MELÉNDEZ, CARINA KÄLLESTÅL and LARS-ÅKE PERSSON | The Nicaraguan Health and Demographic Surveillance Site, HDSS-León: A platform for public health research |
| 2008 | 36 | 3 | 326-329 | ANETTE ANDERSEN, BJØRN E. HOLSTEIN and PERNILLE DUE | Large-scale alcohol use and socioeconomic position of origin: Longitudinal study from ages 15 to 19 years |
| 2008 | 36 | 3 | 330-333 | YERKO ROJAS, ANDREW STICKLEY and PER CARLSON | Too poor to binge? An examination of economic hardship and its relation to alcohol consumption patterns in Taganrog, Russia |
| 2008 | 36 | 3 | 334-335 |  | EU public health strategy 2008-2013: The EU Council and the Commission urge Member States to focus on health policies |
| 2008 | 36 | 3 | 335 |  | Financing health: OECD reports about health in Denmark and Iceland |
| 2008 | 36 | 3 | 336 |  | PUBLIC HEALTH CALENDAR |
| 2008 | 36 | 4 | 337-339 | SAKARI SUOMINEN and BENGT LINDSTRÖM | Salutogenesis |
| 2008 | 36 | 4 | 340-345 | TIJN HENDRIKX, MATS NILSSON and GÖRAN WESTMAN | Sense of coherence in three cross-sectional studies in Northern Sweden 1994, 1999 and 2004 - patterns among men and women |
| 2008 | 36 | 4 | 346-352 | DORTHE NIELSEN, JESPER RYG, NIS NISSEN, WINNIE NIELSEN, BERIT KNOLD and KIM BRDIXEN | Multidisciplinary patient education in groups increases knowledge on osteoporosis: A randomized controlled trial |
| 2008 | 36 | 4 | 353-360 | LARS LIEN | The association between mental health problems and inflammatory conditions across gender and immigrant status: A population-based cross-sectional study among 10th-grade students |
| 2008 | 36 | 4 | 361-368 | INGA D. SIGFUSDOTTIR, BRYNDIS B. ASGEIRSDOTTIR, JON F. SIGURDSSON and GISLI H. GUDJONSSON | Trends in depressive symptoms, anxiety symptoms and visits to healthcare specialists: A national study among Icelandic adolescents |
| 2008 | 36 | 4 | 369-379 | LINE NIELSEN, TINE CURTIS, TAGE S. KRISTENSEN and NAJA ROD NIELSEN | What characterizes persons with high levels of perceived stress in Denmark? A national representative study |
| 2008 | 36 | 4 | 380-388 | LISA VON HUTH SMITH, STEEN LADELUND, KNUT BORCH-JOHNSEN and TORBEN JØRGENSEN | A randomized multifactorial intervention study for prevention of ischaemic heart disease (Inter99): The long-term effect on physical activity |
| 2008 | 36 | 4 | 389-396 | ULLA PETERSSON, CARL JOHAN ÖSTGREN, LARS BRUDIN, INGVAR OVHED and PETER M. NILSSON | Predictors of successful, self-reported lifestyle changes in a defined middle-aged population: The Söderåkra Cardiovascular Risk Factor Study, Sweden |
| 2008 | 36 | 4 | 397-407 | M. SNEVE and R. JORDE | Cross-sectional study on the relationship between body mass index and smoking, and longitudinal changes in body mass index in relation to change in smoking status: The Tromsø Study |
| 2008 | 36 | 4 | 408-414 | MALIN S. SWARTLING, KRISTINA A. E. ALEXANDERSON and ROLF A. WAHLSTRÖM | Barriers to good sickness certification - an interview study with Swedish general practitioners |
| 2008 | 36 | 4 | 415-423 | ELISABETH HERTFELT WAHN and EVA NISSEN | Sociodemographic background, lifestyle and psychosocial conditions of Swedish teenage mothers and their perception of health and social support during pregnancy and childbirth |
| 2008 | 36 | 4 | 424-428 | WILLY PEDERSEN | Abortion and depression: A population-based longitudinal study of young women |
| 2008 | 36 | 4 | 429-435 | SADIQ MOHAMMAD ALI and MARTIN LINDSTRÖM | Psychosocial work conditions, unemployment and health locus of control: A population-based study |
| 2008 | 36 | 4 | 436-441 | YEMANE BERHANE, STIG WALL, MESGANAW FANTAHUN, ANDERS EMMELIN, WUBEGZIER MEKONNEN, ULF HÖGBERG, ALEMAYEHU WORKU, FIKRU TESFAYE, MITIKE MOLLA, NEGUSSIE DEYESSA, ABERA KUMIE, DAMEN HAILEMARIAM, FIKRE ENQUESELASSIE and PETER BYASS | A rural Ethiopian population undergoing epidemiological transition over a generation: Butajira from 1987 to 2004 |
| 2008 | 36 | 4 | 442-445 | CHRIS METCALFE, JOHN MACLEOD, GEORGE DAVEY SMITH and CAROLE L. HART | The scope for biased recall of risk-factor exposure in case-control studies: Evidence from a cohort study of Scottish men |
| 2008 | 36 | 4 | 446-447 |  | World Health Organization Ministerial Conference: European debate about health systems, health and wealth |
| 2008 | 36 | 4 | 447 |  | The health of children and adolescents: A catalogue of Nordic ways to target unhealthy children |
| 2008 | 36 | 4 | 448 |  | PUBLIC HEALTH CALENDAR |
| 2008 | 36 | 5 | 449-451 | ANN ÖHMAN | Global public health and gender theory: The need for integration |
| 2008 | 36 | 5 | 452-459 | TORGEIR LIMSTRAND and NANCY J. REHRER | Young people's use of sports facilities: A Norwegian study on physical activity |
| 2008 | 36 | 5 | 460-466 | JENS F. L. SØRENSEN | The potential migration effect of rural hospital closures: A Danish case study |
| 2008 | 36 | 5 | 467-474 | JON IVAR ELSTAD and MIA VABØ | Job stress, sickness absence and sickness presenteeism in Nordic elderly care |
| 2008 | 36 | 5 | 475-482 | EVA ANDERSSON, SHARON KÜHLMANN-BERENZON, ANNIKA LINDE, LINUS SCHIÖLER, SANDRA RUBINOVA and MARIANNE FRISÉN | Predictions by early indicators of the time and height of the peaks of yearly influenza outbreaks in Sweden |
| 2008 | 36 | 5 | 483-492 | ANNE TIAINEN, GUNNAR EDMAN, LENA FLYCKT, GORAN TOMSON and CLAS REHNBERG | Regional variations and determinants of direct psychiatric costs in Sweden |
| 2008 | 36 | 5 | 493-503 | RICHARD BRÄNSTRÖM and SVEN ANDRÉASSON | Regional differences in alcohol consumption, alcohol addiction and drug use among Swedish adults |
| 2008 | 36 | 5 | 504-515 | ANTONIO DAPONTE-CODINA, JULIA BOLÍVAR-MUÑOZ, SILVIA TORO-CÁRDENAS, RICARDO OCAÑA-RIOLA, JOAN BENACH-ROVIRA and VICENTE NAVARRO-LÓPEZ | Area deprivation and trends in inequalities in self-rated health in Spain, 1987-2001 |
| 2008 | 36 | 5 | 516-523 | JOACIM ROCKLÖV and BERTIL FORSBERG | The effect of temperature on mortality in Stockholm 1998-2003: A study of lag structures and heatwave effects |
| 2008 | 36 | 5 | 524-531 | JÖRDIS JENNIFER OTT, VOLKER WINKLER, CATHERINE KYOBUTUNGI, JUDIT LAKI and HEIKO BECHER | Effects of residential changes and time patterns on external-cause mortality in migrants: Results of a German cohort study |
| 2008 | 36 | 5 | 532-537 | LENA STRAND BERGSTRÖM and NIELS LYNÖE | Enhancing concentration, mood and memory in healthy individuals: An empirical study of attitudes among general practitioners and the general population |
| 2008 | 36 | 5 | 538-545 | BIZU GELAYE, MARC PHILPART, MIRUTS GOSHU, YEMANE BERHANE, ANNETTE L. FITZPATRICK and MICHELLE A. WILLIAMS | Anger expression, negative life events and violent behaviour among male college students in Ethiopia |
| 2008 | 36 | 5 | 546-554 | PIROSKA ÖSTLIN, KRISTINA KLERDAL and NIKLAS HAMMAR | Time trends in incidence of myocardial infarction in male and female dominated occupations in Stockholm, Sweden |
| 2008 | 36 | 5 | 555-557 | Maria Larsson | A new public health policy in Sweden |
| 2008 | 36 | 5 | 558-559 | Göran Hägglund | Launching of major dental care reform |
| 2008 | 36 | 5 | 560 |  | PUBLIC HEALTH CALENDAR |
| 2008 | 36 | 6 | 561-563 | KARIN HELWEG-LARSEN | Violence: News on a public health problem |
| 2008 | 36 | 6 | 564-572 | MARGARETHA VOSS, MALIN JOSEPHSON, STEFAN STARK, MARJAN VAEZ, KRISTINA ALEXANDERSON, LARS ALFREDSSON and EVA VINGÅRD | The influence of household work and of having children on sickness absence among publicly employed women in Sweden |
| 2008 | 36 | 6 | 573-579 | REGINA RÈKLAITIENÈ, VYTAUTAS JANILIONIS, MARIUS NOREIKA, ABDONAS TAMOŠIŪNAS, DALIA VIRVIČIŪTĖ and DIANA ŠOPAGIENĖ | Effects of age, period and cohort on stroke mortality among a middleaged Lithuanian urban population from 1980 to 2004 |
| 2008 | 36 | 6 | 580-588 | KIM BLOOMFIELD, ULRIKE GRITTNER, HANNA BARBARA RASMUSSEN and HANS CHRISTIAN PETERSEN | Socio-demographic correlates of alcohol consumption in the Danish general population |
| 2008 | 36 | 6 | 589-597 | N. DEYESSA, Y. BERHANE, A. ALEM, U. HOGBERG and G. KULLGREN | Depression among women in rural Ethiopia as related to socioeconomic factors: A community-based study on women in reproductive age groups |
| 2008 | 36 | 6 | 598-606 | INGELISE ANDERSEN, NIELS KR. RASMUSSEN, P. O. ÖSTERGREN, FRIDA CARLSSON, MATHIAS GRAHN and FINN DIDERICHSEN | Does job strain mediate the effect of socioeconomic group on smoking behaviour? The impact of different health policies in Denmark and Sweden |
| 2008 | 36 | 6 | 607-618 | ILONA LENCIAUSKIENE and APOLINARAS ZABORSKIS | The effects of family structure, parent-child relationship and parental monitoring on early sexual behaviour among adolescents in nine European countries |
| 2008 | 36 | 6 | 619-628 | SANNA TIIKKAJA and ÖRJAN HEMSTRÖM | Does intergenerational social mobility among men affect cardiovascular mortality? A population-based register study from Sweden |
| 2008 | 36 | 6 | 629-634 | HOANG VAN MINH, DAO LAN HUONG and KIM BAO GIANG | Self-reported chronic diseases and associated sociodemographic status and lifestyle risk factors among rural Vietnamese adults |
| 2008 | 36 | 6 | 635-649 | TINA JØRGENSEN, LAUST H. MORTENSEN and ANNE-MARIE NYBO ANDERSEN | Social inequality in fetal and perinatal mortality in the Nordic countries |
| 2008 | 36 | 6 | 650-661 | TORSTEN LAURITZEN, MORTEN SIG AGER JENSEN, JANUS LAUST THOMSEN, BO CHRISTENSEN and MARIANNE ENGBERG | Health tests and health consultations reduced cardiovascular risk without psychological strain, increased healthcare utilization or increased costs: An overview of the results from a 5-year randomized trial in primary care. The Ebeltoft Health Promotion Project (EHPP) |
| 2008 | 36 | 6 | 662 |  | News on Health Policy and Public Health: Award: Nordic Public Health Award to professor of psychiatry |
| 2008 | 36 | 6 | 662-663 |  | Childrens' teeth for research: Norway might be building the biggest milk teeth bank in the world |
| 2008 | 36 | 6 | 663 |  | European network: A European public health law network is established |
| 2008 | 36 | 6 | 663 |  | Conference premiere: First World Health Professions Conference on Regulation held in Geneva |
| 2008 | 36 | 6 | 664 |  | PUBLIC HEALTH CALENDAR |
| 2008 | 36 | 6 | 665 |  | Acknowledgements |
| 2008 | 36 | 7 | 673-675 | FINN KAMPER-JØRGENSEN | Public health information systems - and EUPHIX |
| 2008 | 36 | 7 | 676-684 | PETER W. ACHTERBERG, PIETER G. N. KRAMERS and HANS A. M. VAN OERS | European community health monitoring: the EUPHIX-model |
| 2008 | 36 | 7 | 685-689 | CASSIE TREWIN, BJØRN HEINE STRAND and ELSE-KARIN GRØHOLT | Norhealth: Norwegian Health Information System |
| 2008 | 36 | 7 | 690-697 | EVA K. CLAUSSON, LENNART KÖHLER and AGNETA BERG | Schoolchildren's health as judged by Swedish school nurses – a national survey |
| 2008 | 36 | 7 | 698-705 | JEANETTE WESTMAN, TUIJA MARTELIN, TOMMI HÄRKÄNEN, SEPPO KOSKINEN and KRISTINA SUNDQUIST | Migration and self-rated health: a comparison between Finns living in Sweden and Finns living in Finland |
| 2008 | 36 | 7 | 706-712 | ANNIE HOGH, MUBORAK SHARIPOVA and VILHELM BORG | Incidence and recurrent work-related violence towards healthcare workers and subsequent health effects. A one-year follow-up study |
| 2008 | 36 | 7 | 713-719 | PETER A. FLACH, BOUDIEN KROL and JOHAN W. GROOTHOFF | Determinants of sick-leave duration: A tool for managers? |
| 2008 | 36 | 7 | 720-727 | ANNA LINDBLAD, RURIK LÖFMARK and NIELS LYNÖE | Physician-assisted suicide: a survey of attitudes among Swedish physicians |
| 2008 | 36 | 7 | 728-736 | MARIANNE HAAPEA, JOUKO MIETTUNEN, ESA LÄÄRÄ, MATTI I. JOUKAMAA, MARJO-RIITTA JÄRVELIN, MATTI K. ISOHANNI and JUHA M. VEIJOLA | Non-participation in a field survey with respect to psychiatric disorders |
| 2008 | 36 | 7 | 737-743 | LINDA L. MAGNUSSON HANSON, TÖRES THEORELL, GABRIEL OXENSTIERNA, MARTIN HYDE and HUGO WESTERLUND | Demand, control and social climate as predictors of emotional exhaustion symptoms in working Swedish men and women |
| 2008 | 36 | 7 | 744-752 | K. NORDFJÄLL, M. ELIASSON, B. STEGMAYR, S. LUNDIN, G. ROOS and P. M. NILSSON | Increased abdominal obesity, adverse psychosocial factors and shorter telomere length in subjects reporting early ageing; the MONICA Northern Sweden Study |
| 2008 | 36 | 7 | 753-760 | IAN COOK, MARIANNE ALBERTS, SANDY BURGER and PETER BYASS | All-cause mortality trends in Dikgale, rural South Africa, 1996-2003 |
| 2008 | 36 | 7 | 761768 | NDEMA ABU HABIB, ANNE KJERSTI DALVEIT, JOSEPH MLAY, OLOLA ONEKO, JOHN SHAO, PER BERGSJØ, ERIK LIE-NIELSEN and ROLV TERJE LIE | Birthweight and perinatal mortality among singletons and twins in north-eastern Tanzania |
| 2008 | 36 | 7 | 769-777 | ULLA KEMPPAINEN, KERTTU TOSSAVAINEN, ERKKI VARTIAINEN, VEIKKO JOKELA, PEKKA PUSKA, VLADIMIR PANTELEJEV and MIHAIL UHANOV | Environmental factors as predictors of alcohol use among ninth-grade adolescents in Pitkäranta (Russian Karelia) and in eastern Finland |
| 2008 | 36 | 7 | 778 |  | Global alcohol strategy: Sweden will host the first global World Health Organization conference on alcohol |
| 2008 | 36 | 7 | 778-779 |  | HBSC report: Nordic teenagers have basically similar health behaviours |
| 2008 | 36 | 7 | 780 |  | PUBLIC HEALTH CALENDAR |
| 2008 | 36 | 8 | 785-788 | JOHN KEMM | Health impact assessment: An aid to political decision-making |
| 2008 | 36 | 8 | 789-794 | ANNE KATRINE RAVN, HENRIETTE NICOLAISEN, KARINA LINNROSE, MIKAEL W. FOLKERSEN, STELLA R. J. KRAEMER and GABRIEL GULIS | Screening tool development for health impact assessment of large administrative structural changes |
| 2008 | 36 | 8 | 795-802 | MAARIT VALTONEN, DAVID E. LAAKSONEN, TOMMI TOLMUNEN, KRISTIINA NYYSSÖNEN, HEIMO VIINAMÄKI, JUSSI KAUHANEN and LEO NISKANEN | Hopelessness - novel facet of the metabolic syndrome in men |
| 2008 | 36 | 8 | 803-811 | ANNA NYBERG, HUGO WESTERLUND, LINDA L. MAGNUSSON HANSON and TÖRES THEORELL | Managerial leadership is associated with self-reported sickness absence and sickness presenteeism among Swedish men and women |
| 2008 | 36 | 8 | 812-817 | DAG HOFOSS and ELLEN DEILKÅS | Roadmap for patient safety research: approaches and roadforks |
| 2008 | 36 | 8 | 818-826 | NINA KONSTANTIN NISSEN, METTE MADSEN and ANN-DORTHE OLSEN ZWISLER | Health service interventions targeting relatives of heart patients: A review of the literature |
| 2008 | 36 | 8 | 827-831 | NINA K. NISSEN, METTE MADSEN, METTE KJØLLER, SUSANNE B. WALDORFF and ANN-DORTHE OLSEN ZWISLER | Are hospitals also for relatives? A survey of hospitals' activities regarding relatives of cardiac patients |
| 2008 | 36 | 8 | 832-840 | THOMAS MILDESTVEDT, EIVIND MELAND and GEIR EGIL EIDE | How important are individual counselling, expectancy beliefs and autonomy for the maintenance of exercise after cardiac rehabilitation? |
| 2008 | 36 | 8 | 841-849 | ANDERS SVENSSON, MARK CONNOLLY, FEDERICO GALLO and LEIF HÄGGLUND | Long-term fiscal implications of subsidizing in-vitro fertilization in Sweden: A lifetime tax perspective |
| 2008 | 36 | 8 | 850-856 | MARIE KRUSE, MICHAEL DAVIDSEN, METTE MADSEN, DORTE GYRD-HANSEN and JAN SØRENSEN | Costs of heart disease and risk behaviour: Implications for expenditure on prevention |
| 2008 | 36 | 8 | 857-858 | JENNIE MEDIN and ALEXANDRA KRETTEK | An apple a day keeps the doctor away: Interdisciplinary approaches to solving major public health threats |
| 2008 | 36 | 8 | 859-869 | JULIE A. SORENSEN, JOHN MAY, RONNE OSTBY-MALLING, TOM LEHMEN, JOHN STRAND, HANS STENLUND, LARS W. EINEHALL and MARIA EMMELIN | Encouraging the installation of rollover protective structures in New York State: the design of a social marketing intervention |
| 2008 | 36 | 8 | 870-874 | PHILIPPE LUNETTA, ANTTI IMPINEN and ANNE LOUNAMAA | Underreporting of external cause codes in the Finnish Hospital Discharge Register |
| 2008 | 36 | 8 | 875-878 | BRITA KALTENBRUNNER BERNITZ | Communicable disease policy development in response to changing European political frontiers in Finland, Norway and Sweden |
| 2008 | 36 | 8 | 879-888 | KOSUKE KAWAI, SYLVIA F. KAAYA, LUSAJO KAJULA, JESSIE MBWAMBO, GAD P. KILONZO and WAFAIE W. FAWZI | Parents' and teachers' communication about HIV and sex in relation to the timing of sexual initiation among young adolescents in Tanzania |
| 2008 | 36 | 8 | 889-895 | STÅLE PALLESEN, JØRN HETLAND, BØRGE SIVERTSEN, ODDRUN SAMDAL, TORBJØRN TORSHEIM and INGER HILDE NORDHUS | Time trends in sleep-onset difficulties among Norwegian adolescents: 1983-2005 |
| 2008 | 36 | 8 | 896-897 |  | World Health Organization Commission on the Social Determinants of Health: Social injustice is killing people on a grand scale |
| 2008 | 36 | 8 | 897 |  | First World Forum Against Drugs: 100 years of global drug prevention marked in Stockholm |
| 2008 | 36 | 8 | 898 |  | First World Forum Against Drugs: 100 years of global drug prevention marked in Stockholm |
| 2009 | 37 | 1 | 1-3 | JØRN OLSEN | Mobiles phones and health |
| 2009 | 37 | 1 | 4-12 | NINA SIMONSEN-REHN, RITVA LAAMANEN, JARI SUNDELL, MATS BROMMELS and SAKARI SUOMINEN | Determinants of health promotion action in primary health care: Comparative study of health and home care personnel in four municipalities in Finland |
| 2009 | 37 | 1 | 13-19 | ÅSA BRINGSÉN, H. INGEMAR ANDERSSON and GÖRAN EJLERTSSON | Development and quality analysis of the Salutogenic Health Indicator Scale (SHIS) |
| 2009 | 37 | 1 | 20-27 | ISABEL JOHNSON, PER TILLGREN and MARIA HAGSTRÖMER | Understanding and interpreting the concept of physical activity – a focus group study among Swedish women |
| 2009 | 37 | 1 | 28-34 | ROBERTO NUEVO, VILLE LEHTINEN, PATRICIA M. REYNA-LIBERATO and JOSÉ LUIS AYUSO-MATEOS | Usefulness of the Beck Depression Inventory as a screening method for depression among the general population of Finland |
| 2009 | 37 | 1 | 35-42 | PETER SALMI, PIA SVEDBERG, JAN HAGBERG, GÖRAN LUNDH, JÜRGEN LINDER and KRISTINA ALEXANDERSON | Multidisciplinary investigations recognize high prevalence of co-morbidity of psychiatric and somatic diagnoses in long-term sickness absentees |
| 2009 | 37 | 1 | 43-49 | HÉLÈNE SANDMARK | Job mismatching, unequal opportunities and long-term sickness absence in female white-collar workers in Sweden |
| 2009 | 37 | 1 | 50-56 | LEIF SIEURIN, MALIN JOSEPHSON and EVA VINGÅRD | Positive and negative consequences of sick leave for the individual, with special focus on part-time sick leave |
| 2009 | 37 | 1 | 57-63 | ULLA GERNER and KRISTINA ALEXANDERSON | Issuing sickness certificates: A difficult task for physicians: A qualitative analysis of written statements in a Swedish survey |
| 2009 | 37 | 1 | 64-74 | ANNE KONU, PEKKA RISSANEN, MERVI IHANTOLA and REIJO SUND | "Effectiveness" in Finnish healthcare studies |
| 2009 | 37 | 1 | 75-82 | J. JONASSON, Y. LINNÉ, M. NEOVIUS and S. RÖSSNER | An Internet-based weight loss programme - a feasibility study with preliminary results from 4209 completers |
| 2009 | 37 | 1 | 83-92 | SANDER GREENLAND and LEEKA KHEIFETS | Designs and analyses for exploring the relationship of magnetic fields to childhood leukaemia: A pilot project for the Danish National Birth Cohort |
| 2009 | 37 | 1 | 93-100 | ULLA BEIJER and SVEN ANDRÉASSON | Physical diseases among homeless people: Gender differences and comparisons with the general population |
| 2009 | 37 | 1 | 101-108 | M. M. AHMED SULEIMAN, A. R. ARO and M. SODEMANN | Evaluation of tuberculosis control programme in Khartoum State for the year 2006 |
| 2009 | 37 | 1 | 110 |  | PUBLIC HEALTH CALENDAR |
| 2009 | 37 | 2 | 111-116 | BJØRN GULDVOG | Strengthening quality of care in four Nordic countries |
| 2009 | 37 | 2 | 117-121 | H. I. KARLBERG and B.-M. BRINKMO | The unethical focus on access: A study of medical ethics and the waiting-time guarantee |
| 2009 | 37 | 2 | 122-130 | FAISAL OMAR, GUSTAV TINGHÖG, PETTER TINGHÖG and PER CARLSSON | Attitudes towards priority-setting and rationing in healthcare - an exploratory survey of Swedish medical students |
| 2009 | 37 | 2 | 131-138 | KRISTIINA MANDERBACKA, MARTTI ARFFMAN, ALASTAIR LEYLAND, ALISON MCCALLUM and ILMO KESKIMÄKI | Change and persistence in healthcare inequities: Access to elective surgery in Finland in 1992-2003 |
| 2009 | 37 | 2 | 139-145 | BIBI HØLGE-HAZELTON and KIRSTI MALTERUD | Gender in medicine - does it matter? |
| 2009 | 37 | 2 | 146-152 | SADIQ M. ALI, BASILE CHAIX, JUAN MERLO, MARIA ROSVALL, SARAH WAMALA and MARTIN LINDSTRÖM | Gender differences in daily smoking prevalence in different age strata: A population-based study in southern Sweden |
| 2009 | 37 | 2 | 153-160 | KRISTIINA PATJA, SAMU M. HAKALA, GUNNELL BOSTRÖM, PAUL NORDGREN and MARGARETHA HAGLUND | Trends of tobacco use in Sweden and Finland: Do differences in tobacco policy relate to tobacco use? |
| 2009 | 37 | 2 | 161-167 | GUNNAR LUNDQVIST, HERBERT SANDSTRÖM, ANN ÖHMAN and LARS WEINEHALL | Patterns of tobacco use: A 10-year follow-up study of smoking and snus habits in a middle-aged Swedish population |
| 2009 | 37 | 2 | 168-175 | STURLA GJESDAL, PIA SVEDBERG, JAN HAGBERG and KRISTINA ALEXANDERSON | Mortality among disability pensioners in Norway and Sweden 1990-96: Comparative prospective cohort study |
| 2009 | 37 | 2 | 176-186 | MICHELLE SCHNEIDER, DEBBIE BRADSHAW, KRISELA STEYN, ROSANA NORMAN and RIA LAUBSCHER | Poverty and non-communicable diseases in South Africa |
| 2009 | 37 | 2 | 187-200 | N. SAHAL, R. REINTJES and A. R. ARO | Communicable diseases surveillance lessons learned from developed and developing countries: Literature review |
| 2009 | 37 | 2 | 201-205 | ANTON C. J. LAGER, BJÖÖRN FOSSUM, GÖRAN RÖRVALL and SVEN G. BREMBERG | Children's overweight and obesity: Local and national monitoring using electronic health records |
| 2009 | 37 | 2 | 206-219 | LAURA KESTILÄ, OSSI RAHKONEN, TUIJA MARTELIN, MARJAANA LAHTI-KOSKI and SEPPO KOSKINEN | Do childhood social circumstances affect overweight and obesity in early adulthood? |
| 2009 | 37 | 2 | 220-221 |  | News on Health Policy and Public Health |
| 2009 | 37 | 2 | 222 |  | PUBLIC HEALTH CALENDAR |
| 2009 | 37 | 3 | 223-226 | HELGE GARÅSEN and CARSTEN HENDRIKSEN | Health care for the elderly - perspectives in relation to implementation of results from intervention studies in Norway and Denmark |
| 2009 | 37 | 3 | 227-231 | ROBERT ERIKSON and JENNY TORSSANDER | Clerics die, doctors survive: A note on death risks among highly educated professionals |
| 2009 | 37 | 3 | 232-238 | ANDREJ M. GRJIBOVSKI, PER MAGNUS and CAMILLA STOLTENBERG | Decrease in consanguinity among parents of children born in Norway to women of Pakistani origin: A registry-based study |
| 2009 | 37 | 3 | 239-245 | KARI VIK, INGER MARGRETE AASS, ANNE BRITT WILLUMSEN and MARIT HAFTING | "It's about focusing on the mother's mental health": Screening for postnatal depression seen from the health visitors' perspective - a qualitative study |
| 2009 | 37 | 3 | 246-251 | CHARLOTTE OLESEN, NANA THRANE, ANN-MARGRETHE RØNHOLT, JØRN OLSEN and TINE B HENRIKSEN | Association between social position and congenital anomalies: A population-based study among 19,874 Danish women |
| 2009 | 37 | 3 | 252-259 | KAROLIINA HARKONMÄKI, PEKKA MARTIKAINEN, EERO LAHELMA, JANNE PITKÄNIEMI, TUOMO HAIJVOEENMÄKI, KARRI SILVENTOINEN and OSSI RAHKONEN | Intentions to retire, life dissatisfaction and the subsequent risk of disability retirement |
| 2009 | 37 | 3 | 260-264 | ANNA LINDBLAD, RURIK LÖFMARK and NIELS LYNÖE | Would physician-assisted suicide jeopardize trust in the medical services? An empirical study of attitudes among the general public in Sweden |
| 2009 | 37 | 3 | 265-272 | PIETRO FERRARA, ANTONIO GATTO, ALESSANDRO NICOLETTI, VALENTINA EMMANUELE, ALFONSO FASANO and VINCENZO CURRÒ | Health care of children living with their mother in prison compared with the general population |
| 2009 | 37 | 3 | 273-279 | CLAES ANDERSSON, KENT O. JOHNSSON, MATS BERGLUND and AGNETA ÖJEHAGEN | Measurement properties of the Arnetz and Hasson stress questionnaire in Swedish university freshmen |
| 2009 | 37 | 3 | 280-286 | STEPHEN HEWITT and SIDSEL GRAFF-IVERSEN | Risk factors for cardiovascular diseases and diabetes in disability pensioners aged 40-42 years: A cross-sectional study in Norway |
| 2009 | 37 | 3 | 287-294 | METTE RASMUSSEN, PERNILLE DUE, MOGENS T. DAMSGAARD and BJØRN E. HOLSTEIN | Social inequality in adolescent daily smoking: Has it changed over time? |
| 2009 | 37 | 3 | 295-303 | MARIA G. IVERSEN, KAREN S. HANSEN, MORTEN FREIL and SVEND KREINER | Do surgical patients differ in the way they prioritise aspects of hospital care? |
| 2009 | 37 | 3 | 304-309 | JIANMING WANG and HONGBING SHEN | Direct observation and completion of treatment of tuberculosis in rural areas of China |
| 2009 | 37 | 3 | 310-319 | JENNY KÖRLIN, KRISTINA ALEXANDERSON and PIA SVEDBERG | Sickness absence among women and men in the police: A systematic literature review |
| 2009 | 37 | 3 | 320-326 | INGEBORG HARTZ, ELDAR LUNDESGAARD, AAGE TVERDAL and SVETLANA SKURTVEIT | Disability pension is associated with the use of benzodiazepines 20 years later: A prospective study |
| 2009 | 37 | 3 | 327-328 | PREBEN AAVITSLAND | Abortion and depression: Cause or confounding? |
| 2009 | 37 | 3 | 329-330 | WILLY PEDERSEN | Response to Aavitsland's comments on "Abortion and depression: A population-based longitudinal study of young women" |
| 2009 | 37 | 3 | 331-332 |  | News on Health Policy and Public Health |
| 2009 | 37 | 3 | 333 |  | PUBLIC HEALTH CALENDAR |
| 2009 | 37 | 4 | 335-339 | FINN BOERLUM KRISTENSEN | Health technology assessment in Europe |
| 2009 | 37 | 4 | 340-346 | MIKA GISSLER, OSSI RAHKONEN, LAUST MORTENSEN, ANNETT ARNTZEN, SVEN CNATTINGIUS, ANNE-MARIE NYBO ANDERSEN and ELINA HEMMINKI | Sex differences in child and adolescent mortality in the Nordic countries, 1981-2000 |
| 2009 | 37 | 4 | 347-356 | BIRGIT NICLASEN and LENNART KÖHLER | National indicators of child health and well-being in Greenland |
| 2009 | 37 | 4 | 357-363 | PIA S. KIVELÄ, ANNEKE KROL, AIRI L.A. PARTANEN and MATTI A. RISTOLA | High prevalence of unprotected sex among Finnish HIV-positive and HIV-negative injecting drug users |
| 2009 | 37 | 4 | 364-371 | TONE KRINGELAND, ANNE KJERSTI DALTVEIT and ANDERS MØLLER | What characterizes women in Norway who wish to have a caesarean section? |
| 2009 | 37 | 4 | 372-379 | MARY HASSANDRA, YIANNIS THEODORAKIS, EVDOXIA KOSMIDOU, VASSILIS GRAMMATIKOPOULOS and ANTONIS HATZIGEORGIADIS | I do not smoke - I exercise: A pilot study of a new educational resource for secondary education students |
| 2009 | 37 | 4 | 380-386 | THOMAS T. PETERSEN, KIRSTEN FONAGER, HENRIK BØGGILD, LARS PEDERSEN and JENS T. MORTENSEN | Application for disability pension and change in use of prescribed drugs. A regional Danish cohort study |
| 2009 | 37 | 4 | 387-394 | STURLA GJESDAL, KJELL HAUG, PEDER RINGDAL, JOHN GUNNAR MÆELAND, JAN HAGBERG, THOMAS RØRAAS, STEIN EMIL VOLLSET and KRISTINA ALEXANDERSON | Sickness absence with musculoskeletal or mental diagnoses, transition into disability pension and all-cause mortality: A 9-year prospective cohort study |
| 2009 | 37 | 4 | 395-400 | BJØRGULF CLAUSSEN, ODD STEFFEN DALGARD and DAG BRUUSGAARD | Disability pensioning: Can ethnic divides be explained by occupation, income, mental distress, or health? |
| 2009 | 37 | 4 | 401-408 | BERIT ROSTAD, BERIT SCHEI and TOM IVAR LUND NILSEN | Social inequalities in mortality in older women cannot be explained by biological and health behavioural factors - results from a Norwegian health survey (the HUNT Study) |
| 2009 | 37 | 4 | 409-417 | SOLVEIG A. ARNADOTTIR, ELIN D. GUNNARSDOTTIR and LILLEMOR LUNDIN-OLSSON | Are rural older Icelanders less physically active than those living in urban areas? A population-based study |
| 2009 | 37 | 4 | 418-426 | M. V. GROTH, S. FAGT, A. STOCKMARR, J. MATTHIESSEN and A. BILTOFT-JENSEN | Dimensions of socioeconomic position related to body mass index and obesity among Danish women and men |
| 2009 | 37 | 4 | 427-433 | ANNA-LENA ÖSTBERG, MARIA NYHOLM, BO GULLBERG, LENNART RÅSTAM and ULF LINDBLAD | Tooth loss and obesity in a defined Swedish population |
| 2009 | 37 | 4 | 434-443 | BERNT LINDAHL, TORBJÖRN K. NILSSON, KNUT BORCH-JOHNSEN, MICHAEL E. RØDER, STEFAN SÖDERBERG, LARS WIDMAN, OWE JOHNSON, GÖRAN HALLMANS and JAN-HÅKAN JANSSON | A randomized lifestyle intervention with 5-year follow-up in subjects with impaired glucose tolerance: Pronounced short-term impact but long-term adherence problems |
| 2009 | 37 | 4 | 444-445 | Public Health Calendar — Public Health Events | News On Health Policy And Public Health |
| 2009 | 37 | 5 | 447-449 | HENRIK BRØNNUM-HANSE | Quantitative health impact assessment modelling |
| 2009 | 37 | 5 | 450-458 | PAULA HOLLAND, BO BURSTRÖM, IDA MÖLLER and MARGARET WHITEHEAD | Socioeconomic inequalities in the employment impact of ischaemic heart disease: a longitudinal record linkage study in Sweden |
| 2009 | 37 | 5 | 459-466 | KIM U. WITTRUP-JENSEN, JØRGEN LAURIDSEN, CLAIRE GUDEX and KJELD M. PEDERSEN | Generation of a Danish TTO value set for EQ-5D health states |
| 2009 | 37 | 5 | 467-474 | JAN SØRENSEN, MICHAEL DAVIDSEN, CLAIRE GUDEX, KJELD MØLLER PEDERSEN and HENRIK BRØNNUM-HANSEN | Danish EQ-5D population norms |
| 2009 | 37 | 5 | 475-480 | MAGRITT BRUSTAD, TORUNN PETTERSEN, MARITA MELHUS and EILIV LUND | Mortality patterns in geographical areas with a high vs. low Sami population density in Arctic Norway |
| 2009 | 37 | 5 | 481-486 | BRAD RODU and PHILIP COLE | Lung cancer mortality: Comparing Sweden with other countries in the European Union |
| 2009 | 37 | 5 | 487-493 | MATS SUNDBECK, MATHIAS GRAHN, VINCENT LÖNNGREN, NILS OVE MÅNSSON, LENNART RÅSTAM and ULF LINDBLAD | Snuff use associated with abdominal obesity in former smokers |
| 2009 | 37 | 5 | 494-502 | OUTI SIMONEN, ELINA VIITANEN, ANNE KONU and MARJA BLOM | Effectiveness in political-administrative decision-making in specialized healthcare |
| 2009 | 37 | 5 | 503-508 | JUDITH ROSTA, MAGNE NYLENNA and OLAF G. AASLAND | Job satisfaction among hospital doctors in Norway and Germany. A comparative study on national samples |
| 2009 | 37 | 5 | 509-517 | CAMILLA PETTERSSON, MARGARETA LINDEN-BOSTRÖM and CHARLI ERIKSSON | Parental attitudes and behaviour concerning adolescent alcohol consumption: do sociodemographic factors matter? |
| 2009 | 37 | 5 | 518-524 | TAMANNA FERDOUS, TOMMY CEDERHOLM, ABDUR RAZZAQUE, ÅKE WAHLIN and ZARINA NAHAR KABIR | Nutritional status and self-reported and performance-based evaluation of physical function of elderly persons in rural Bangladesh |
| 2009 | 37 | 5 | 525-531 | MICHAL BRONIKOWSKI and MALGORZATA BRONIKOWSKA | Salutogenesis as a framework for improving health resources of adolescent boys |
| 2009 | 37 | 5 | 532-544 | FRIDA EEK and P.-O. ÖSTERGREN | Factors associated with BMI change over five years in a Swedish adult population. Results from the Scania Public Health Cohort Study |
| 2009 | 37 | 5 | 545-553 | BENNO KRACHLER, MATS ELIASSON, HANS STENLUND, INGEGERD JOHANSSON, GÖRAN HALLMANS and BERNT LINDAHL | Population-wide changes in reported lifestyle are associated with redistribution of adipose tissue |
| 2009 | 37 | 5 | 554-555 |  | News on Health Policy and Public Health |
| 2009 | 37 | 5 | 556-557 |  | Acknowledgements |
| 2009 | 37 | 5 | 558 |  | Public Health Calendar — Public Health Events |
| 2009 | 37 | 6 | 559-561 | GUNNAR TELLNES | How can nature and culture promote health? |
| 2009 | 37 | 6 | 562-568 | TORILL BULL and MAURICE B. MITTELMARK | Work life and mental wellbeing of single and non-single working mothers in Scandinavia |
| 2009 | 37 | 6 | 569-576 | POUL FROST, JENS P. HAAHR and JOHAN H. ANDERSEN | Impact of work, health and health beliefs on new episodes of pain-related and general absence-taking |
| 2009 | 37 | 6 | 577-583 | BJARNE LAURSEN and HANNE MØLLER | Unintentional injuries in children of Danish and foreign-born mothers |
| 2009 | 37 | 6 | 584-589 | LIV F. HEKTOEN, ELINE AAS and HILDE LURÅS | Cost-effectiveness in fall prevention for older women |
| 2009 | 37 | 6 | 590-597 | BJØRGULF CLAUSSEN and ODD STEFFEN DALGARD | Disability pensioning: The gender divide can be explained by occupation, income, mental distress and health |
| 2009 | 37 | 6 | 598-603 | BJÖRN LANTDSTRÖM, BENGT MATTSSON and CARL EDVARD RUDEBECK | Attributes of competence – on GPs' work performance in daily practice |
| 2009 | 37 | 6 | 604-612 | MERETE LAUBJERG, ANNE MAJ CHRISTENSEN and BIRGIT PETERSSON | Psychiatric status among stepchildren and domestic and international adoptees in Denmark. A comparative nationwide register-based study |
| 2009 | 37 | 6 | 613-620 | ANNE-LISE TAMBER and DAG BRUUSGAARD | Self-reported faintness or dizziness – comorbidity and use of medicines. An epidemiological study |
| 2009 | 37 | 6 | 621-626 | SINE SKOVBJERG, STIG BRORSON, ALICE RASMUSSEN, JEANNE DUUS JOHANSEN and JESPER ELBERLING | Impact of self-reported multiple chemical sensitivity on everyday life: A qualitative study |
| 2009 | 37 | 6 | 627-631 | SIRI NAESS, JOHN ERIKSEN and KRISTIAN TAMBS | Perceived change in life satisfaction following epilepsy diagnosis |
| 2009 | 37 | 6 | 632-639 | ANDREW STICKLEY and PER CARLSON | The social and economic determinants of smoking in Moscow, Russia |
| 2009 | 37 | 6 | 640-646 | LISE LUND HÅHEIM, PER NAFSTAD, INGAR OLSEN, PER SCHWARZE and KJERSTI S. RØNNINGEN | C-reactive protein variations for different chronic somatic disorders |
| 2009 | 37 | 6 | 647-653 | B. SEMARK, K. FREDLUND, B. ÅSTRAND and L. BRUDIN | Reimbursement for drugs – a register study comparing economic outcome for five healthcare centres in areas with different socioeconomic conditions |
| 2009 | 37 | 6 | 650-660 | JENS BONKE and KAREN BORREGAARD | The prevalence of problematic gambling behaviour: A Scandinavian comparison |
| 2009 | 37 | 6 | 661-663 | OLA EKHOLM and HENRIK BRØNNUM-HANSEN | Cross-national comparisons of non-harmonized indicators may lead to more confusion than clarification |
| 2009 | 37 | 6 | 664-667 | JOACHIM SCHÜZ | Lost in laterality: interpreting "preferred side of the head during mobile phone use and risk of brain tumour" associations |
| 2009 | 37 | 6 | 668-669 |  | News on Health Policy and Public Health |
| 2009 | 37 | 6 | 670 |  | Public Health Calendar — Public Health Events |
| 2009 | 37 | 7 | 671-673 | ELINA HEMMINKI | Perinatal health in the Nordic countries - Current challenges |
| 2009 | 37 | 7 | 674-681 | LISBETH SMEBY, DAG BRUUSGAARD and BJØRGULF CLAUSSEN | Sickness absence: Could gender divide be explained by occupation, income, mental distress and health? |
| 2009 | 37 | 7 | 682-689 | HANS JØRGEN SØGAARD and PER BECH | Psychiatric disorders in long-term sickness absence - a population-based cross-sectional study |
| 2009 | 37 | 7 | 690-696 | ELIN THYGESEN, TORILL CHRISTINE LINDSTROM, HANS INGE SAEVAREID and KNUT ENGEDAL | The Subjective Health Complaints Inventory: A useful instrument to identify various aspects of health and ability to cope in older people? |
| 2009 | 37 | 7 | 697-705 | IGOR VAN LAERE, MATTY DE WIT and NIEK KLAZINGA | Preventing evictions as a potential public health intervention: Characteristics and social medical risk factors of households at risk in Amsterdam |
| 2009 | 37 | 7 | 706-712 | ANN BLOMSTRAND, CECILIA BJÖRKELUND, NASHMIL ARIAI, LAUREN LISSNER and CALLE BENGTSSON | Effects of leisure-time physical activity on well-being among women: a 32-year perspective |
| 2009 | 37 | 7 | 713-719 | CLAES ANDERSSON, KENT O JOHNSSON, MATS BERGLUND and AGNETA ÖJEHAGEN | Stress and hazardous alcohol use: Associations with early dropout from university |
| 2009 | 37 | 7 | 720-727 | KRISTIINA RAJALEID, JOHAN HALLQVIST and ILONA KOUPIL | The effect of early life factors on 28 day case fatality after acute myocardial infarction |
| 2009 | 37 | 7 | 728-735 | LOTTE EVRON, KIRSTEN SCHULTZ-LARSEN and TINE FRISTRUP | Barriers to participation in a hospital-based falls assessment clinic programme: an interview study with older people |
| 2009 | 37 | 7 | 736-743 | METTE WAADDEGAARD, MICHAEL DAVIDSEN and METTE KJØLLER | Obesity and prevalence of risk behaviour for eating disorders among young Danish women |
| 2009 | 37 | 7 | 744-750 | WENCHE MALMEDAL, ANDI HAMMERVOLD and BRITT-INGER SAVEMAN | To report or not report? Attitudes held by Norwegian nursing home staff on reporting inadequate care carried out by colleagues |
| 2009 | 37 | 7 | 751-757 | BJØRG HJERKINN, ELIN O ROSVOLD and MORTEN LINDBÆK | Neonatal findings among children of substance-abusing women attending a special child welfare clinic in Norway |
| 2009 | 37 | 7 | 758-765 | OLA EKHOLM, ULRIK HESSE, MICHAEL DAVIDSEN and METTE KJØLLER | The study design and characteristics of the Danish national health interview surveys |
| 2009 | 37 | 7 | 766-773 | WEI-QING CHEN, TZE-WAI WONG and TAK-SUN YU | Influence of occupational stress on mental health among Chinese off-shore oil workers |
| 2009 | 37 | 7 | 774-777 | DEBORAH L REAS, JAN F NYGÅRD and TOM SØRENSEN | Do quitters have anything to lose? Changes in body mass index for daily, never, and former smokers over an 11-year period (1990-2001) |
| 2009 | 37 | 7 | 778-780 | MERETE LABRIOLA, HELENE FEVEILE, KARL BANG CHRISTENSEN, UTE BÜLTMANN and THOMAS LUND | The impact of job satisfaction on the risk of disability pension. A 15-year prospective study |
| 2009 | 37 | 7 | 781-782 |  | News on Health Policy and Public Health |
| 2009 | 37 | 8 | 783-784 | URBAN JANLERT | Economic crisis, unemployment and public health |
| 2009 | 37 | 8 | 785-792 | PIA V. PEDERSEN, METTE KJØLLER, OLA EKHOLM, MORTEN GRØNBÆK and TINE CURTIS | Readiness to change level of physical activity in leisure time among physically inactive Danish adults |
| 2009 | 37 | 8 | 793-800 | PAULINA NOWICKA, JAN LANKE, ANGELO PIETROBELLI, ERWIN APITZSCH and CARL-ERIK FLODMARK | Sports camp with six months of support from a local sports club as a treatment for childhood obesity |
| 2009 | 37 | 8 | 801-819 | MARKUS GERBER and UWE PÜHSE | Do exercise and fitness protect against stress-induced health complaints? A review of the literature |
| 2009 | 37 | 8 | 820-825 | SILLE OHREM NAPER | All-cause and cause-specific mortality of social assistance recipients in Norway: A register-based follow-up study |
| 2009 | 37 | 8 | 826-838 | CARLES MUNTANER, CARME BORRELL, JUDIT SOLÀ, MARC MARÍ-DELL'OLMO, HAEJOO CHUNG, MAICA RODRÍGUEZ-SANZ, JOAN BENACH and SAMUEL NOH | Capitalists, managers, professionals and mortality: Findings from the Barcelona Social Class and All Cause Mortality Longitudinal Study |
| 2009 | 37 | 8 | 839-845 | ANNA BRYNGELSON | Long-term sickness absence and social exclusion |
| 2009 | 37 | 8 | 846-854 | AINO SALONSALMI, MIKKO LAAKSONEN, EERO LAHELMA and OSSI RAHKONEN | Drinking habits and sickness absence: The contribution of working conditions |
| 2009 | 37 | 8 | 855-863 | HANS JØRGEN SØGAARD and PER BECH | Psychometric analysis of Common Mental Disorders - Screening Questionnaire (CMD-SQ) in long-term sickness absence |
| 2009 | 37 | 8 | 864-871 | HANS JØRGEN SØGAARD and PER BECH | The effect on length of sickness absence by recognition of undetected psychiatric disorder in long-term sickness absence. A randomized controlled trial |
| 2009 | 37 | 8 | 872-880 | HANS J. SØGAARD | Choosing screening instrument and cut-point on screening instruments. A comparison of methods |
| 2009 | 37 | 8 | 881-889 | SIGRID BJORNELV, STIAN LYDERSEN, JOSTEIN HOLMEN, TOM IVAR LUND NILSEN and TURID LINGAAS HOLMEN | Sex differences in time trends for overweight and obesity in adolescents: The Young-HUNT study |
| 2009 | 37 | 8 | 890-892 |  | News on Health Policy and Public Health |
| 2009 | 37 | 8 | 893 |  | Public Health Calendar — Public Health Events |
| 2010 | 38 | 1 | 1-8 | ULRIKA LINDMARK, ULF STENSTRÖM, ELISABETH WÄRNBERG GERDIN and ANDERS HUGOSON | The distribution of "sense of coherence" among Swedish adults: A quantitative cross-sectional population study |
| 2010 | 38 | 1 | 9-16 | CAMILLA B. LAAKSONEN, MINNA E. AROMAA, RIITTA E. ASANTI, OLLI J. HEINONEN, LEENA K. KOIVUSILTA, PASI J. KOSKI, SAKARI B. SUOMINEN, TERO J. VAHLBERG and SANNA SALANTERÄ | The change in child self-assessed and parent proxy-assessed Health Related Quality of Life (HRQL) in early adolescence (age 10-12) |
| 2010 | 38 | 1 | 17-24 | TOVE NYSTAD, MARITA MELHUS, MAGRITT BRUSTAD and EILIV LUND | Ethnic differences in the prevalence of general and central obesity among the Sami and Norwegian populations: The SAMINOR study |
| 2010 | 38 | 1 | 25-31 | GUNILLA E. KULLA, SIRKKA-LIISA EKMAN, KRISTIINA HEIKKILÄ and ANNELI M. SARVIMÄKI | Differences in self-rated health among older immigrants — A comparison between older Finland-Swedes and Finns in Sweden |
| 2010 | 38 | 1 | 32-39 | CLAES A. HERLITZ and MARGARETA FORSBERG | Sexual behaviour and risk assessment in different age cohorts in the general population of Sweden (1989-2007) |
| 2010 | 38 | 1 | 40-45 | LAUST H. MORTENSEN, JØRGEN T. LAURIDSEN, FINN DIDERICHSEN, GEORGE A. KAPLAN, MIKA GISSLER and ANNE-MARIE N. ANDERSEN | Income-related and educational inequality in small-for-gestational age and preterm birth in Denmark and Finland 1987-2003 |
| 2010 | 38 | 1 | 46-52 | AARO TUPASELA, SINIKKA SIHVO, KAROLIINA SNELL, PIIA JALLINOJA, ARJA R. ARO and ELINA HEMMINKI | Attitudes towards biomedical use of tissue sample collections, consent, and biobanks among Finns |
| 2010 | 38 | 1 | 53-63 | MARIA MÜLLERSDORF, FREDRIK GRANSTRÖM, LOTTA SAHLQVIST and PER TILLGREN | Aspects of health, physical/leisure activities, work and socio-demographics associated with pet ownership in Sweden |
| 2010 | 38 | 1 | 64-70 | MERETE LAUBJERG and BIRGIT PETERSSON | Greenlandic adoptees' psychiatric inpatient contact. A comparative register-based study |
| 2010 | 38 | 1 | 71-77 | BEATA HUBICKA, HANS LAURELL and HANS BERGMAN | Psychosocial characteristics of drunk drivers assessed by the Addiction Severity Index, prediction of relapse |
| 2010 | 38 | 1 | 78-85 | ANDERS HANSSON, TOBIAS ARVEMO, BERTIL MARKLUND, BIRGITTA GEDDA and BENGT MATTSSON | Working together - primary care doctors' and nurses' attitudes to collaboration |
| 2010 | 38 | 1 | 86-94 | DIDIER JOURDAN, JEANINE POMMIER and FRÉDÉRIQUE QUIDU | Practices and representations of health education among primary school teachers |
| 2010 | 38 | 1 | 95-103 | AYAGA A. BAWAH, JAMES F. PHILLIPS, MARTIN ADJUIK, MAYA VAUGHAN-SMITH, BRUCE MACLEOD and FRED N. BINKA | The impact of immunization on the association between poverty and child survival: Evidence from Kassena-Nankana District of northern Ghana |
| 2010 | 38 | 1 | 104-107 | G. GULIS, M. L. ERIKSEN and A. R. ARO | Public health research in Denmark in the years 1995-2005 |
| 2010 | 38 | 1 | 108-109 | KIRSTI KÄHÄRÄ, JUHANI GRÖNLUND and KARI J. MATTILA | Symptoms of adolescent mental health problems and public concern |
| 2010 | 38 | 1 | 110-111 |  | News on Health Policy and Public Health |
| 2010 | 38 | 1 | 112 |  | Public Health Calendar — Public Health Events |
| 2010 | 38 | 2 | 113-114 | FINN KAMPER-JØRGENSEN | The healthy city |
| 2010 | 38 | 2 | 115-120 | IDA KNUTSSON and ANITA LINELL | Health impact assessment developments in Sweden |
| 2010 | 38 | 2 | 121-128 | CHRISTINA BJØRK PETERSEN, LAU CASPAR THYGESEN, JØRN WULFF HELGE, MORTEN GRØNBÆK and JANNE SCHURMANN TOLSTRUP | Time trends in physical activity in leisure time in the Danish population from 1987 to 2005 |
| 2010 | 38 | 2 | 129-134 | KERSTI PÄRNA and INGE RINGMETS | Comparison of socioeconomic differences in self-perceived health in Estonia and Finland |
| 2010 | 38 | 2 | 135-140 | BERIT SCHEI and HEIN STIGUM | A study of men who pay for sex, based on the Norwegian National Sex Surveys |
| 2010 | 38 | 2 | 141-150 | CHARLOTTE L. DEOGAN, MARTA K. HANSSON BOCANGEL, SARAH P. WAMALA and ANNA M. MÅNSDOTTER | A cost-effectiveness analysis of the Chlamydia Monday - A community-based intervention to decrease the prevalence of chlamydia in Sweden |
| 2010 | 38 | 2 | 151-159 | ÅSA ROME, ULF PERSSON, CHARLOTTE EKDAHL and GUNVOR GARD | Willingness to pay for health improvements of physical activity on prescription |
| 2010 | 38 | 2 | 160-167 | GUDRUN GUDMUNDSDOTTIR and RUNAR VILHJALMSSON | Group differences in outpatient help-seeking for psychological distress: Results from a national prospective study of Icelanders |
| 2010 | 38 | 2 | 168-176 | K. APPELQVIST-SCHMIDLECHNER, M. UPANNE, M. HENRIKSSON, K. PARKKOLA and E. STENGÅRD | Young men exempted from compulsory military or civil service in Finland - A group of men in need of psychosocial support? |
| 2010 | 38 | 2 | 177-183 | PALLE BO PALLESEN, TORILL TVERBORGVIK, HANNA BARBARA RASMUSSEN and ELSEBETH LYNGE | Data on education: From population statistics to epidemiological research |
| 2010 | 38 | 2 | 184-191 | MIKAEL SVENSSON and CURT HAGQUIST | Adolescent alcohol and illicit drug use among first- and second-generation immigrants in Sweden |
| 2010 | 38 | 2 | 192-199 | NINA ØSTERÅS, PÅL GULBRANDSEN, INGER CATHRINE KANN and SØREN BRAGE | Structured functional assessments in general practice increased the use of part-time sick leave: A cluster randomised controlled trial |
| 2010 | 38 | 2 | 200-207 | MARIA NORDIN and ROBERT M. KAPLAN | Sleep discontinuity and impaired sleep continuity affect transition to and from obesity over time: Results from the Alameda County Study |
| 2010 | 38 | 2 | 208-219 | ULRIK SCHIØLER KESMODEL, METTE UNDERBJERG, TINA RØNDRUP KILBURN, LEIV BAKKETEIG, ERIK LYKKE MORTENSEN, NILS INGE LANDRØ, DIANA SCHENDEL, JACQUELYN BERTRAND, JAKOB GROVE, SHAHUL EBRAHIM and POUL THORSEN | Lifestyle during pregnancy: Neurodevelopmental effects at 5 years of age. The design and implementation of a prospective follow-up study |
| 2010 | 38 | 2 | 220-221 |  | News on Health Policy and Public Health |
| 2010 | 38 | 2 | 222-223 |  | Acknowledgements |
| 2010 | 38 | 2 | 224 |  | Public Health Calendar — Public Health Events |
| 2010 | 38 | 3 | 225-231 | ROBERT IRESTIG, KRISTINA BURSTRÖM, MAJA WESSEL and NIELS LYNÖE | How are homeless people treated in the healthcare system and other societal institutions? Study of their experiences and trust |
| 2010 | 38 | 3 | 232-238 | B. SAHLBERG, G. WIESLANDER and D. NORBÄCK | Sick building syndrome in relation to domestic exposure in Sweden – A cohort study from 1991 to 2001 |
| 2010 | 38 | 3 | 239-245 | HANNE GIVER, ANNE FABER, HARALD HANNERZ, JESPER STRØYER and REINER RUGULIES | Psychological well-being as a predictor of dropout among recently qualified Danish eldercare workers |
| 2010 | 38 | 3 | 246-252 | PERNILLE TANGGAARD ANDERSEN and JENS-JØRGEN JENSEN | Healthcare reform in Denmark |
| 2010 | 38 | 3 | 253-258 | OLAF G. AASLAND, JUDITH ROSTA and MAGNE NYLENNA | Healthcare reforms and job satisfaction among doctors in Norway |
| 2010 | 38 | 3 | 259-265 | JURGITA BUIVYDIENE, LIUDVIKA STARKIENE and KASTYTIS SMIGELSKAS | Healthcare reform in Lithuania: Evaluation of changes in human resources and infrastructure |
| 2010 | 38 | 3 | 266-274 | ANNE M. KOPONEN, RITVA LAAMANEN, NINA SIMONSEN-REHN, JARI SUNDELL, MATS BROMMELS and SAKARI SUOMINEN | Job involvement of primary healthcare employees: Does a service provision model play a role? |
| 2010 | 38 | 3 | 275-282 | INGUNN HARSTAD, EINAR HELDAL, SIGURD L. STEINSHAMN, HELGE GARÅSEN, BRITA A. WINJE and GEIR W. JACOBSEN | Screening and treatment of latent tuberculosis in a cohort of asylum seekers in Norway |
| 2010 | 38 | 3 | 283-290 | METTE SAGBAKKEN, GUNNAR A. BJUNE and JAN C. FRICH | Experiences of being diagnosed with tuberculosis among immigrants in Norway – Factors associated with diagnostic delay: A qualitative study |
| 2010 | 38 | 3 | 291-298 | JORLAUG HEIMISDOTTIR, RUNAR VILHJALMSSON, GUDRUN KRISTJANSDOTTIR and DAN WOLF MEYROWITSCH | The social context of drunkenness in mid-adolescence |
| 2010 | 38 | 3 | 299-308 | CHRISTIAN D. G. STOLTENBERG and PEDER G. SKOV | Determinants of return to work after long-term sickness absence in six Danish municipalities |
| 2010 | 38 | 3 | 309-316 | PEKKA VIRTANEN, JUSSI VAHTERA and CLAS-HÅKAN NYGÅRD | Locality differences of sickness absence in the context of health and social conditions of the inhabitants |
| 2010 | 38 | 3 | 317-324 | TUULIKKI VEHKO, KRISTIINA MANDERBACKA, MARTTI ARFFMAN, REIJO SUND, ANTTI REUNANEN and ILMO KESKIMÄKI | Changing patterns of secondary preventive medication among newly diagnosed coronary heart disease patients with diabetes in Finland: A register-based study |
| 2010 | 38 | 3 | 325-331 | NIKLAS EKERSTAD, RURIK LÖFMARK and PER CARLSSON | Elderly people with multi-morbidity and acute coronary syndrome: Doctors' views on decision-making |
| 2010 | 38 | 3 | 332-333 | MARIA ROSARIA GALANTI, PETER ALLEBECK, CECILIA MAGNUSSON and GUNILLA BOLINDER | Use of snus and lung cancer mortality: Unwarranted claim of causal association |
| 2010 | 38 | 3 | 334-335 |  | News on Health Policy and Public Health |
| 2010 | 38 | 3 | 336 |  | Public Health Calendar — Public Health Events |
| 2010 | 38 | 4 | 337-343 | RENÉE FLACKING, FIONA DYKES and UWE EWALD | The influence of fathers' socioeconomic status and paternity leave on breastfeeding duration: A population-based cohort study |
| 2010 | 38 | 4 | 344-350 | ULRIKA ERIKSSON and EVA SELLSTRÖM | School demands and subjective health complaints among Swedish schoolchildren: A multilevel study |
| 2010 | 38 | 4 | 351-358 | KATRINA NORDYKE, ANNA MYLÉUS, ANNELI IVARSSON, ANNELIE CARLSSON, LARS DANIELSSON, LOTTA HÖGBERG, EVA KARLSSON and MARIA EMMELIN | How do children experience participating in a coeliac disease screening? A qualitative study based on children's written narratives |
| 2010 | 38 | 4 | 359-367 | EIVIND MELAND, JAN HENRIK RYDNING, STIAN LOBBEN, HANS-JOHAN BREIDABLIK and TOR-JOHAN EKELAND | Emotional, self-conceptual, and relational characteristics of bullies and the bullied |
| 2010 | 38 | 4 | 368-374 | BERNDT STENBERG, BIRGITTA MEDING and ÅKE SVENSSON | Dermatology in public health - a model for surveillance of common skin diseases |
| 2010 | 38 | 4 | 375-385 | HANS JØRGEN SØGAARD and PER BECH | Predictive validity of common mental disorders screening questionnaire as a screening instrument in long term sickness absence |
| 2010 | 38 | 4 | 386-394 | RAIMUNDAS LUNEVICIUS, KENT A. STEVENS, PRASANTHI PUVANACHANDRA and ADNAN A. HYDER | The epidemiology of injury in the Republic of Lithuania |
| 2010 | 38 | 4 | 395-403 | PEETER VÄRNIK, MERIKE SISASK, AIRI VÄRNIK, ANDRIY YUR'YEV, KAIRI KÕLVES, LAURI LEPPIK, ALEKSANDER NEMTSOV and DANUTA WASSERMAN | Massive increase in injury deaths of undetermined intent in ex-USSR Baltic and Slavic countries: Hidden suicides? |
| 2010 | 38 | 4 | 404-410 | ANDREW STICKLEY and PER CARLSON | Factors associated with non-lethal violent victimization in Sweden in 2004-2007 |
| 2010 | 38 | 4 | 411-417 | ULRIKA K. STIGSDOTTER, OLA EKHOLM, JASPER SCHIPPERIJN, METTE TOFTAGER, FINN KAMPER-JØRGENSEN and THOMAS B. RANDRUP | Health promoting outdoor environments - Associations between green space, and health, health-related quality of life and stress based on a Danish national representative survey |
| 2010 | 38 | 4 | 418-425 | S. LÖFGREN, G. LJUNGGREN and M. BROMMELS | No ticking time bomb: Hospital utilisation of 28,528 hip fracture patients in Stockholm during 1998-2007 |
| 2010 | 38 | 4 | 426-433 | ULLA A. HVIDTFELDT, METTE GERSTER, LISBETH B. KNUDSEN and NIELS KEIDING | Are low Danish fertility rates explained by changes in timing of births? |
| 2010 | 38 | 4 | 432-441 | M. RIITTA LESKINEN, TIINA K. M. LAATIKAINEN, JAAKKO O. I. TUOMILEHTO, M. AULIKKI NISSINEN, MARKKU PELTONEN, PENTTI O. I. KOISTINEN and RIITTA L. ANTIKAINEN | Trends in health status and the use of services among the 80-84-year-old Finnish war veterans during 1992 and 2004 |
| 2010 | 38 | 4 | 442-443 |  | News on Health Policy and Public Health |
| 2010 | 38 | 4 | 444 |  | Public Health Calendar — Public Health Events |
| 2010 | 38 | 5 | 445-456 | BENTE TRÆEN and HEIN STIGUM | Sexual problems in 18-67-year-old Norwegians |
| 2010 | 38 | 5 | 457-464 | MALIN ANDRÉ, LAUREN LISSNER, CALLE BENGTSSON, TORE HÄLLSTRÖM, VALTER SUNDH and CECILIA BJÖRKELUND | Cohort differences in personality in middle-aged women during a 36-year period. Results from the Population Study of Women in Gothenburg |
| 2010 | 38 | 5 | 465-473 | ANNE M. HAUSKEN, KARI FURU, AAGE TVERDAL and SVETLANA SKURTVEIT | Mental distress and subsequent use of anxiolytic drugs – A prospective population-based cohort study of 16,000 individuals |
| 2010 | 38 | 5 | 474-480 | VIBEKE KOUSHEDE, BJØRN E. HOLSTEIN, ANETTE ANDERSEN, OLA EKHOLM and EBBA H. HANSEN | Use of over-the-counter analgesics is associated with perceived stress among 25-44-year-olds: A national cross-sectional study |
| 2010 | 38 | 5 | 481-488 | PEDER A. HALVORSEN, RANDI M. SELMER and IVAR S. KRISTIANSEN | Anticipated longevity among lay people screened for cardiovascular risk factors: A cross-sectional questionnaire study |
| 2010 | 38 | 5 | 489-494 | ANNE S. TOLSTRUP, JANE L. HANSEN, MORTEN GRØNBÆK, ULLA VOGEL, ANNE TJØNNELAND, ALBERT MARNI JOENSEN and KIM OVERVAD | Alcohol drinking habits, alcohol dehydrogenase genotypes and risk of acute coronary syndrome |
| 2010 | 38 | 5 | 495-501 | MYRIAM KHLAT and NEARKASEN CHAU | Social disparities in musculoskeletal disorders and associated mental malaise: Findings from a population-based survey in France |
| 2010 | 38 | 5 | 502-507 | DORTHE S. NIELSEN, BENTE L. LANGDAHL, OLE H. SØRENSEN, HENRIK A. SØRENSEN and KIM T. BRIXEN | Persistence to medical treatment of osteoporosis in women at three different clinical settings – A historical cohort study |
| 2010 | 38 | 5 | 508-517 | HEGE BØEN, ODD STEFFEN DALGARD, RUNE JOHANSEN and ERIK NORD | Socio-demographic, psychosocial and health characteristics of Norwegian senior centre users: A cross-sectional study |
| 2010 | 38 | 5 | 518-523 | PIA HEILMANN | To have and to hold: Personnel shortage in a Finnish healthcare organisation |
| 2010 | 38 | 5 | 524-532 | CHRISTIAN FAERGEMANN, JENS M. LAURITSEN, OLE BRINK and PREBEN BO MORTENSEN | Do repeat victims of interpersonal violence have different demographic and socioeconomic characters from non-repeat victims of interpersonal violence and the general population? A population-based case-control study |
| 2010 | 38 | 5 | 533-540 | MARIA ROSVALL, HENRIK OHLSSON, OLE HANSEN, BASILE CHAIX and JUAN MERLO | Auditing patient registration in the Swedish quality register for acute coronary syndrome |
| 2010 | 38 | 5 | 541-547 | JERRY LARSSON, EVA ESBJÖRNSSON, ANN BJÖRKDAHL, INGRID MORBERG, MICHAEL NILSSON and KATHARINA S. SUNNERHAGEN | Sick leave after traumatic brain injury: The person or the diagnosis – Which has greater impact? |
| 2010 | 38 | 5 | 548-552 | BRIAN KØSTER, CAMILLA THORGAARD, ANJA PHILIP and INGE H. CLEMMENSEN | Prevalence of sunburn and sun-related behaviour in the Danish population: A cross-sectional stud |
| 2010 | 38 | 5 | 553-554 | CHARLOTTE HEDBERG, INGEMAR ENGSTRÖM, RENEE VICKHOFF and NIELS LYNÖE | Can evidence-based medicine become counter-productive? |
| 2010 | 38 | 5 | 555-559 |  | News on Health Policy and Public Health |
| 2010 | 38 | 5 | 560 |  | Public Health Calendar — Public Health Events |
| 2010 | 38 | 6 | 561-564 | PER CARLSSON | Priority setting in health care: Swedish efforts and experiences |
| 2010 | 38 | 6 | 565 | Finn Kamper-Jørgensen | Important message to all authors from the Chief Editor |
| 2010 | 38 | 6 | 566-573 | AGNETA ANDERSSON, KRISTINA LINDWALL SUNDEL, ANNA-LENA UNDÉN, KARIN SCHENCK-GUSTAFSSON and INGEBORG ERIKSSON | A five-year rehabilitation programme for younger women after a coronary event reduces the need for hospital care |
| 2010 | 38 | 6 | 574-579 | M. BRATTWALL, M. WARRÉN STOMBERG, N. RAWAL, M. SEGERDAHL, E. HOULTZ and J. JAKOBSSON | Patient assessed health profile: A six-month quality of life questionnaire survey after day surgery |
| 2010 | 38 | 6 | 580-586 | RICCARDO N. CANIATO, MARLIES E. ALVARENGA, HERIBERT L. STICH, HOLGER JANSEN and BERHARD T. BAUNE | Kindergarten attendance may reduce developmental impairments in children: Results from the Bavarian Pre-School Morbidity Survey |
| 2010 | 38 | 6 | 587-596 | LENA M. HANSSON, ERIK NÄSLUND and FINN RASMUSSEN | Perceived discrimination among men and women with normal weight and obesity. A population-based study from Sweden |
| 2010 | 38 | 6 | 597-604 | ÅNEN RINGARD | Why do general practitioners abandon the local hospital? An analysis of referral decisions related to elective treatment |
| 2010 | 38 | 6 | 605-610 | TOMAS FARESJÖ and MIKAEL RAHMQVIST | Educational level is a crucial factor for good perceived health in the local community |
| 2010 | 38 | 6 | 611-617 | MARIE KRUSE, JAN SØRENSEN, HENRIK BRØNNUM-HANSEN and KARIN HELWEG-LARSEN | Identifying victims of violence using register-based data |
| 2010 | 38 | 6 | 618-624 | SILJE ENDRESEN REME and HEGE R. ERIKSEN | Is one question enough to screen for depression? |
| 2010 | 38 | 6 | 625-632 | HANS JØRGEN SØGAARD and PER BECH | Compensating for non-response in a study estimating the incidence of mental disorders in long-term sickness absence by a two-phased design |
| 2010 | 38 | 6 | 633-638 | MARC NOCON, FALK MÜLLER-RIEMENSCHNEIDER, KATLEEN NITZSCHKE and STEFAN N. WILLICH | Increasing physical activity with point-of-choice prompts – a systematic review |
| 2010 | 38 | 6 | 639-647 | GIEDRIUS VANAGAS, ŽILVINAS PADAIGA, JUOZAS KURTINAITIS and ŽENETA LOGMINIENĖ | Cost-effectiveness of 12- and 15-year-old girls' human papillomavirus 16/18 population-based vaccination programmes in Lithuania |
| 2010 | 38 | 6 | 648-656 | MATHILDE VINTHER-LARSEN, METTE RIEGELS, MORTEN HULVEJ ROD, MICHAELA SCHIØTZ, TINE CURTIS and MORTEN GRØNBÆK | The Danish Youth Cohort: Characteristics of participants and non-participants and determinants of attrition |
| 2010 | 38 | 6 | 657-663 | PIA SVEDBERG, PETER SALMI, JAN HAGBERG, GÖRAN LUNDH, JÜRGEN LINDER and KRISTINA ALEXANDERSON | Does multidisciplinary assessment of long-term sickness absentees result in modification of sick-listing diagnoses? |
| 2010 | 38 | 6 | 664-669 | KIRSTEN KAYA ROESSLER | Exercise treatment for drug abuse – A Danish pilot study |
| 2010 | 38 | 6 | 670-671 | JEFFREY V. LAZARUS, SAMANTHA A. WALLACE and JERKER LILJESTRAND | Improving African health research capacity |
| 2010 | 38 | 6 | 672 |  | Public Health Calendar — Public Health Events |
| 2010 | 38 | 7 | 673-677 | MATS BROMMELS | Management and medicine: Odd couple no more. Bonding through medical management research |
| 2010 | 38 | 7 | 678-685 | LINDA ERNSTSEN, OTTAR BJERKESET and STEINAR KROKSTAD | Educational inequalities in ischaemic heart disease mortality in 44,000 Norwegian women and men: The influence of psychosocial and behavioural factors. The HUNT Study |
| 2010 | 38 | 7 | 686-690 | DAG BRUUSGAARD, LISBETH SMEBY and BJØRGULF CLAUSSEN | Education and disability pension: A stronger association than previously found |
| 2010 | 38 | 7 | 691-698 | ERJA FORSSAS, MARTTI ARFFMAN, SEPPO KOSKINEN, ANTTI REUNANEN and ILMO KESKIMÄKI | Socioeconomic differences in mortality among diabetic people in Finland |
| 2010 | 38 | 7 | 699-706 | OLA EKHOLM, JENS GUNDGAARD, NIELS K.R. RASMUSSEN and EBBA HOLME HANSEN | The effect of health, socio-economic position, and mode of data collection on non-response in health interview surveys |
| 2010 | 38 | 7 | 707-714 | LISE G. ANDERSEN, MOGENS GROENVOLD, TORBEN JØRGENSEN and METTE AADAHL | Construct validity of a revised Physical Activity Scale and testing by cognitive interviewing |
| 2010 | 38 | 7 | 715-723 | RITVA LAAMANEN, NINA SIMONSEN-REHN, SAKARI SUOMINEN and MATS BROMMELS | Does patients' choice of health centre doctor depend on the organization? A comparative study of four municipalities with different forms of service provision in Finland |
| 2010 | 38 | 7 | 724-730 | JERE P. REIJULA and KARI E. REIJULA | The impact of Finnish tobacco legislation on restaurant workers' exposure to tobacco smoke at work |
| 2010 | 38 | 7 | 731-738 | ELI MOLDE HAGEN, KIRSTI HOKKANEN ØDELIEN, STEIN ATLE LIE and HEGE R. ERIKSEN | Adding a physical exercise programme to brief intervention for low back pain patients did not increase return to work |
| 2010 | 38 | 7 | 739-747 | PERTTI R.T. HUOTARI, HEIMO NUPPONEN, LAURI LAAKSO and URHO M. KUJALA | Secular trends in muscular fitness among Finnish adolescents |
| 2010 | 38 | 7 | 748-755 | DAVID PARKER and KRISTI RÜÜTEL | Associations of high-risk behaviour and HIV status with HIV knowledge among persons in Tallinn, Estonia |
| 2010 | 38 | 7 | 756-760 | MARIKA E. JYLHÄ, PERTTI P. KIRKINEN, KAIJA L. PUURA and EIJA I. TOMAS | Fetal sex determination: Obstetricians' attitudes in antenatal screening units in Finland |
| 2010 | 38 | 7 | 761-767 | GUNNAR MALMBERG, LARS-GÖRAN NILSSON and LARS WEINEHALL | Longitudinal data for interdisciplinary ageing research. Design of the Linnaeus Database |
| 2010 | 38 | 7 | 768-776 | JOSEPHA JOSEPH, JOHAN SVARTBERG, INGER NJØLSTAD and HENRIK SCHIRMER | Incidence of and risk factors for type-2 diabetes in a general population: The Tromsø Study |
| 2010 | 38 | 7 | 777-779 | JØRN OLSEN, HENRIK BRØNNUM-HANSEN, MIKA GISSLER, MATTI HAKAMA, ANDERS HJERN, FINN KAMPER-JØRGENSEN, VILHJÁLMUR RAFNSSON, GRETHE S. TELL, IVAN THAULOW and LAU C. THYGESEN | High-throughput epidemiology: Combining existing data from the Nordic countries in health-related collaborative research |
| 2010 | 38 | 7 | 780-783 |  | News from the National Institutes of Public Health |
| 2010 | 38 | 7 | 784 |  | Public Health Calendar — Public Health Events |
| 2010 | 38 | 8 | 785-787 | CLAUS VINTHER NIELSEN and LUCETTE KIRSTEN MEILLIER | We need more focus on social inequality in rehabilitation |
| 2010 | 38 | 8 | 788-793 | LISBETH Ø. RYGG, MARIT BY RISE, BORGHILD LOMUNDAL, HILDE STRØM SOLBERG and ASLAK STEINSBEKK | Reasons for participation in group-based type 2 diabetes self-management education. A qualitative study |
| 2010 | 38 | 8 | 894-802 | JOHANNA LEPPÄLÄ, HANNA LAGSTRÖM, ANNE KALJONEN and KIRSI LAITINEN | Construction and evaluation of a self-contained index for assessment of diet quality |
| 2010 | 38 | 8 | 803-809 | GUNNAR JOHANSSON | Overweight and obesity in Sweden. A five year follow-up, 2004-2008 |
| 2010 | 38 | 8 | 810-816 | ANNE FABER, HANNE GIVER, JESPER STRØYER and HARALD HANNERZ | Are low back pain and low physical capacity risk indicators for dropout among recently qualified eldercare workers? A follow-up study |
| 2010 | 38 | 8 | 817-825 | PENTTI NIEMINEN, TUULA TOLJAMO, ANNA HAMARI and VUOKKO L. KINNULA | Attitudes to new smoking restrictions and second-hand smoke among young Finnish males |
| 2010 | 38 | 8 | 826-837 | MICHELE ARCANGELO MARTIELLO and MARIANO VINCENZO GIACCHI | High temperatures and health outcomes: A review of the literature |
| 2010 | 38 | 8 | 838-844 | FAWAD JAVED, FERNANDA O. BELLO CORREA, MILISHA CHOTAI, ANWAR R. TAPPUNI and KHALID ALMAS | Systemic conditions associated with areca nut usage: A literature review |
| 2010 | 38 | 8 | 845-856 | HANS JØRGEN SØGAARD and PER BECH | The effect of detecting undetected common mental disorders on psychological distress and quality of life in long-term sickness absence: A randomised controlled trial |
| 2010 | 38 | 8 | 857-863 | MICHAEL S. DAHL, JIMMI NIELSEN and RAMIN MOJTABAI | The effects of becoming an entrepreneur on the use of psychotropics among entrepreneurs and their spouses |
| 2010 | 38 | 8 | 854-872 | MAJ BRITT D. NIELSEN, UTE BÜLTMANN, MALENE AMBY, ULLA CHRISTENSEN, FINN DIDERICHSEN and REINER RUGULIES | Return to work among employees with common mental disorders: Study design and baseline findings from a mixed-method follow-up study |
| 2010 | 38 | 8 | 873-879 | PALMA CHILLÓN, FRANCISCO B. ORTEGA, JONATAN R. RUIZ, TOOMAS VEIDEBAUM, LEILA OJA, JAREK MÄESTU and MICHAEL SJÖSTRÖM | Active commuting to school in children and adolescents: An opportunity to increase physical activity and fitness |
| 2010 | 38 | 8 | 880-888 | OLIVIA WAI MAN FUNG and ALICE YUEN LOKE | Disaster preparedness of families with young children in Hong Kong |
| 2010 | 38 | 8 | 889-892 | TAHEREH MORADI, ANNA SIDORCHUK and JOHAN HALLQVIST | Translation of questionnaire increases the response rate in immigrants: Filling the language gap or feeling of inclusion? |
| 2010 | 38 | 8 | 894-895 |  | News from the National Institutes of Public Health |
| 2010 | 38 | 8 | 896 |  | Public Health Calendar — Public Health Events |
| 2011 | 39 | 1 | 1-2 | FINN KAMPER-JØRGENSEN | 4th European Public Health Conference 2011 in Copenhagen: Public Health and Welfare - Welfare Development and Health |
| 2011 | 39 | 1 | 3-9 | MARTIN LINDSTRÖM | Social capital, political trust, and health locus of control: A population-based study |
| 2011 | 39 | 1 | 10-16 | KARIN HELWEG-LARSEN, JAN SØRENSEN, HENRIK BRØNNUM-HANSEN and MARIE KRUSE | Risk factors for violence exposure and attributable healthcare costs: Results from the Danish national health interview surveys |
| 2011 | 39 | 1 | 17-25 | EVELINA LANDSTEDT and KATJA GILLANDER GÅDIN | Deliberate self-harm and associated factors in 17-year-old Swedish students |
| 2011 | 39 | 1 | 26-34 | IEVA REINE, MEHMED NOVO and ANNE HAMMARSTRÖM | Is participation in labour market programmes related to mental health? Results from a 14-year follow-up of the Northern Swedish Cohort |
| 2011 | 39 | 1 | 35-43 | MELITA SAUKA, ILGA S. PRIEDITE, LUDMILA ARTJUHOVA, VIESTURS LARINS, GUNTARS SELGA, ÖRJAN DAHLSTRÖM and TOOMAS TIMPKA | Physical fitness in northern European youth: Reference values from the Latvian Physical Health in Youth Study |
| 2011 | 39 | 1 | 44-50 | KARINA NYGREN, URBAN JANLERT and LENNART NYGREN | Norm compliance and self-reported health among Swedish adolescents |
| 2011 | 39 | 1 | 51-57 | YLVA STÅHL, MATS GRANLUND, BOEL GÄRE-ANDERSSON and KARIN ENSKÄR | Mapping of children's health and development data on population level using the classification system ICF-CY |
| 2011 | 39 | 1 | 58-63 | O. R. HAAVET, Å. SAGATUN and L. LIEN | Adolescents' adverse experiences and mental health in a prospective perspective |
| 2011 | 39 | 1 | 64-69 | BRIAN KØSTER, CAMILLA THORGAARD, ANJA PHILIP and INGE HAUNSTRUP CLEMMENSEN | Vacations to sunny destinations, sunburn, and intention to tan: A cross-sectional study in Denmark, 2007-2009 |
| 2011 | 39 | 1 | 70-78 | WILLIAM B. GRANT, ASTA JUZENIENE and JOHAN E. MOAN | Health benefit of increased serum 25(OH)D levels from oral intake and ultraviolet-B irradiance in the Nordic countries |
| 2011 | 39 | 1 | 79-87 | WILLIAM J. VALENTINE, MARK AAGREN, MATTIAS HAGLUND, ÅSA ERICSSON and MANUELA H. GSCHWEND | Evaluation of the long-term cost-effectiveness of insulin detemir compared with neutral protamine hagedorn insulin in patients with type 1 diabetes using a basal-bolus regimen in Sweden |
| 2011 | 39 | 1 | 88-97 | NELIA P. STEYN, JOHANNA H. NEL, WHADI-AH PARKER, ROSEMARY AYAH and DORCUS MBITHE | Dietary, social, and environmental determinants of obesity in Kenyan women |
| 2011 | 39 | 1 | 98-105 | A. L. ÖSTBERG, I. WIKSTRAND and K. BENGTSSON BOSTRÖM | Group treatment of obesity in primary care practice: A qualitative study of patients' perspectives |
| 2011 | 39 | 1 | 106-111 |  | News on Health Policy and Public Health |
| 2011 | 39 | 1 | 112 |  | Public Health Calendar — Public Health Events |
| 2011 | 39 | 2 | 113-120 | KARI BATT-RAWDEN and GUNNAR TELLNES | How music may promote healthy behaviour |
| 2011 | 39 | 2 | 121-127 | ULLA BEIJER, SVEN ANDREASSON, GUNNAR ÅGREN and ANNA FUGELSTAD | Mortality and causes of death among homeless women and men in Stockholm |
| 2011 | 39 | 2 | 128-136 | ANDERS B. GOTTLIEB HANSEN, ULLA ARTHUR HVIDTFELDT, MORTEN GRØNBÆK, ULRIK BECKER, ANETTE SØGAARD NIELSEN and JANNE SCHURMANN TOLSTRUP | The number of persons with alcohol problems in the Danish population |
| 2011 | 39 | 2 | 137-146 | ANOUK M. SPEETS, JUDITH H. WOLLESWINKEL, ARNE FORSGREN and PATRIK A. SOBOCKI | Use of medical resources and indirect costs of otitis media in Sweden |
| 2011 | 39 | 2 | 147-155 | LOUISE HERBILD, MICKAEL BECH, DORTE GYRD-HANSEN, MONA CHRISTENSEN, THOMAS WERGE and KIRSTEN ANNETTE NIELSEN | Do guidelines recommending pharmacogenetic testing of psychiatric patients affect treatment costs and the use of healthcare services? |
| 2011 | 39 | 2 | 156-163 | FAISAL OMAR, GUSTAV TINGHÖG and STELLAN WELIN | Incentivizing deceased organ donation: A Swedish priority-setting perspective |
| 2011 | 39 | 2 | 164-171 | HELENE FEVEILE, LONE SCHMIDT, HARALD HANNERZ and KARIN SØRIG HOUGAARD | Industrial differences in female fertility treatment rates - A new approach to assess differences related to occupation? |
| 2011 | 39 | 2 | 172-178 | SUSANNE SUNDELL LECEROF, RAGNAR WESTERLING, MAHNAZ MOGHADDASSI and PER-OLOF ÖSTERGREN | Health information for migrants: The role of educational level in prevention of overweight |
| 2011 | 39 | 2 | 179-186 | STEFAN FORS, CARIN LENNARTSSON and OLLE LUNDBERG | Live long and prosper? Childhood living conditions, marital status, social class in adulthood and mortality during mid-life: A cohort study |
| 2011 | 39 | 2 | 187-193 | ANNE HAMMARSTRÖM, PER E. GUSTAFSSON, MATTIAS STRANDH, PEKKA VIRTANEN and URBAN JANLERT | It's no surprise! Men are not hit more than women by the health consequences of unemployment in the Northern Swedish Cohort |
| 2011 | 39 | 2 | 194-202 | KAISA PYRHÖNEN, ESA LÄÄRÄ, MINNA KAILA, LIISA HILTUNEN and SIMO NÄYHÄ | SKARP - A population-based cohort study of childhood food-associated symptoms perceived by parents and food allergies diagnosed by physicians: Design, methods and participation |
| 2011 | 39 | 2 | 203-211 | LOUISE ERIKSEN, MORTEN GRØNBÆK, JØRN WULFF HELGE, JANNE SCHURMANN TOLSTRUP and TINE CURTIS | The Danish Health Examination Survey 2007-2008 (DANHES 2007-2008) |
| 2011 | 39 | 2 | 212-219 | FATUMO A. OSMAN and MAJA SÖDERBÄCK | Perceptions of the use of khat among Somali immigrants living in Swedish society |
| 2011 | 39 | 2 | 220-223 |  | News from the National Institutes of Public Health |
| 2011 | 39 | 2 | 224 |  | Public Health Calendar — Public Health Events |
| 2011 | 39 | 3 | 225-229 | JØRN OLSEN | Register-based research: Some methodological considerations |
| 2011 | 39 | 3 | 230-238 | JANKO JANKOVIC, JELENA MARINKOVIC and SNEZANA SIMIC | Utility of data from a national health survey: Do socioeconomic inequalities in morbidity exist in Serbia? |
| 2011 | 39 | 3 | 239-244 | ESPERANZA DÍAZ and STEFAN HJÖRLEIFSSON | Immigrant general practitioners in Norway: A special resource? A qualitative study |
| 2011 | 39 | 3 | 245-254 | ANNA LÖFGREN, CHARLOTTE SILÉN and KRISTINA ALEXANDERSON | How physicians have learned to handle sickness-certification cases |
| 2011 | 39 | 3 | 255-261 | PIA SOLIN and JUHANI LEHTO | Mental health in complex health promotion policy programmes: The contribution of programme evaluations |
| 2011 | 39 | 3 | 262-271 | HELLE TERKILDSEN MAINDAL, ANNELLI SANDBÆK, MARIT KIRKEVOLD and TORSTEN LAURITZEN | Effect on motivation, perceived competence, and activation after participation in the "Ready to Act" programme for people with screen-detected dysglycaemia: A 1-year randomised controlled trial, Addition-DK |
| 2011 | 39 | 3 | 272-279 | DRAŽENKA VADLA, JADRANKA BOŽIKOV, BENGT ÅKERSTRÖM, WAI-YEE CHEUNG, LUKA KOVAČIĆ, MARIA MASANOVIC, SATU MERILAINEN, SANDRA MIHEL, HANNA NUMMELIN-NIEMI, IOANNA STEFANAKI and BO STENCRANTZ | Differences in healthcare service utilisation in elderly, registered in eight districts of five European countries |
| 2011 | 39 | 3 | 280-286 | HANNE GIVER, ANNE FABER, JESPER STRØYER, HARALD HANNERZ and KAREN ALBERTSEN | Do lifestyle factors and general health predict dropout among recently qualified eldercare workers? A two-year follow-up study |
| 2011 | 39 | 3 | 287-295 | GRAŽINA RIMŠELIENÈ, LINE VOLD, LUCY ROBERTSON, CHRISTIAN NELKE, KJERSTI SØLI, ØYSTEIN HAARKLAU JOHANSEN, FRANK S. THRANA and KARIN NYGÅRD | An outbreak of gastroenteritis among schoolchildren staying in a wildlife reserve: Thorough investigation reveals Norway's largest cryptosporidiosis outbreak |
| 2011 | 39 | 3 | 296-302 | ANNE-METTE LARSEN, RIKKE LUND, MARGIT KRIEGBAUM, KIRSTEN AVLUND and MERETE OSLER | Childhood social circumstances and body mass index in adult life: The Metropolit 1953 Danish male birth cohort |
| 2011 | 39 | 3 | 303-311 | BORGHILD LØYLAND, CHRISTINE MIASKOWSKI, ESPEN DAHL, STEVEN M. PAUL and TONE RUSTØEN | Psychological distress and quality of life in long-term social assistance recipients compared to the Norwegian population |
| 2011 | 39 | 3 | 312-318 | KATRINE HASS RUBIN, BO ABRAHAMSEN, ANNE PERNILLE HERMANN, MICKAEL BECH, JEPPE GRAM and KIM BRIXEN | Fracture risk assessed by Fracture Risk Assessment Tool (FRAX) compared with fracture risk derived from population fracture rates |
| 2011 | 39 | 3 | 319-325 | MONIREH FARSI RAZAVI, LARS FALK, ÅKE BJÖRN and SUSAN WILHELMSSON | Experiences of the Swedish healthcare system: An interview study with refugees in need of long-term health care |
| 2011 | 39 | 3 | 426-332 | JOHN BRODERSEN, VOLKERT SIERSMA and METTE RYLE | Breast cancer screening: "reassuring" the worried well? |
| 2011 | 39 | 3 | 333 | ANNE K. JENUM, LINE SLETNER, NANNA VOLDNER, SIRI VANGEN, KJERSTI MØRKRID, LENE F. ANDERSEN, BRITT NAKSTAD, TORILD SKRIVARHAUG, ODD-HARALD ROGNERUD-JENSEN, BORGHILD ROALD and KARE I. BIRKELAND | CORRIGENDUM |
| 2011 | 39 | 3 | 334-336 |  | News from the National Institutes of Public Health |
| 2011 | 39 | 4 | 337-344 | ANNE-SOFIE HELVIK, KNUT ENGEDAL, STEINAR KROKSTAD and GEIR SELBÆK | A comparison of life satisfaction in elderly medical inpatients and the elderly in a population-based study: Nord-Trøndelag Health Study 3 |
| 2011 | 39 | 4 | 345-353 | NIKLAS EKERSTAD, RURIK LÖFMARK, DAVID ANDERSSON and PER CARLSSON | A tentative consensus-based model for priority setting: An example from elderly patients with myocardial infarction and multi-morbidity |
| 2011 | 39 | 4 | 354-360 | ANNELIE K. GUSDAL, CHRISTEL BECKMAN, ROLF WAHLSTRÖM and LENA TÖRNKVIST | District nurses' use for an assessment tool in their daily work with elderly patients' medication management |
| 2011 | 39 | 4 | 361-370 | LEENA FORMA, MARJA JYLHÄ, MARI AALTONEN, JANI RAITANEN and PEKKA RISSANEN | Municipal variation in health and social service use in the last 2 years of life among old people |
| 2011 | 39 | 4 | 371-388 | MATILDA ANNERSTEDT and PETER WÄHRBORG | Nature-assisted therapy: Systematic review of controlled and observational studies |
| 2011 | 39 | 4 | 389-395 | KAI PART, KAJA RAHU, MATI RAHU and HELLE KARRO | Gender differences in factors associated with sexual intercourse among Estonian adolescents |
| 2011 | 39 | 4 | 396-402 | LOUISE H. SMITH and LOTTE HOLM | Obesity in a life-course perspective: An exploration of lay explanations of weight gain |
| 2011 | 39 | 4 | 403-409 | MAIJA A. ALAHUHTA, EVELIINA E. KORKIAKANGAS, ANNA-MARIA KERÄNEN, HELVI A. KYNGÄS and JAANA H. LAITINEN | Using pictures as vignettes to assess stages of change in weight management |
| 2011 | 39 | 4 | 410-418 | RUI WANG, CHENG WU, XIU-QIANG MA, YAN-FANG ZHAO, XIAO-YAN YAN and JLA HE | Health-related quality of life in Chinese people: A population-based survey of five cities in China |
| 2011 | 39 | 4 | 419-426 | CHARLOTTE VAN TUYCKOM | Macro-environmental factors associated with leisure-time physical activity: A cross-national analysis of EU countries |
| 2011 | 39 | 4 | 427-436 | LINUS SCHIÖLER | Characterisation of influenza outbreaks in Sweden |
| 2011 | 39 | 4 | 437-438 | LONE JØRGENSEN, BENTE MORSETH, MARIT ANDREASSEN and BJARNE K. JACOBSEN | Comments to Emaus et al. "Does a variation in self-reported physical activity reflect variation in objectively measured physical activity, resting heart rate, and physical fitness? Results from the Tromsø study" |
| 2011 | 39 | 4 | 439-441 | JOHAN SUNDSTRÖM, LIISA BYBERG, ROLF GEDEBORG, KARL MICHAËLSSON and LARS BERGLUND | Useful tests of usefulness of new risk factors: Tools for assessing reclassification and discrimination |
| 2011 | 39 | 4 | 442 | JOSEPHA JOSEPH, JOHAN SVARTBERG, INGER NJØLSTAD and HENRIK SCHIRMER | CORRIGENDUM: Incidence of and risk factors for type-2 diabetes in a general population: The Tromsø Study |
| 2011 | 39 | 4 | 443-445 |  | News from the National Institutes of Public Health |
| 2011 | 39 | 4 | 446-447 |  | Acknowledgements |
| 2011 | 39 | 4 | 448 |  | Public Health Calendar — Public Health Events |
| 2011 | 39 | 5 | 449-456 | MARIA MORALES-SUÁREZ-VARELA, LINDA KAERLEV, JIN LIANG ZHU, JENS P. BONDE, ELLEN A. NOHR, AGUSTÍN LLOPIS-GONZÁLEZ, NATALIA GIMENO-CLEMENTE and JØRN OLSEN | Unemployment and pregnancy outcomes: A study within the Danish National Birth Cohort |
| 2011 | 39 | 5 | 457-463 | SARI RÄISÄNEN, KATRI VEHVILÄINEN-JULKUNEN, MIKA GISSLER and SEPPO HEINONEN | High episiotomy rate protects from obstetric anal sphincter ruptures: A birth register-study on delivery intervention policies in Finland |
| 2011 | 39 | 5 | 464-470 | SOEN ENG YAP BJERKE, SIRI VANGEN, ELLEN HOLTER and BABILL STRAY-PEDERSEN | Infectious immune status in an obstetric population of Pakistani immigrants in Norway |
| 2011 | 39 | 5 | 471-478 | ANNA CARLSSON, ANN-CATHRINE BRAMHAGEN, ANNKRISTIN JANSSON and ANNA-KARIN DYKES | Precautions taken by mothers to prevent burn and scald injuries to young children at home: An intervention study |
| 2011 | 39 | 5 | 479-483 | LADAN REZAI, CAMILLA THORGAARD and ANJA PHILIP | Influential factors for sun policy implementation in Danish kindergartens |
| 2011 | 39 | 5 | 484-491 | LINA JARUSEVICIENE, JEFFREY V. LAZARUS and APOLINARAS ZABORSKIS | Confidentiality and parental involvement in adolescent sexual and reproductive health care: A cross-sectional study of Lithuanian general practitioners |
| 2011 | 39 | 5 | 492-500 | NANCY J. REHRER, CLAIRE FREEMAN, TANIA CASSIDY, DEBRA L. WATERS, GARY E. BARCLAY and NOELA WILSON | Through the eyes of young people: Favourite places for physical activity |
| 2011 | 39 | 5 | 501-507 | KLAS GUSTAFSSON, GUNNEL BACKENROTH-OHSAKO, ULF ROSENHALL, ELISABETH TERNEVALL-KJERULF, MATS ULFENDAHL and KRISTINA ALEXANDERSON | Future risk for disability pension among people with sickness absence due to otoaudiological diagnoses: a population-based cohort study with a 12-year follow-up |
| 2011 | 39 | 5 | 508-516 | EMMA NILSING, ELSY SÖDERBERG, HELENA NORMELLI and BIRGITTA ÖBERG | Description of functioning in sickness certificates |
| 2011 | 39 | 5 | 517-524 | EIVIND MELAND, SIRI GRØNHAUG, KRISTIN ØYSTESE and THOMAS MILDESTVEDT | Examining the Matthew effect on the motivation and ability to stay at work after heart disease |
| 2011 | 39 | 5 | 525-532 | ANNUKKA KIMANEN, MARIA RAUTIO, PIRJO MANNINEN, KIMMO RÄSÄNEN, PÄIVI HUSMAN and KAJ HUSMAN | Primary care visits to occupational health physicians and nurses in Finland |
| 2011 | 39 | 5 | 533-539 | ANNA-KARIN WAENERLUND, PEKKA VIRTANEN and ANNE HAMMARSTRÖM | Is temporary employment related to health status? Analysis of the Northern Swedish Cohort |
| 2011 | 39 | 5 | 540-546 | MALGORZATA GAJEWSKA, PAWEL GORYNSKI and MIROSLAW J. WYSOCKI | Hospitalisation of people with obesity in Poland in years 1985-2007 |
| 2011 | 39 | 5 | 547-552 | TUULA VASANKARI, PEKKA JOUSILAHTI, PAUL KNEKT, JUKKA MARNIEMI, SAMI HEISTARO, KARI LIIPPO and MARKKU HELIOVAARA | Serum cotinine predicts bronchial obstruction regardless of self-reported smoking history |
| 2011 | 39 | 5 | 553-554 | SUSANNE WALDAU and LARS LINDHOLM | Some corrective notes regarding recent priority setting in Sweden |
| 2011 | 39 | 5 | 555 | LOUISE HERBILD, MICKAEL BECH, DORTE GYRD-HANSEN, MONA CHRISTENSEN, THOMAS WERGE and KIRSTEN ANNETTE NIELSEN | CORRIGENDUM: Do guidelines recommending pharmacogenetic testing of psychiatric patients affect treatment costs and the use of healthcare services? |
| 2011 | 39 | 5 | 556-558 |  | News from the National Institutes of Public Health |
| 2011 | 39 | 5 | 559 |  | Public Health Calendar — Public Health Events |
| 2011 | 39 | 6 | 561-570 | NINA RAUTIO, JARI JOKELAINEN, HEIKKI OKSA, TIMO SAARISTO, MARKKU PELTONEN, LEO NISKANEN, HANNU PUOLIJOKI, MAUNO VANHALA, MATTI UUSITUPA and SIRKKA KEINÄNEN-KIUKAANNIEMI | Socioeconomic position and effectiveness of lifestyle intervention in prevention of type 2 diabetes: One-year follow-up of the FIN-D2D project |
| 2011 | 39 | 6 | 571-576 | ANN-SOFIE SONNE HOLM, GITTE STENTEBJERG OLSEN, ANDERS BORGLYKKE and TORBEN JØRGENSEN | Estimating the proportion of Danes at high risk of fatal cardiovascular disease |
| 2011 | 39 | 6 | 577-581 | PEKKA JOHANNES PUUSTINEN, HANNU KOPONEN, HANNU KAUTIAINEN, PEKKA MÄNTYSELKÄ and MAUNO VANHALA | Psychological distress measured by the GHQ-12 and mortality: A prospective population-based study |
| 2011 | 39 | 6 | 582-589 | MADS UFFE PEDERSEN, MORTEN HESSE and KIM BLOOMFIELD | Abstinence-orientated residential rehabilitation of opioid users in Denmark: Do changes in national treatment policies affect treatment outcome? |
| 2011 | 39 | 6 | 590-597 | AGNETA KULLBERG, CECILIA NORDQVIST, TOOMAS TIMPKA and KENT LINDQVIST | Residents' perspectives on safety support needs in different types of housing areas |
| 2011 | 39 | 6 | 598-607 | LENE H. HASTRUP, BERNARD VAN DEN BERG and DORTE GYRD-HANSEN | Do informal caregivers in mental illness feel more burdened? A comparative study of mental versus somatic illnesses |
| 2011 | 39 | 6 | 608-617 | M.J.H. VAN BON-MARTENS, L.A.M. VAN DE GOOR, P.W. ACHTERBERG and J.A.M. VAN OERS | The development of an empirical model for regional public health reporting. A descriptive study in two Dutch pilot regions |
| 2011 | 39 | 6 | 618-626 | ANNA BRYNGELSON, JENNIE BACCHUS HERTZMAN and JOHAN FRITZELL | The relationship between gender segregation in the workplace and long-term sickness absence in Sweden |
| 2011 | 39 | 6 | 627-633 | ELISABET WERNTOFT and ANNA-KARIN EDBERG | Lack of support structures in prioritization decision making concerning patients and resources. Interviews with Swedish physicians |
| 2011 | 39 | 6 | 634-639 | ERIK AARDEN, INE VAN HOYWEGHEN and KLASIEN HORSTMAN | The paradox of public health genomics: Definition and diagnosis of familial hypercholesterolaemia in three European countries |
| 2011 | 39 | 6 | 640-648 | ROGER KELLER CELESTE, PAULO NADANOVSKY and JOHAN FRITZELL | Trends in socioeconomic disparities in the utilization of dental care in Brazil and Sweden |
| 2011 | 39 | 6 | 649-655 | M.C.M. GOOSSENS and ANNE MARIE DEPOORTER | Contacts between general practitioners and migrants without a residence permit and the use of "urgent" medical care |
| 2011 | 39 | 6 | 656-664 | PENGQIAN FANG, XUELEI HAN, JIE CHEN, LUZHAO FENG, SUSAN TANG and HONOJlE YU | How many working days would be missed due to moderate or severe influenza pandemic in China? |
| 2011 | 39 | 6 | 665-667 |  | News from the National Institutes of Public Health |
| 2011 | 39 | 6 | 668 |  | Public Health Calendar — Public Health Events |
| 2011 | 39 | 7 | 669-677 | MIRJA HIRVENSALO, RISTO TELAMA, MICHAEL D. SCHMIDT, TUIJA H. TAMMELIN, XIAOLIN YANG, COSTAN G. MAGNUSSEN, JORMA S.A. VIIKARI and OLLI T. RAITAKARI | Daily steps among Finnish adults: Variation by age, sex, and socioeconomic position |
| 2011 | 39 | 7 | 678-686 | INGER K. DAHL-PETERSEN, MARIT E. JØRGENSEN and PETER BJERREGAARD | Physical activity patterns in Greenland: A country in transition |
| 2011 | 39 | 7 | 687-695 | ANNETTE QUINTO ROMANI | Children's weight and participation in organized sports |
| 2011 | 39 | 7 | 696-703 | A. A. OGUNLEYE, C. VOSS, J. L. BARTON, J. N. PRETTY and G. R. H. SANDERCOCK | Contrasting physical activity patterns in children and adolescents living in differing environments in the UK |
| 2011 | 39 | 7 | 704-713 | MICHAL BRONIKOWSKI and MALGORZATA BRONIKOWSKA | Will they stay fit and healthy? A three-year follow-up evaluation of a physical activity and health intervention in Polish youth |
| 2011 | 39 | 7 | 714-722 | MARJAANA PENNANEN, ARI HAUKKALA, HEIN DE VRIES and ERKKI VARTIAINEN | Academic achievement and smoking: Is self-efficacy an important factor in understanding social inequalities in Finnish adolescents? |
| 2011 | 39 | 7 | 723-729 | ANTERO HELOMA, SATU HELAKORPI, JARKKO HONKONEN, PETRI DANIELSSON and ANTTI UUTELA | Exposure to secondhand smoke in Finnish workplaces and compliance with national smoke-free workplace legislation |
| 2011 | 39 | 7 | 730-741 | EVA EURENIUS, MARIE LINDKVIST, MAGDALENA SUNDQVIST, ANNELI IVARSSON and INGRID MOGREN | Maternal and paternal self-rated health and BMI in relation to lifestyle in early pregnancy: The Salut Programme in Sweden |
| 2011 | 39 | 7 | 742-748 | DORTE HØST, CARSTEN HENDRIKSEN and INA BORUP | Older people's perception of and coping with falling, and their motivation for fall-prevention programmes |
| 2011 | 39 | 7 | 749-756 | JANNE LEINO, LAURA KAUHANEN, LASSE HOKKINEN, SUDHIR KURL, ADETUNPJI T. TORIOLA, SARI VOUTILAINEN, JOHN W. LYNCH and JUSSI KAUHANEN | Psychosocial problems in childhood and later alcohol consumption: A life course approach with historical information |
| 2011 | 39 | 7 | 757-765 | ANNA K. FORSMAN, FREDRICA NYQVIST and KRISTIAN WAHLBECK | Cognitive components of social capital and mental health status among older adults: A population-based cross-sectional study |
| 2011 | 39 | 7 | 766-772 | M. RUDBECK and K. FONAGER | Agreement between medical expert assessments in social medicine |
| 2011 | 39 | 7 | 773-775 | SIGNILD VALLGÅRDA | Why the concept "lifestyle diseases" should be avoided |
| 2011 | 39 | 7 | 776-779 | INGEBORG LUND and KARL ERIK LUND | Post-ban self-reports on economic impact of smoke-free bars and restaurants are biased by pre-ban attitudes. A longitudinal study among employees |
| 2011 | 39 | 7 | 780-783 |  | News from the National Institutes of Public Health |
| 2011 | 39 | 7 | 784 |  | Public Health Calendar — Public Health Events |
| 2011 | 39 | 8 | 785-796 | BETINA HØJGAARD, KIM ROSE OLSEN, CHARLOTTA PISINGER, HANNE TØNNESEN and DORTE GYRD-HANSEN | The potential of smoking cessation programmes and a smoking ban in public places: Comparing gain in life expectancy and cost effectiveness |
| 2011 | 39 | 8 | 797-804 | SUSANNA TOIVANEN | Income differences in stroke mortality: A 12-year follow-up study of the Swedish working population |
| 2011 | 39 | 8 | 805-812 | MARGARETA KRISTENSON, JOHANNA LUNDBERG and PETER GARVIN | Socioeconomic differences in outpatient healthcare utilisation are mainly seen for musculoskeletal problems in groups with poor self-rated health |
| 2011 | 39 | 8 | 813-822 | BJØRG AGLEN, MARIANNE HEDLUND and BODIL J. LANDSTAD | Self-help and self-help groups for people with long-lasting health problems or mental health difficulties in a Nordic context: A review |
| 2011 | 39 | 8 | 823-829 | HÉLÈNE SANDMARK | Health, sleep, and professional career in female white-collar workers back to work after long-term sick-listing due to minor mental disorders |
| 2011 | 39 | 8 | 830-838 | ANNINA ROPPONEN, KARRI SILVENTOINEN, PER TYNELIUS and FINN RASMUSSEN | Association between hand grip/body weight ratio and disability pension due to musculoskeletal disorders: A population-based cohort study of 1 million Swedish men |
| 2011 | 39 | 8 | 839-848 | ANNINA ROPPONEN, KARRI SILVENTOINEN, PIA SVEDBERG, KRISTINA ALEXANDERSON, KAROLIINA KOSKENVUO, ANTTI HUUNAN-SEPPÄLÄ, MARKKU KOSKENVUO and JAAKKO KAPRIO | Health-related risk factors for disability pensions due to musculoskeletal diagnoses: A 30-year Finnish twin cohort study |
| 2011 | 39 | 8 | 849-856 | KARIN HELWEG-LARSEN, MARIE LOUISE FREDERIKSEN and HELMER BØVING LARSEN | Violence, a risk factor for poor mental health in adolescence: A Danish nationally representative youth survey |
| 2011 | 39 | 8 | 857-863 | INGELISE ANDERSEN, KARSTEN THIELEN, PER BECH, ELSE NYGAARD and FINN DIDERICHSEN | Increasing prevalence of depression from 2000 to 2006 |
| 2011 | 39 | 8 | 864-869 | ULLA TOFT, PETER ERBS-MAIBING and CHARLOTTE GLÜMER | Identifying fast-food restaurants using a central register as a measure of the food environment |
| 2011 | 39 | 8 | 870-879 | KAIJA APPELQVIST-SCHMIDLECHNER, MARKUS HENRIKSSON, MATTI JOUKAMAA, KAI PARKKOLA, MAILA UPANNE and EIJA STENGÅRD | Psychosocial factors associated with suicidal ideation among young men exempted from compulsory military or civil service |
| 2011 | 39 | 8 | 880-887 | LISE SKRUBBELTRANG SKOV-ETTRUP, MARIE ELIASEN, OLA EKHOLM, MORTEN GRØNBÆK and JANNE SCHURMANN TOLSTRUP | Binge drinking, drinking frequency, and risk of ischaemic heart disease: A population-based cohort study |
| 2011 | 39 | 8 | 888-893 | SILJE MAELAND, LIV H. MAGNUSSEN, HEGE R. ERIKSEN and KIRSTI MALTERUD | Why are general practitioners reluctant to enrol patients into a RCT on sick leave? A qualitative study |
| 2011 | 39 | 8 | 894-895 |  | News from the National Institutes of Public Health |
| 2011 | 39 | 8 | 896 |  | Public Health Calendar — Public Health Events |
| 2012 | 40 | 1 | 1-9 | SIGURVEIG H. SIGURDARDOTTIR, GERDT SUNDSTROM, BO MALMBERG and MARIE ERNSTH BRAVELL | Needs and care of older people living at home in Iceland |
| 2012 | 40 | 1 | 10-17 | ÅNEN RINGARD | Equitable access to elective hospital services: The introduction of patient choice in a decentralised healthcare system |
| 2012 | 40 | 1 | 18-24 | HEIDI GAUTUN, ANNE WERNER and HILDE LURÅS | Care challenges for informal caregivers of chronically ill lung patients: Results from a questionnaire survey |
| 2012 | 40 | 1 | 25-34 | MAJDA LAMKADDEM, PETER M. SPREEUWENBERG, WALTER L. DEVILLÉ, MARLEEN M. FOETS and PETER P. GROENEWEGEN | Importance of quality aspects of GP care among ethnic minorities: Role of cultural attitudes, language and healthcare system of reference |
| 2012 | 40 | 1 | 35-42 | PAULA M.C. MOMMERSTEEG, JOHAN DENOLLET and ELISABETH J. MARTENS | Type D personality, depressive symptoms and work-related health outcomes |
| 2012 | 40 | 1 | 43-50 | ANNA C. SVENSSON, PAUL LICHTENSTEIN, SVEN SANDIN, SARA ÖBERG, PATRICK F. SULLIVAN and CHRISTINA M. HULTMAN | Familial aggregation of schizophrenia: The moderating effect of age at onset, parental immigration, paternal age and season of birth |
| 2012 | 40 | 1 | 51-60 | MARTIN LINDSTRÖM, SADIQ M. ALI and MARIA ROSVALL | Socioeconomic status, labour market connection, and self-rated psychological health: The role of social capital and economic stress |
| 2012 | 40 | 1 | 61-68 | MIKA T. VUORI, LASSE K. KANNAS, JARI VILLBERG, S.A. KRISTIINA OJALA, JORMA A. TYNJÄLÄ and RAILI S. VÄLIMAA | Is physical activity associated with low-risk health behaviours among 15-year-old adolescents in Finland? |
| 2012 | 40 | 1 | 69-75 | GIUSEPPE LA TORRE, LEDA SEMYONOV, ALICE MANNOCCI and ANTONIO BOCCIA | Knowledge, attitude, and behaviour of public health doctors towards pandemic influenza compared to the general population in Italy |
| 2012 | 40 | 1 | 76-84 | CECILIE KYRØ, GURI SKEIE, LARS O. DRAGSTED, JANE CHRISTENSEN, KIM OVERVAD, GÖRAN HALLMANS, INGEGERD JOHANSSON, EILIV LUND, NADIA SLIMANI, NINA F. JOHNSEN, JYTTE HALKJÆR, ANNE TJØNNELAND and ANJA OLSEN | Intake of whole grain in Scandinavia: Intake, sources and compliance with new national recommendations |
| 2012 | 40 | 1 | 85-91 | VERONIKA HALVARSSON, SARA STRÖM and FREDRIK LILJEROS | The prescription of oral contraceptives and its relation to the incidence of chlamydia and abortion in Sweden 1997—2005 |
| 2012 | 40 | 1 | 92-101 | PEIYUAN QIU, YANG YANG, XIAO MA, FANG WU, PING YUAN, QIAOLAN LIU and ERIC CAINE | Respondent-driven sampling to recruit in-country migrant workers in China: A methodological assessment |
| 2012 | 40 | 1 | 102-108 | BJÖRN E. ROSENGREN, HENRIK G. AHLBORG, PER GÄRDSELL, INGEMAR SERNBO, JAN-ÅKE NILSSON, ROBIN M. DALY and MAGNUS K KARLSSON | Forearm bone mineral density and incidence of hip fractures in Swedish urban and rural men 1987-2002 |
| 2012 | 40 | 1 | 109-111 |  | News from the National Institutes of Public Health |
| 2012 | 40 | 1 | 112 |  | Public Health Calendar — Public Health Events |
| 2012 | 40 | 2 | 113 | SAKARI SUOMINEN | Call for a new Chief Editor |
| 2012 | 40 | 2 | 114 | FINN KAMPER-JØRGENSEN | Message from the Chief Editor to all authors on waiting time |
| 2012 | 40 | 2 | 115-125 | SUN SUN, ROBERT IRESTIG, BO BURSTRÖM, ULLA BEIJER and KRISTINA BURSTRÖM | Health-related quality of life (EQ-5D) among homeless persons compared to a general population sample in Stockholm County, 2006 |
| 2012 | 40 | 2 | 126-132 | M.W. WÜRGLER, L.T. SONNE, J. KILSMARK, H. VOSS and J. SØGAARD | Danish heart patients' participation in and experience with rehabilitation |
| 2012 | 40 | 2 | 133-141 | SARA MARIE NILSEN, LINDA ERNSTSEN, STEINAR KROKSTAD and STEINAR WESTIN | Educational inequalities in disability pensioning — the impact of illness and occupational, psychosocial, and behavioural factors: The Nord-Trøndelag Health Study (HUNT) |
| 2012 | 40 | 2 | 142-149 | ANDERS MØLSTER GALAASEN, DAG BRUUSGAARD and BJØRGULF CLAUSSEN | Excluded from social security: Rejections of disability pension applications in Norway 1998-2004 |
| 2012 | 40 | 2 | 150-156 | SANNA KÄÄRIÄ, MIKKO LAAKSONEN, PÄIVI LEINO-ARJAS, PEPPIINA SAASTAMOINEN and EERO LAHELMA | Low back pain and neck pain as predictors of sickness absence among municipal employees |
| 2012 | 40 | 2 | 157-166 | TAINA LEINONEN, PEKKA MARTIKAINEN and EERO LAHELMA | Interrelationships between education, occupational social class, and income as determinants of disability retirement |
| 2012 | 40 | 2 | 167-176 | PETER DALUM, GEORG ALUDAN-MÜLLER, GERDA ENGHOLM and GERJO KOK | A cluster randomised controlled trial of an adolescent smoking cessation intervention: Short and long-term effects |
| 2012 | 40 | 2 | 177-182 | ERKER KARLÉN, TOMAS FARESJÖ and JOHNNY LUDVIGSSON | Could the social environment trigger the induction of diabetes related autoantibodies in young children? |
| 2012 | 40 | 2 | 183-190 | ÅSA SAMUELSSON, INGE HOUKES, PETRA VERDONK and ANNE HAMMARSTRÖM | Types of employment and their associations with work characteristics and health in Swedish women and men |
| 2012 | 40 | 2 | 191-202 | KATARINA SWAHNBERG, JOHANNA DAVIDSSON-SIMMONS, JEFF HEARN and BARBRO WIJMA | Men's experiences of emotional, physical, and sexual abuse and abuse in health care: A cross-sectional study of a Swedish random male population sample |
| 2012 | 40 | 2 | 203-210 | M. LISBY, L.P. NIELSEN, B. BROCK and J. MAINZ | How should medication errors be defined? Development and test of a definition |
| 2012 | 40 | 2 | 211-216 | MARLENE MAKENZIUS, TANJA TYDÉN, ELISABETH DARJ and MARGARETA LARSSON | Risk factors among men who have repeated experience of being the partner of a woman who requests an induced abortion |
| 2012 | 40 | 2 | 217-218 |  | News from the National Institutes of Public Health |
| 2012 | 40 | 2 | 219 |  | Public Health Calendar — Public Health Events |
| 2012 | 40 | 3 | 221-228 | MASOUD VAEZGHASEMI, EVA EURENIUS, MARIE LINDKVIST and ANNELI IVARSSON | Overweight and lifestyle among 13-15 year olds: A cross-sectional study in northern Sweden |
| 2012 | 40 | 3 | 229-238 | NELIA P. STEYN, JOHANNA H. NEL, W. PARKER, ROSEMARY AYAH and DORCUS MBITHE | Urbanisation and the nutrition transition: A comparison of diet and weight status of South African and Kenyan women |
| 2012 | 40 | 3 | 239-244 | MARI BJORKMAN and KIRSTI MALTERUD | Lesbian women coping with challenges of minority stress: A qualitative study |
| 2012 | 40 | 3 | 245-252 | LINE A.B. BØRRESTAD, LARS ØSTERGAARD, LARS B. ANDERSEN and ELLING BERE | Experiences from a randomised, controlled trial on cycling to school: Does cycling increase cardiorespiratory fitness? |
| 2012 | 40 | 3 | 253-259 | MARK MCCARTHY and AGNESE KNABE | Civil society and public health research in the European Union new member states |
| 2012 | 40 | 3 | 260-270 | SIGNE S. NIELSEN, NANA F. HEMPLER, FRANS B. WALDORFF, ALLAN KRASNIK and SVEND KREINER | Is there equity in use of healthcare services among immigrants, their descendants, and ethnic Danes? |
| 2012 | 40 | 3 | 271-277 | CLAUDIA SIKORSKI, CHRISTIANE RIEDEL, MELANIE LUPPA, BEATE SCHULZE, PERLA WERNER, HANS-HELMUT KÖNIG and STEFFI G RIEDEL-HELLER | Perception of overweight and obesity from different angles: A qualitative study |
| 2012 | 40 | 3 | 278-285 | KARIN E. ISAKSSON RO, REIDAR TYSSEN, TORE GUDE and OLAF G. AASLAND | Will sick leave after a counselling intervention prevent later burnout? A 3-year follow-up study of Norwegian doctors |
| 2012 | 40 | 3 | 286-293 | LUCETTE KIRSTEN MEILLIER, KIRSTEN MELGAARD NIELSEN, FINN BREINHOLT LARSEN and MOGENS LYTKEN LARSEN | Socially differentiated cardiac rehabilitation: Can we improve referral, attendance and adherence among patients with first myocardial infarction? |
| 2012 | 40 | 3 | 294-302 | JONAS TOLJANDER, ANNA DOVÄRN, YVONNE ANDERSSON, SOFIE IVARSSON and ROLAND LINDQVIST | Public health burden due to infections by verocytotoxin-producing "Escherichia coli" (VTEC) and "Campylobacter" spp. as estimated by cost of illness and different approaches to model disability-adjusted life years |
| 2012 | 40 | 3 | 303-305 |  | News from the National Institutes of Public Health |
| 2012 | 40 | 3 | 306-307 |  | Acknowledgements |
| 2012 | 40 | 3 | 308 |  | Public Health Calendar — Public Health Events |
| 2012 | 40 | 4 | 309-315 | ØYSTEIN LAPPEGARD and PER HJORTDAHL | Acute admissions to a community hospital: Experiences from Hallingdal sjukestugu |
| 2012 | 40 | 4 | 316-324 | SOLVEJ MÅRTENSSON, KRISTINE HALLING HANSEN, KIM ROSE OLSEN, TORBEN BRUNSE HØJMARK, JØRGEN HOLM PETERSEN and PERNILLE DUE | Does participation in preventive child health care at the general practitioner minimise social differences in the use of specialist care outside the hospital system? |
| 2012 | 40 | 4 | 325-332 | PALLE B. PALLESEN and ELSEBETH LYNGE | Employment effects of the Danish rehabilitation benefit |
| 2012 | 40 | 4 | 333-339 | IRENE GARCÍA-MOYA, FRANCISCO RIVERA, CARMEN MORENO, BENGT LINDSTRÖM and ANTONIA JIMÉNEZ-IGLESIAS | Analysis of the importance of family in the development of sense of coherence during adolescence |
| 2012 | 40 | 4 | 340-347 | S. GERWARD, K. PERSSON, P. MIDLÖV, R. EKESBO, B. GULLBERG and B. HEDBLAD | Trends in out-of-hospital ischaemic heart disease deaths 1992 to 2003 in southern Sweden |
| 2012 | 40 | 4 | 348-354 | KAREN WISTOFT and HOLGER HØJLUND | Operative links: The importance of combining perspectives in municipal strategies aimed at children's and adolescents' health |
| 2012 | 40 | 4 | 355-359 | LENE POVLSEN | Immigrant women's clubs in a health-promotion perspective |
| 2012 | 40 | 4 | 360-367 | ANITTA SIRVIÖ, ELLEN EK, JARI JOKELAINEN, MARKKU KOIRANEN, TIMO JÄRVIKOSKI and ANJA TAANILA | Precariousness and discontinuous work history in association with health |
| 2012 | 40 | 4 | 368-376 | HEIDI CARLERBY, ERLING ENGLUND, EIJA VIITASARA, ANDERS KNUTSSON and KATJA GILLANDER GÅDIN | Risk behaviour, parental background, and wealth: A cluster analysis among Swedish boys and girls in the HBSC study |
| 2012 | 40 | 4 | 377-384 | CARL LYSBECK HANSEN, JESPER BAELUM, LARS SKADHAUGE, GERT THOMSEN, ØYVIND OMLAND, TRINE THILSING, SØREN DAHL, TORBEN SIGSGAARD and DAVID SHERSON | Consequences of asthma on job absenteeism and job retention |
| 2012 | 40 | 4 | 385-390 | EVA ANDERBERG, KATARINA STEEN CARLSSON and KERSTIN BERNTORP | Use of healthcare resources after gestational diabetes mellitus: A longitudinal case-control analysis |
| 2012 | 40 | 4 | 391-397 | ANNE ILLEMANN CHRISTENSEN, OLA EKHOLM, CHARLOTTE GLÜMER, ANNE HELMS ANDREASEN, MICHAEL FALK HVIDBERG, PETER LUND KRISTENSEN, FINN BREINHOLT LARSEN, BRITTA ORTIZ and KNUD JUEL | The Danish National Health Survey 2010. Study design and respondent characteristics |
| 2012 | 40 | 4 | 398-401 | HEGE SOLBERG, GERD E. NYSETHER and ASLAK STEINSBEKK | Patients' experiences with metaphors in a solution-focused approach to improve self-management skills: A qualitative study |
| 2012 | 40 | 4 | 402-403 |  | News from the National Institutes of Public Health |
| 2012 | 40 | 4 | 404 |  | Public Health Calendar — Public Health Events |
| 2012 | 40 | 5 | 405 | INGVAR KARLBERG | A message from the new Editor-in-Chief |
| 2012 | 40 | 5 | 406-411 | GIEDRIUS VANAGAS and ZILVINAS PADAIGA | Healthcare spending in the case of a HPV16/18 population-wide vaccination programme |
| 2012 | 40 | 5 | 412-417 | ALI ASGARY | Assessing households' willingness to pay for an immediate pandemic influenza vaccination programme |
| 2012 | 40 | 5 | 418-422 | HELENA A. FIGUEIRA, ALAN A. FIGUEIRA, SAMÁRIA A. CADER, ANDREA C. GUIMARÃES, RICARDO J. DE OLIVEIRA, JOANA A. FIGUEIRA, OLIVIA A. FIGUEIRA and ESTÉLIO H.M. DANTAS | Effects of a physical activity governmental health programme on the quality of life of elderly people |
| 2012 | 40 | 5 | 423-430 | AAKKO ANTTILA, RAMI KANKAANPÄÄ, MIMMI TOLVANEN, SALLA SARANPÄÄ, ANNE HIIRI and SATU LAHTI | Do schools put children's oral health at risk owing to lack of a health-promoting policy? |
| 2012 | 40 | 5 | 431-438 | FREDRICA NYQVIST, MIKAEL NYGÅRD and GUNBORG JAKOBSSON | Social participation, interpersonal trust, and health: A study of 65- and 75-year-olds in western Finland |
| 2012 | 40 | 5 | 439-448 | INGER MARGRETE DYRHOLM SIEMSEN, MARLENE DYRLØV MAUSEN, LENE FUNCK PEDERSEN, LISA MICHAELSEN, ANETTE VESTERSKOV PEDERSEN, HENNING BOJE ANDERSEN and DORIS ØSTERGAARD | Factors that impact on the safety of patient handovers: An interview study |
| 2012 | 40 | 5 | 449-456 | AFSANEH BJORVATN | Making incentives work: Hospital organisation and performance |
| 2012 | 40 | 5 | 457-465 | LARS HARPELUND, SIGNE SMITH NIELSEN and ALLAN KRASNIK | Self-perceived need for interpreter among immigrants in Denmark |
| 2012 | 40 | 5 | 466-474 | NEVENA SKROZA, ERSILIA TOLINO, LEDA SEMYONOV, ILARIA PROIETTI, NICOLETTA BERNARDINI, FRANCESCA NICOLUCCI, GIORGIO LA VIOLA, GIUSEPPE DEL PRETE, ROSELLA SAULLE, CONCETTA POTENZA and GIUSEPPE LA TORRE | Mediterranean diet and familial dysmetabolism as factors influencing the development of acne |
| 2012 | 40 | 5 | 475-481 | CHARLOTTE JEPPESEN and PETER BJERREGAARD | Consumption of traditional food and adherence to nutrition recommendations in Greenland |
| 2012 | 40 | 5 | 482-490 | EMILIE DUPRET, CHRISTINE BOCÉRÉAN, MARDJANE TEHERANI, MARTINE FELTRIN and JAN HYLD PEJTERSEN | Psychosocial risk assessment: French validation of the Copenhagen Psychosocial Questionnaire (COPSOQ) |
| 2012 | 40 | 5 | 491-497 | MEKDES K. GEBREMARIAM, LENE F. ANDERSEN, MONA BJELLAND, KNUT-INGE KLEPP, TORUNN H. TOTLAND, INGUNN H. BERGH and NANNA LIEN | Does the school food environment influence the dietary behaviours of Norwegian 11-year-olds? The HEIA study |
| 2012 | 40 | 5 | 498-500 | IVY SHIUE | Neighbourhood satisfaction and happiness but not urbanization level affect self-rated health in adolescents |
| 2012 | 40 | 5 | 501 |  | News from the National Institutes of Public Health |
| 2012 | 40 | 5 | 502 |  | Public Health Calendar — Public Health Events |
| 2012 | 40 | 6 | 505-515 | REIJO SUND | Quality of the Finnish Hospital Discharge Register: A systematic review |
| 2012 | 40 | 6 | 516-522 | LAILA DAERGA, PER SJÖLANDER, LARS JACOBSSON and ANETTE EDIN-LILJEGREN | The confidence in health care and social services in northern Sweden - a comparison between reindeer-herding Sami and the non-Sami majority population |
| 2012 | 40 | 6 | 523-530 | LIV JOHANNE WEKRE, KJERSTI BAKKEN, HELGE GARÅSEN and ANDERS GRIMSMO | GPs' prescription routines and cooperation with other healthcare personnel before and after implementation of multidose drug dispensing |
| 2012 | 40 | 6 | 531-536 | TORGEIR GILJE LID, KRISTIAN OPPEDAL, BOLETTE PEDERSEN and KIRSTI MALTERUD | Alcohol-related hospital admissions: Missed opportunities for follow up? A focus group study about general practitioners' experiences |
| 2012 | 40 | 6 | 537-543 | TORE TJORA, JØRN HETLAND, LEIF EDVARD AARØ, BENTE WOLD and SIMON ØVERLAND | Late-onset smokers: How many, and associations with health behaviours and socioeconomic status |
| 2012 | 40 | 6 | 544-552 | DAVID T. LEVY, KENNETH BLACKMAN, LAURA M. CURRIE, JEFFREY LEVY and LUKE CLANCY | SimSmokeFinn: How far can tobacco control policies move Finland toward tobacco-free 2040 goals? |
| 2012 | 40 | 6 | 553-562 | LINAS ŠUMSKAS, APOLINARAS ZABORSKIS, KATRIN AASVEE, INESE GOBINA and IVETA PUDULE | Health-behaviour inequalities among Russian and ethnic majority school-aged children in the Baltic countries |
| 2012 | 40 | 6 | 563-570 | ANU LIETTU, HANNU SÄÄVÄLÄ, HELINÄ HAKKO, MATTI JOUKAMAA and PIRKKO RÄSÄNEN | Weapons used in serious violence against a parent: Retrospective comparative register study |
| 2012 | 40 | 6 | 571-578 | OLIVER HIRSCH, SUSANNE TRÄGER, STEFAN BÖSNER, MUAZZEZ ILHAN, ANNETTE BECKER, ERIKA BAUM and NORBERT DONNER-BANZHOFF | Referral from primary to secondary care in Germany: Developing a taxonomy based on cluster analysis |
| 2012 | 40 | 6 | 579-581 | JANE BRANDT SØRENSEN, ALESSANDRO DEMAIO, LISE ROSENDAL ØSTERGAARD, KAROLINE KRAGELUND NIELSEN, I.B. CHRISTIAN BYGBJERG and MAXIMILIAN DE COURTEN | Non-communicable diseases: Harnessing the current opportunities |
| 2012 | 40 | 6 | 582 |  | News from the National Institutes of Public Health |
| 2012 | 40 | 6 | 583 |  | Public Health Calendar — Public Health Events |
| 2012 | 40 | 7 | 585-590 | NYNNE JOHANNE SAHL FREDERIKSEN, SUNNIVA LEONORE BAKKE and PETER DALUM | "No alcohol, no party": An explorative study of young Danish moderate drinkers |
| 2012 | 40 | 7 | 591-595 | THOR NORSTRÖM, ERICA SUNDIN, DANIEL MÜLLER and HÅKAN LEIFMAN | Hazardous drinking among restaurant workers |
| 2012 | 40 | 7 | 596-604 | MARIANNE SKREDEN, HANS SKARI, ULRIK F. MALT, ARE H. PRIPP, MERETHE D. BJÖRK, ANNE FAUGLI and RAGNHILD EMBLEM | Parenting stress and emotional wellbeing in mothers and fathers of preschool children |
| 2012 | 40 | 7 | 605-613 | ASLAUG PALSDOTTIR, BRYNDIS BJORK ASGEIRSDOTTIR and INGA DORA SIGFUSDOTTIR | Gender difference in wellbeing during school lessons among 10-12-year-old children: The importance of school subjects and student-teacher relationships |
| 2012 | 40 | 7 | 614-620 | BIRGIT NICLASEN, MAX PETZOLD and CHRISTINA W. SCHNOHR | The association between high recreational physical activity and physical activity as a part of daily living in adolescents and availability of local indoor sports facilities and sports clubs |
| 2012 | 40 | 7 | 621-628 | AILA J. AHOLA, VERA MIKKILÄ, MARKKU SARAHEIMO, JOHAN WADÉN, SARI MÄKIMATTILA, CAROL FORSBLOM, RIITTA FREESE and PER-HENRIK GROOP | Sense of coherence, food selection and leisure time physical activity in type 1 diabetes |
| 2012 | 40 | 7 | 629-633 | KRISTI RÜÜTEL, VALENTINA USTINA and R. DAVID PARKER | Piloting HIV rapid testing in community-based settings in Estonia |
| 2012 | 40 | 7 | 634-640 | XINJUN LI, KRISTINA SUNDQUIST and JAN SUNDQUIST | Risks of small-for-gestational-age births in immigrants: A nationwide epidemiological study in Sweden |
| 2012 | 40 | 7 | 641-647 | JOAKIM D. DALEN, TIM HUIJTS, STEINAR KROKSTAD and TERJE A. EIKEMO | Are there educational differences in the association between self-rated health and mortality in Norway? The HUNT Study |
| 2012 | 40 | 7 | 548-655 | EIRIK VIKUM, STEINAR KROKSTAD, DORTHE HOLST and STEINAR WESTIN | Socioeconomic inequalities in dental services utilisation in a Norwegian county: The third Nord-Trøndelag Health Survey |
| 2012 | 40 | 7 | 656-662 | BERIT BRINGEDAL and PER ARNE TUFTE | Social and diagnostic inequality in health |
| 2012 | 40 | 7 | 663-667 | SVEINBJÖRN GIZURARSON, LINDA RÓS BJÖRNSDÓTTIR, RANNVEIG EINARSDÓTTIR, MATTHÍAS HALLDÓRSSON and KARL ANDERSEN | Clinical consequences following regulatory changes in respect to reimbursement of statins cost by the Icelandic Social Insurance Administration |
| 2012 | 40 | 7 | 668-673 | MORTEN BLEKESAUNE | Job insecurity and sickness absence: Correlations between attrition and absence in 36 occupational groups |
| 2012 | 40 | 7 | 674-680 | MAARIT K. VALTONEN, DAVID E. LAAKSONEN, JARI A. LAUKKANEN, TOMMI TOLMUNEN, HEIMO VIINAMÄKI, HANNA-MAARIA LAKKA, TIMO A. LAKKA, LEO NISKANEN and JUSSI KAUHANEN | Low-grade inflammation and depressive symptoms as predictors of abdominal obesity |
| 2012 | 40 | 7 | 681-688 | RUNE JOHANSEN, MARIT ROGNERUD, JON MARTIN SUNDET and LEIF EDVARD AARØ | Observed trends in mental health: A strategy to adjust for nonresponse bias and demographic changes in survey data |
| 2012 | 40 | 7 | 689-690 |  | News from the National Institutes of Public Health |
| 2012 | 40 | 7 | 691 |  | Public Health Calendar — Public Health Events |
| 2012 | 40 | 8 | 693-703 | CAMILLA T. DAMSGAARD, STINE-MATHILDE DALSKOV, RIKKE A. PETERSEN, LOUISE B. SØRENSEN, CHRISTIAN MØLGAARD, ANJA BILTOFT-JENSEN, RIKKE ANDERSEN, ANNE V. THORSEN, INGE TETENS, ANDERS SJÖDIN, MADS F. HJORTH, DITTE VASSARD, JØRGEN D. JENSEN, NIELS EGELUND, CAMILLA B. DYSSEGAARD, IB SKOVGAARD, ARNE ASTRUP and KIM F. MICHAELSEN | Design of the OPUS School Meal Study: A randomised controlled trial assessing the impact of serving school meals based on the New Nordic Diet |
| 2012 | 40 | 8 | 704-711 | INGEBORG HARTZ, KARI FURU, TROND BRATLID, MARTE HANDAL and SVETLANA SKURTVEIT | Hypnotic drug use among 0-17 year olds during 2004-2011: A nationwide prescription database study |
| 2012 | 40 | 8 | 712-717 | TRACEY GALLOWAY, BIRGIT V.L. NICLASEN, GINA MUCKLE, KUE YOUNG and GRACE M. EGELAND | Growth measures among preschool-age Inuit children living in Canada and Greenland |
| 2012 | 40 | 8 | 718-724 | INGELA ENMARKER, OVE HELLZÉN, KNUT EKKER and ANN-GRETHE BERG | Health in older cat and dog owners: The Nord-Trondelag Health Study (HUNT)-3 study |
| 2012 | 40 | 8 | 725-729 | ANDERS HAKANSSON, PERNILLA ISENDAHL, CAMILLA WALLIN and MATS BERGLUND | Respondent-driven sampling in a syringe exchange setting |
| 2012 | 40 | 8 | 730-737 | CLAES-GÖRAN ÖSTENSON, AGNETA HILDING, VALDEMAR GRILL and SUAD EFENDIC | High consumption of smokeless tobacco ("snus") predicts increased risk of type 2 diabetes in a 10-year prospective study of middle-aged Swedish men |
| 2012 | 40 | 8 | 738-745 | RIGMOR C. BERG | High rates of unprotected sex and serosorting among men who have sex with men: A national online study in Norway |
| 2012 | 40 | 8 | 746-752 | ANNE HAMMARSTRÖM and SUSAN P. PHILLIPS | Gender inequity needs to be regarded as a social determinant of depressive symptoms: Results from the Northern Swedish cohort |
| 2012 | 40 | 8 | 753-760 | Experience of violation during the past 3 months, social capital, and self-rated health: A population-based study | Experience of violation during the past 3 months, social capital, and self-rated health: A population-based study |
| 2012 | 40 | 8 | 761-764 | VICTOR GRECH | Sex ratios at birth in Scandinavia over the past sixty years |
| 2012 | 40 | 8 | 765-772 | TOAN K. TRAN, BO ERIKSSON, CHUC TK NGUYEN, PETER HORBY, GORAN BONDJERS and MAX PETZOLD | DodaLab: An urban health and demographic surveillance site, the first three years in Hanoi, Vietnam |
| 2012 | 40 | 8 | 773-776 | MALIN INGHAMMAR, GUNNAR ENGSTRÖM, CLAES-GÖRAN LÖFDAHL and ARNE EGESTEN | Validation of a COPD diagnosis from the Swedish Inpatient Registry |
| 2012 | 40 | 8 | 777-783 | NEDIM SELIMOVIC, BENGT RUNDQVIST, EWA KJÖRK, JOHAN VIRIDEN, JAMES TWISS and STEPHEN P. MCKENNA | Adaptation and validation of the Cambridge pulmonary hypertension outcome review for Sweden |
| 2012 | 40 | 8 | 784-794 | GUSTAV TINGHÖG and PER CARLSSON | Individual responsibility for healthcare financing: Application of an analytical framework exploring the suitability of private financing of assistive devices |
| 2012 | 40 | 8 | 795-805 | KIRSTI MALTERUD | Systematic text condensation: A strategy for qualitative analysis |
| 2012 | 40 | 8 | 806-807 |  | News from the National Institutes of Public Health |
| 2012 | 40 | 8 | 808 |  | Public Health Calendar — Public Health Events |
| 2013 | 41 | 1 | 1-3 | CRAIG M. BECKER and PATRICIA RHYNDERS | It's time to make the profession of health about health |
| 2013 | 41 | 1 | 4-10 | SUSANNE REINDAHL RASMUSSEN | The cost effectiveness of telephone counselling to aid smoking cessation in Denmark: A modelling study |
| 2013 | 41 | 1 | 11-17 | TARJA HEPONIEMI, ANNE KOUVONEN, TIMO SINERVO and MARKO ELOVAINIO | Is the public healthcare sector a more strenuous working environment than the private sector for a physician? |
| 2013 | 41 | 1 | 18-24 | KIRSTEN JØRGENSEN and BJARNE LAURSEN | Absence from work due to occupational and non-occupational accidents |
| 2013 | 41 | 1 | 25-31 | KATRI KORPELA, EIRA ROOS, TEA LALLUKKA, OSSI RAHKONEN, EERO LAHELMA and MIKKO LAAKSONEN | Different measures of body weight as predictors of sickness absence |
| 2013 | 41 | 1 | 32-42 | LENA KARLQVIST and GUNVOR GARD | Health-promoting educational interventions: A one-year follow-up study |
| 2013 | 41 | 1 | 43-50 | RIKKE SØGAARD, ES LINDHOLT and DORTE GYRD-HANSE | Individual decision making in relation to participation in cardiovascular screening: A study of revealed and stated preferences |
| 2013 | 41 | 1 | 51-57 | STEFANIE SCHÜTTE, JEAN-FRANÇOIS CHASTANG, AGNÈS PARENT-THIRION, GREET VERMEYLEN and ISABELLE NIEDHAMMER | Social differences in self-reported health among men and women in 31 countries in Europe |
| 2013 | 41 | 1 | 58-64 | GIEDRIUS VANAGAS, AUŠRA MICKEVIČIENÉ and ALBERTAS ULYS | Does quality of life of prostate cancer patients differ by stage and treatment? |
| 2013 | 41 | 1 | 65-80 | ANNE ELISE EGGEN, ELLISIV B. MATHIESEN, TOM WILSGAARD, BJARNE K. JACOBSEN and INGER NJØLSTAD | The sixth survey of the Tromso Study (Tromse 6) in 2007-08: Collaborative research in the interface between clinical medicine and epidemiology: Study objectives, design, data collection procedures, and attendance in a multipurpose population-based health survey |
| 2013 | 41 | 1 | 81-91 | YLVA B. ALMQUIST | School performance as a precursor of adult health: Exploring associations to disease-specific hospital care and their possible explanations |
| 2013 | 41 | 1 | 92-101 | TRINE N WINDING, ELLEN A NOHR, MERETE LABRIOLA, KARIN BIERING and JOHAN H ANDERSEN | Personal predictors of educational attainment after compulsory school: Influence of measures of vulnerability, health, and school performance |
| 2013 | 41 | 1 | 102-108 | GRO IDLAND, KNUT ENGEDAL and ASTRID BERGLAND | Physical performance and 13.5-year mortality in elderly women |
| 2013 | 41 | 1 | 109-110 |  | News from the National Institutes of Public Health |
| 2013 | 41 | 1 | 111 |  | Public Health Calendar — Public Health Events |
| 2013 | 41 | 2 | 113-118 | JØRGEN NEXØE, JULIE PRÆST WILCHE, BIRGIT NICLASEN, ANN BIRKEKÆR KJELDSEN, CHRISTIAN FÆRGEMANN, ANDERS MUNCK and JENS MARTIN LAURITSEN | Violence- and alcohol-related acute healthcare visits in Greenland |
| 2013 | 41 | 2 | 119-125 | LINN GJERSING, KRISTINE V. JONASSEN, STIAN BIONG, EDLE RAVNDAL, HELGE WAAL, JØRGEN G. BRAMNESS and THOMAS CLAUSEN | Diversity in causes and characteristics of drug-induced deaths in an urban setting |
| 2013 | 41 | 2 | 126-133 | CAROLYN DRESLER, MEI WEI, JULIA E. HECK, SHANE ALLWRIGHT, MARGARETHA HAGLUND, SARA SANCHEZ, EVA KRALIKOVA, ISABELLE STÜCKER, ELIZABETH TAMANG, ELLEN R. GRITZ and MIA HASHIBE | Attitudes of women from five European countries regarding tobacco control policies |
| 2013 | 41 | 2 | 134-141 | STEFAN FORS, NEDA AGAHI and BENJAMIN A SHAW | Paying the price? The impact of smoking and obesity on health inequalities in later life |
| 2013 | 41 | 2 | 142-149 | C. CATALINA-ROMERO, E. CALVO, M. A. SÁNCHEZ-CHAPARRO, P. VALDIVIELSO, J. C. SAINZ, M. CABRERA, A. GONZÁLEZ-QUINTELA and J. ROMÁN | The relationship between job stress and dyslipidemia |
| 2013 | 41 | 2 | 150-157 | OTTO MELCHIOR POULSEN, ROGER PERSSON, JESPER KRISTIANSEN, LARS LOUIS ANDERSEN, EBBE VILLADSEN and PALLE ØRBÆK | Distribution of subjective health complaints, and their association with register based sickness absence in the Danish working population |
| 2013 | 41 | 2 | 158-165 | LEIF JOSTEIN REIME and BJØRGULF CLAUSSEN | Municipal unemployment and municipal typologies as predictors of disability pensioning in Norway: A multilevel analysis |
| 2013 | 41 | 2 | 166-173 | IDUNN BREKKE, JOHN E. BERG, LINE SLETNER and ANNE KAREN JENUM | Doctor-certified sickness absence in first and second trimesters of pregnancy among native and immigrant women in Norway |
| 2013 | 41 | 2 | 174-179 | JAN SCHYLLANDER, STAFFAN JANSON, CECILIA NYBERG, ULLA-BRITT ERIKSSON and DIANA STARK EKMAN | Case analyses of all children's drowning deaths occurring in Sweden 1998-2007 |
| 2013 | 41 | 2 | 180-184 | ELLING BERE and JOHNE H. WESTERSJØ | Nature trips and traditional methods for food procurement in relation to weight status |
| 2013 | 41 | 2 | 185-190 | CAMILLA BUCH GUDDE, TURID MØLLER OLSØ, DAG ØIVIND ANTONSEN, MARIT RØ, LASSE ERIKSEN and SOLFRID VATNE | Experiences and preferences of users with major mental disorders regarding helpful care in situations of mental crisis |
| 2013 | 41 | 2 | 191-197 | MAJ BRITT D. NIELSEN, IDA E.H. MADSEN, UTE BÜLTMANN, ULLA CHRISTENSEN, FINN DIDERICHSEN and REINER RUGULIES | Encounters between workers sick-listed with common mental disorders and return-to-work stakeholders. Does workers' gender matter? |
| 2013 | 41 | 2 | 198-205 | ANNA ELISABET OLAFSDOTTIR, PASCALE ALLOTEY and DANIEL D. REIDPATH | A health system in economic crises: A case study from Iceland |
| 2013 | 41 | 2 | 206-215 | FAISAL OMAR, GUSTAV TINGHÖG, PER CARLSSON, MARIE OMNELL-PERSSON and STELLAN WELIN | Priority setting in kidney transplantation: A qualitative study evaluating Swedish practices |
| 2013 | 41 | 2 | 216 |  | News from the National Institutes of Public Health |
| 2013 | 41 | 2 | 217 |  | Public Health Calendar — Public Health Events |
| 2013 | 41 | 3 | 221-229 | METTE GRØNKJÆR, TINE CURTIS, CHARLOTTE DE CRESPIGNY and CHARLOTTE DELMAR | Drinking contexts and the legitimacy of alcohol use: Findings from a focus group study on alcohol use in Denmark |
| 2013 | 41 | 3 | 230-232 | GRACE WYSHAK | Health disparities paradoxes: Depression and chronic back pain in college alumnae and U.S. women, 39-49 |
| 2013 | 41 | 3 | 233-239 | BIRGITTA KERSTIS, GABRIELLA ENGSTRÖM, BIRGITTA EDLUND and CLARA AARTS | Association between mothers' and fathers' depressive symptoms, sense of coherence and perception of their child's temperament in early parenthood in Sweden |
| 2013 | 41 | 3 | 240-246 | RIKKE LINDGAARD HEDELAND, MARIANNE H. JØRGENSEN, GRETE TEILMANN, LINE R. THIESEN, METTE VALENTINER, AMNE ISKANDAR, BRITT MORTHORST and JESPER ANDERSEN | Childhood suicide attempts with acetaminophen in Denmark: Characteristics, social behaviour, trends and risk factors |
| 2013 | 41 | 3 | 247-255 | PATRICIA OLAYA-CONTRERAS and JORMA STYF | Biopsychosocial function analyses changes the assessment of the ability to work in patients on long-term sick-leave due to chronic musculoskeletal pain: The role of undiagnosed mental health comorbidity |
| 2013 | 41 | 3 | 256-259 | R HOEDEMAN, AH BLANKENSTEIN, PC KOOPMANS and JW GROOTHOFF | What bothers the sick-listed employee with severe MUPS? |
| 2013 | 41 | 3 | 260-268 | ALEXANDER MIETHING, OLLE LUNDBERG and SIEGFRIED GEYER | Income and health in different welfare contexts: A comparison of Sweden, East and West Germany |
| 2013 | 41 | 3 | 269-283 | SUSANNA GEIDNE, MIKAEL QUENNERSTEDT, CHARLI ERIKSSON and CC-G Eriksson | The youth sports club as a health-promoting setting: An integrative review of research |
| 2013 | 41 | 3 | 284-292 | ANNICA BRÄNNLUND, ANNE HAMMARSTRÖM and MATTIAS STRANDH | Education and health-behaviour among men and women in Sweden: A 27-year prospective cohort study |
| 2013 | 41 | 3 | 293-301 | STEVEN NORDIN, EVA PALMQUIST and ANNA-SARA CLAESON | Metric properties and normative data for brief noise and electromagnetic field sensitivity scales |
| 2013 | 41 | 3 | 302-310 | CHRISTINE L. BEHRENS, CAMILLA THORGAARD, ANJA PHILIP and JOAN BENTZEN | Sunburn in children and adolescents: associations with parents' behaviour and attitudes |
| 2013 | 41 | 3 | 311-317 | ARUN K SIGURDARDOTTIR, SOLVEIG A ARNADOTTIR and ELIN DIANNA GUNNARSDOTTIR | Socioeconomic status and differences in medication use among older people according to ATC categories and urban-rural residency |
| 2013 | 41 | 3 | 318-325 | JANNE AGERHOLM, DANIEL BRUCE, ANTONIO PONCE DE LEON and BO BURSTRÖM | Socioeconomic differences in healthcare utilization, with and without adjustment for need: An example from Stockholm, Sweden |
| 2013 | 41 | 3 | 326 | ASLAUG PALSDOTTIR, BRYNDIS BJORK ASGEIRSDOTTIR and INGA DORA | CORRIGENDUM: Gender difference in wellbeing during school lessons among 10-12-year-old children: The importance of school subjects and student-teacher relationships |
| 2013 | 41 | 3 | 327-328 |  | Acknowledgements |
| 2013 | 41 | 3 | 329-331 |  | News from the National Institutes of Public Health |
| 2013 | 41 | 3 | 332 |  | Public Health Calendar - Public Health Events |
| 2013 | 41 | 4 | 333-335 | JELENA BOSKOVIC, MARCEL LEPPÉE, JOSIP CULIG and MIRELA ERIC | Patient self-reported adherence for the most common chronic medication therapy |
| 2013 | 41 | 4 | 336-339 | JOHAN MOAN, ZIVILE BATURAITE, MANTAS GRIGALAVICIUS and ASTA JUZENIENE | Cutaneous malignant melanoma incidence rates in Norway |
| 2013 | 41 | 4 | 340-343 | NANA FOLMANN HEMPLER, OLA EKHOLM and INGRID WILLAING | Differences in social relations between persons with type 2 diabetes and the general population |
| 2013 | 41 | 4 | 344-350 | SHAMIMA YASMIN, ANINDYA MUKHERJEE, NIRMALYA MANNA, BAIJAYANTI BAUR, MOUSUMI DATTA, MANABENDRA SAU, MANIDIPA ROY and SAMIR DASGUPTA | Gender preference and awareness regarding sex determination among antenatal mothers attending a medical college of eastern India |
| 2013 | 41 | 4 | 351-358 | PASI LAMPELA, PIIA LAVIKAINEN, RISTO HUUPPONEN, ESKO LESKINEN and SIRPA HARTIKAINEN | Comprehensive geriatric assessment decreases prevalence of orthostatic hypotension in older persons |
| 2013 | 41 | 4 | 359-365 | BIRGITTE GRAVERHOLT, TROND RIISE, GRO JAMTVEDT, BETTINA S. HUSEBO and MONICA W. NORTVEDT | Acute hospital admissions from nursing homes: predictors of unwarranted variation? |
| 2013 | 41 | 4 | 366-373 | CORINNA ELISABETH VOSSIUS, ARNT EGIL YDSTEBØ, INGELIN TESTAD and HILDE LURÅS | Referrals from nursing home to hospital: Reasons, appropriateness and costs |
| 2013 | 41 | 4 | 374-383 | DAGMAR LAGERBERG and MARGARETHA MAGNUSSON | Utilization of child health services, stress, social support and child characteristics in primiparous and multiparous mothers of 18-month-old children |
| 2013 | 41 | 4 | 384-391 | SIGRIDUR Þ. EIDSDÓTTIR, ÁLFGEIR L. KRISTJÁNSSON, INGA D. SIGFÚSDÓTTIR, CAROL E. GARBER and JOHN P. ALLEGRANTE | Secular trends in overweight and obesity among Icelandic adolescents: Do parental education levels and family structure play a part? |
| 2013 | 41 | 4 | 392-397 | MARCO MANIERI, HAMPUS SVENSSON and MARTIN STAFSTRÖM | Sex tourist risk behaviour - An on-site survey among Swedish men buying sex in Thailand |
| 2013 | 41 | 4 | 398-404 | RIGMOR C. BERG  Scandinavian Journal of Public Health | Predictors of never testing for HIV among a national online sample of men who have sex with men in Norway |
| 2013 | 41 | 4 | 405-411 | HANNAMARIA KUUSIO, TARJA HEPONIEMI, JUKKA VÄNSKÄ, ANNA-MARI AALTO, JUHO RUSKOAHO and MARKO ELOVAINIO | Psychosocial stress factors and intention to leave job: differences between foreign-born and Finnish-born general practitioners |
| 2013 | 41 | 4 | 412-420 | ANNA KIESSLING, BRITT ARRELÖV, ANNY LARSSON and PETER HENRIKSSON | Quality of medical certificates issued in long-term sick leave or disability in relation to patient characteristics and delivery of health care |
| 2013 | 41 | 4 | 421-428 | ANNE-SOFIE HELVIK, KNUT ENGEDAL and GEIR SELBÆK | Perceived health among the medically hospitalized elderly: A one-year follow-up study |
| 2013 | 41 | 4 | 429-435 | RANDI NOSSUM, MARIT B. RISE and ASLAK STEINSBEKK | Patient education – Which parts of the content predict impact on coping skills? |
| 2013 | 41 | 4 | 436 |  | News from the National Institutes of Public Health |
| 2013 | 41 | 4 | 437 |  | Public Health Calendar — Public Health Events |
| 2013 | 41 | 5 | 441 | Ingvar Karlberg | A message from your Editor |
| 2013 | 41 | 5 | 442-454 | MAGNUS K. KARLSSON, THORD VONSCHEWELOV, CAROLINE KARLSSON, MARIA CÖSTER and BJÖRN E. ROSENGEN | Prevention of falls in the elderly: A review |
| 2013 | 41 | 5 | 455-462 | KRISTINE PAPE, JOHAN HÅKON BJØRNGAARD, KARIN A.A. DE RIDDER, STEINAR WESTIN, TURID LINGAAS HOLMEN and STEINAR KROKSTAD | Medical benefits in young Norwegians and their parents, and the contribution of family health and socioeconomic status. The HUNT Study, Norway |
| 2013 | 41 | 5 | 463-469 | FAISAL AL-EMRANI, MARTIN STAFSTRÖM and PER-OLOF ÖSTERGREN | The influences of childhood and adult socioeconomic position on body mass index: A longitudinal Swedish cohort study |
| 2013 | 41 | 5 | 470-478 | ANU POLVINEN, RAIJA GOULD, EERO LAHELMA and PEKKA MARTIKAINEN | Socioeconomic differences in disability retirement in Finland: The contribution of ill-health, health behaviours and working conditions |
| 2013 | 41 | 5 | 479-485 | PÄIVI VIITANEN, HEIKKI VARTIAINEN, JORMA AARNIO, VIRPI VON GRUENEWALDT, SIRPA HAKAMÄKI, TOMI LINTONEN, AINO K MATTILA, TERHI WUOLIJOKI and MATTI JOUKAMAA | Finnish female prisoners - heavy consumers of health services |
| 2013 | 41 | 5 | 486-491 | DAVID PHILIP MCARTHUR, TROND TJERBO and TERJE P. HAGEN | The role of young users in determining long-term care expenditure in Norway |
| 2013 | 41 | 5 | 492-499 | LASSE SKOVGAARD, PETER HALKIER NICOLAJSEN, ELENA PEDERSEN, MATTHIAS KANT, STEN FREDRIKSON, MARJA VERHOEF and DAN MEYROWITSCH | Differences between users and non-users of complementary and alternative medicine among people with multiple sclerosis in Denmark: A comparison of descriptive characteristics |
| 2013 | 41 | 5 | 500-507 | GUNNHILD ÅBERGE VIE, STEINAR KROKSTAD, ROAR JOHNSEN and JOHAN HÅKON BJØRNGAARD | The Health Hazards of Marriage. A cohort study of work related disability within 12,500 Norwegian couples - the HUNT Study |
| 2013 | 41 | 5 | 508-515 | JAKOB AXELSSON, BIRGIT MODÉN, MARIA ROSVALL and MARTIN LINDSTRÖM | Sexual orientation and self-rated health: the role of social capital, offence, threat of violence, and violence |
| 2013 | 41 | 5 | 516-523 | TEA LALLUKKA, PEIJA HAARAMO, OSSI RAHKONEN and BØRGE SIVERTSEN | Joint associations of sleep duration and insomnia symptoms with subsequent sickness absence: The Helsinki Health Study |
| 2013 | 41 | 5 | 524-530 | JUSSI PULJULA, SAARA LESONEN, MARJA-LEENA KORTELAINEN, SEPPO JUVELA and MATTI HILLBOM | Mortality from traumatic brain injury after reduction of alcohol prices: A population-based study from northern Finland |
| 2013 | 41 | 5 | 531-540 | LONE ROSS, BIRTHE LYKKE THOMSEN, SIDSEL HELLE BOESEN, KIRSTEN FREDERIKSEN, RIKKE LUND, CHRISTIAN MUNK, SUSANNE OKSBJERG DALTON, PERNILLE ENVOLD BIDSTRUP, SUSANNE KRÜGER KJÆR, ANNE TJØNNELAND and CHRISTOFFER JOHANSEN | Social relations and smoking abstinence among ever-smokers: A report from two large population-based Danish cohort studies |
| 2013 | 41 | 5 | 541-545 | SAARA M. KOTILA, TARJA PITKÄNEN, JON BRAZIER, ERKKI EEROLA, JARI JALAVA, MARKKU KUUSI, EIJA KÖNÖNEN, JANNE LAINE, ILKKA T. MIETTINEN, RISTO VUENTO and ANNI VIROLAINEN | "Clostridium difficile" contamination of public tap water distribution system during a waterborne outbreak in Finland |
| 2013 | 41 | 5 | 546 |  | News from the National Institutes of Public Health |
| 2013 | 41 | 5 | 547 |  | Public Health Calendar - Public Health Events |
| 2013 | 41 | 6 | 549 | INGVAR KARLBERG | A unique institution to be closed down, and the consequences for the Journal |
| 2013 | 41 | 6 | 550-552 | YAN LI | A perspective on health care for the elderly who lose their only child in China |
| 2013 | 41 | 6 | 553-559 | ELVIIRA LEHTO, HANNA KONTTINEN, PEKKA JOUSILAHTI and ARI HAUKKALA | The role of psychosocial factors in socioeconomic differences in physical activity: A population-based study |
[truncated: 200,961 more chars]
